# Research on multi-path dense networks for MRI spinal segmentation

**DOI:** 10.1371/journal.pone.0248303

**Published:** 2021-03-12

**Authors:** ShuFen Liang, Huilin Liu, Chen Chen, Chuanbo Qin, FangChen Yang, Yue Feng, Zhuosheng Lin

**Affiliations:** Faculty of Intelligent Manufacturing, Wuyi University, Jiangmen, Guangdong, China; University of Engineering & Technology, Taxila, PAKISTAN

## Abstract

Accurate and robust segmentation of anatomical structures from magnetic resonance images is valuable in many computer-aided clinical tasks. Traditional codec networks are not satisfactory because of their low accuracy of edge segmentation, the low recognition rate of the target, and loss of detailed information. To address these problems, this study proposes a series of improved models for semantic segmentation and progressively optimizes them from the three aspects of convolution module, codec unit, and feature fusion. Instead of the standard convolution structure, we apply a new type of convolution module for the feature extraction. The networks integrate a multi-path method to obtain richer-detail edge information. Finally, a dense network is utilized to strengthen the ability of the feature fusion and integrate more different-level information. The evaluation of the Accuracy, Dice coefficient, and Jaccard index led to values of 0.9855, 0.9185, and 0.8507, respectively. These metrics of the best network increased by 1.0%, 4.0%, and 6.1%, respectively. Boundary F1-Score reached 0.9124 indicating that the proposed networks can segment smaller targets to obtain smoother edges. Our methods obtain more key information than traditional methods and achieve superiority in segmentation performance.

## Introduction

With the continuous development of society, people have become increasingly busy, and various pressures of daily life and diseases (e.g., low back pain) have been discovered [1]. Over four-fifths of the population is suggested to suffer from such diseases [2].

Spine problems have become one of the most common and urgent health problems in modern society. With the development of computer and digital information technologies, people have increasingly focused on the acquisition and analysis of medical images. To improve the feasibility of diagnosis and treatment before clinical diagnosis or spinal surgery, a doctor can prioritize clinical analysis based on the patient’s medical image and efficiently obtain more accurate clinical information from the segmented spinal image [3].

Magnetic resonance imaging (MRI) is known as the most sensitive non-invasive medical image technique with an outstanding effect on the spinal structure [4]. With the application of computer-aided diagnosis in the field of clinical diagnosis, doctors and scholars have increased visual research on spinal MRI. However, achieving the required segmentation accuracy of the target is difficult because of the complex structure and variable shapes of the human spine and the similarity of bone structures in other regions.

Several researchers have proposed many methods to achieve accurate spine segmentation, including edge information-based segmentation [5], threshold-based segmentation [6], region growing [6–9], active contour-based segmentation [10], and clustering-based segmentation [11, 12]. However, segmentation efficiency is limited because of the complexity of the traditional method and noise from imaging devices.

Image segmentation research is constantly evolving with the development of deep learning [13]. Compared with traditional segmentation methods, the deep convolutional network is characterized by automatic feature extraction, which can achieve end-to-end training results. Image segmentation algorithms based on deep learning mainly include the fully convolutional network (FCN) [14], U-type network (U-Net) [15], V-type network (V-Net) [16], and SegNet [17].

End-to-end and pixel-to-pixel convolutional neural networks are demonstrated as superior to the most advanced semantic segmentation methods during their time. Their architecture uses multilayer common convolution and pooling operations interchangeably. Finally, two transformations are implemented: a transformation of the classification network to a segmentation network and image-level classification to pixel-level classification. The operated objects of the abovementioned models are mostly concentrated in a local area of the input image, and the processing is similar to the sliding window model [17–20].

Olaf Ronneberger et al. [15] proposed a more concise network structure based on the FCN structure using a U-Net segmentation model. U-Net is an extraordinary, advanced, and popular network model in the semantic segmentation of medical images. It comprises two parts, namely encoding and decoding parts. The down-sampling operation in the encoding part decreases the feature map resolution and increases the number of feature map channels to obtain the feature maps of different dimensions. The up-sampling operation in the decoding part reduces the number of feature map channels and gradually recovers the feature map resolution. Meanwhile, U-Net has an FCN-like architecture that employs skip connection to avoid losing details. The output segmentation map is then generated.

The U-Net architecture has three main advantages of segmentation tasks. First, the model can handle global and local semantic information at the same time. Second, for an insufficient training dataset, it can also be used for training to obtain an ideal result with a small number of samples. Third, end-to-end segmentation training delivers the entire information to the next step and directly generates a segmentation map such that the network can save the complete semantic information of the input images. Many researchers have used this model for MRI segmentation. Norman Berk et al. [21] used U-Net and its variant networks to segment human knee joint images. Consequently, they obtained more accurate and efficient segmentation results. Some researchers have also proposed a spinal CT image segmentation method based on U-Net [22, 23]. Gu et al. [24] integrated the proposed dense atrous convolution (DAC) and residual multi-kernel pooling (RMP) blocks into an encoder-decoder structure to capture more high-level features and preserve more spatial information.

In recent years, researchers have constantly tried improving the U-Net model. He et al. [25] proposed a new method that substitutes an ordinary convolution module with a residual network (ResNet). The method neither introduces additional model parameters nor increases the computational complexity of the model. ResNet is also utilized to prevent gradient vanishing. The generalized module consists of two layers of input information with a 3×3 kernel, a stride size of 1, and a dilation rate of 1. The sum of the results and the input information is calculated as the model output.

The dense network, DenseNet [26], utilizes a more concise feature connection based on ResNet. The overall architecture of DenseNet is equivalent to that of ResNet, but note that the former uses a large number of skip connections to improve image feature utilization and enhance the semantic segmentation effect. It sums up the output of all previous layers by a concatenation operation; hence, it can obtain the subsequent layer input. The DenseNet function is modularized to obtain more universal convolutional units and achieve generalizability. The generalized module consists of two layers of input information with a 3×3 kernel, a stride size of 1, and a dilation rate of 1. A cross-fusion of information from each layer is used as the final input of each operation level.

However, the encoder-decoder network has some disadvantages. U-Net, which is an encoder-decoder model, finds it difficult to recover all the feature information of the input images after obtaining the segmentation result by upsampling. The disadvantage of fewer datasets for medical image segmentation has always existed; therefore, inadequate samples have led to overfitting.

Considering the problems of existing spinal segmentation methods, this study proposes a new convolutional architecture based on typical codec networks. This architecture has been improved across three aspects: convolution module, codec unit, and feature fusion. The experimental results illustrate that our method achieves more accurate segmentation results than traditional methods.

The main contributions of this work are summarized as follows:

1. We propose a new multi-path dense network for capturing more high-level features and preserving more detailed information.
2. We improve the encoder-decoder structure in three aspects: convolution module, multi-path network, and feature fusion.
3. We apply the proposed method in spine segmentation, with the results showing that the proposed method outperforms state-of-the-art methods.

The remainder of this paper is organized as follows: Section 2 reviews some recent lumbar spine segmentation methods and lumbar spine datasets; Section 3 introduces and analyzes the proposed method in detail; Section 4 presents the experimental results; Section 5 provides relevant discussions and Section 6 draws some conclusions.

## Literature review

Researchers have considered deep learning as a rising subset of machine learning techniques [27]. Rather than using pre-defined hand-crafted features, deep neural networks can learn hierarchical features thoroughly from the input images [28]. Automated and semi-automated detection and segmentation of spinal and vertebral structures from MRI is a challenging task due to a relatively high degree of anatomical complexity [29]. The main problem is the presence of unclear boundaries and articulation of vertebrae with each other [30].

In recent years, several deep learning-based methods for vertebra segmentation have been developed. Robert Korez et al. [31] designed a novel framework for the automated spine and vertebrae detection and segmentation from three-dimensional (3D) computed tomography images. Subsequently, they proposed an automated method for supervised segmentation of vertebral bodies from 3D MRI that is based on coupling deformable models with convolutional neural networks [29]. Marko Rak et al. [32] proposed an automatic approach for fast vertebral body segmentation in 3D MRI of the whole spine. Jose Dolz et al. [33] proposed an architecture based on U-Net for intervertebral disc localization and segmentation in multi-modal MRI, contributing to better data representation and discriminative power. Li et al. [34] presented a novel multi-scale and modality dropout learning framework to locate and segment the spine from four-modality MRI. Dominik GaweB et al. [35] combined multiple stages of deep learning to recognize and separate different tissues of the human spine. Faisal Rehman1 et al. [36] presented a novel combination of the traditional region-based level set with deep learning framework in order to predict shape of vertebral bones accurately. Martin Kolarík et al. [37] designed a 3D Dense-U-Net neural network architecture implementing densely connected layers for high-resolution 3D volumetric segmentation of medical image data.

Neural networks with deep layers contain enormous parameters and large-scale datasets can be used for avoiding over-fitting [28]. Additionally, novel- and well-constructed datasets can push deep learning research forward in various areas. The most widely used spine segmentation dataset is the MICCAI Vertebrae Segmentation Challenge [38] and xVertSeg Challenge [39].

## Methods

### Convolution module

**Fig 1:**
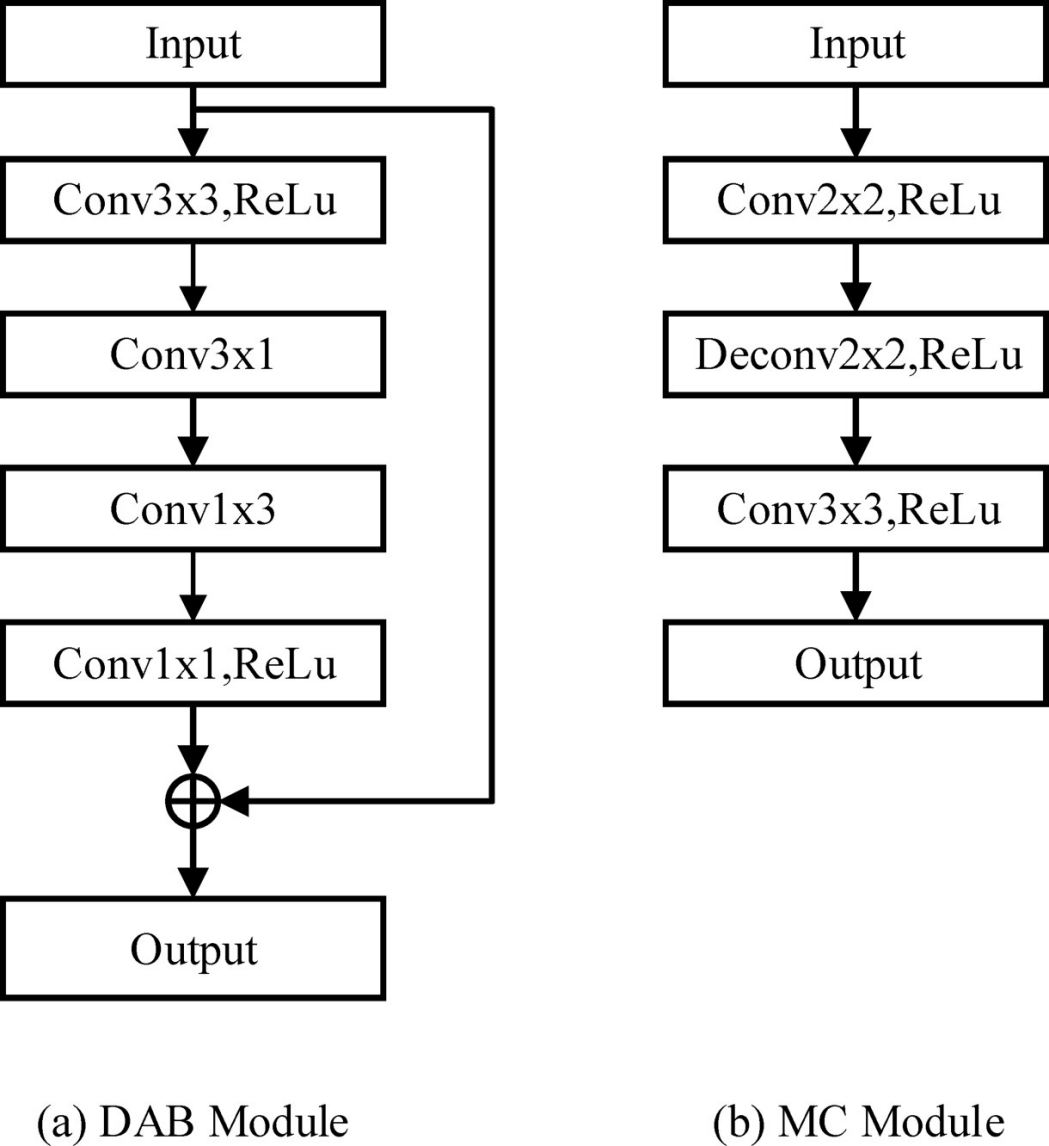
Two convolutional architectures. On the left is the DAB and on the right is the MC.

Common convolutional structures have limited ability when it comes to obtaining input image features; hence, based on the standard convolutional module, depth-wise asymmetric bottleneck (DAB), which is a deep separable convolutional structure, was constructed [40]. DAB can extract more detailed information about the target and improve the ability to extract image features. The special structure not only incorporates a one-dimensional convolution structure for dimensionality reduction but also includes a residual structure. It is mainly used to balance the accuracy and the running speed of image processing. The structure can also effectively compress data and improve network efficiency and performance. Fig 1 shows that the DAB structure employs a standard convolutional layer as input and puts it in two grouped convolutional layers decomposed into two-layer convolutional layers with kernel sizes of 1×3 and 3×1. The 3×3 convolution is divided into two convolutions with kernel sizes of 1×3 and 3×1 to reduce the calculation. DAB then applies a 1×1 kernel as the output. The residual structure is introduced as part of the output.

The spine structure includes massive blocks and detailed branches [41]. We obtain more detailed information by employing a micro codec (MC) convolutional module (see Fig 1), whose structure is similar to that of the general convolutional module. Three convolutional layers are considered, and one of which has a kernel size of 2×2, a stride size of 1, and a dilation rate of 0. The second section applies a deconvolution operation, whose parameters are similar to those of the first section. The last layer uses a common convolution with a kernel size of 3×3, a stride size of 1, and a dilation rate of 1. Each layer has its activation unit. Adding a deconvolutional unit after the common-convolution operation can effectively accumulate the semantic features and detailed information of the previous layer.

The abovementioned convolutional module has strong adaptability and can be embedded in most deep neural networks; thus, we can derive different benefits from a variety of convolutional modules.

### Codec unit

In the encoder-decoder structure, the encoder and the decoder correspond to each other. In other words, the spatial scale and the number of channels of a coding unit are related to their corresponding decoding unit to enhance the image input data and simplify the preprocessing on the training network.

**Fig 2:**
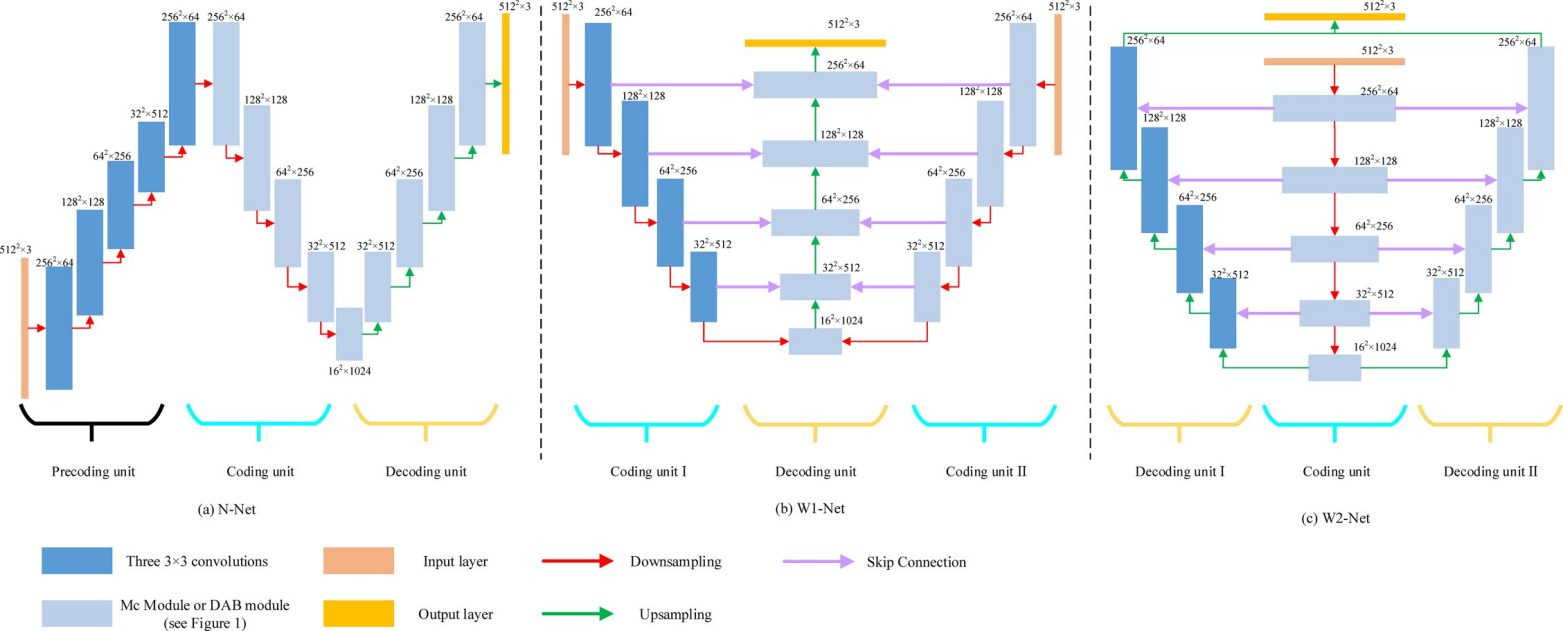
Three network architectures. From left to right: MCN-Net, MCW1-Net, and MCW2-Net, respectively. The downsampling is a max-pooling operation and the upsampling is a bilinear interpolation.

We take the combination of the MC convolution module as an example. Fig 2 depicts the three newly proposed multipath codec segmentation networks: MC N-type network (MCN-Net); MC W-type network1 (MCW1-Net); and MC W-type network2 (MCW2-Net). N-Net consists of a precoding unit, a coding unit, and a decoding unit. W1-Net is composed of two coding units and one decoding unit. W1-Net has composed of one coding unit and two decoding units.

Based on the N-type network, MCN-Net (see Fig 2) substitutes common convolutional modules with MC convolutional modules. Precoding is a feature extraction structure, in which the convolutional and pooling layers alternately operate and resize the image to its original size. Finally, the pre-encoded result is used as the input of the typical U-Net. The overall network model is completed after making appropriate adjustments in the network structure.

Subsequently, the MCW1-Net (see Fig 2) is proposed considering the effect of combining image features from different feature extractors. A new coding unit is added based on the convolution module that retains the original coding unit. The output result is then merged into the down-sampling result of the last layer. Both the added and decoding units apply MC convolutional modules.

The convolution architecture has a smaller effect on image segmentation when the feature extraction of the encoder reaches a certain level in the contractive path [14]. Furthermore, increasing the efficiency is useful in recovering the image resolution and accuracy of the classification label in each pixel [17]. We adopt MCW1-Net to design MCW2-Net (see Fig 2) for spine segmentation in MRI. The network retains the convolution module in the original decoding unit and adds a new decoding unit, whose input is the output of the first up-sampling layer. Finally, the two feature outputs of the decoder are fused in the output layer. Because a single decoding unit will produce errors in the process of restoring resolution [42], MCW2-Net is applied two decoding units to decrease the probability of deviation and improve the accuracy.

### Feature fusion

The convolutional operation in a deep neural network is the feature extraction of the input data. The convolution kernel of any layer in the operation layer is the corresponding feature extractor. It can extract shape, color, and other characteristics from the input image or the feature layer of the middle layer. The codec network uses two common feature fusion methods: shortcut connection [25] and skip connection [15]. Both feature fusion methods integrate low-dimensional features containing high-level semantic information into high-dimensional feature layers with semantic information loss [43], the whose merged result is taken as the output data.

**Fig 3:**
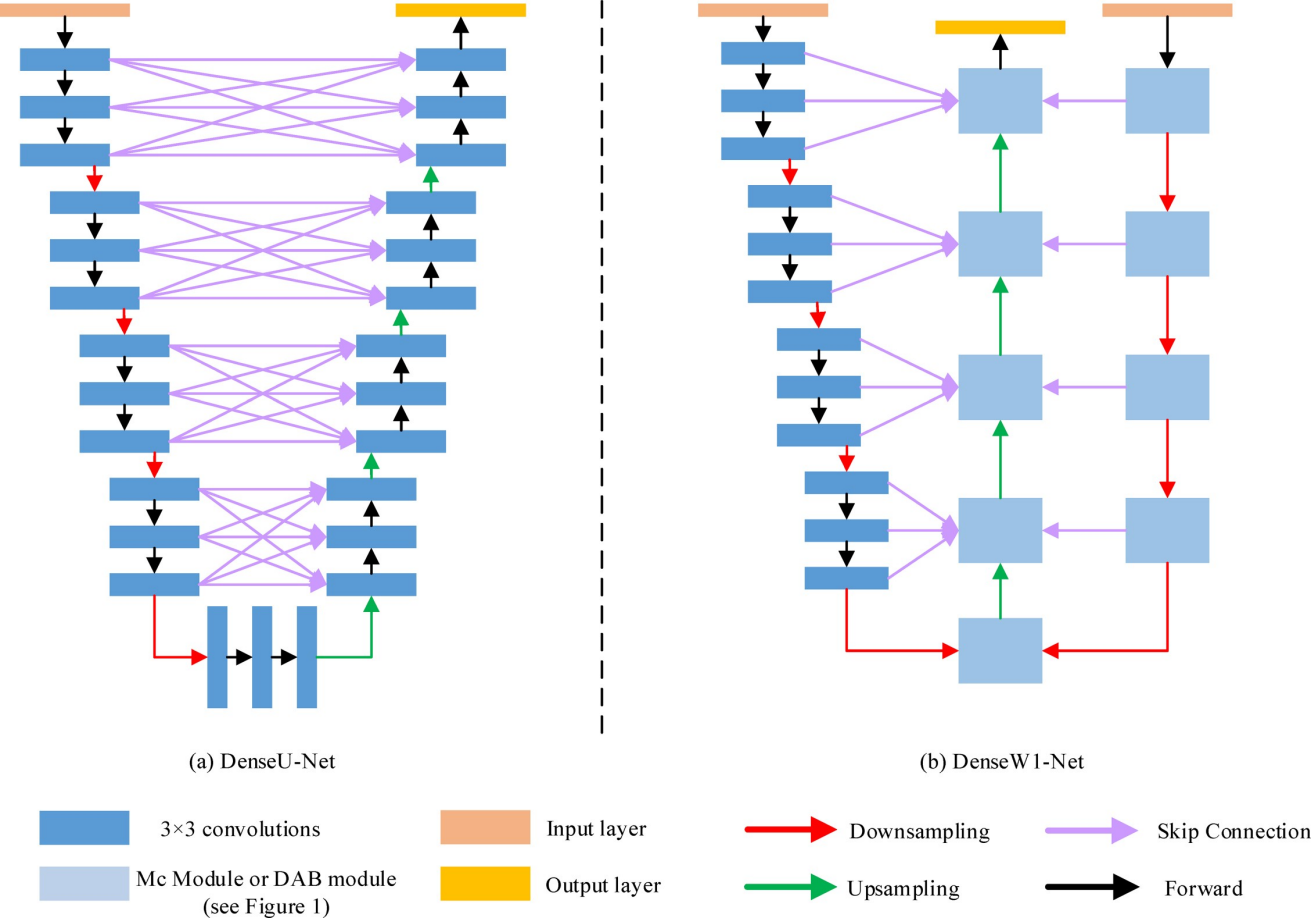
DenseXY-Net structure. On the left is the DenseU-Net and on the right is DenseMCW1-Net.

A new dense network, called DenseXY-Net (X: the type of convolution module; Y: network shape), which was constructed by a quick connection, was proposed to mitigate the effects of information loss combined with densely connected layers of the deep dense network [26]. The network was combined with the proposed convolutional modules and codec units, and the dense feature fusion was realized by a concatenate function (see Fig 3). The new DenseXY-Net concatenate the outputs of each convolutional layer in each coding unit with the corresponding feature maps of the decoding unit.

The result of the convolution unit in each layer was saved and put into the dense skip connection. Finally, the fusion features were cross-computed and merged into the feature convolution unit of the classic U-Net. It can minimize the loss of information and obtain more information in each convolutional layer.

DenseMCW1-Net was taken as an example. Fig 3 depicts its structure. The left side of the W type is the precoding structure using the ordinary convolution module, whereas the right side is the coding structure using four MC modules for down-sampling operation. The left and right sides are concentrated feature maps. The output map is obtained from the decoding structure in the middle path. In this process, the encoding structure copies its information to the decoding structure using skip connections. Finally, the decoding structure can obtain a higher feature resolution and richer low-level information, which are beneficial for the restoration of the target contours and segmentation mask generation.

## Results

### Experimental setup

#### Dataset

The experimental database was derived from SpineWeb’s high-anisotropy MRI images of the lower back [44, 45], including 210 cases of MRI images of the human lower lumbar vertebrae and 50 cases of MRI images of the human cross-sectional spine. After removing the original images without segmentation targets and their corresponding labels, the database contained 2460 sets of original images and their corresponding labels. The sizes of the image ranged from 512×512 to 1024×1024 px, and most of which were 880×880 px. Among the remaining sets, 200 samples were randomly selected as the small dataset and divided into the training, verifying, and testing sets with a ratio of 8:1:1. The images were resized to 512×512 px and subsequently, data augmentation was applied to avoid the model overfitting, including rotation, flip, translation, and mirroring. The changed spine images and their labels are used as input images into the proposed network.

#### Experiment settings

During the training, we utilized the Adam optimizer [46] to train our networks and its hyperparameters are set to the default values, where the initial learning rate lr = 2e-3, betas = (0.5, 0.999). The maximum epoch is 300. The loss function is Binary Cross-Entropy (BCE) loss function [47], which is defined as:

$$L_{BCE}\left(y,\hat{y}\right)=-\left(y\log\left(\hat{y}\right)+\left(1-y\right)\log\left(1-\hat{y}\right)\right)$$ (1)

Here, $\hat{y}$ is the predicted value by the prediction model. It is widely used for classification objectives as semantic segmentation is pixel-level classification [48].

The implementation was based on the public PyTorch platform. The training and testing bed was Windows 10 system with an NVIDIA GeForce RTX 2080 TI graphics card.

### Quantitative evaluation metrics

Six different evaluation metrics are employed to assess the performance of segmentation results: accuracy, sensitivity, specificity, precision, Jaccard similarity, Dice coefficient, and Boundary F1-Score (BF-Score), as defined in Table 1. These metrics were all expressed through the calculated TP (true positives), FP (false positives), FN (false negatives), and TN (true negatives). BF-Score is calculated from precision and recall values with a distance threshold to decide whether a boundary point has a match or not [49]. It is experimentally found that the distance threshold is set to 2 which is suitable for the evaluation. Besides, the parameters of the networks are applied to compare differences in network complexity.

**Table 1:** Six different evaluation metrics.

Evaluation Metrics	Formulas
Accuracy	$\frac{TP+TN}{TP+TN+FP+FN}$
Sensitivity	$\frac{TP}{TP+FN}$
Specificity	$\frac{TN}{FP+TN}$
Precision	$\frac{TP}{TP+FP}$
Jaccard similarity	$\frac{TP}{TP+FN+FP}$
Dice coefficient	$\frac{2\times TP}{2\times TP+FP+FN}$
BF-Score	$\frac{2\times Precision\times Sensitivity}{Precision+Sensitivity}$

### Analysis

The experiment was performed via inputting, preprocessing (such as data augmentation), model training, postprocessing, and outputting. Under the same system, different network models were used to train the model for the pixel-level segmentation of medical MRI spine images to distinguish the target spine from the background. After preprocessing, the output image of the segmented spine was converted to a binary image. Subsequently, we obtained the evaluation metrics of spine segmentation by each network using the abovementioned indices.

#### Convolution module

**Fig 4:**
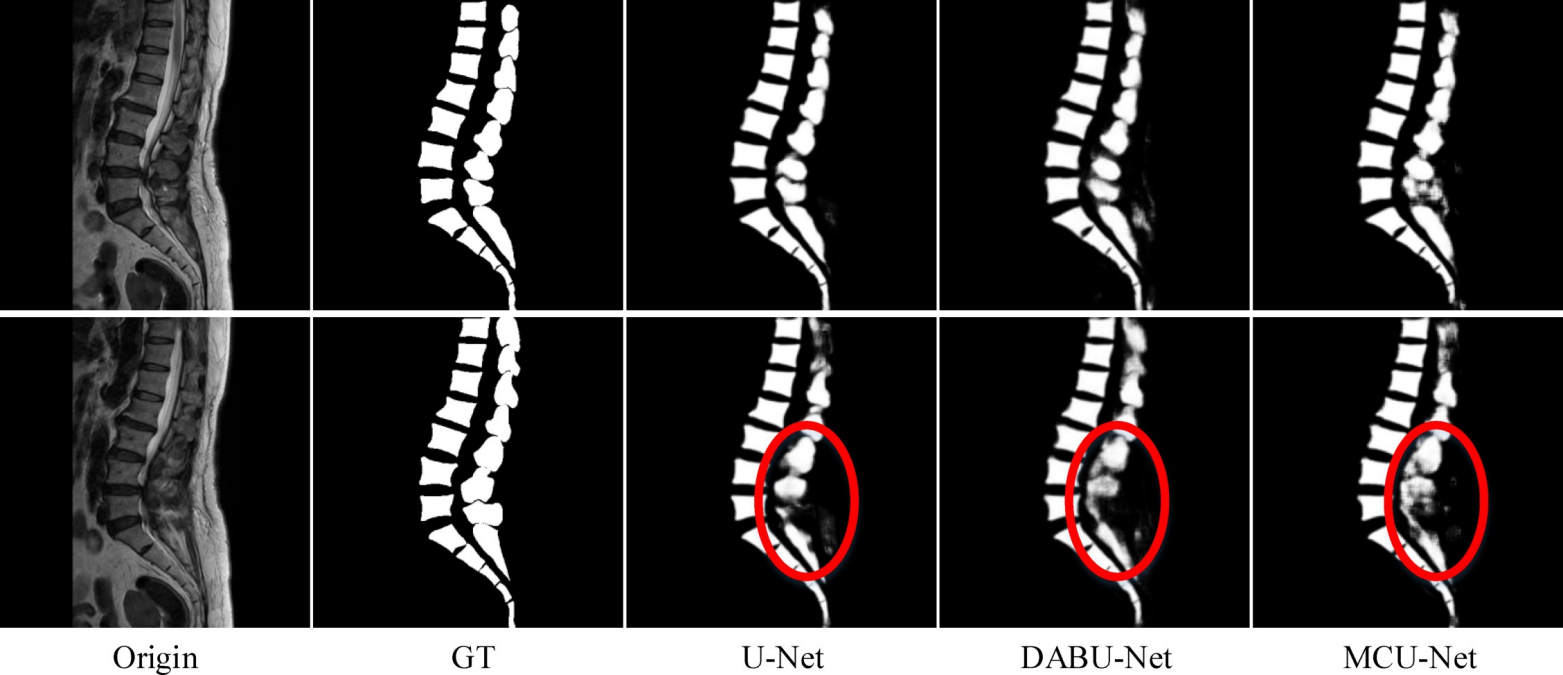
Sample results of spine segmentation (two adjacent slices). From left to right: original images, ground truth, U-Net, DABU-Net, and MCU-Net, respectively.

As illustrated in Fig 4, the original images, labels, and predictions were made through the proposed methods. In the labels and results, the white areas denoted the area where the vertebrae lie in the original image, and the black areas were the background. Compared with the prediction results, the designed segmented network was beneficial for describing the features in medical spine images and represents more comprehensive details. In addition, some models had different degrees of loss information during the experimental prediction processing. Some information on the spine (i.e., white areas in Fig 4) was missed in the segmentation results. Only changing the convolution module did not achieve good performance.

In the quantitative analysis of the spinal segmentation experiment, we compared the results of the convolution modules in the training sets. Table 2 described the evaluation. The table recorded the evaluation metrics of three different types of neural network models on the small experimental dataset. DABU-Net and MCU-Net replaced the ordinary convolution block with the DAB and MC modules, respectively. A comparison of the performance evaluations in the tables indicated that the proposed convolution module produced different degrees of effects on different models. There was almost no difference in their network parameters. The DAB module achieved a good improvement in Dice and Jaccard, that was, 0.8916 and 0.8069, respectively. Both the DAB and MC modules obtained better results on the indices. The performance was relatively boosted, and the robustness was high. There was little difference in their total parameters. However, the BF-Score of DABU-Net and MCU-Net did not have obvious improvement. As shown in Fig 4, the segmentation results of the spine (in the red circle) were not good, whose edges were not clear. Therefore, we designed a new multipath structure and combined it with the convolution module to improve the segmentation performance.

**Table 2:** Evaluation metrics of the convolution module.

Model Type	Accuracy	Sensitivity	Specificity	Precision	Dice Coefficient	Jaccard	BF-Score	Parameters
U-Net	0.9749	0.8267	0.9947	0.9478	0.8785	0.7899	0.8809	131M
DABU-Net	0.9772	0.8492	0.9939	0.9431	**0.8916**	**0.8069**	**0.8924**	123M
MCU-Net	0.9765	0.8408	0.9941	0.9450	0.8874	0.8009	0.8921	131M

#### Codec unit

**Fig 5:**
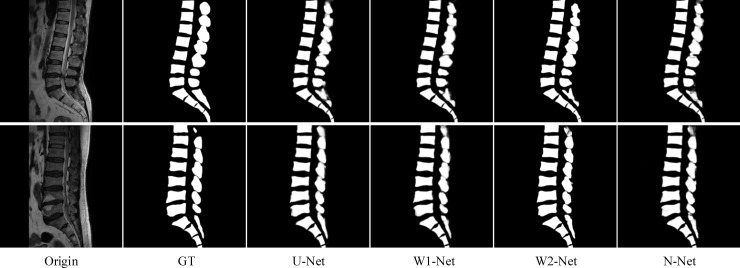
Sample results of spine segmentation (two adjacent slices). From left to right: original images, ground truth, U-Net, W1-Net, W2-Net, and N-Net, respectively.

Three new multi-path encoding and decoding structures were proposed (i.e., W1-Net, W2-Net, and N-Net) based on the classic U-Net encoding and decoding network. Fig 5 showed some examples for a visual comparison. A rough spine contour can be identified and segmented when comparing the mask with the ground truth.

Table 3 showed a comparison of different structures. The proposed structures were better than U-Net. The Jaccard of all three new networks can be higher than 0.81. A comparison showed that W1-Net achieved 0.9830, 0.9042, and 0.8267 of accuracy, Dice, and Jaccard, respectively, which were better than those obtained by the other methods. Compared with U-Net, the Dice of the best structure increased from 0.8785 to 0.9042 by 2.6%, while the Jaccard increased from 0.7899 to 0.8267, showing that the proposed W1-Net was beneficial for spine segmentation. The parameters of the multipath networks were increased, but their BF-Scores are higher than that of U-Net. W1-Net can achieve 0.8827 and its edges of the segmented result are smoother.

**Table 3:** Evaluation metrics of the multipath networks.

Model Type	Accuracy	Sensitivity	Specificity	Precision	Dice Coefficient	Jaccard	BF-Score	Parameters
U-Net	0.9749	0.8267	0.9947	0.9478	0.8785	0.7899	0.8351	131M
W1-Net	0.9830	0.9201	0.9892	0.8912	**0.9042**	**0.8267**	**0.8827**	161M
W2-Net	0.9817	0.9186	0.9880	0.8796	0.8971	0.8155	0.8715	191M
N-Net	0.9814	0.9101	0.9884	0.8841	0.8953	0.8123	0.8801	181M

**Fig 6:**
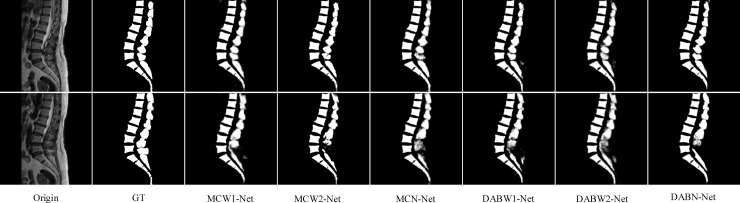
Sample results of spine segmentation (two adjacent slices). From left to right: original images, ground truth, MCW1-Net, MC W2-Net, MC N-Net, DABW1-Net, DABW2-Net, and DABN-Net, respectively.

The codec unit was used to experiment on the same dataset. Table 3 listed the five indices of the neural network segmentation models. The networks used different codec paths as the model frameworks for spine image segmentation. The MC and DAB modules were applied to form six hybrid networks. The visual comparison of the networks showed in Fig 6.

Table 4 showed that the N-type multi-path network architecture only slightly improved the spine segmentation BF-Score. The evaluation did not change much compared with the improved convolution modules. In contrast, W1-Net with the MC module was found to greatly improve the Jaccard, Dice coefficients, and BF-Score, which was valuable in continuous research.

**Table 4:** Evaluation metrics of the combination.

Model Type	Accuracy	Sensitivity	Specificity	Precision	Dice Coefficient	Jaccard	BF-Score	Parameters
MCW1-Net	**0.9847**	**0.9279**	0.9903	0.9010	**0.9135**	**0.8420**	**0.8925**	**155M**
MCW2-Net	0.9840	0.9155	**0.9908**	0.9052	0.9095	0.8352	0.8885	185M
MCN-Net	0.9796	0.8981	0.9875	0.8755	0.8853	0.7956	0.8458	182M
DABW1-Net	0.9844	0.9037	0.9921	**0.9187**	0.9099	0.8360	0.8912	155M
DABW2-Net	0.9838	0.9217	0.9899	0.8967	0.9082	0.8328	0.8844	189M
DABN-Net	0.9845	0.9228	0.9905	0.9042	0.9125	0.8402	0.8687	174M

#### Feature fusion

**Fig 7:**
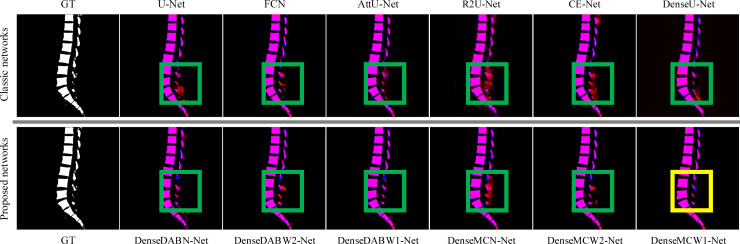
Sample results of classic and proposed networks. Top row: Examples of the spine were tested in the six classic methods (U-Net, FCN, AttU-Net, R2U-Net, CE-Net, DenseU-Net, respectively). Bottom row: the six proposed DenseX-Net.

The experiment was performed to compare the feature fusion results (see Table 5). We compared the proposed DenseXY-Net to some classical deep-learning-based methods [12, 13, 21]. The dense structure provided the network with more complete details of layers, as shown in Fig 7. The edge results of DenseMCW1-Net (in the yellow rectangle) were better than those of other networks (in the green rectangles). The ground truth (blue) and the segmented result(red) are overlapped into one image (purple). It can be seen from Fig 7 that the classic networks have fewer purple areas (overlapped areas) in the green rectangles. DenseMCW1-Net can segment a smaller target and obtain smoother edges.

**Table 5:** Evaluation metrics of feature fusion.

Model Type	Accuracy	Sensitivity	Specificity	Precision	Dice Coefficient	Jaccard	BF-Score	Parameters
U-Net[15]	0.9749	0.8267	**0.9947**	**0.9478**	0.8785	0.7899	0.8351	131M
FCN [14]	0.9828	0.9297	0.9882	0.8828	0.9044	0.8266	0.8542	992M
AttU-Net [50]	0.9854	0.9116	0.9927	0.9245	0.9171	0.8475	0.8972	133M
R2U-Net [51]	0.9760	0.8901	0.9851	0.8494	0.8674	0.7669	0.8068	149M
CE-Net [24]	0.9828	**0.9426**	0.9869	0.8762	0.9067	0.8305	0.8592	110M
DenseU-Net	0.9827	0.9260	0.9885	0.8884	0.9055	0.8287	0.8831	271M
DenseDABN-Net	0.9806	0.9120	0.9874	0.8734	0.8908	0.8049	0.9001	299M
DenseDABW2-Net	0.9835	0.9235	0.9892	0.8955	0.9079	0.8330	0.8909	289M
DenseDABW1-Net	0.9840	0.9274	0.9897	0.8965	0.9107	0.8369	0.8972	279M
DenseMCN-Net	0.9846	0.9285	0.9902	0.9011	0.9133	0.8421	0.8730	295M
DenseMCW2-Net	0.9849	0.9184	0.9914	0.9113	0.9140	0.8429	0.9119	287M
DenseMCW1-Net	**0.9855**	0.9316	0.9908	0.9075	**0.9185**	**0.8507**	**0.9124**	283M

Compared with typical networks, such as U-Net, the proposed network for spine segmentation improved the accuracy, Jaccard similarity index, and Dice coefficient. In particular, using the MC module and the W1-type structure had a superior effect on image segmentation and capturing feature details. The segmentation results of the proposed method were more similar to the ground-truth maps than those of other techniques. In addition, the new DenseXY-Net based on U-Net achieved better accuracy than the traditional methods.

DenseMCW1-Net had 0.9855, 0.9185, and 0.8507 of accuracy, Dice coefficient, and Jaccard similarity index, respectively. These values were better than those obtained by other methods. More detailed vertebral bodies can be segmented and their edges were clearer. Its BF-Score greatly exceeded that of ordinary codec networks, but its number of parameters was much higher than those of other classic models. The Jaccard similarity index was also greatly improved. The Dice coefficient and BF-Score increased by nearly 3% and 8%, respectively. In other words, DenseMCW1-Net was beneficial for spine segmentation. Moreover, other hybrid methods with a dense structure can obtain better results than the previously proposed methods.

**Fig 8:**
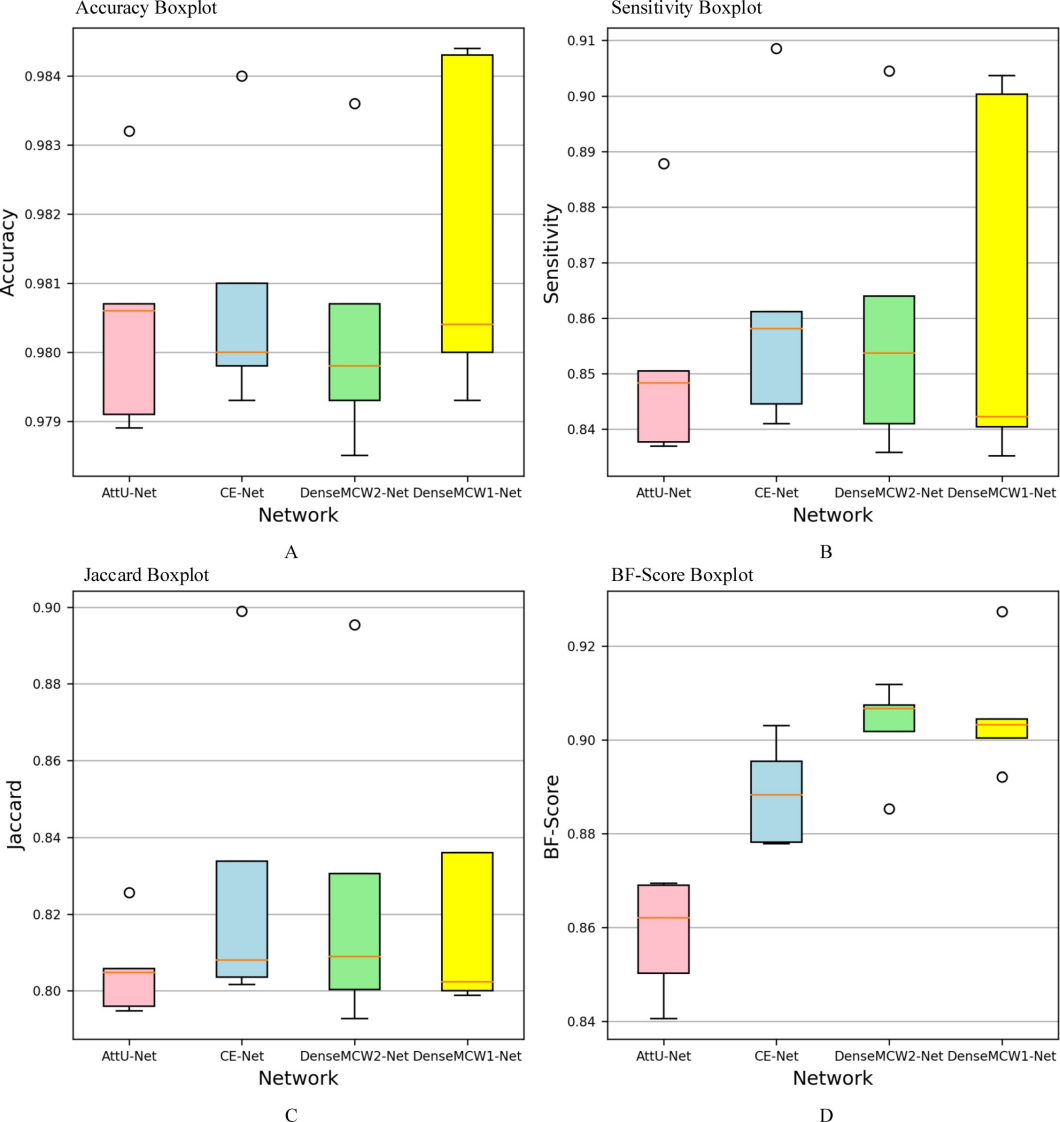
Boxplots of classic and proposed networks. (A) Accuracy boxplots for four methods over five-fold cross-validation, (B) Sensitivity boxplots for four methods over five-fold cross-validation, (C) Jaccard boxplots for four methods over five-fold cross-validation, (D) BF-Score boxplots for four methods over five-fold cross-validation.

The cross-validation approach is used to evaluate the performance of the network and obtain as much valid information as possible from the small dataset. We chose respectively the four best classic and proposed networks (AttU-Net, CE-Net, DenseMCW2-Net, and DenseMCW1-Net) and applied a five-fold cross-validation approach. The results were listed in Table 6. Four boxplots of classic and proposed networks over the five-fold cross-validation were also shown in Fig 8. It can directly visualize the different performances of different networks. From the table and figure, it can be observed that the performance of the proposed DenseMCW1-Net is the best on metrics among all the compared methods for spine segmentation. In Fig 8(A)–8(C), the three networks (AttU-Net, CE-Net and DenseMCW2-Net) have outliers and the proposed DenseMCW1-Net has little changes. On the contrary, the proposed network in Fig 8(D) has two outliers, but it can be seen that the values are higher than those of the other three networks so the proposed network is significantly better than the other three networks. In Table 6, we found that the performance of the segmentation model is relatively good and stable. The overall accuracy is 0.9817 which proves the effectiveness of DenseMCW1-Net. The result of the network is similar to the above results. Second, Jaccard is 0.8346 and Dice coefficients is 0.8958, indicating that DenseMCW1-Net have relatively large overlapped areas (ground true and predicted image). Finally, the overall BF-Score on the spine image dataset is 0. 9055, which proves that the proposed DenseMCW1-Net can segment clearer edges.

**Table 6:** Five-fold cross-validation results of networks.

Model	Fold	Accuracy	Sensitivity	Specificity	Precision	Dice Coefficient	Jaccard	BF-Score
**AttU-Net**	Fold 1	0.9791	0.8377	0.9947	0.9442	0.8825	0.7948	0.8405
	Fold 2	0.9806	0.8505	0.9947	0.9433	0.8900	0.8058	0.8621
	Fold 3	0.9807	0.8369	0.9964	0.9577	0.8895	0.8048	0.8694
	Fold 4	0.9789	0.8483	0.9933	0.9332	0.8845	0.7960	0.8502
	Fold 5	0.9832	0.8878	0.9931	0.9258	0.9027	0.8256	0.8690
	Average	0.9805	0.8522	0.9944	0.9408	0.8898	0.8054	0.8582
**CE-Net**	Fold 1	0.9800	0.8581	0.9937	0.9323	0.8882	0.8036	0.8883
	Fold 2	0.9798	0.8410	0.9950	0.9445	0.8848	0.8989	0.8782
	Fold 3	0.9810	0.8445	0.9959	0.9521	0.8917	0.8080	0.8954
	Fold 4	0.9793	0.8612	0.9926	0.9248	0.8875	0.8017	0.8779
	Fold 5	0.9840	0.9086	0.9918	0.9130	0.9078	0.8338	0.9030
	Average	0.9808	0.8627	0.9938	0.9333	0.8920	0.8292	0.8886
**DenseMCW2-Net**	Fold 1	0.9793	0.8358	0.9951	0.9478	0.8822	0.8954	0.9067
	Fold 2	0.9807	0.8640	0.9933	0.9317	0.8921	0.8089	0.9018
	Fold 3	0.9798	0.8537	0.9937	0.9317	0.8868	0.8003	0.9074
	Fold 4	0.9785	0.8410	0.9939	0.9368	0.8816	0.7927	0.8853
	Fold 5	0.9836	0.9045	0.9919	0.9140	0.9060	0.8306	0.9118
	Average	0.9804	0.8598	0.9936	0.9324	0.8897	0.8256	0.9026
**DenseMCW1-Net**	Fold 1	0.9844	0.9037	0.9921	0.9187	0.9099	0.8360	0.8921
	Fold 2	0.9800	0.8352	0.9927	0.9521	0.8853	0.7988	0.9044
	Fold 3	0.9804	0.8422	0.9955	0.9478	0.8880	0.8024	0.9032
	Fold 4	0.9793	0.8404	0.9950	0.9452	0.8864	0.8000	0.9004
	Fold 5	0.9843	0.9003	0.9931	0.9248	0.9095	0.8360	0.9274
	Average	0.9817	0.8644	0.9937	0.9376	0.8958	0.8346	0.9055

The experimental results illustrate that the sensitivity of the DAB module combined with different structures was slightly higher than that of the typical U-Net. Meanwhile, the MC module used in the different structures mainly strengthened the Jaccard and Dice coefficients. Compared with the N- and U-type networks, the W1-Net architecture made great progress in the evaluation metrics. Besides, the Specificity and the Dice coefficient slightly improved. For the proposed network structure, the DenseXY-Net architecture had advantages in terms of the Jaccard similarity index and BF-Score.

## Discussion

A series of improved models for segmentation of the MRI spinal images was proposed in this study. Compared with the traditional codec structure network, the improved models were optimized in three aspects: convolution module, codec unit, and feature fusion. The direction of improvement is a progressive relationship. First, the improved convolution module was utilized to replace the convolution module of the traditional codec network and capture feature information. The improved networks combined multiple codec units to obtain more detailed information. Finally, a dense network was applied to integrate multi-level information. As a result, the improved model can obtain more precise results in MRI spinal image segmentation.

We proposed herein two improved convolution modules: MC and DAB. The MC module contains three layers of convolution operations. The convolution kernels are 2×2, 2×2, and 3×3. The second layer is a deconvolution. It is employed to restore the lost information during the convolution process and capture more detailed information. The DAB convolution module decomposes the ordinary 3×3 convolution into two convolutions of 3×1 and 1×3 is used to obtain multi-scale feature information. Residual structures are useful in preventing gradient vanishing during the training process. The results in Table 2 showed that DABU-Net and MCU-Net both exhibited a slight improvement, but the mask indicated that some details were missing.

The effect of integrating different feature maps from different extractors should be considered to improve the efficiency of the recovery and classification accuracy of each pixel. The improved convolution module is not very useful in the segmentation process when the encoder reaches the limitation of feature extraction. Therefore, we designed and compared three new multi-path structures and added an encoding, decoding, and pre-encoding path to obtain more local information, such that the mask edge is smoother. The results in Table 3 showed that the three networks (i.e., W1-Net, W2-Net, and N-Net) significantly improved compared to U-Net. We then combined the two abovementioned modules and the three networks to form six hybrid models (i.e., MCW1-Net, MCW2-Net, MCN-Net, DABW1-Net, DABW2-Net, and DABN-Net). The results in Table 4 indicated that the hybrid networks were better than the three original networks. The segmentation result of the MCW1-Net was the best.

We obtained more detailed edge information herein by employing a dense network to strengthen the feature fusion ability and deliver more encoding information to the decoding structure. The encoding structure copied its information to the decoding structure using skip connections. Meanwhile, the decoding structure obtained a higher feature resolution and richer low-level information, which are beneficial to the restoration of the target contours and segmentation mask generation. Six dense hybrid networks (i.e., DenseMCW1-Net, DenseMCW2-Net, DenseMCN-Net, DenseDABW1-Net, DenseDABW2-Net, and DenseDABN-Net) were proposed. The proposed networks can detect and segment smaller targets. The results in Table 5 showed that the DenseMCW1-Net model had the best segmentation result.

Compared with the traditional segmentation method based on deep learning, the improved method no longer required a large number of training samples. It also obtained more precise segmentation results even though its number of parameters is much higher than those of other classic models. The experimental results illustrated that, when compared with various networks, our network was still superior in obtaining abundant detailed information of the spine images. Therefore, the model is generally slightly better than the traditional convolutional neural network.

## Conclusions

This study proposed a series of improved models for the segmentation of MRI spinal images. The network was adjusted by means of convolutional modules, coding units, and feature fusion to gain a newly segmented network and improve the segmentation accuracy. Compared with the traditional segmentation method based on deep learning, the improved method no longer required a large number of training samples. More importantly, it obtained more accurate segmentation results. The experimental results illustrated that compared to the same type of network, the proposed network was still superior in obtaining abundant detailed information of the target and effectively segmented the spine in the MRI images. However, some limitations must be noted. The proposed network must still be improved considering the conflict between accuracy and Dice coefficient for segmentation. Accordingly, we need to design a lightweight and accurate spine segmentation network in the future.

## Supporting information

S1 File (ZIP)
