# Supplementary figures and images for: Research on multi-path dense networks for MRI spinal segmentation (part 2 of 2)
Source: PLoS One. 2021 Mar 12;16(3):e0248303. doi: 10.1371/journal.pone.0248303 (PMC7954354; doi:10.1371/journal.pone.0248303)

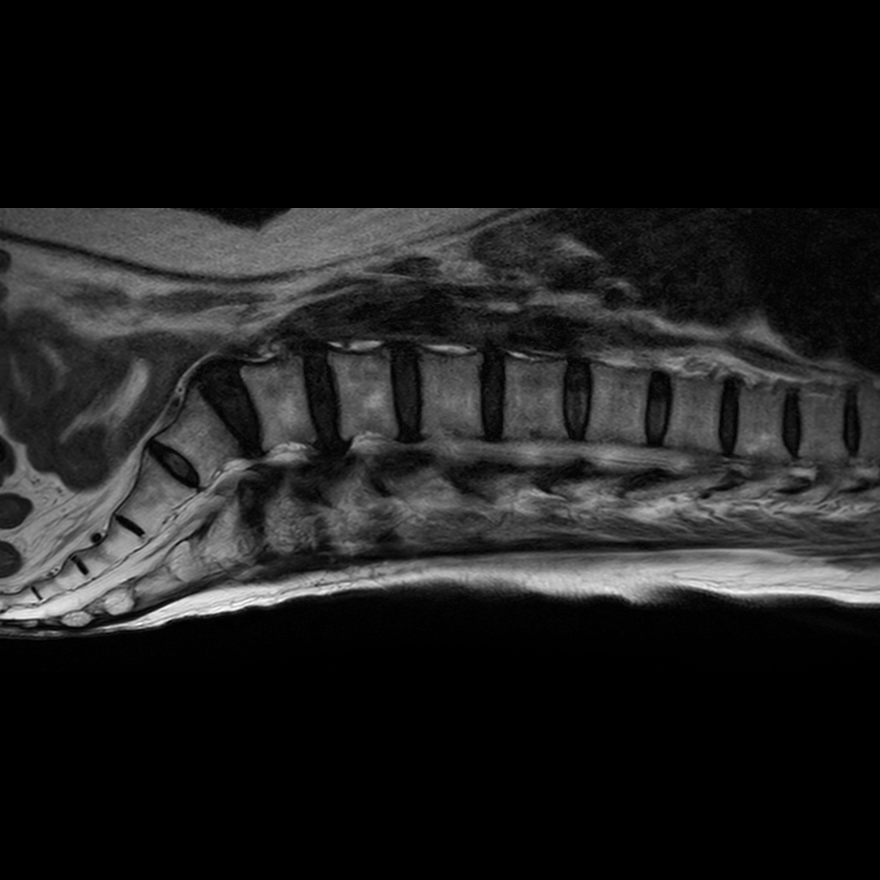

Supplement: S1 File — (ZIP) [file pone.0248303.s001.zip › Code and data/dataset/train/153.png]

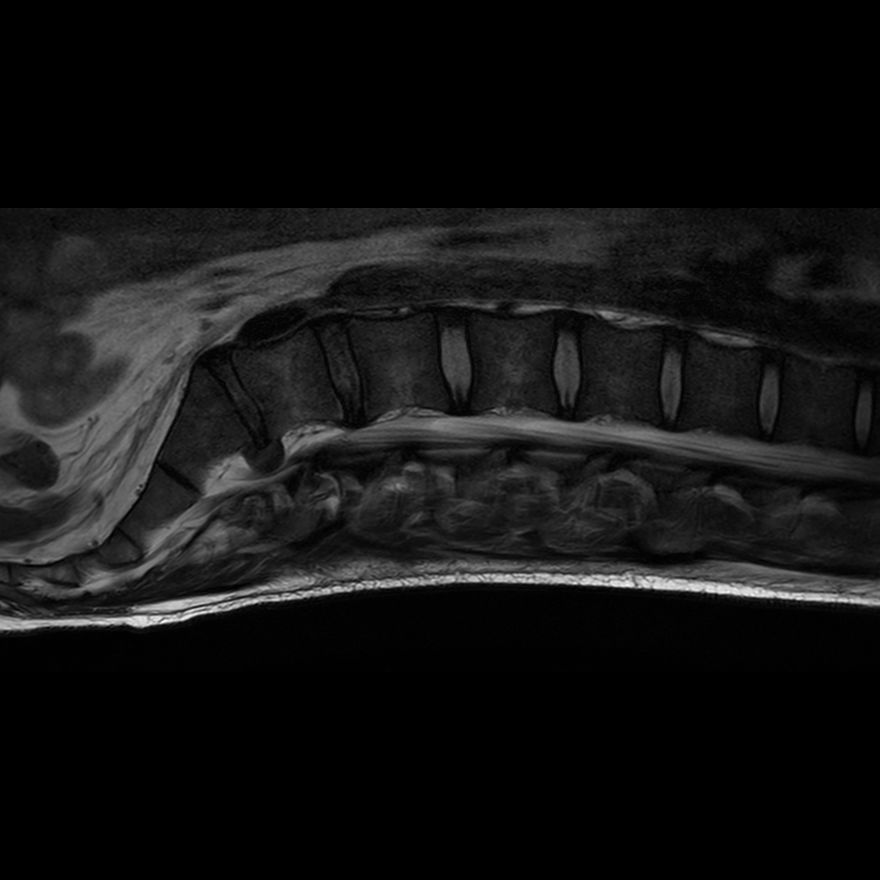

Supplement: S1 File — (ZIP) [file pone.0248303.s001.zip › Code and data/dataset/train/154.png]

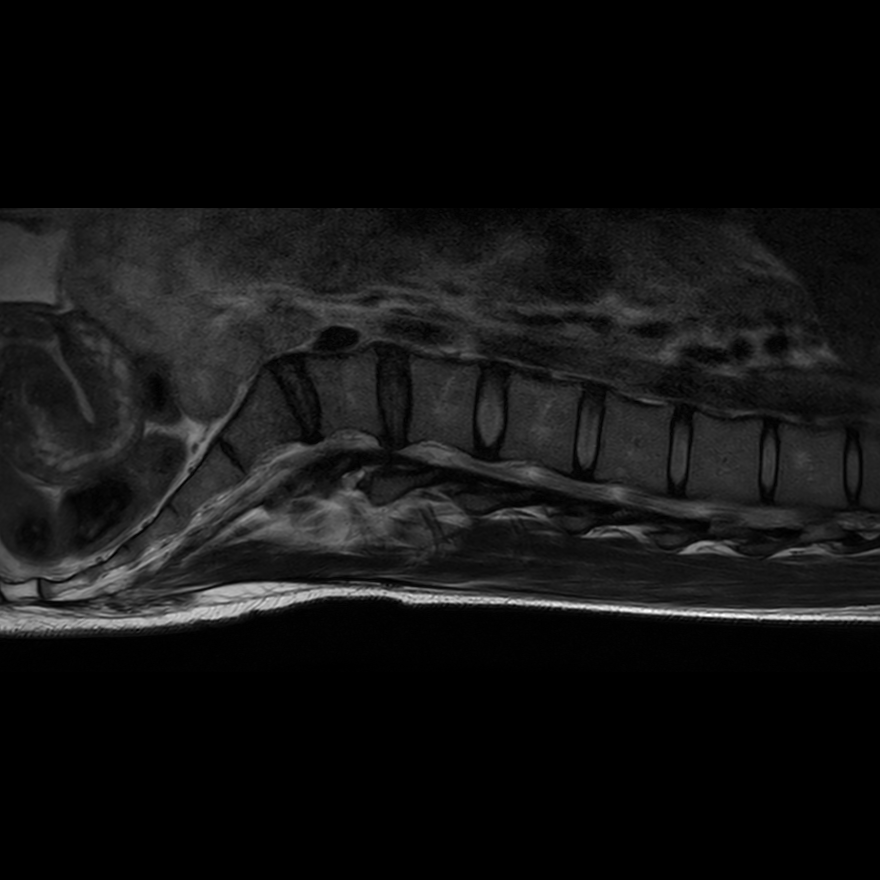

Supplement: S1 File — (ZIP) [file pone.0248303.s001.zip › Code and data/dataset/train/155.png]

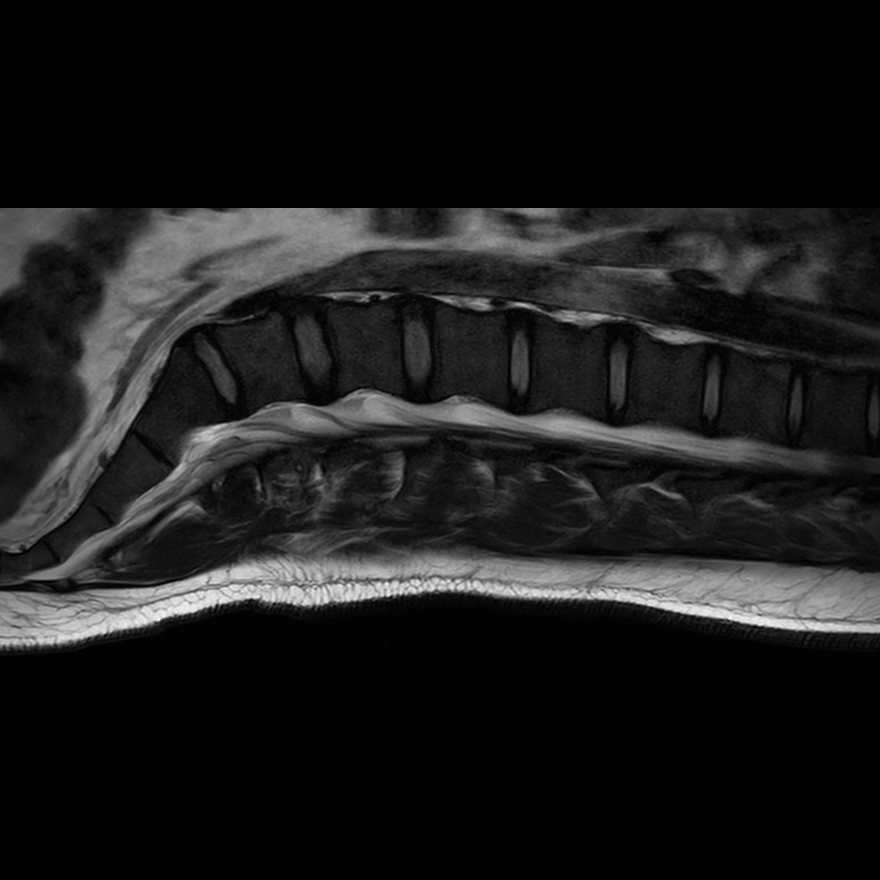

Supplement: S1 File — (ZIP) [file pone.0248303.s001.zip › Code and data/dataset/train/156.png]

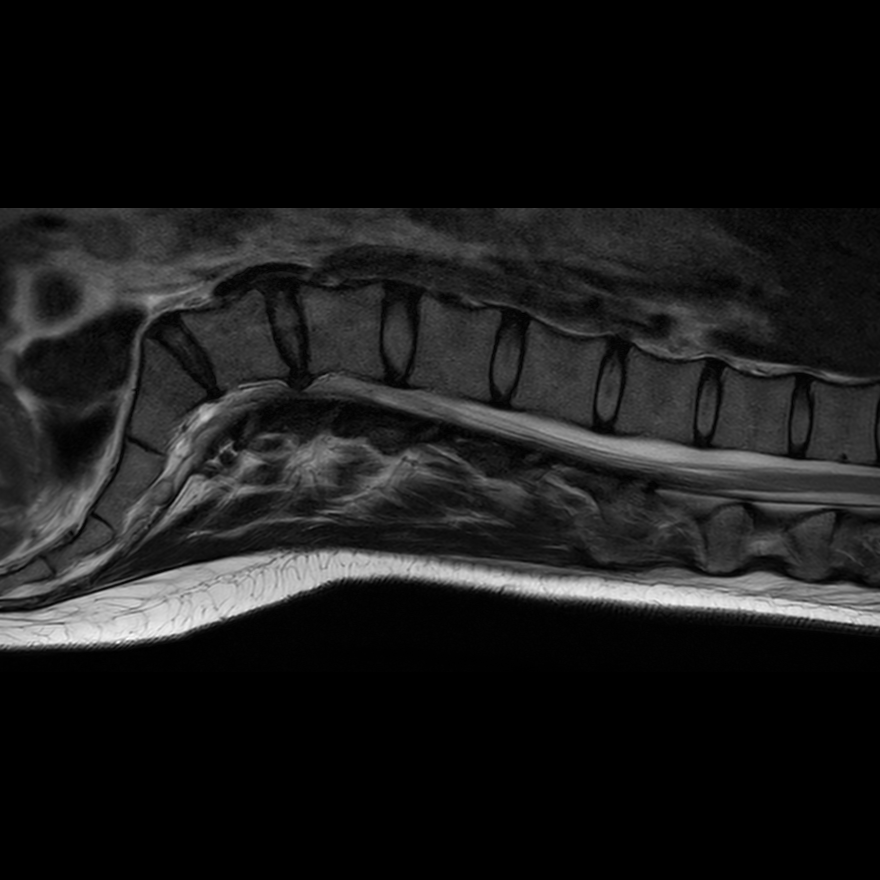

Supplement: S1 File — (ZIP) [file pone.0248303.s001.zip › Code and data/dataset/train/157.png]

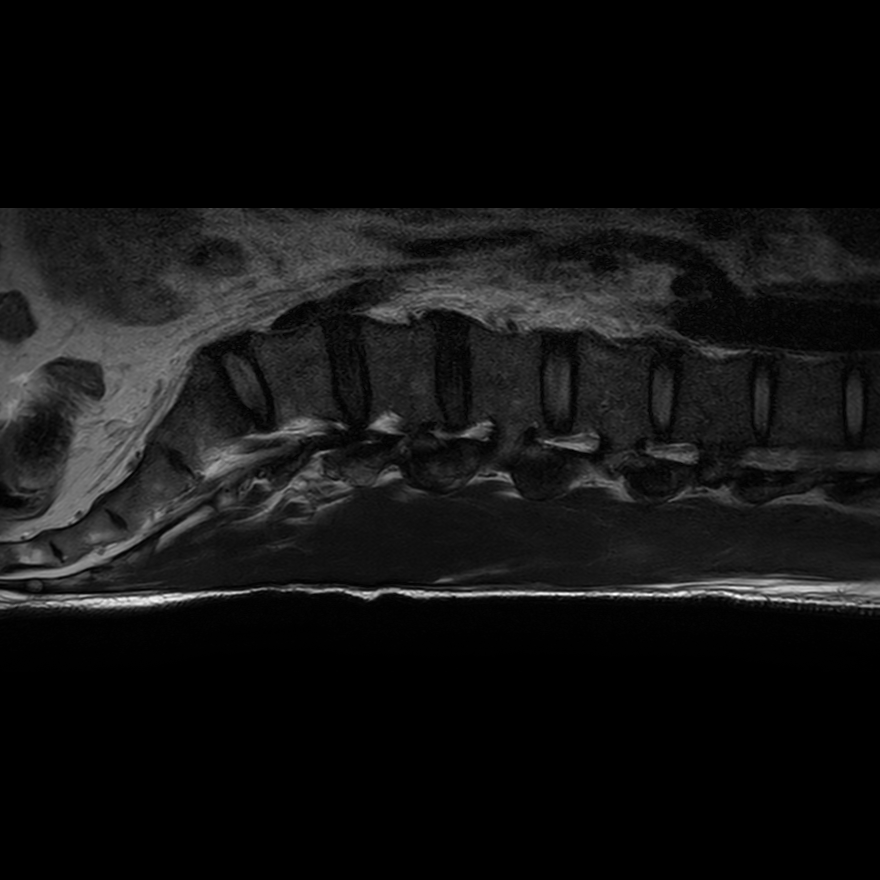

Supplement: S1 File — (ZIP) [file pone.0248303.s001.zip › Code and data/dataset/train/158.png]

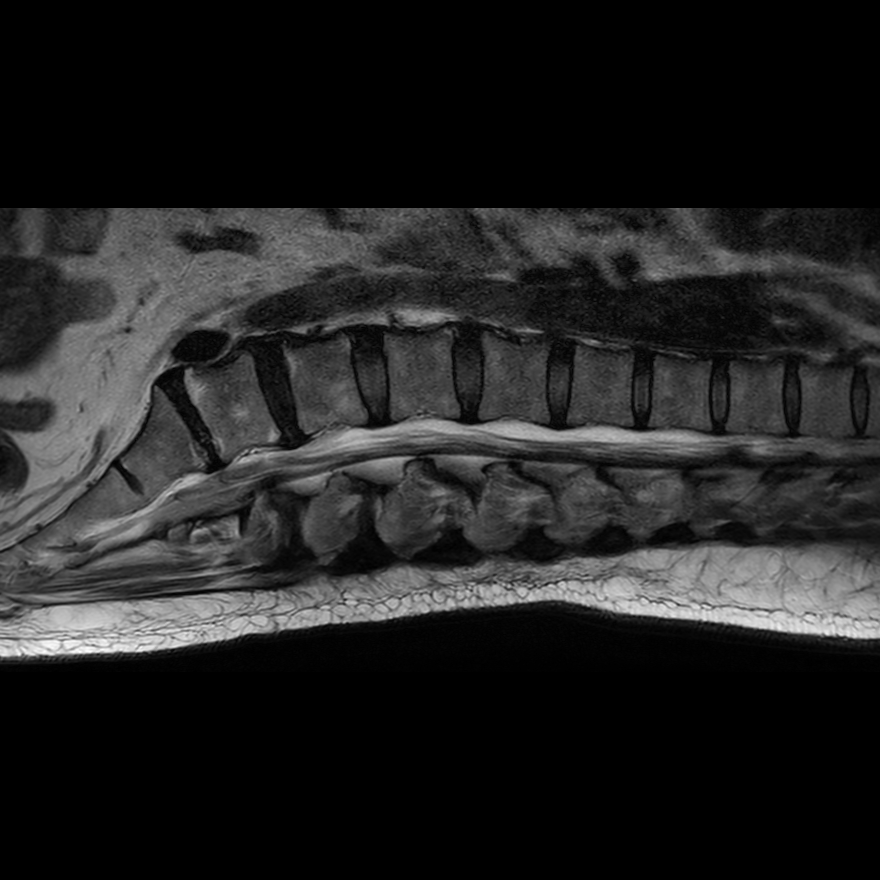

Supplement: S1 File — (ZIP) [file pone.0248303.s001.zip › Code and data/dataset/train/159.png]

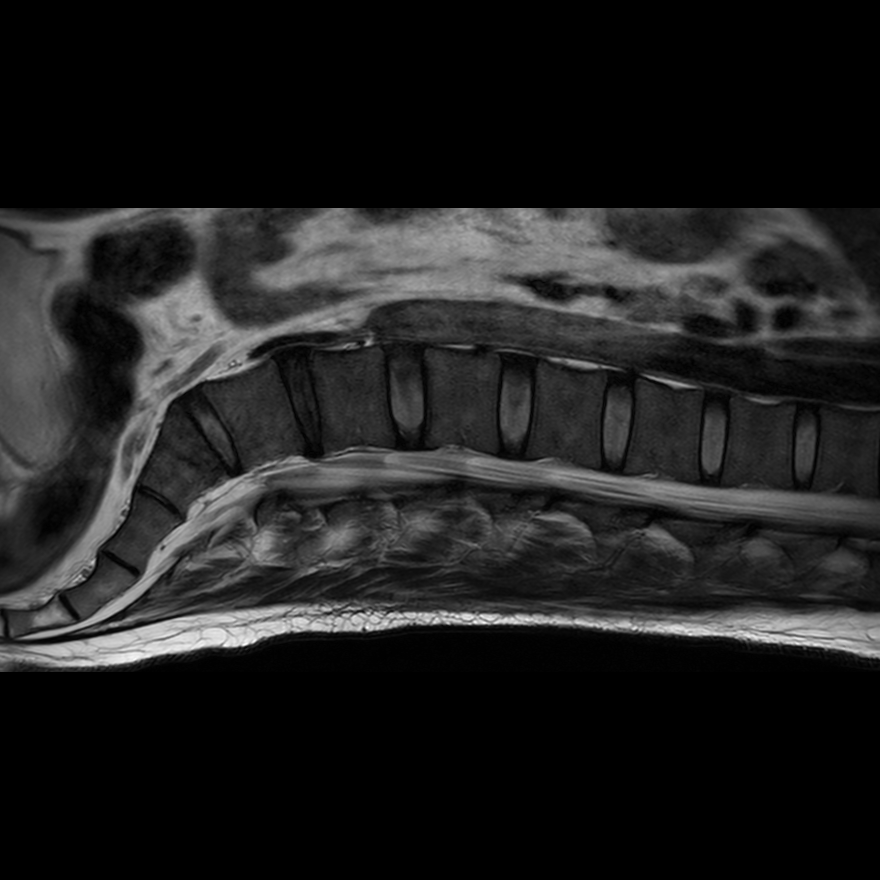

Supplement: S1 File — (ZIP) [file pone.0248303.s001.zip › Code and data/dataset/train/16.png]

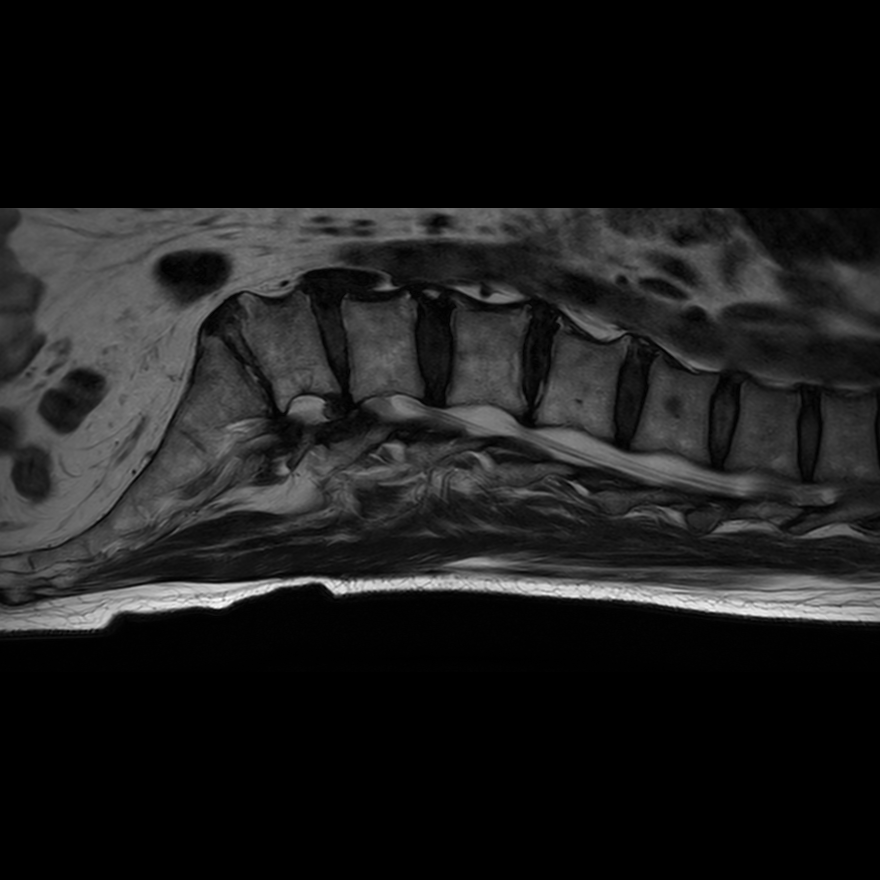

Supplement: S1 File — (ZIP) [file pone.0248303.s001.zip › Code and data/dataset/train/17.png]

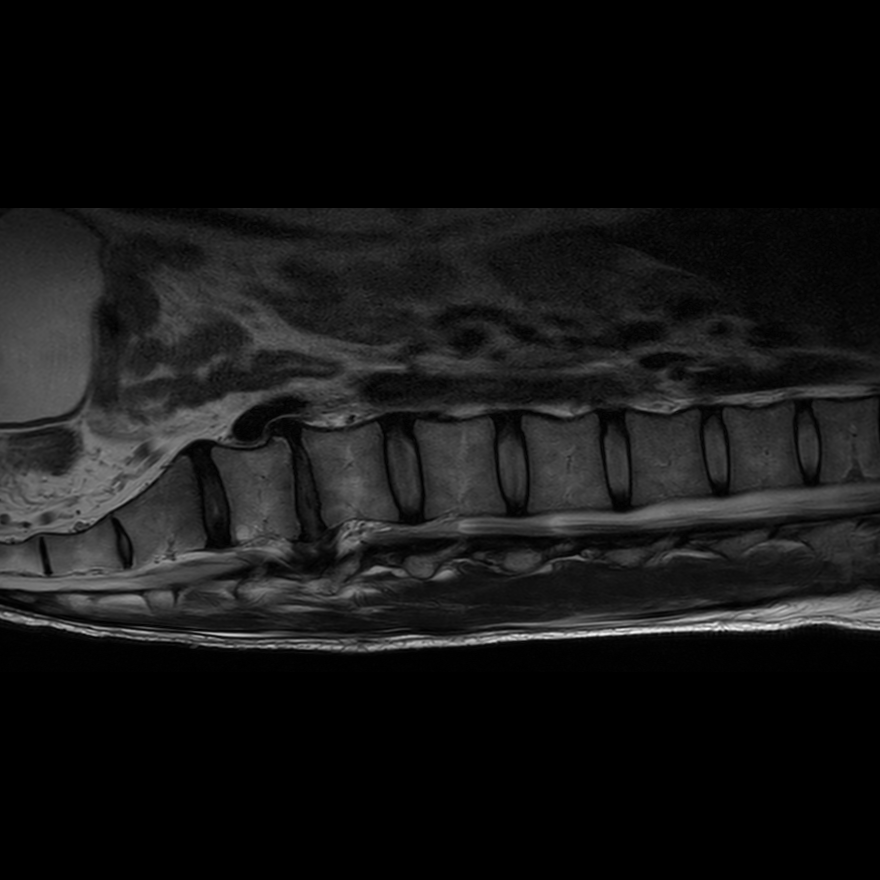

Supplement: S1 File — (ZIP) [file pone.0248303.s001.zip › Code and data/dataset/train/18.png]

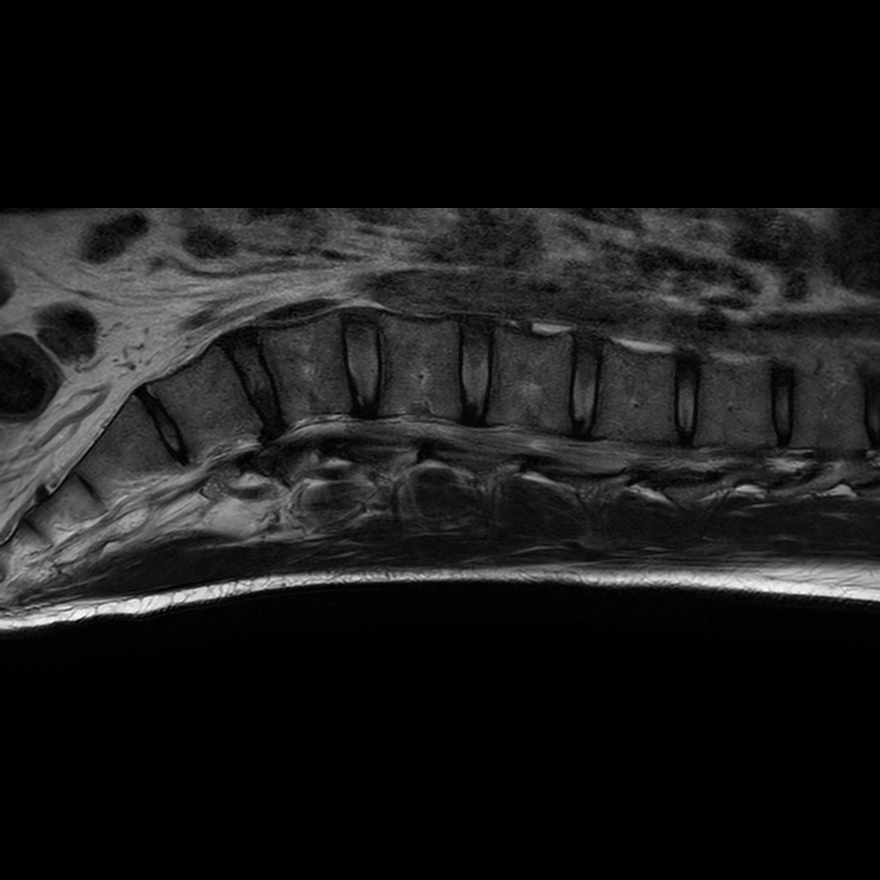

Supplement: S1 File — (ZIP) [file pone.0248303.s001.zip › Code and data/dataset/train/19.png]

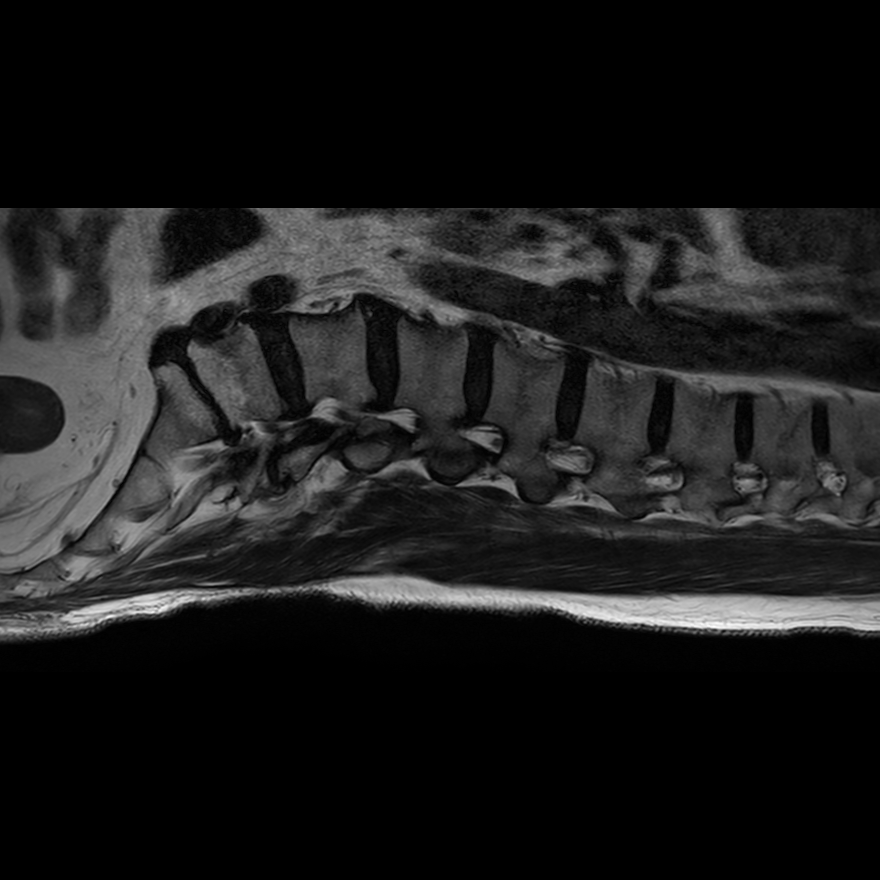

Supplement: S1 File — (ZIP) [file pone.0248303.s001.zip › Code and data/dataset/train/20.png]

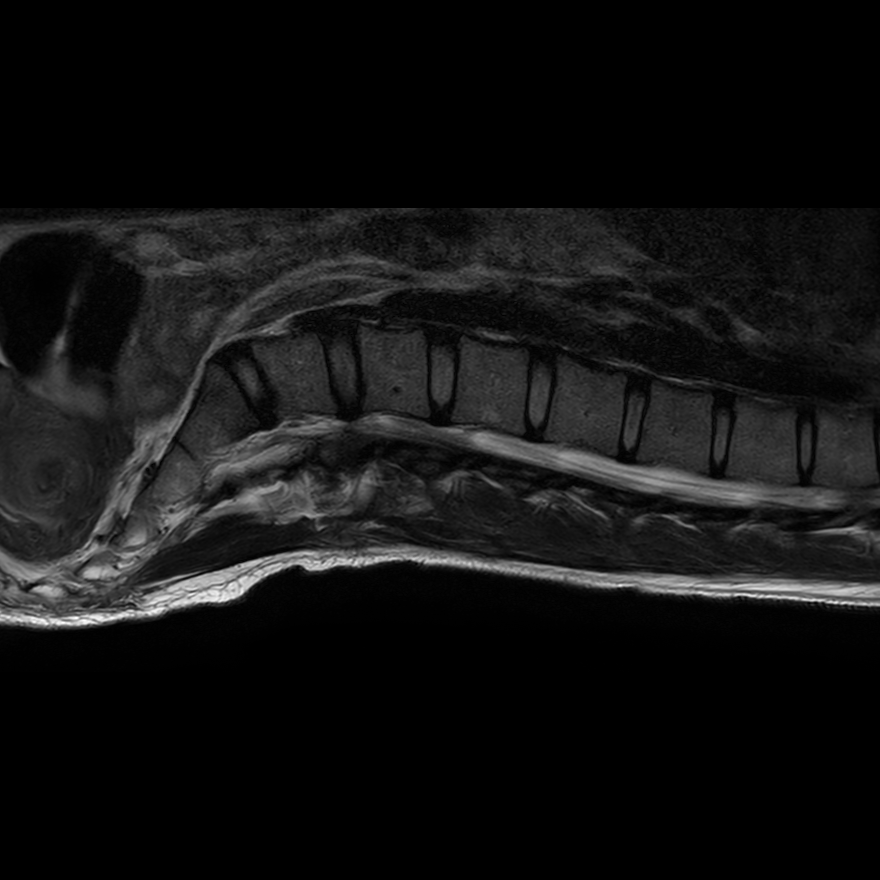

Supplement: S1 File — (ZIP) [file pone.0248303.s001.zip › Code and data/dataset/train/21.png]

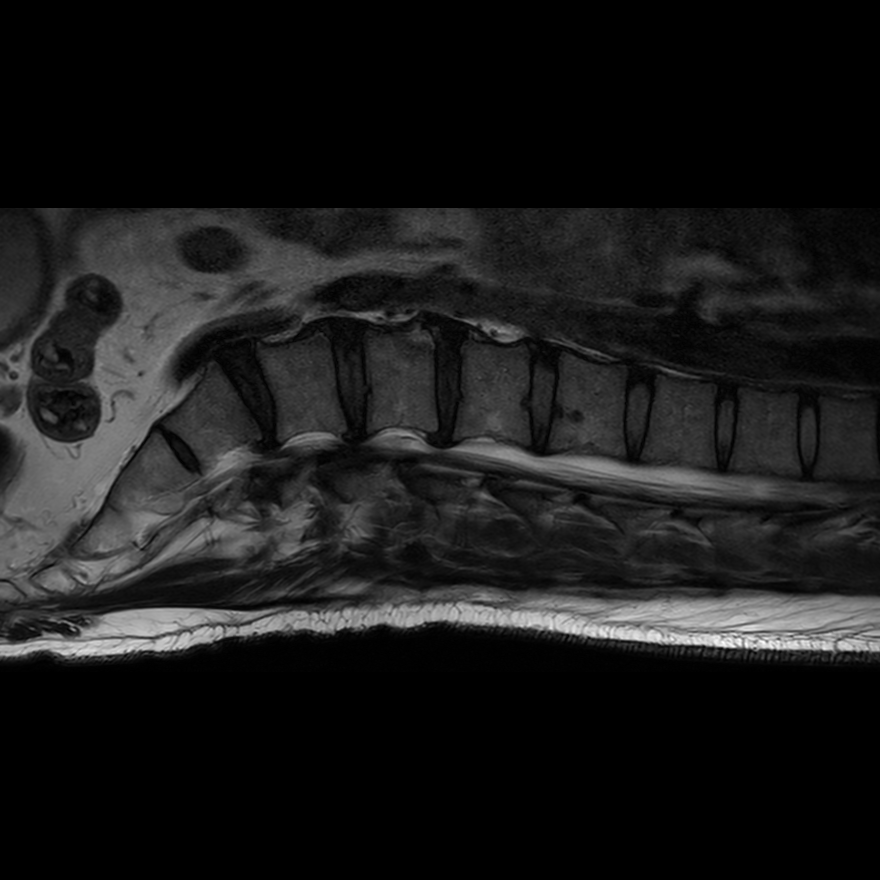

Supplement: S1 File — (ZIP) [file pone.0248303.s001.zip › Code and data/dataset/train/22.png]

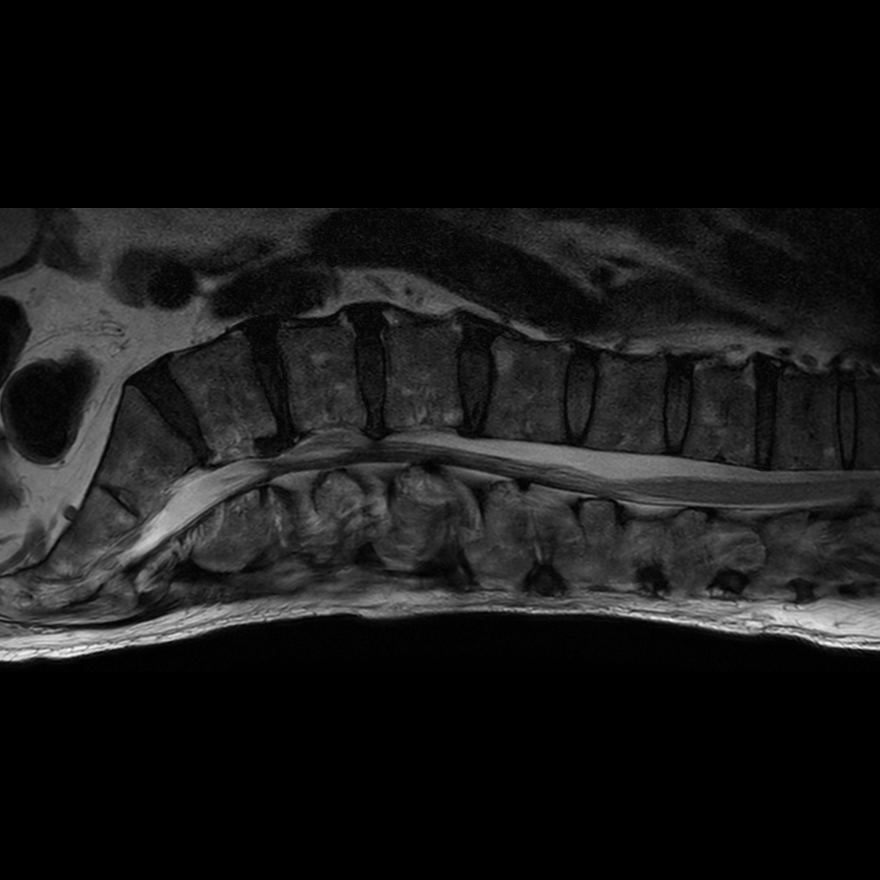

Supplement: S1 File — (ZIP) [file pone.0248303.s001.zip › Code and data/dataset/train/23.png]

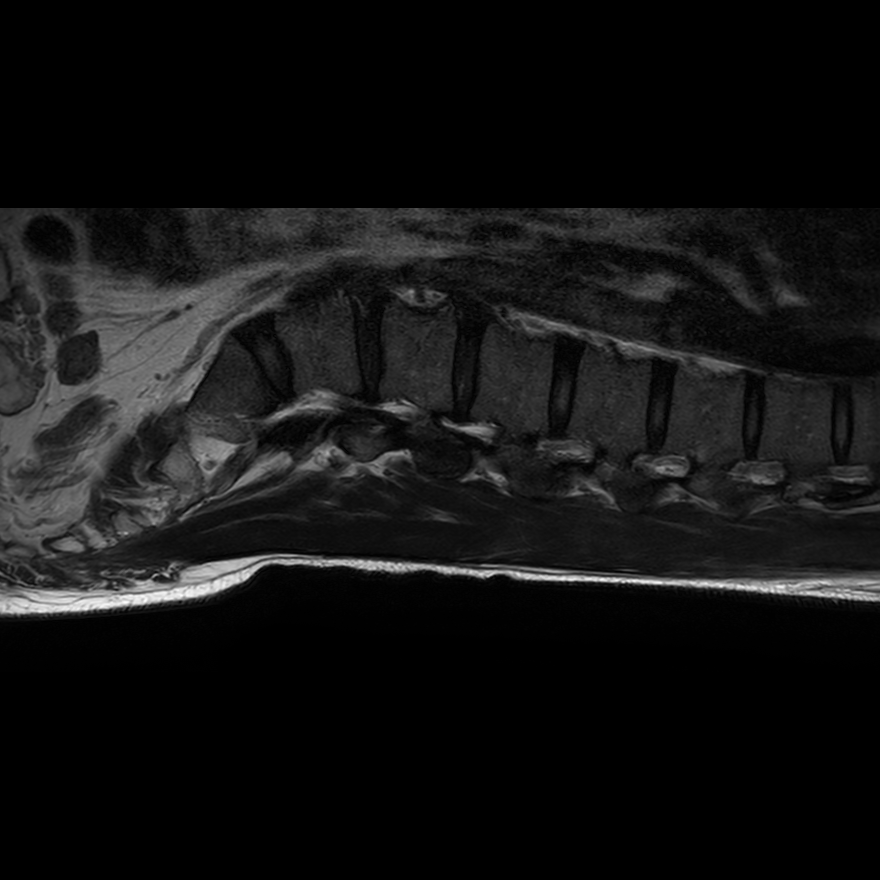

Supplement: S1 File — (ZIP) [file pone.0248303.s001.zip › Code and data/dataset/train/24.png]

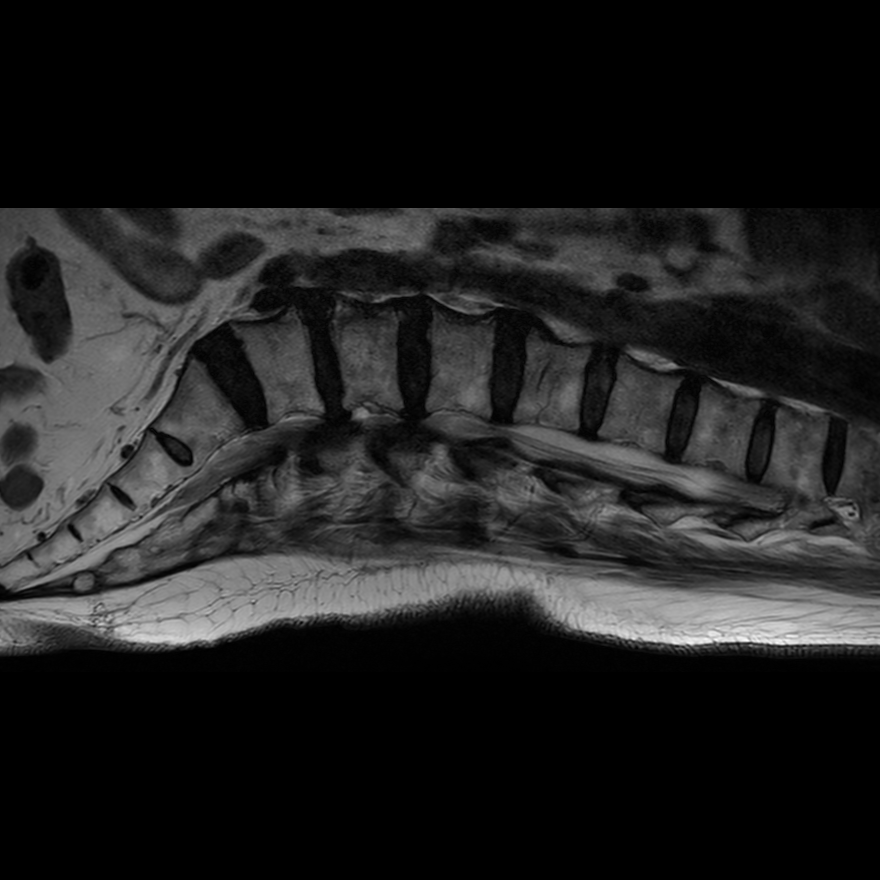

Supplement: S1 File — (ZIP) [file pone.0248303.s001.zip › Code and data/dataset/train/25.png]

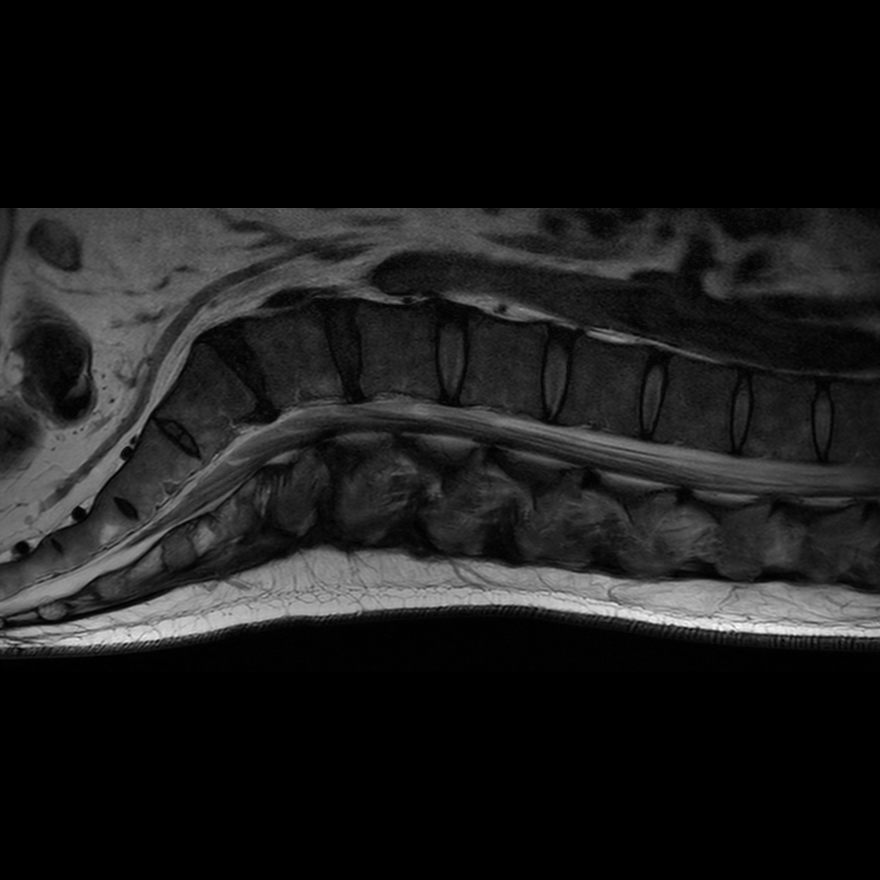

Supplement: S1 File — (ZIP) [file pone.0248303.s001.zip › Code and data/dataset/train/26.png]

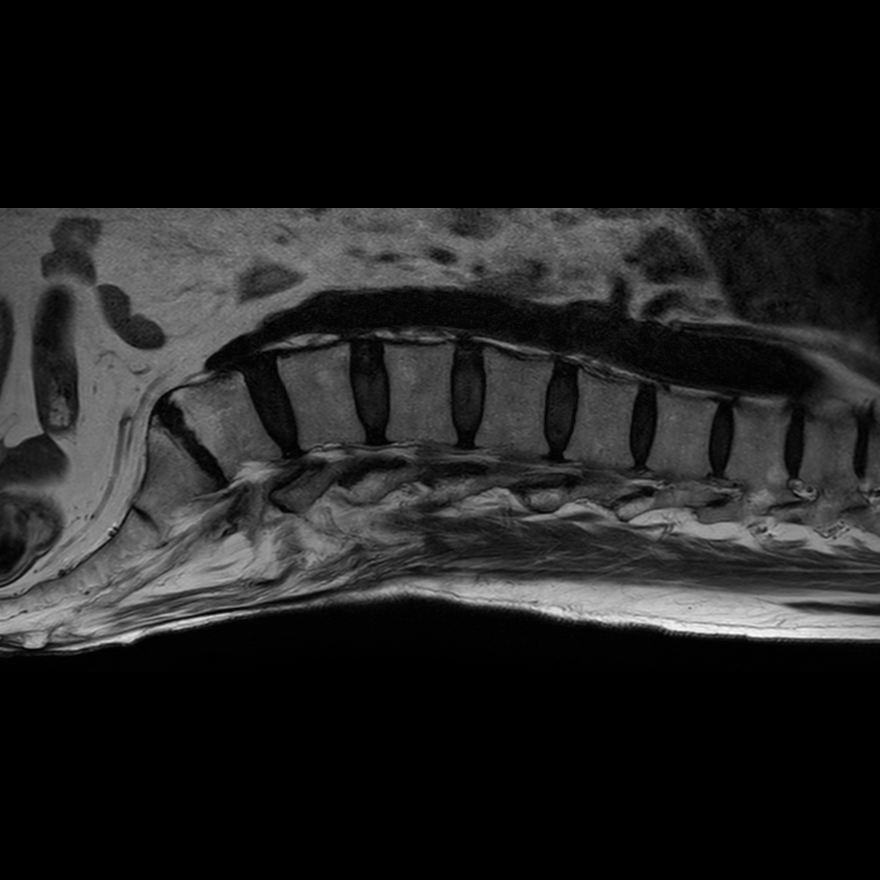

Supplement: S1 File — (ZIP) [file pone.0248303.s001.zip › Code and data/dataset/train/27.png]

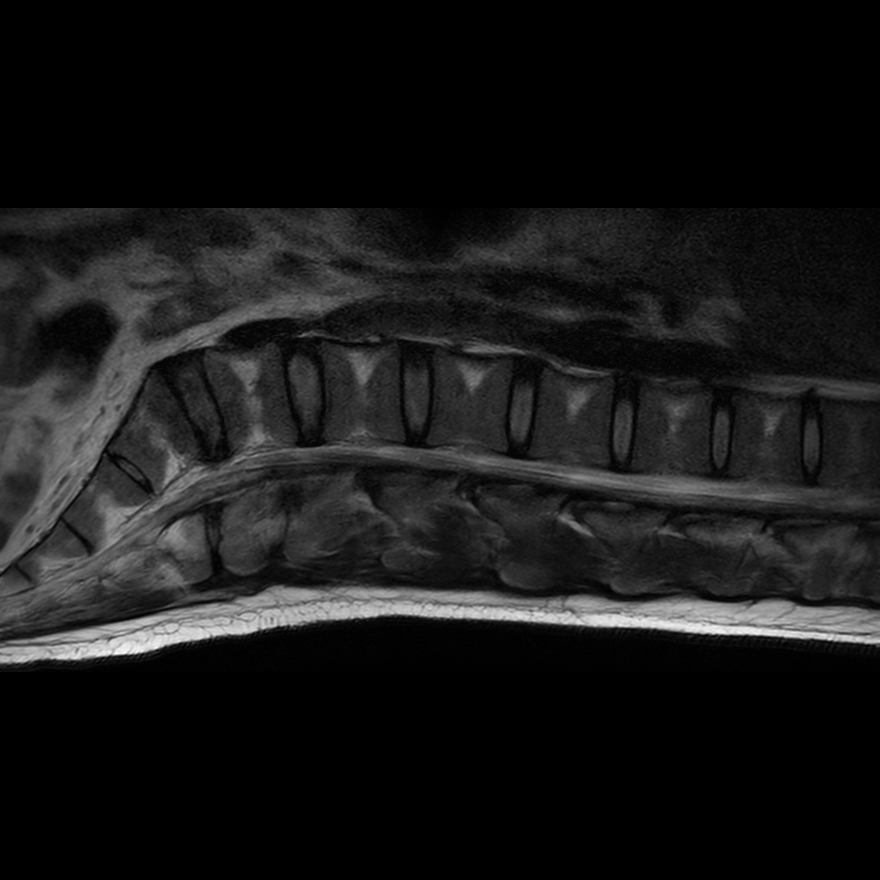

Supplement: S1 File — (ZIP) [file pone.0248303.s001.zip › Code and data/dataset/train/28.png]

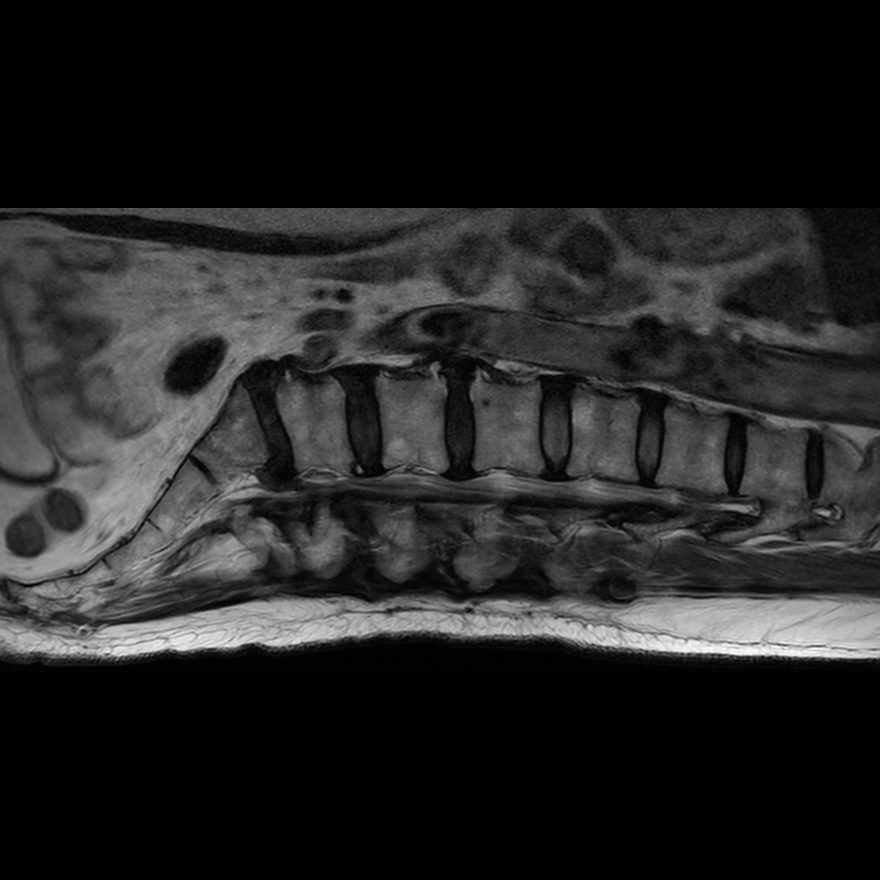

Supplement: S1 File — (ZIP) [file pone.0248303.s001.zip › Code and data/dataset/train/29.png]

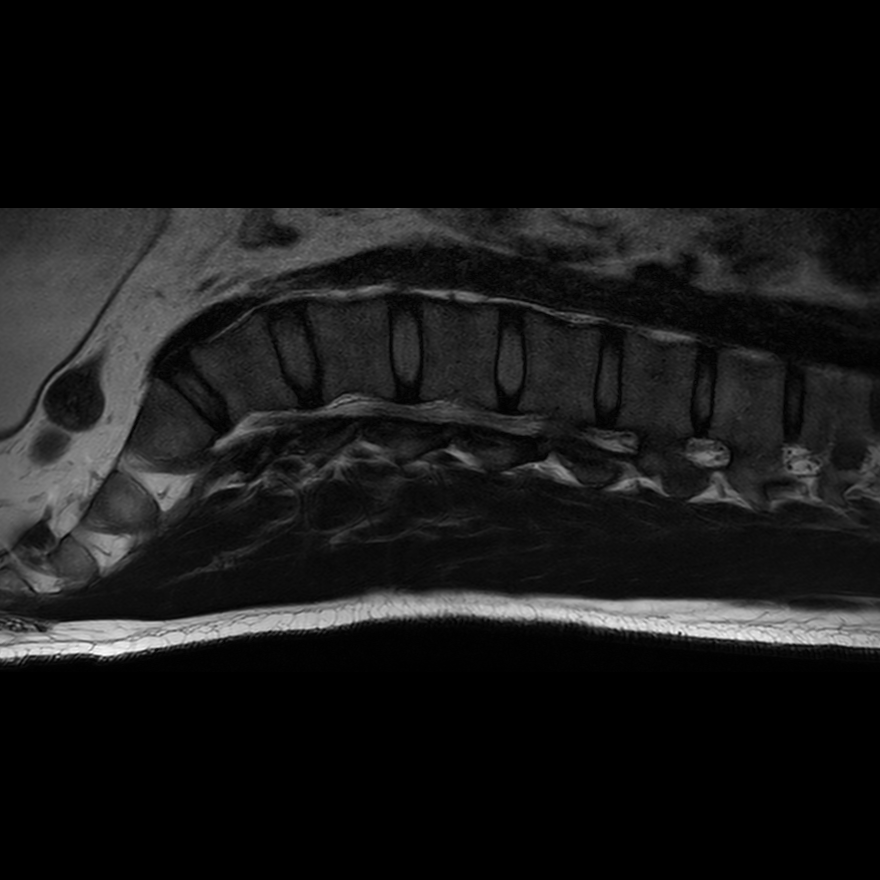

Supplement: S1 File — (ZIP) [file pone.0248303.s001.zip › Code and data/dataset/train/3.png]

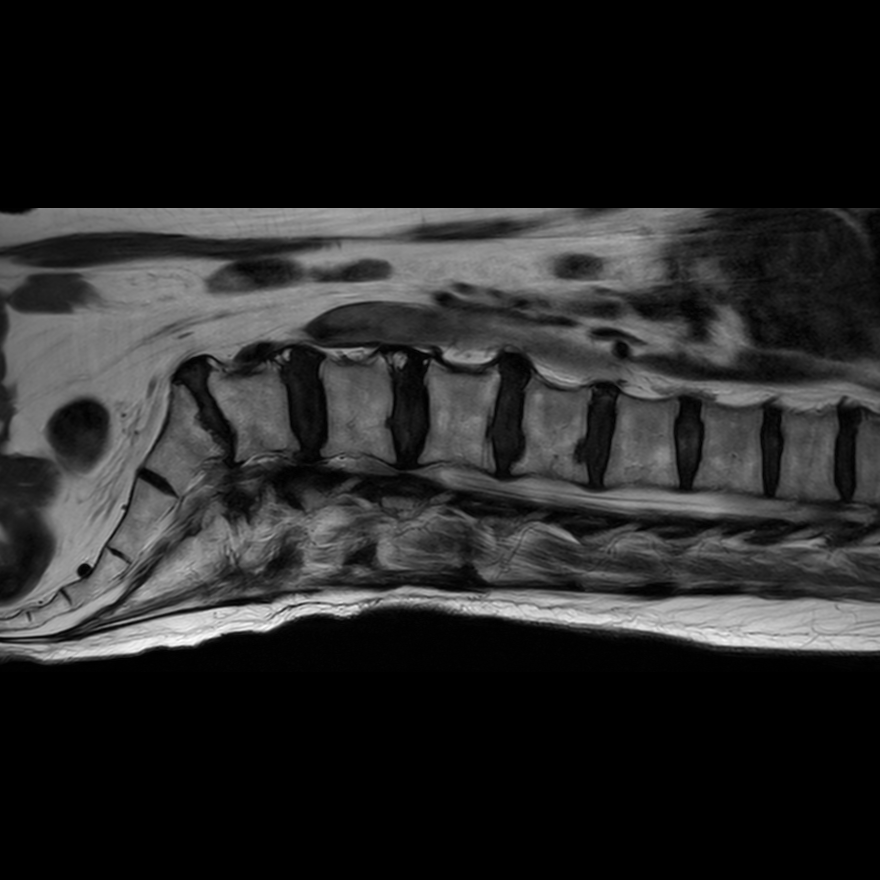

Supplement: S1 File — (ZIP) [file pone.0248303.s001.zip › Code and data/dataset/train/30.png]

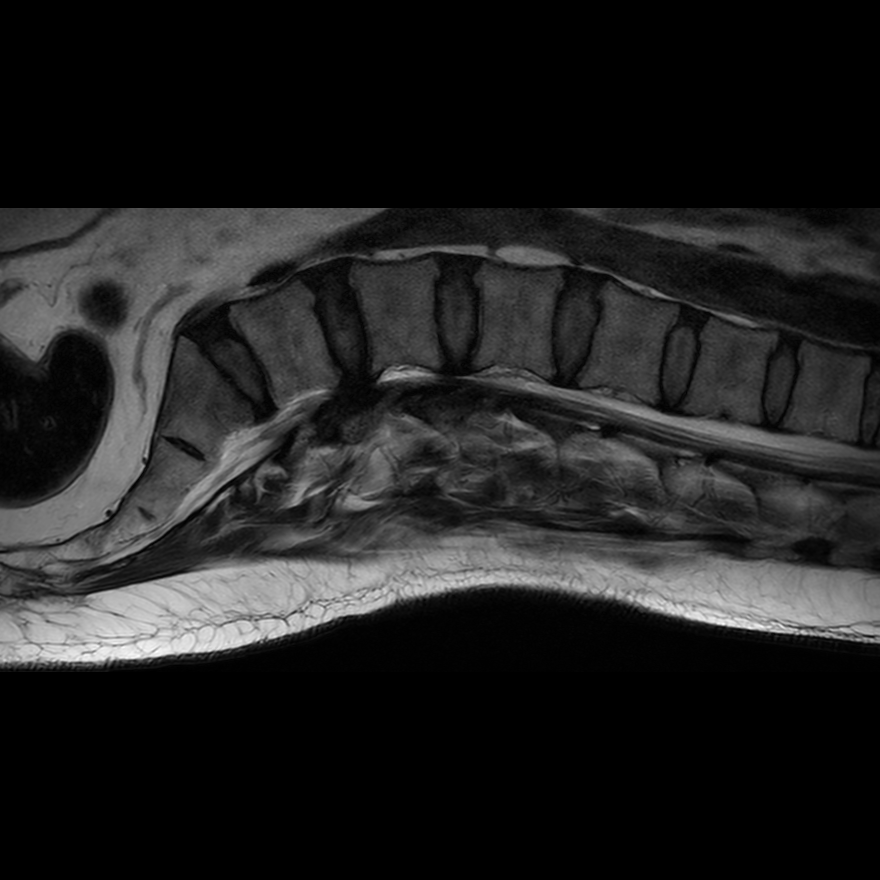

Supplement: S1 File — (ZIP) [file pone.0248303.s001.zip › Code and data/dataset/train/31.png]

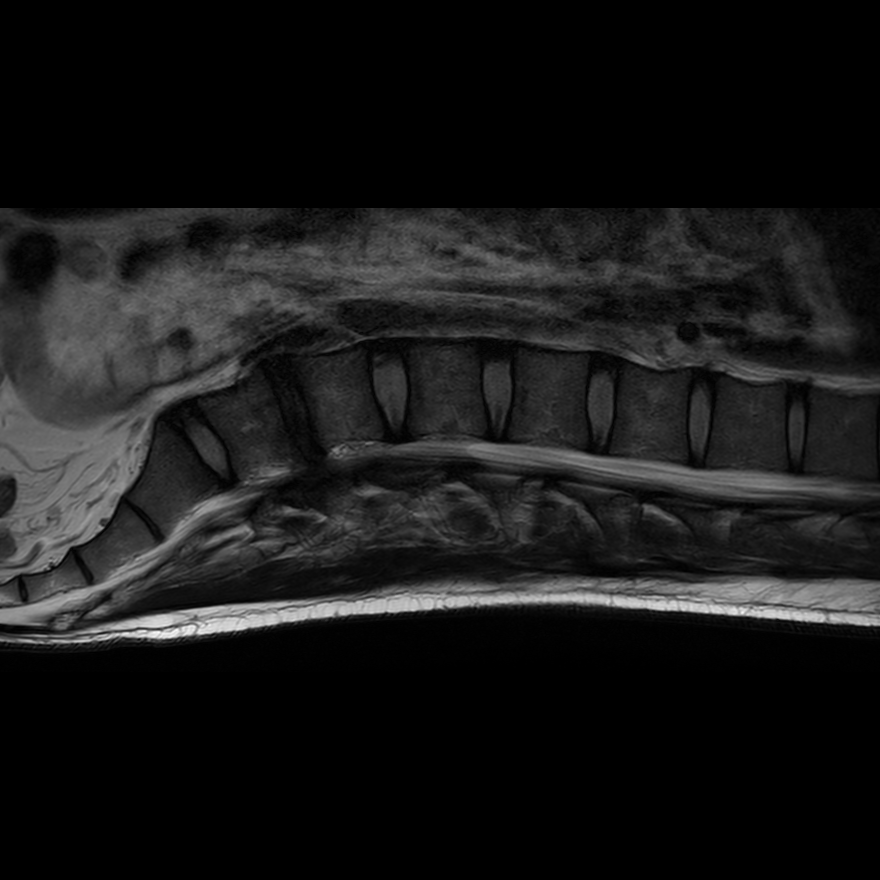

Supplement: S1 File — (ZIP) [file pone.0248303.s001.zip › Code and data/dataset/train/32.png]

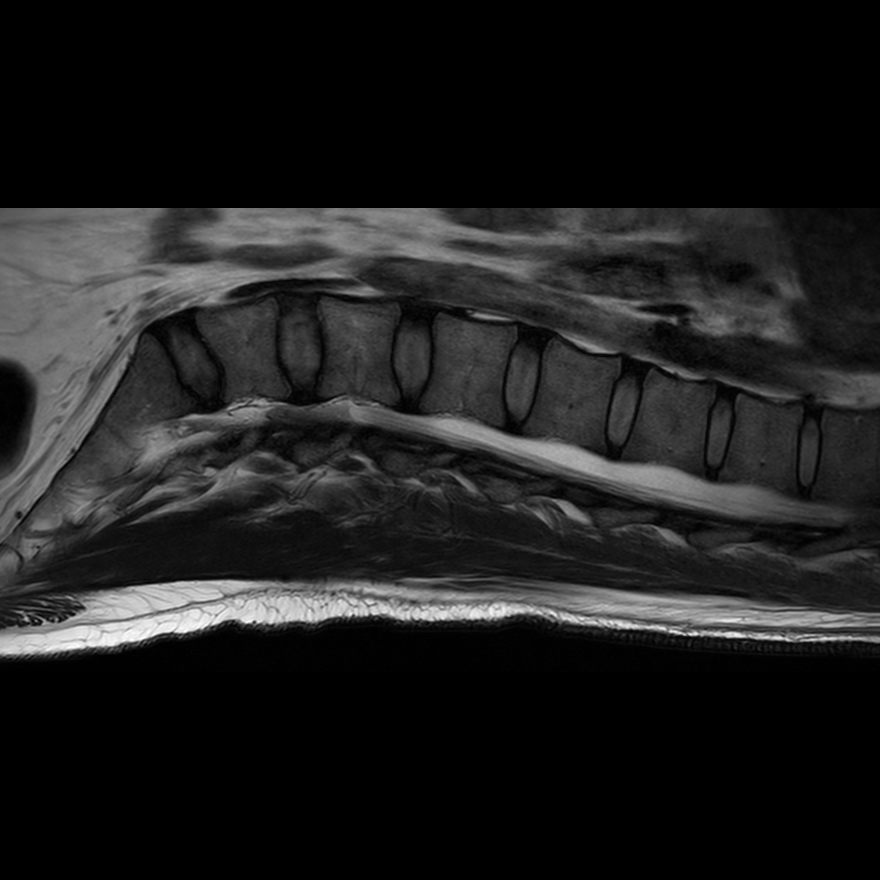

Supplement: S1 File — (ZIP) [file pone.0248303.s001.zip › Code and data/dataset/train/33.png]

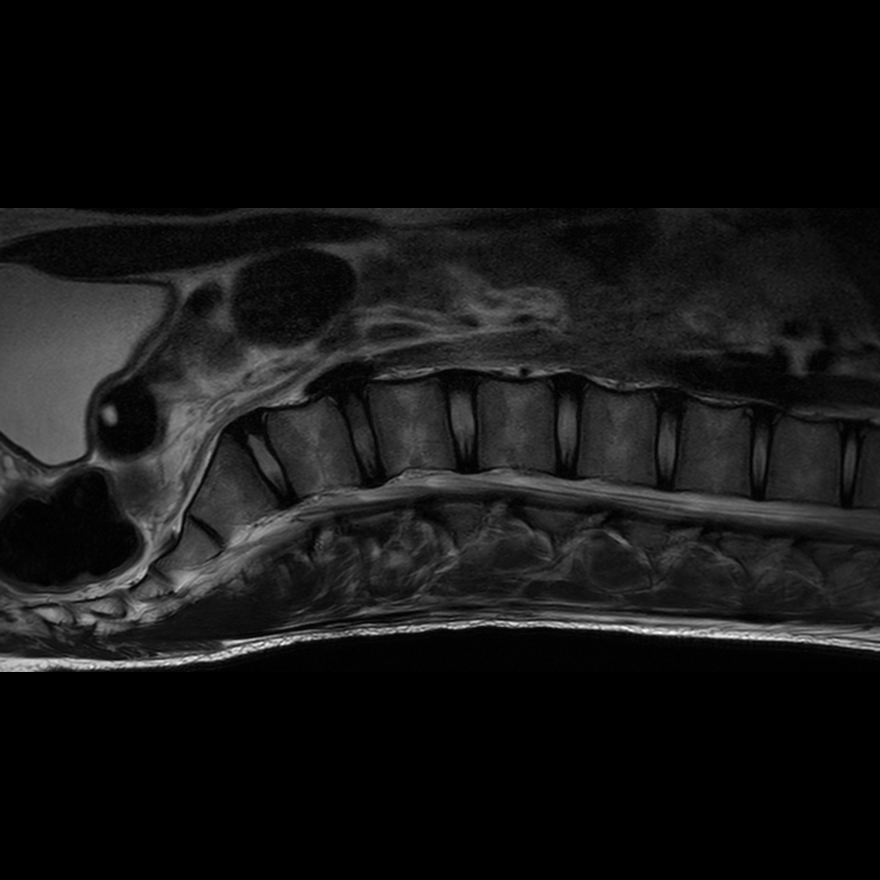

Supplement: S1 File — (ZIP) [file pone.0248303.s001.zip › Code and data/dataset/train/34.png]

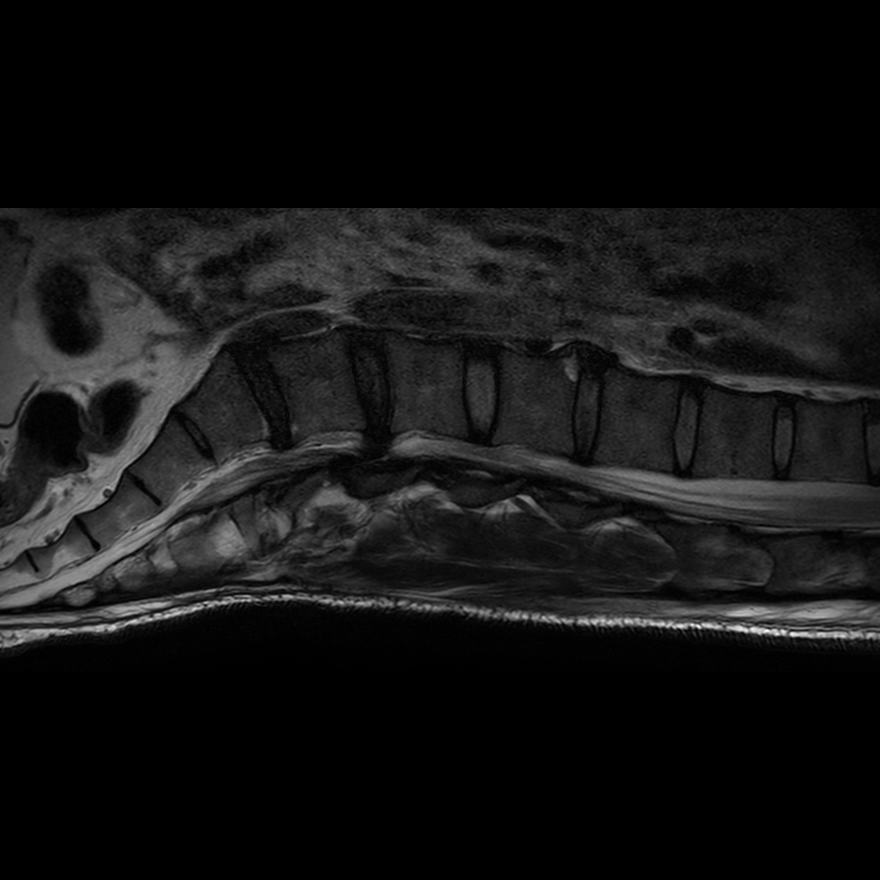

Supplement: S1 File — (ZIP) [file pone.0248303.s001.zip › Code and data/dataset/train/35.png]

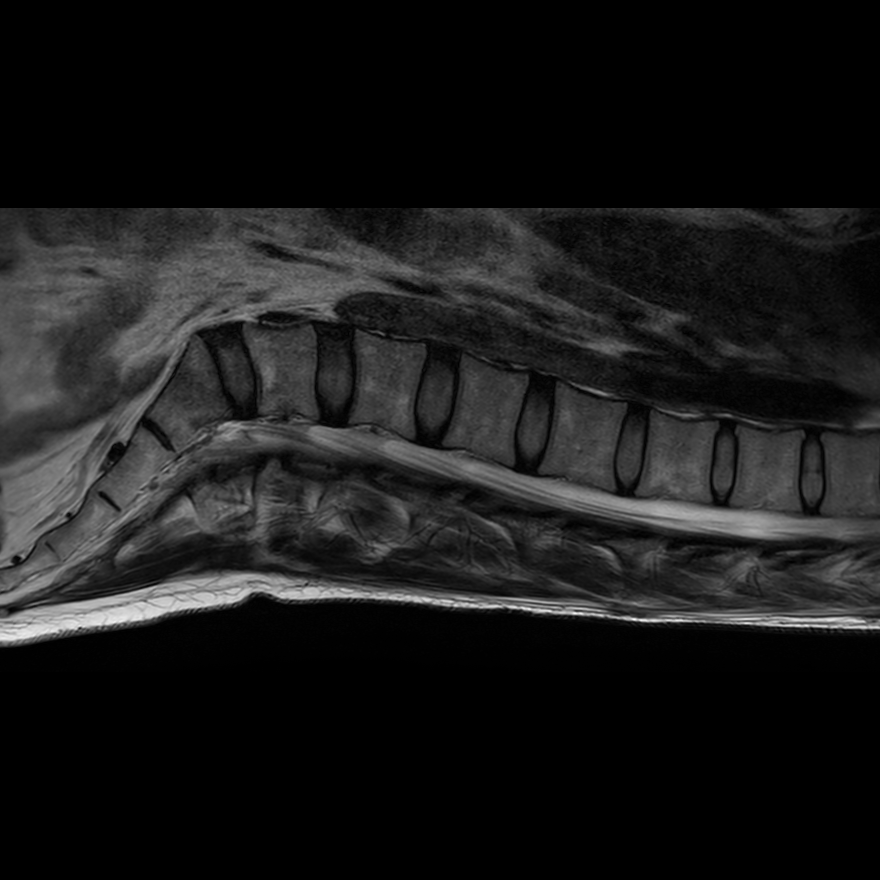

Supplement: S1 File — (ZIP) [file pone.0248303.s001.zip › Code and data/dataset/train/36.png]

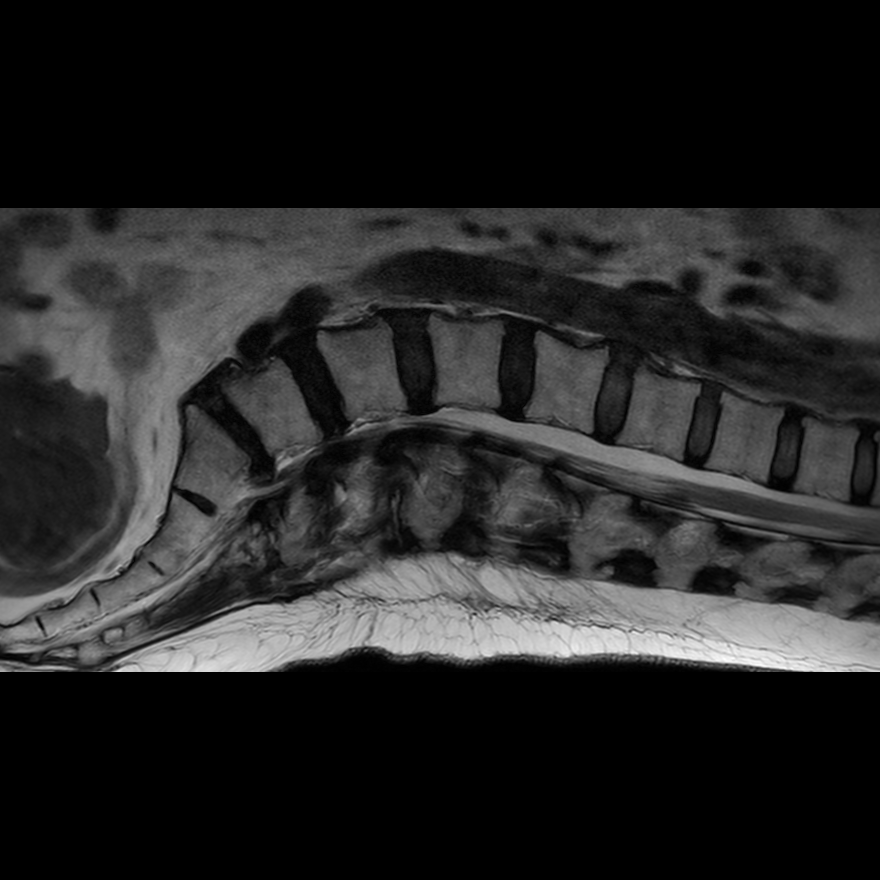

Supplement: S1 File — (ZIP) [file pone.0248303.s001.zip › Code and data/dataset/train/37.png]

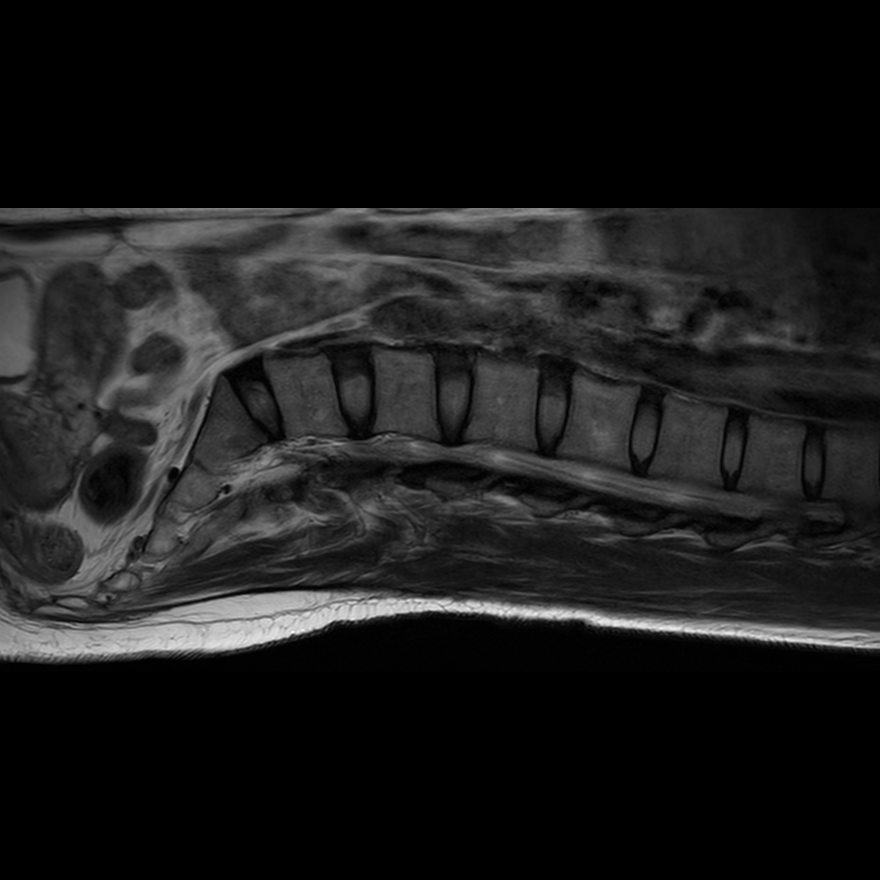

Supplement: S1 File — (ZIP) [file pone.0248303.s001.zip › Code and data/dataset/train/38.png]

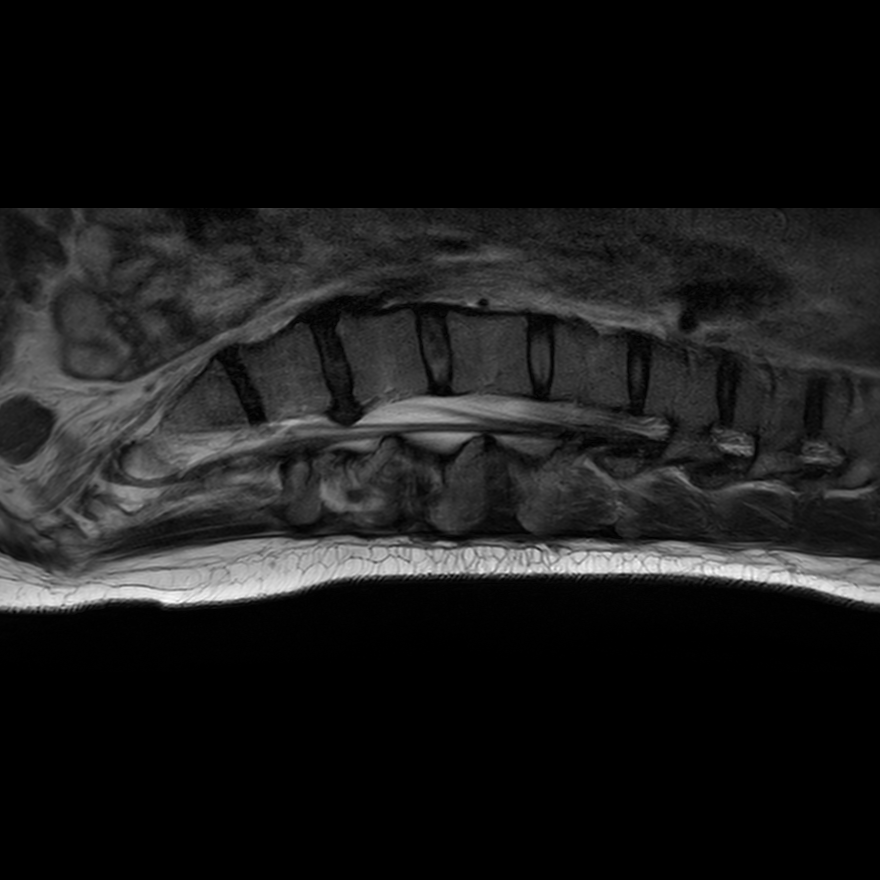

Supplement: S1 File — (ZIP) [file pone.0248303.s001.zip › Code and data/dataset/train/39.png]

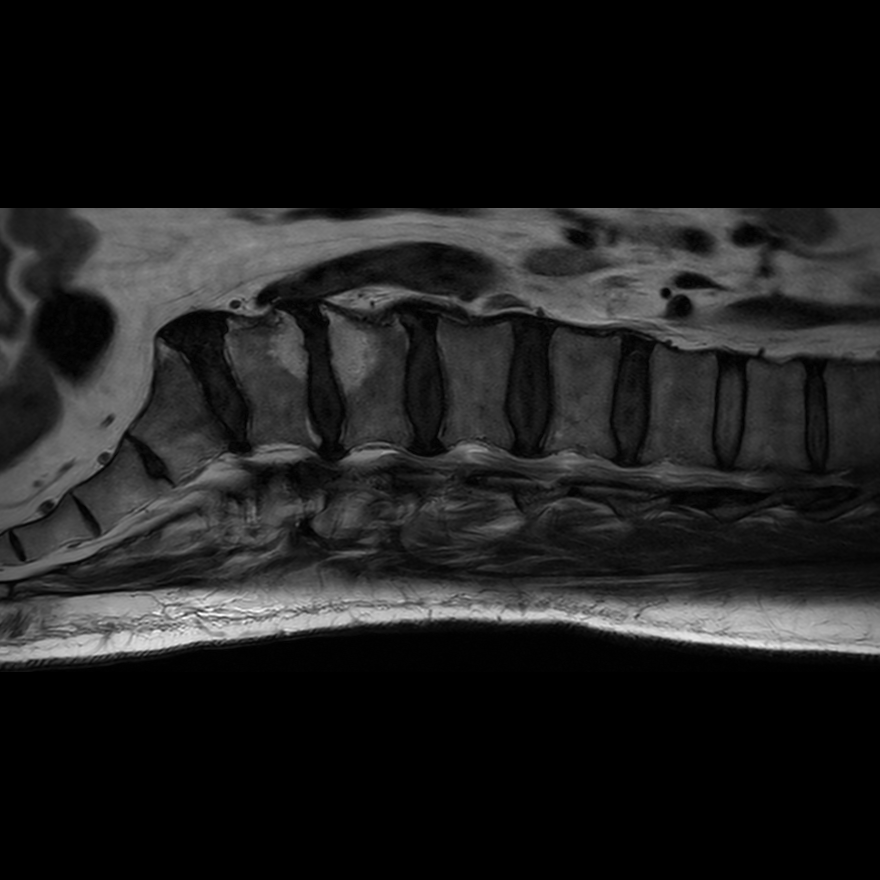

Supplement: S1 File — (ZIP) [file pone.0248303.s001.zip › Code and data/dataset/train/4.png]

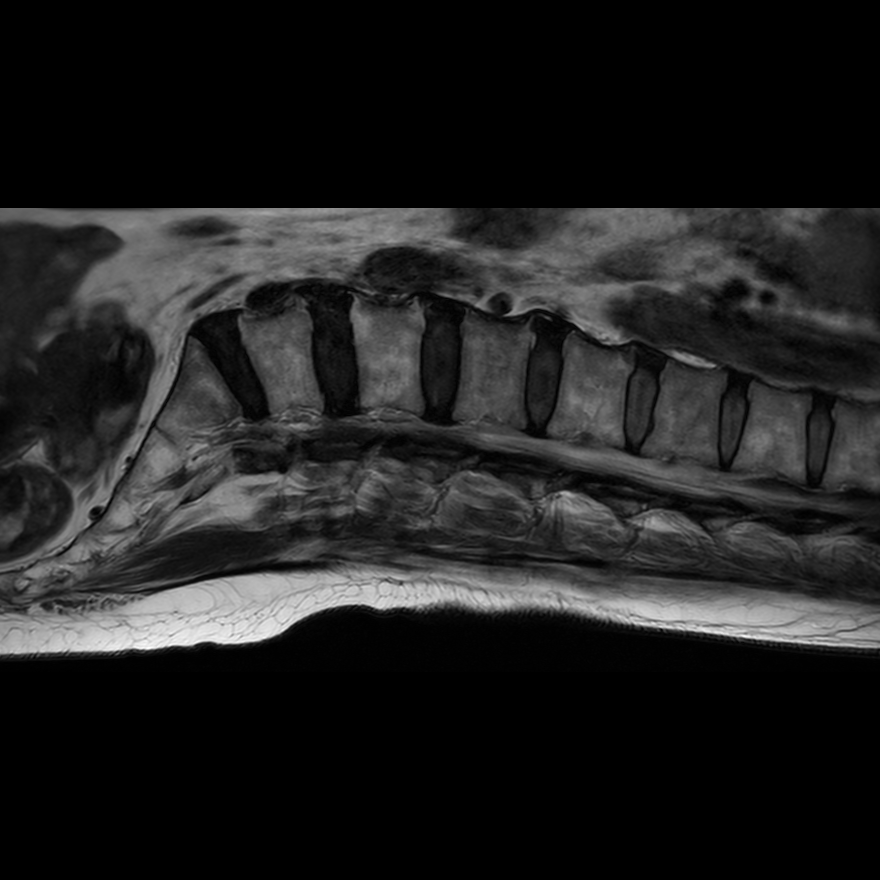

Supplement: S1 File — (ZIP) [file pone.0248303.s001.zip › Code and data/dataset/train/40.png]

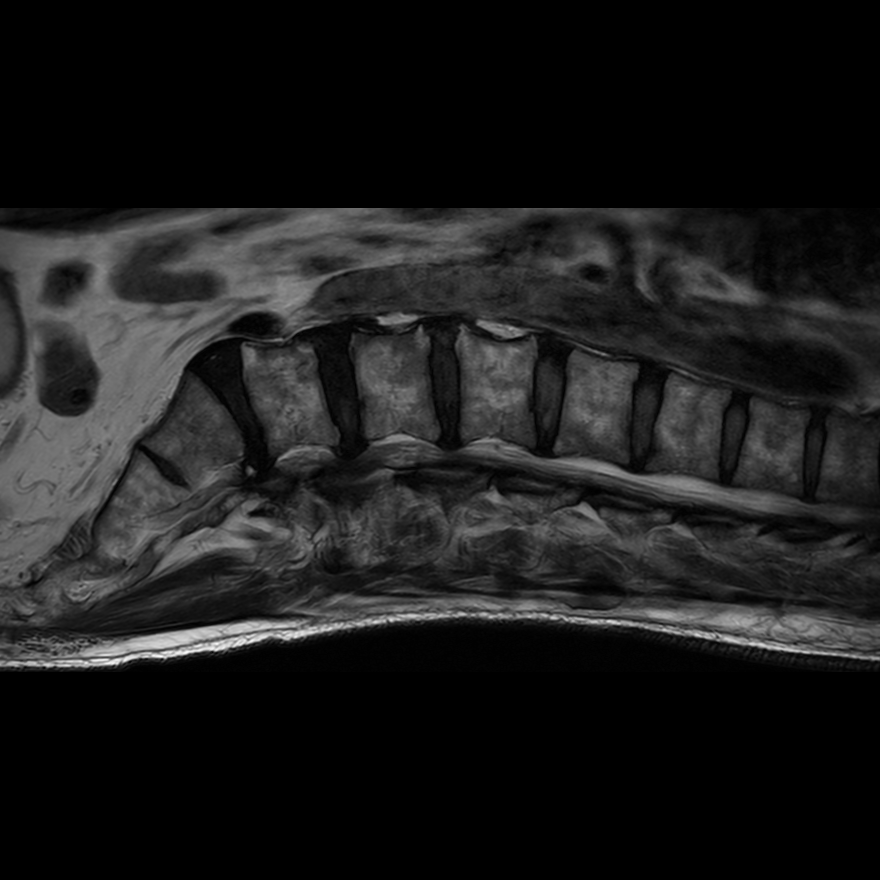

Supplement: S1 File — (ZIP) [file pone.0248303.s001.zip › Code and data/dataset/train/41.png]

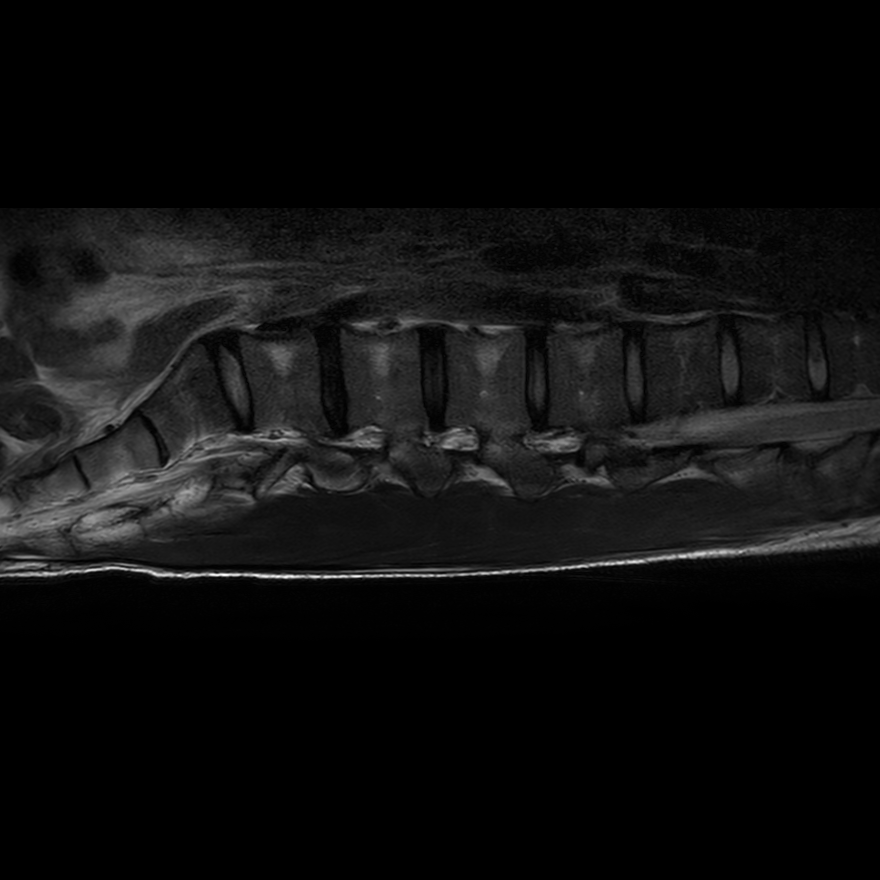

Supplement: S1 File — (ZIP) [file pone.0248303.s001.zip › Code and data/dataset/train/42.png]

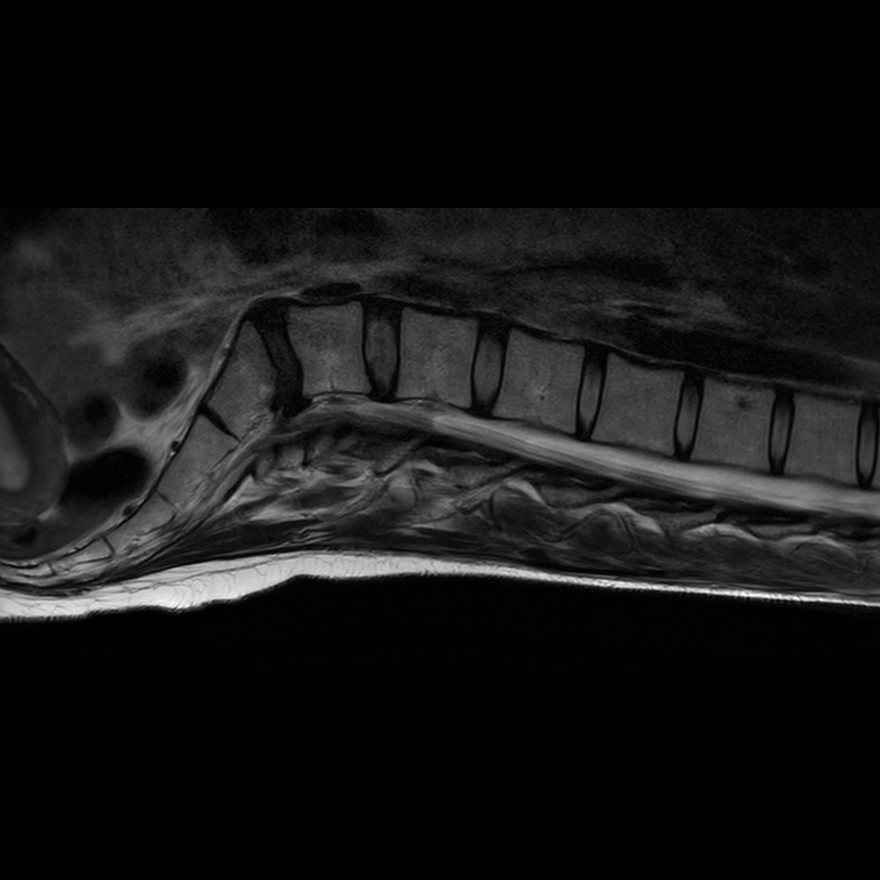

Supplement: S1 File — (ZIP) [file pone.0248303.s001.zip › Code and data/dataset/train/43.png]

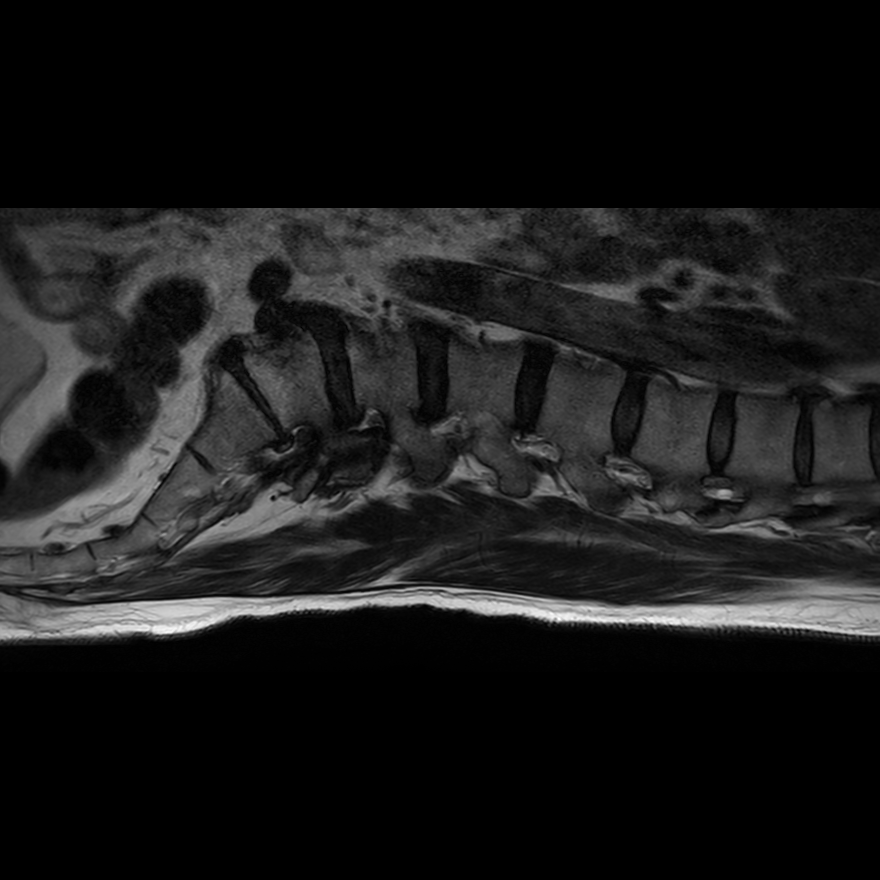

Supplement: S1 File — (ZIP) [file pone.0248303.s001.zip › Code and data/dataset/train/44.png]

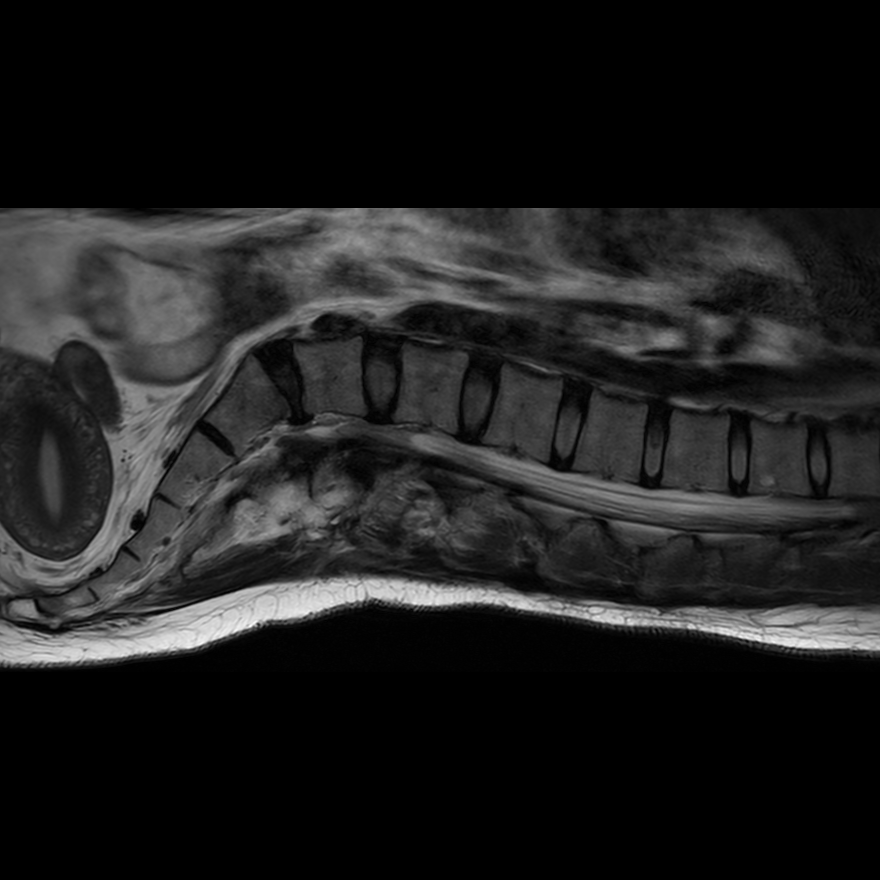

Supplement: S1 File — (ZIP) [file pone.0248303.s001.zip › Code and data/dataset/train/45.png]

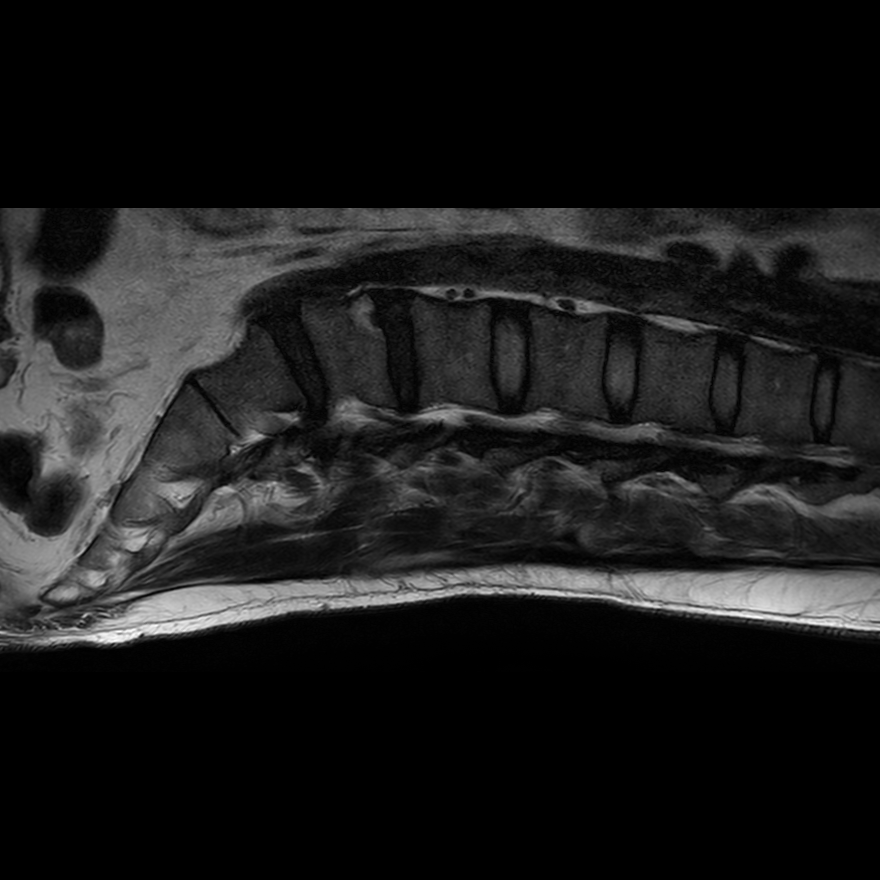

Supplement: S1 File — (ZIP) [file pone.0248303.s001.zip › Code and data/dataset/train/46.png]

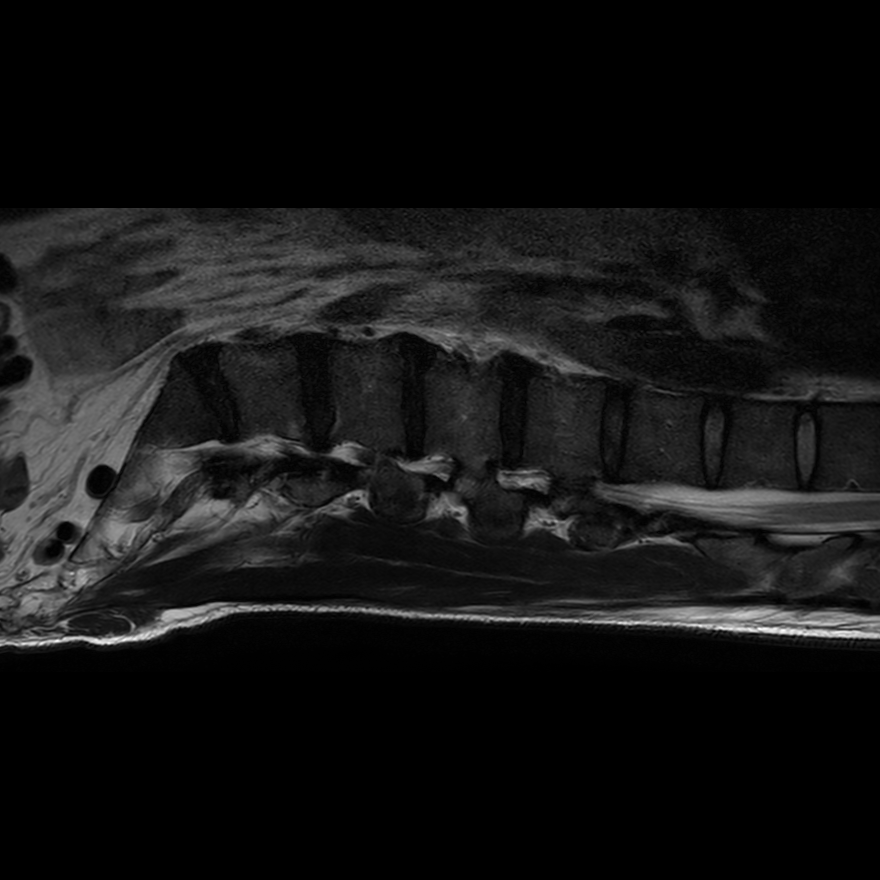

Supplement: S1 File — (ZIP) [file pone.0248303.s001.zip › Code and data/dataset/train/47.png]

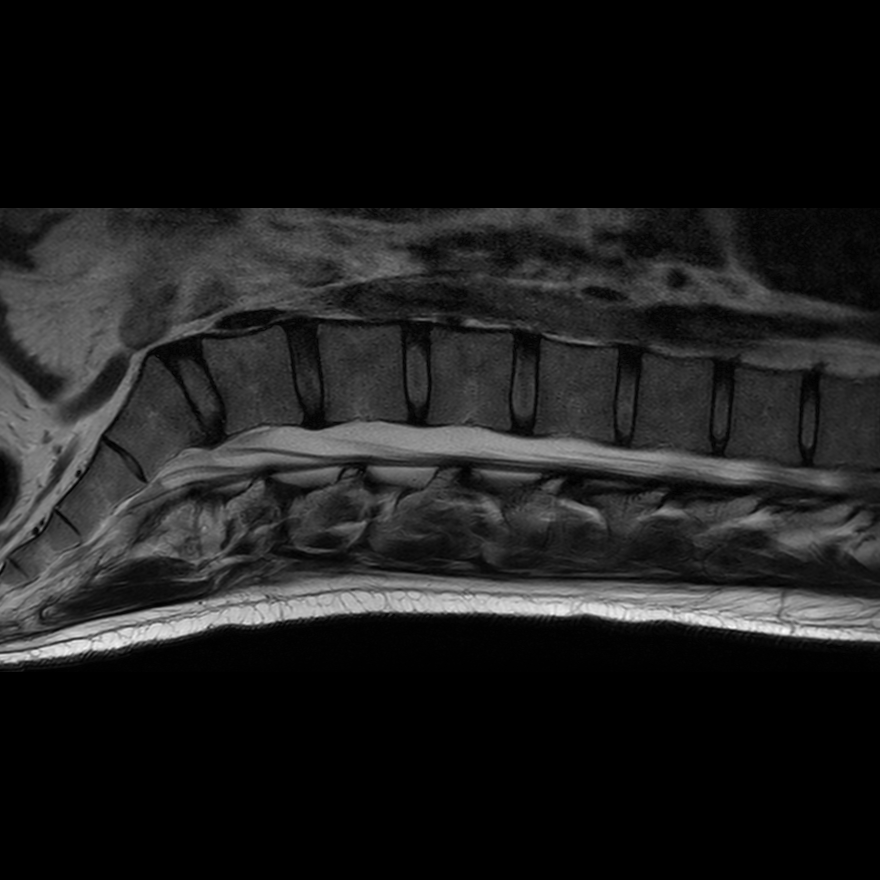

Supplement: S1 File — (ZIP) [file pone.0248303.s001.zip › Code and data/dataset/train/48.png]

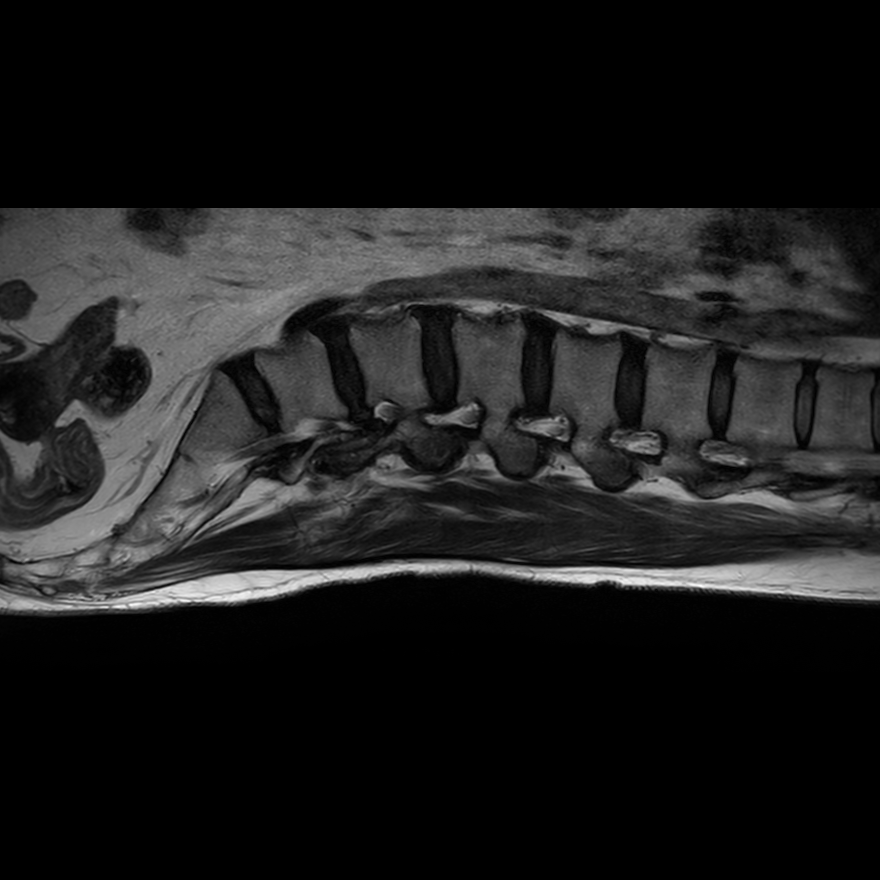

Supplement: S1 File — (ZIP) [file pone.0248303.s001.zip › Code and data/dataset/train/49.png]

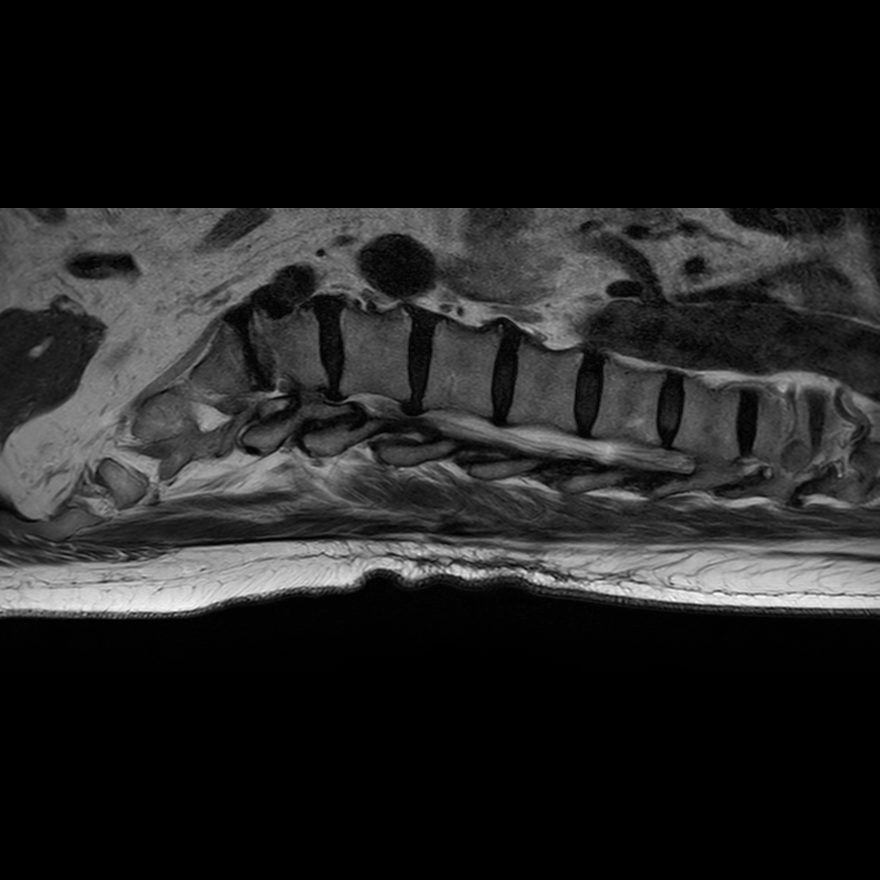

Supplement: S1 File — (ZIP) [file pone.0248303.s001.zip › Code and data/dataset/train/5.png]

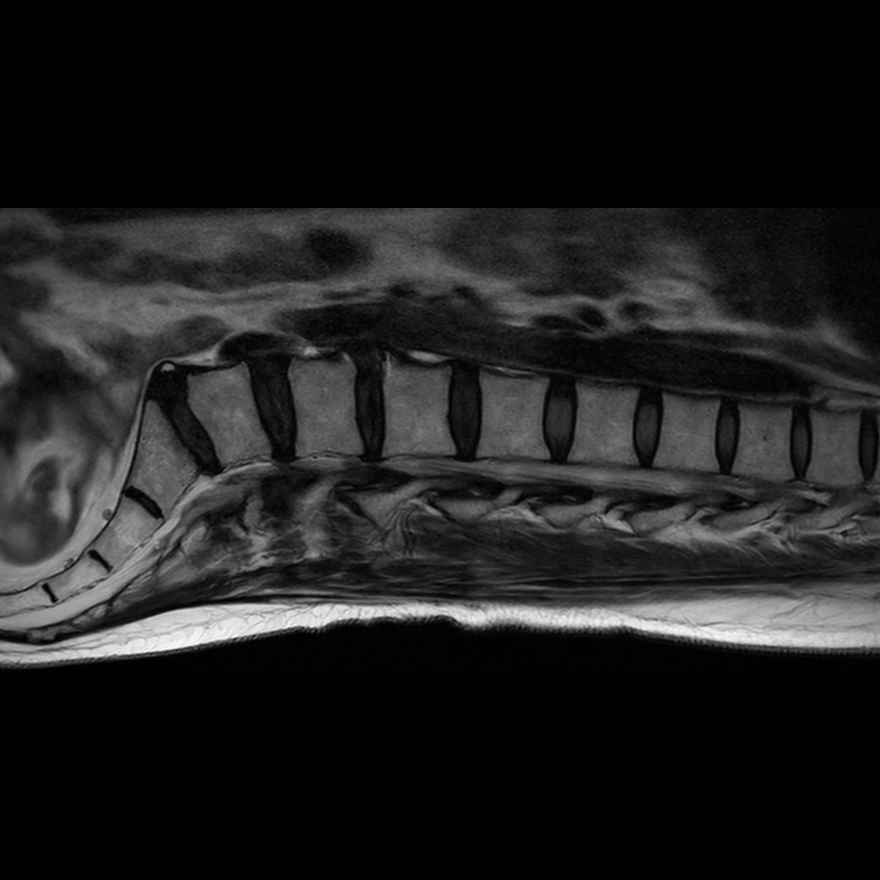

Supplement: S1 File — (ZIP) [file pone.0248303.s001.zip › Code and data/dataset/train/50.png]

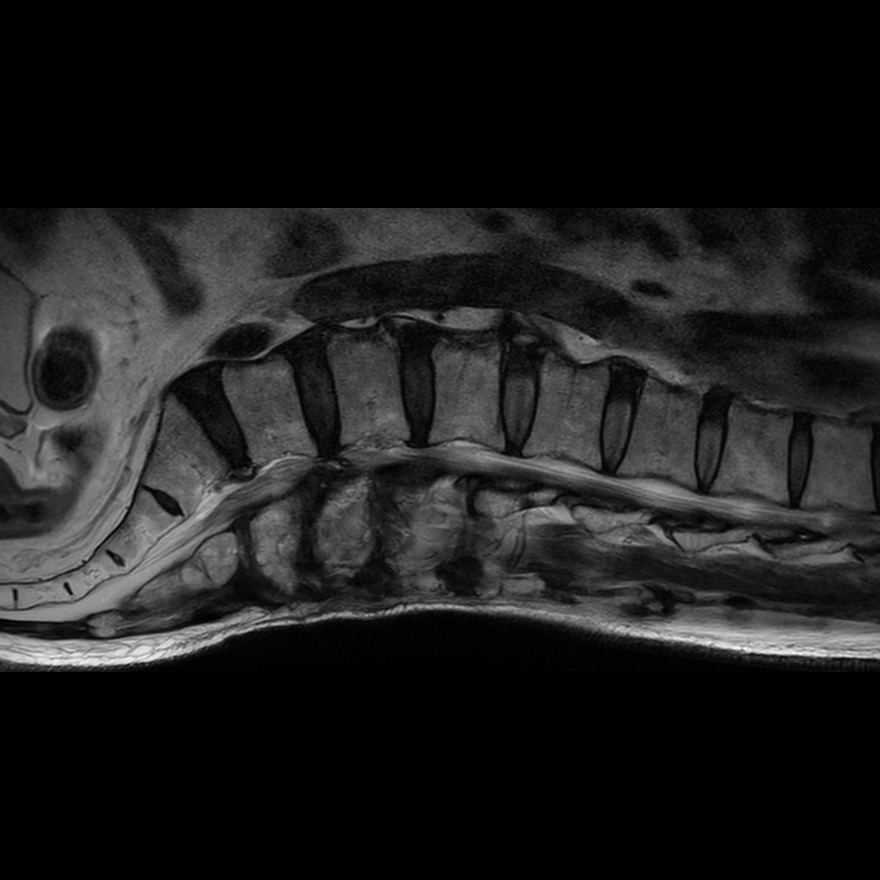

Supplement: S1 File — (ZIP) [file pone.0248303.s001.zip › Code and data/dataset/train/51.png]

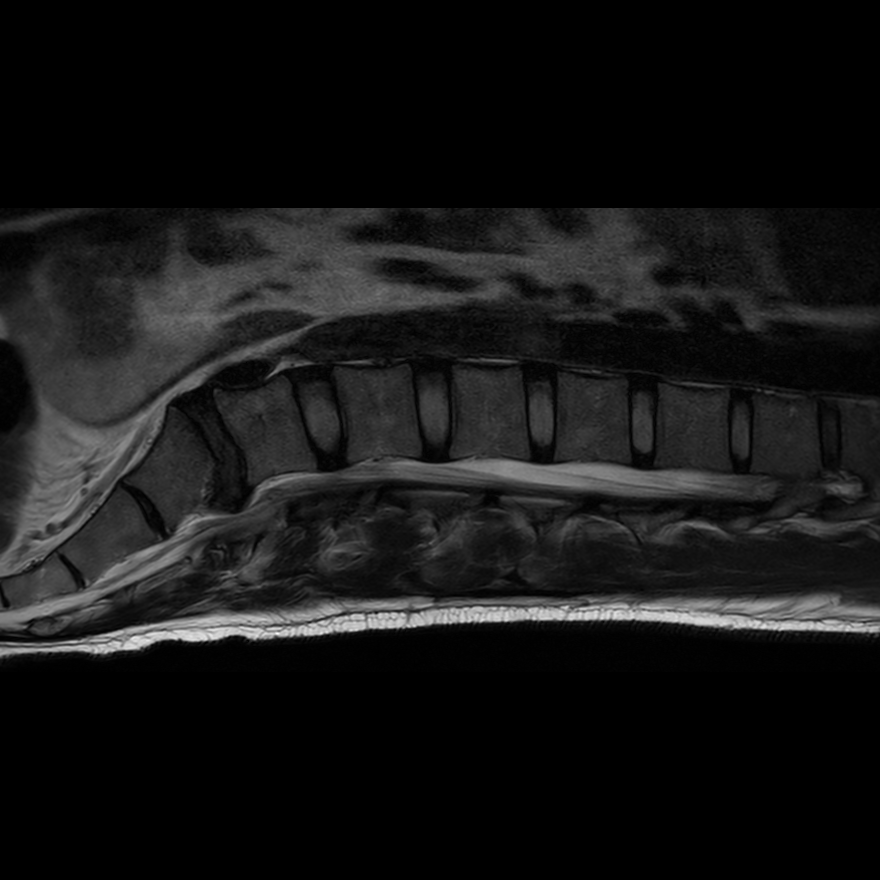

Supplement: S1 File — (ZIP) [file pone.0248303.s001.zip › Code and data/dataset/train/52.png]

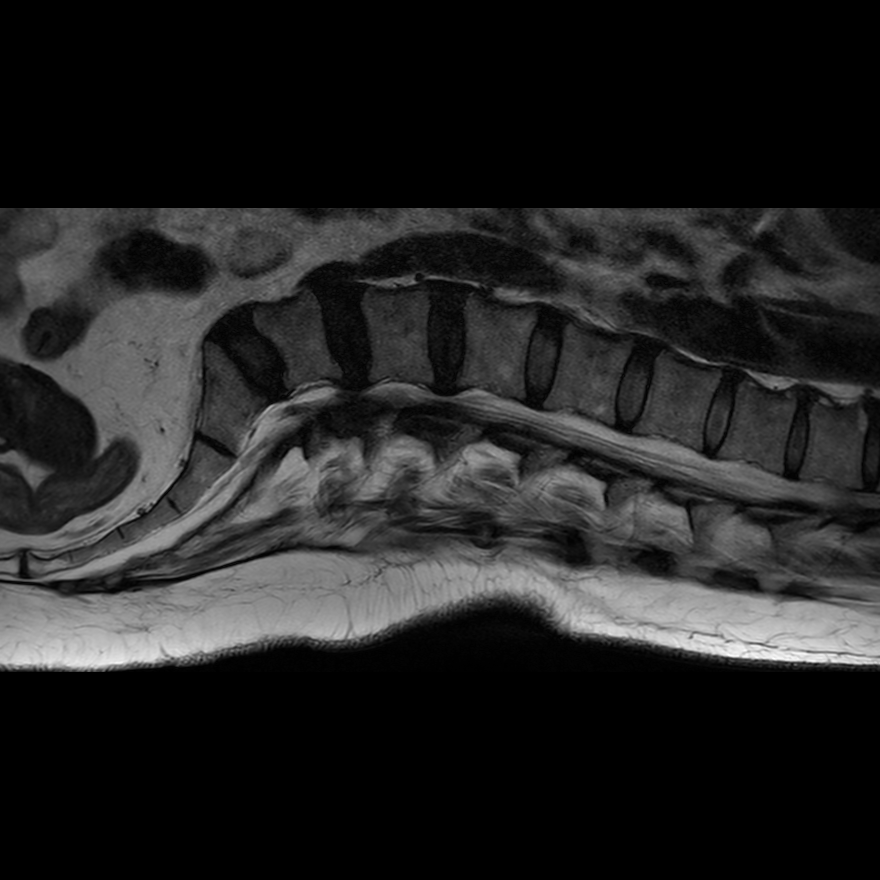

Supplement: S1 File — (ZIP) [file pone.0248303.s001.zip › Code and data/dataset/train/53.png]

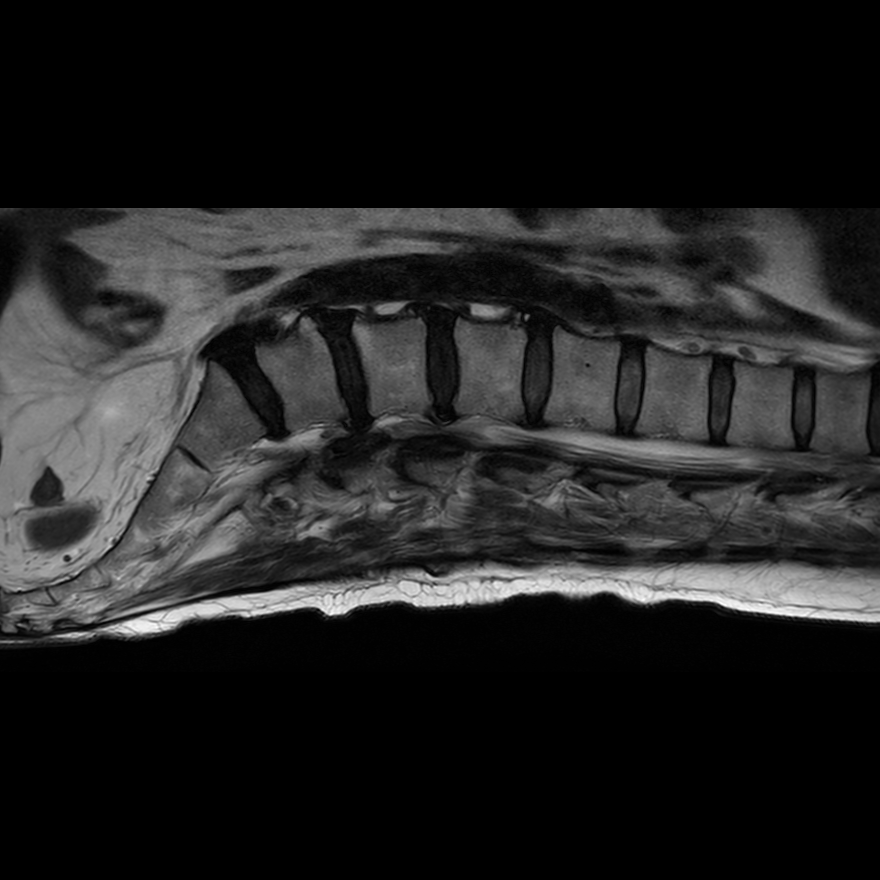

Supplement: S1 File — (ZIP) [file pone.0248303.s001.zip › Code and data/dataset/train/54.png]

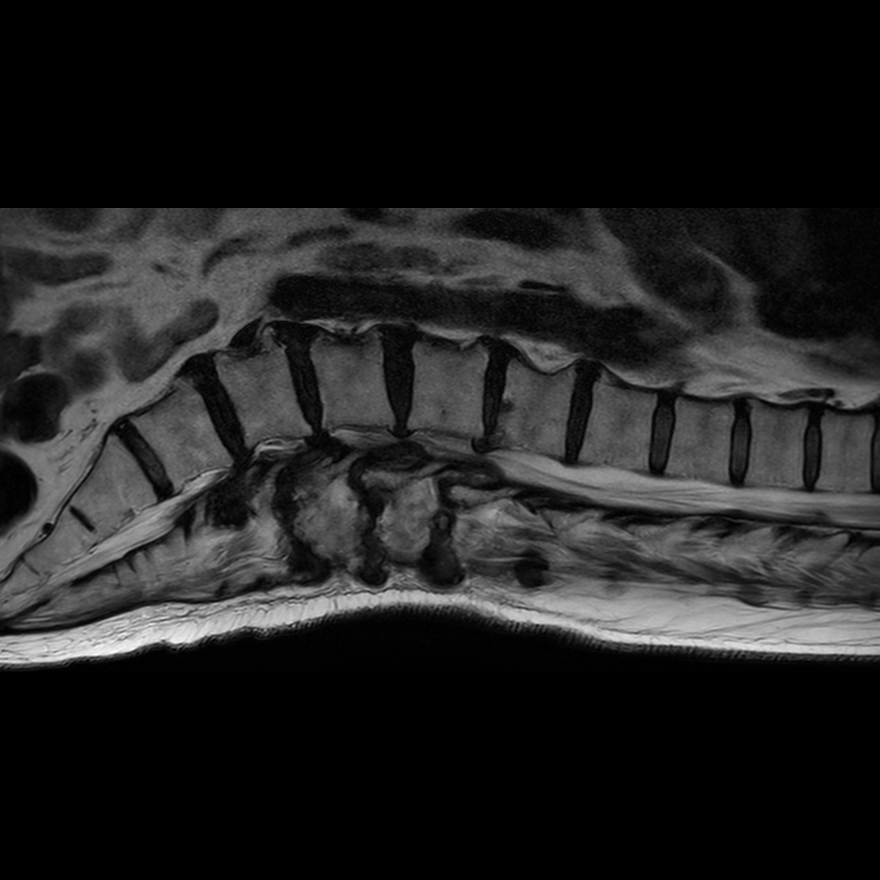

Supplement: S1 File — (ZIP) [file pone.0248303.s001.zip › Code and data/dataset/train/55.png]

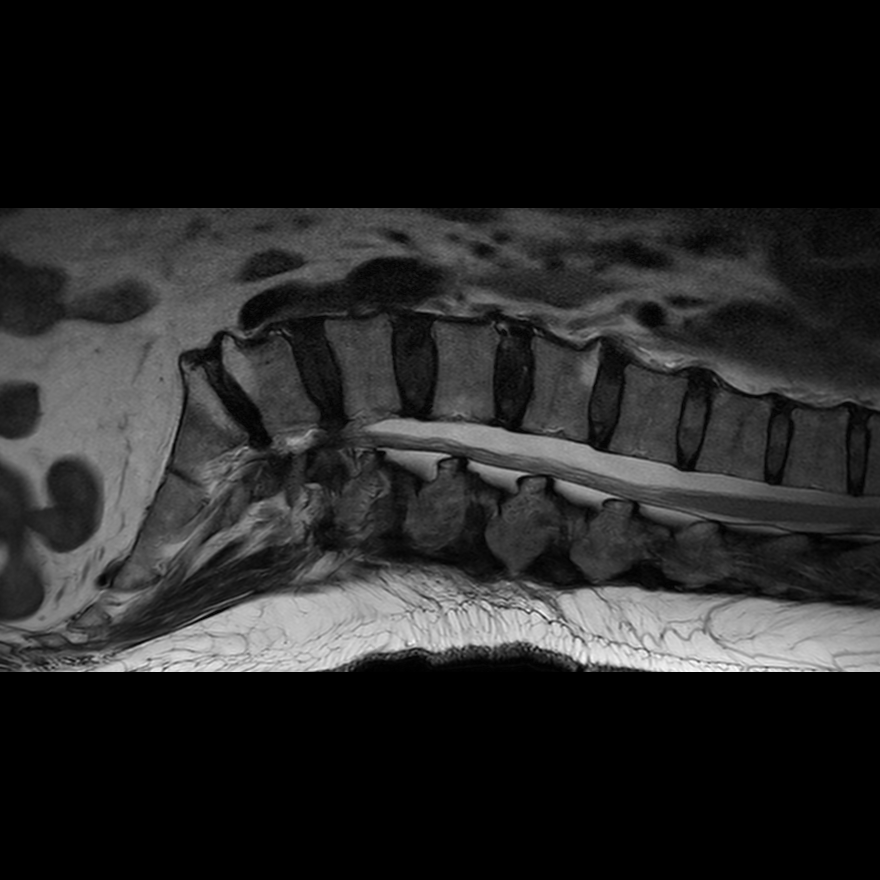

Supplement: S1 File — (ZIP) [file pone.0248303.s001.zip › Code and data/dataset/train/56.png]

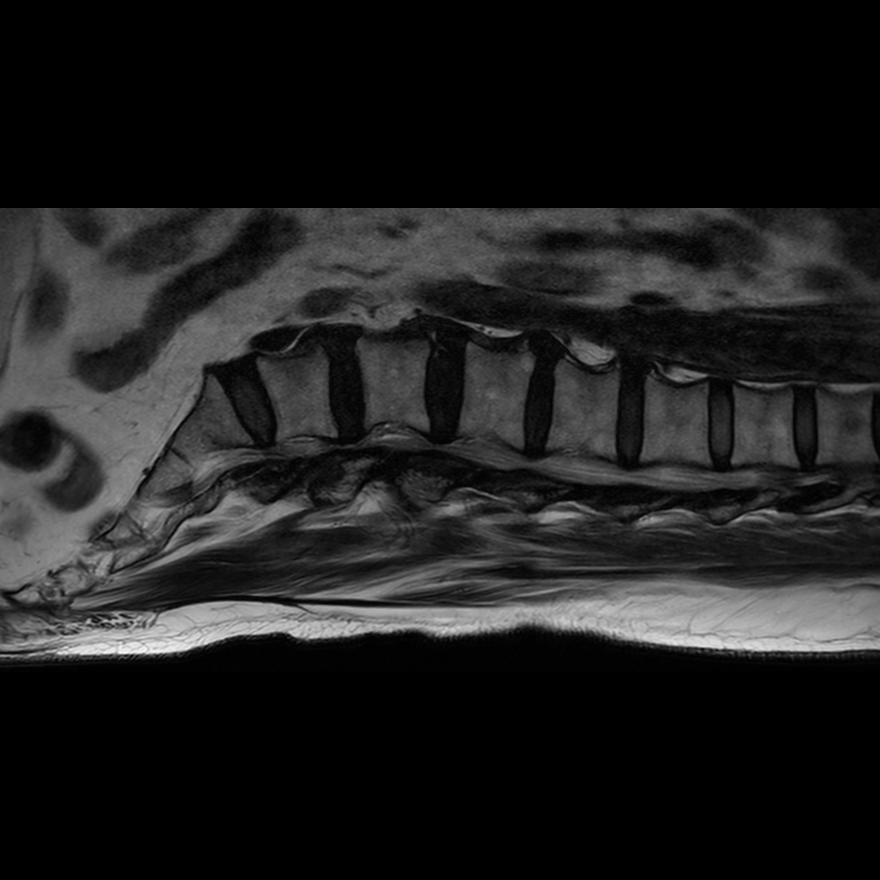

Supplement: S1 File — (ZIP) [file pone.0248303.s001.zip › Code and data/dataset/train/57.png]

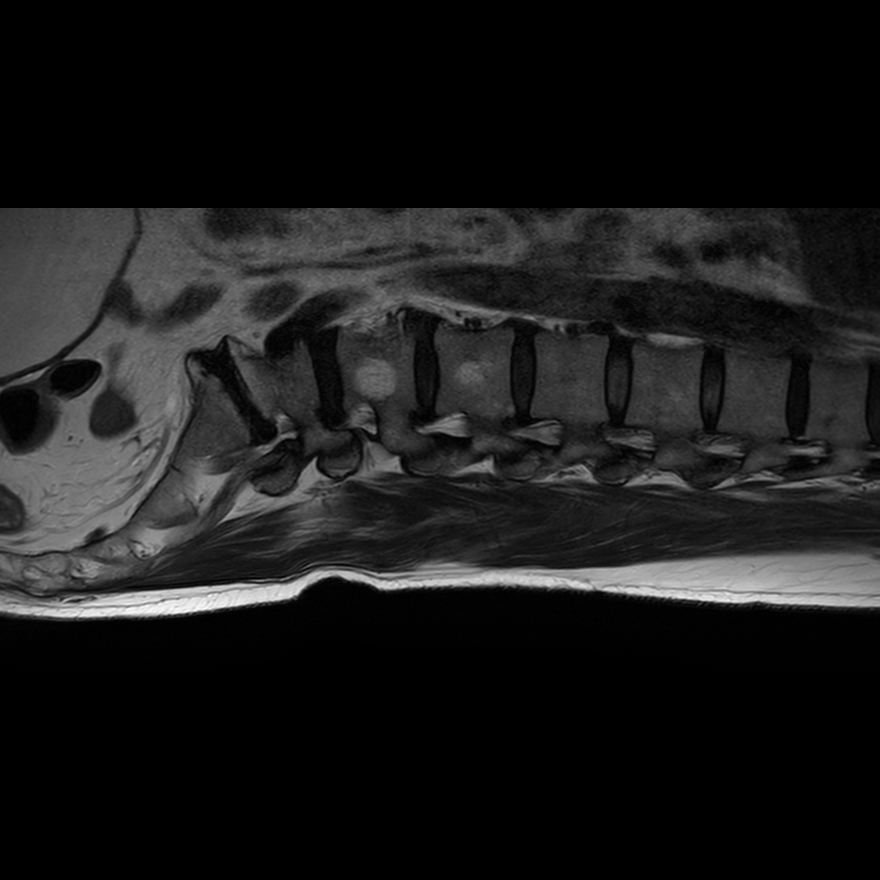

Supplement: S1 File — (ZIP) [file pone.0248303.s001.zip › Code and data/dataset/train/58.png]

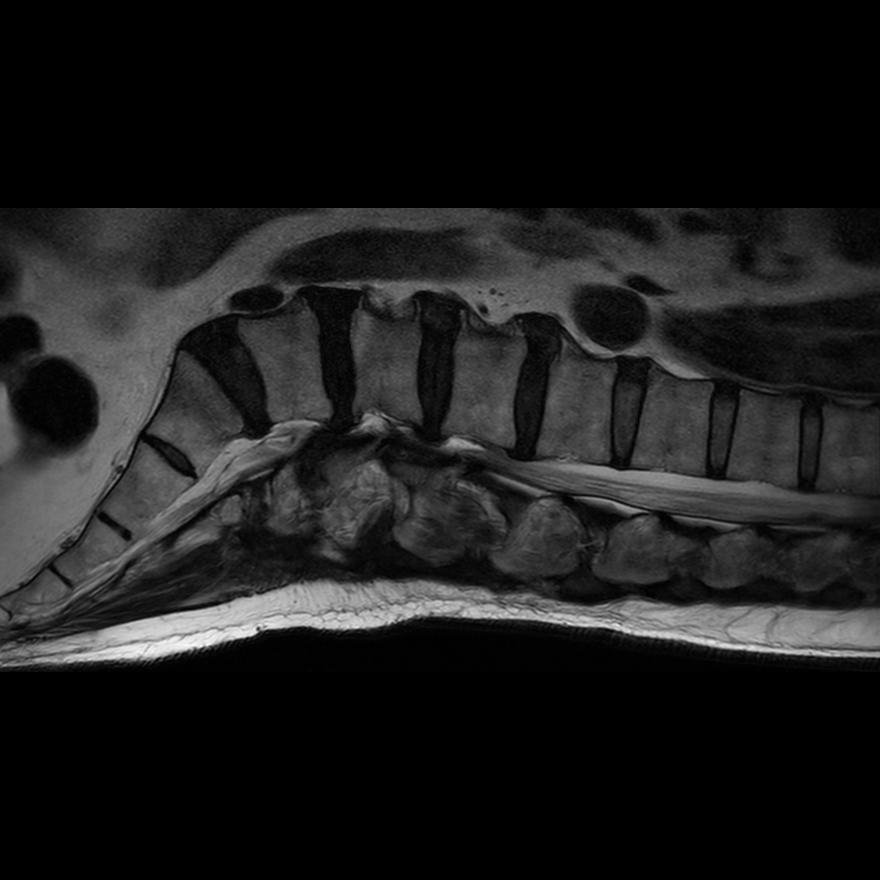

Supplement: S1 File — (ZIP) [file pone.0248303.s001.zip › Code and data/dataset/train/59.png]

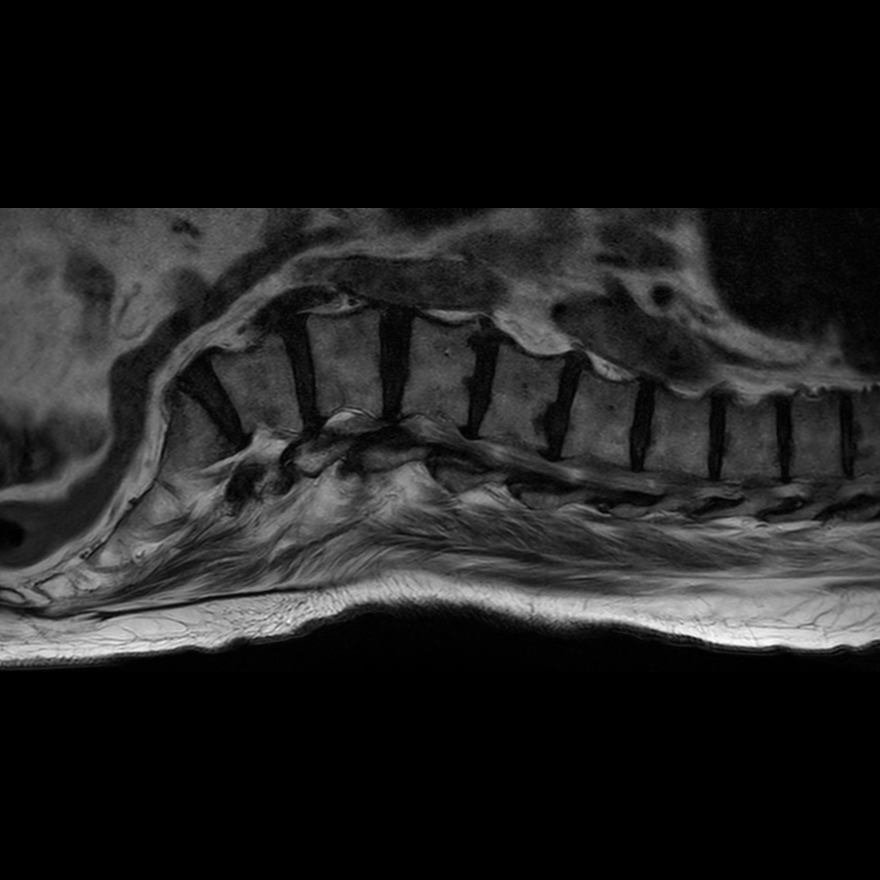

Supplement: S1 File — (ZIP) [file pone.0248303.s001.zip › Code and data/dataset/train/6.png]

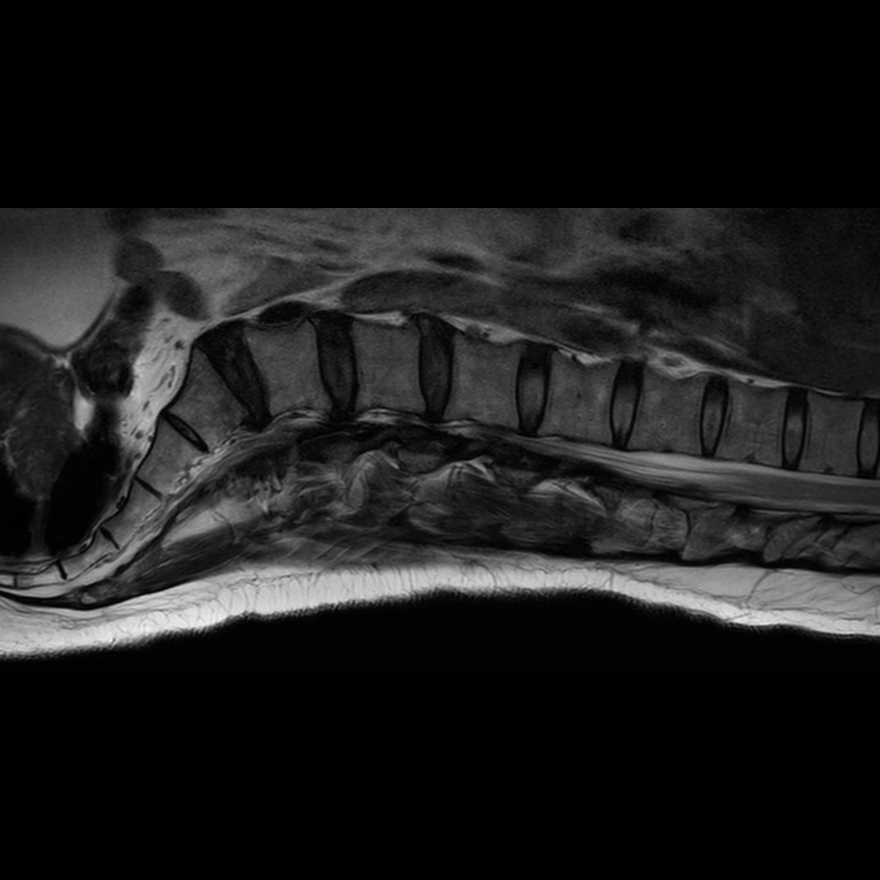

Supplement: S1 File — (ZIP) [file pone.0248303.s001.zip › Code and data/dataset/train/60.png]

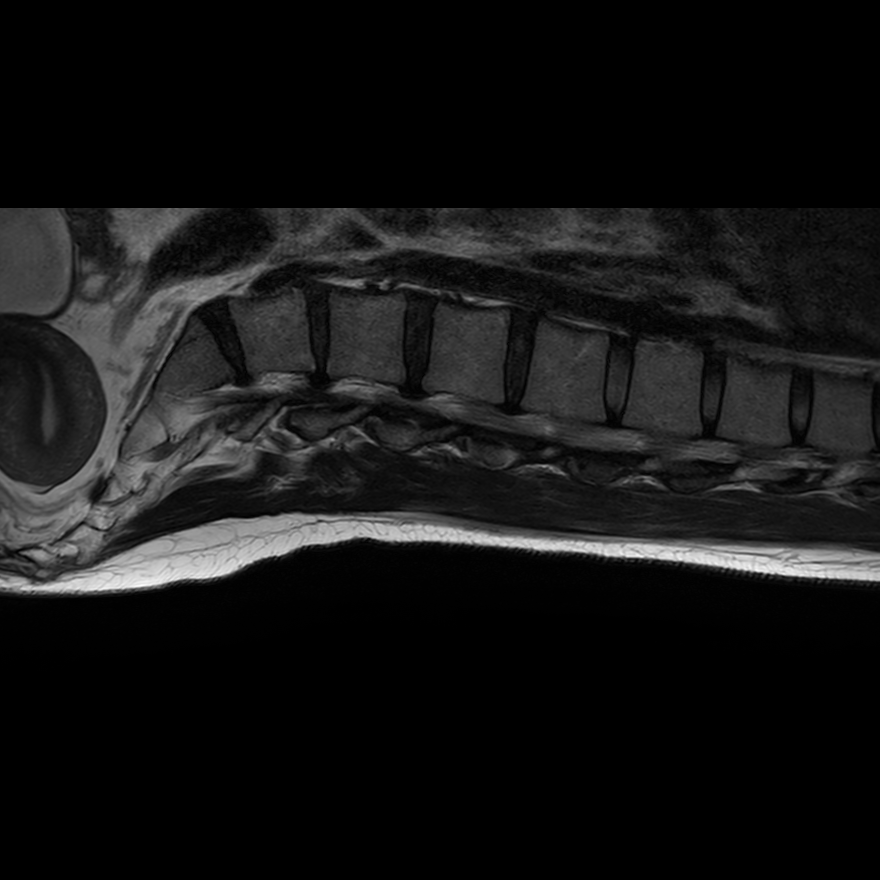

Supplement: S1 File — (ZIP) [file pone.0248303.s001.zip › Code and data/dataset/train/61.png]

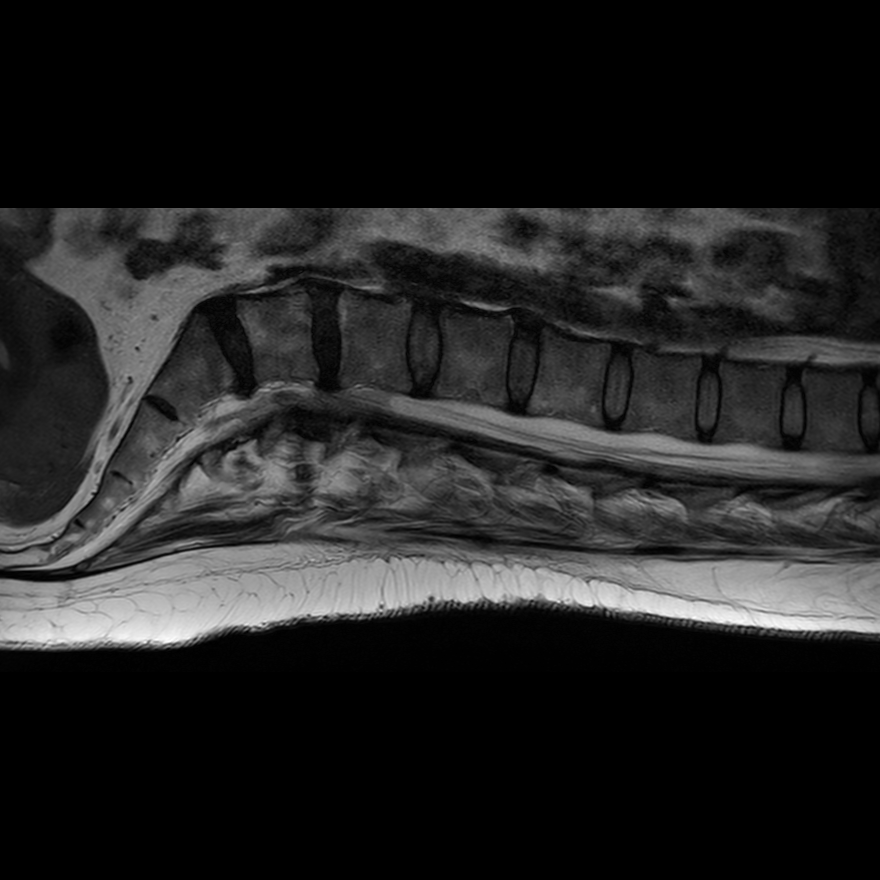

Supplement: S1 File — (ZIP) [file pone.0248303.s001.zip › Code and data/dataset/train/62.png]

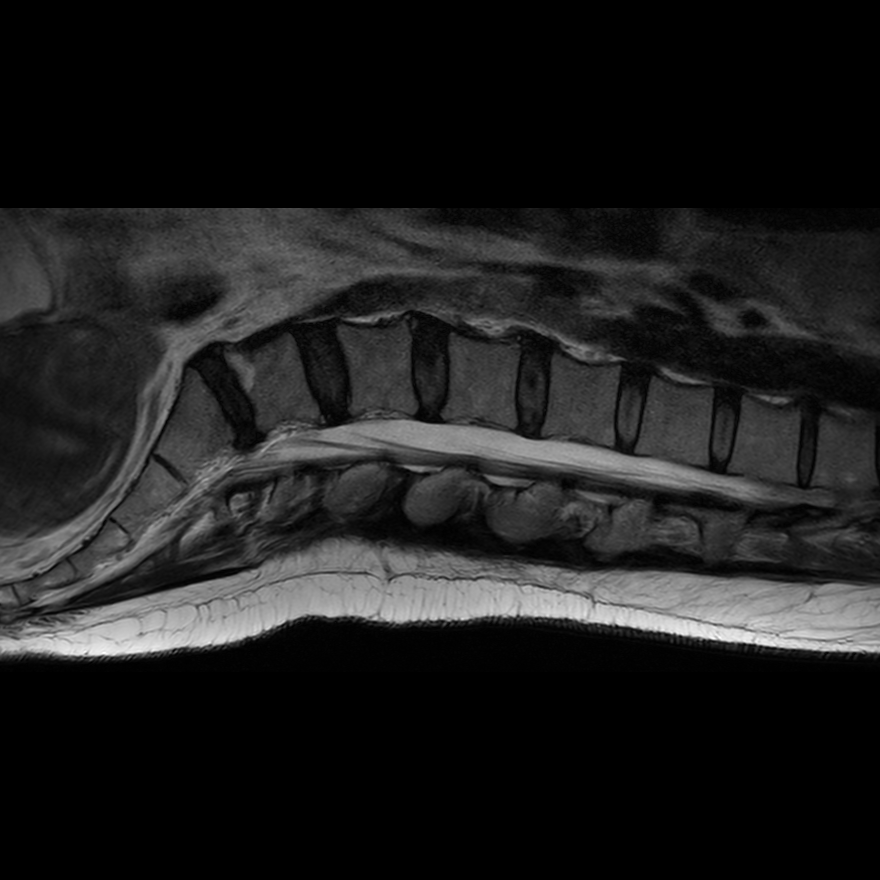

Supplement: S1 File — (ZIP) [file pone.0248303.s001.zip › Code and data/dataset/train/63.png]

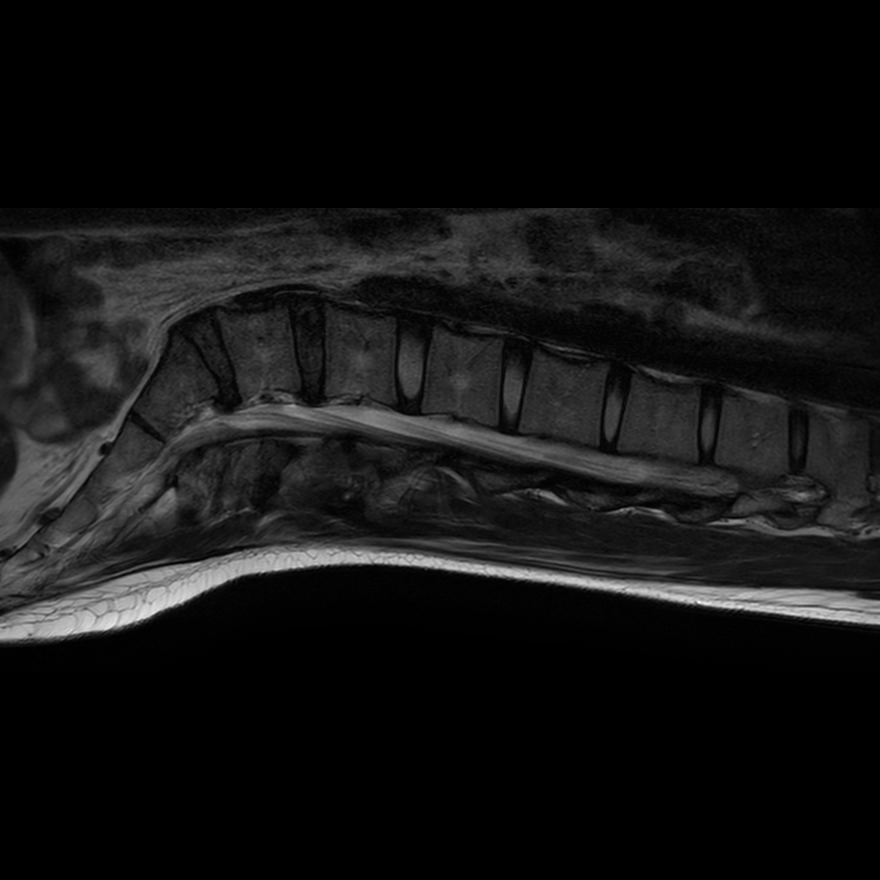

Supplement: S1 File — (ZIP) [file pone.0248303.s001.zip › Code and data/dataset/train/64.png]

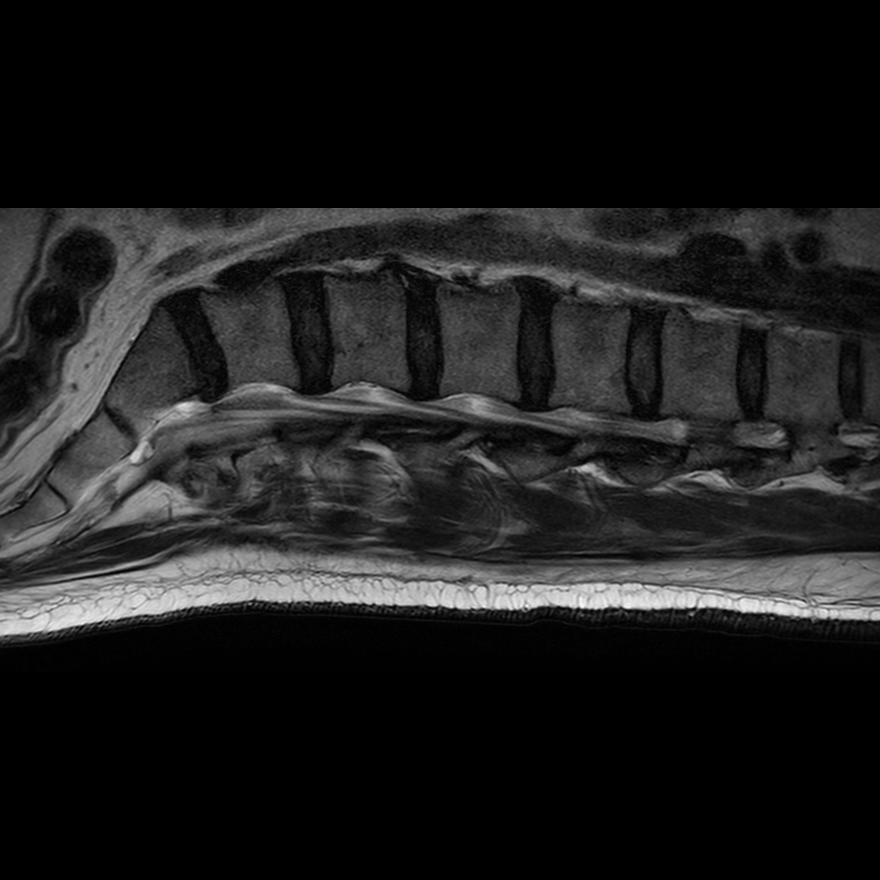

Supplement: S1 File — (ZIP) [file pone.0248303.s001.zip › Code and data/dataset/train/65.png]

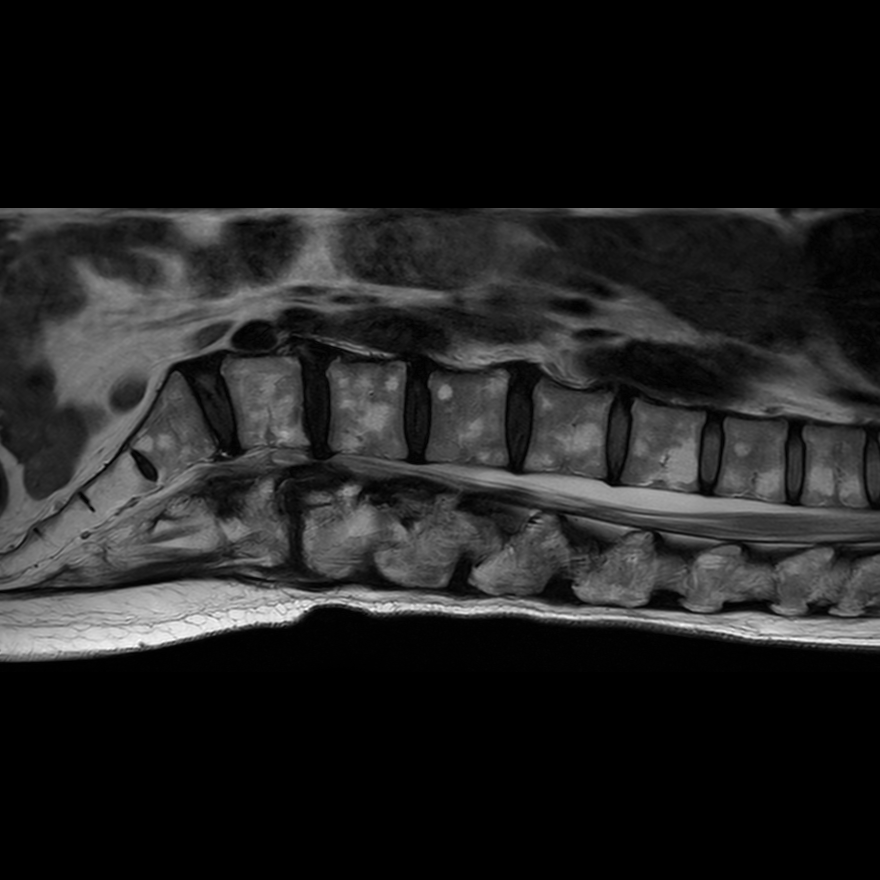

Supplement: S1 File — (ZIP) [file pone.0248303.s001.zip › Code and data/dataset/train/66.png]

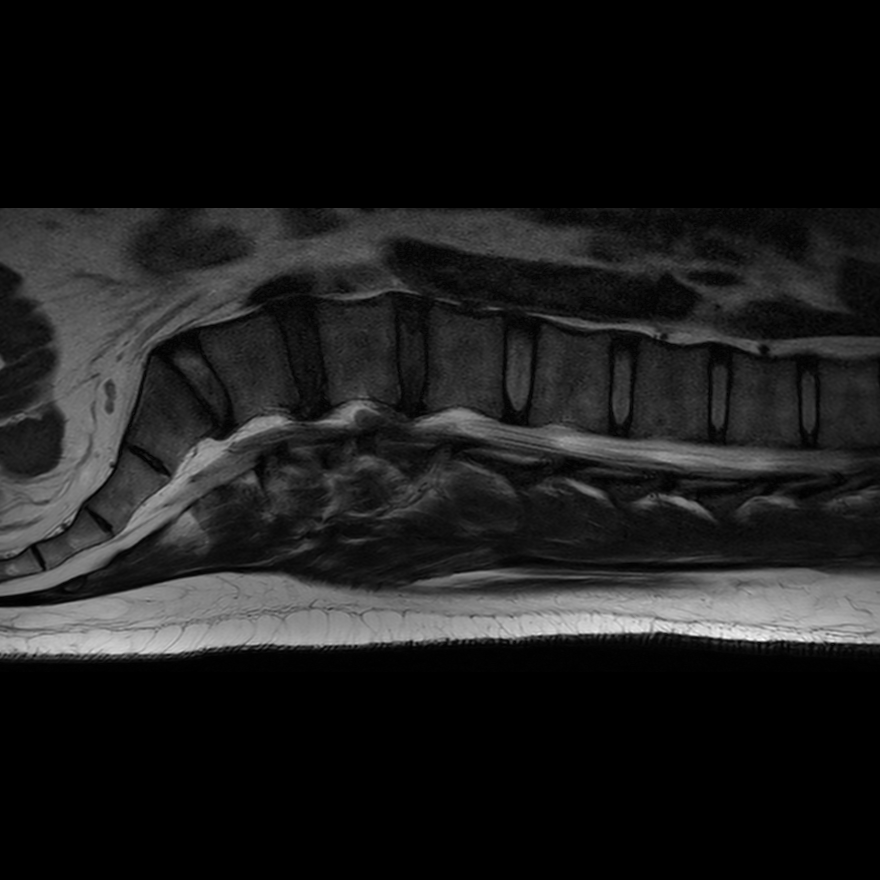

Supplement: S1 File — (ZIP) [file pone.0248303.s001.zip › Code and data/dataset/train/67.png]

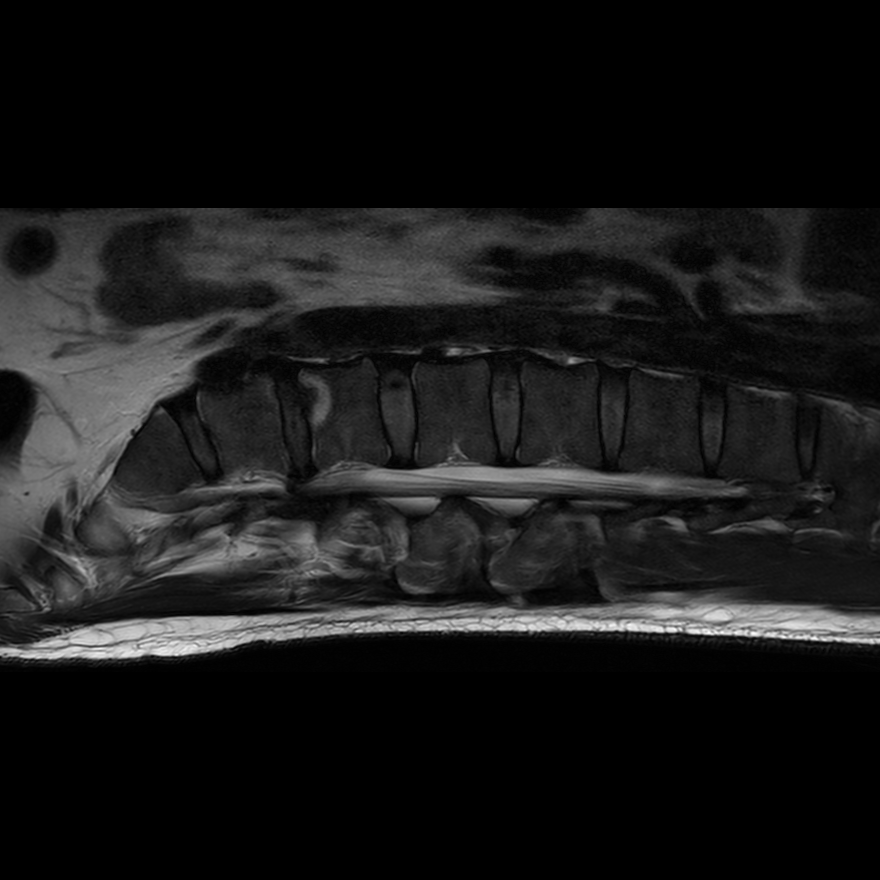

Supplement: S1 File — (ZIP) [file pone.0248303.s001.zip › Code and data/dataset/train/68.png]

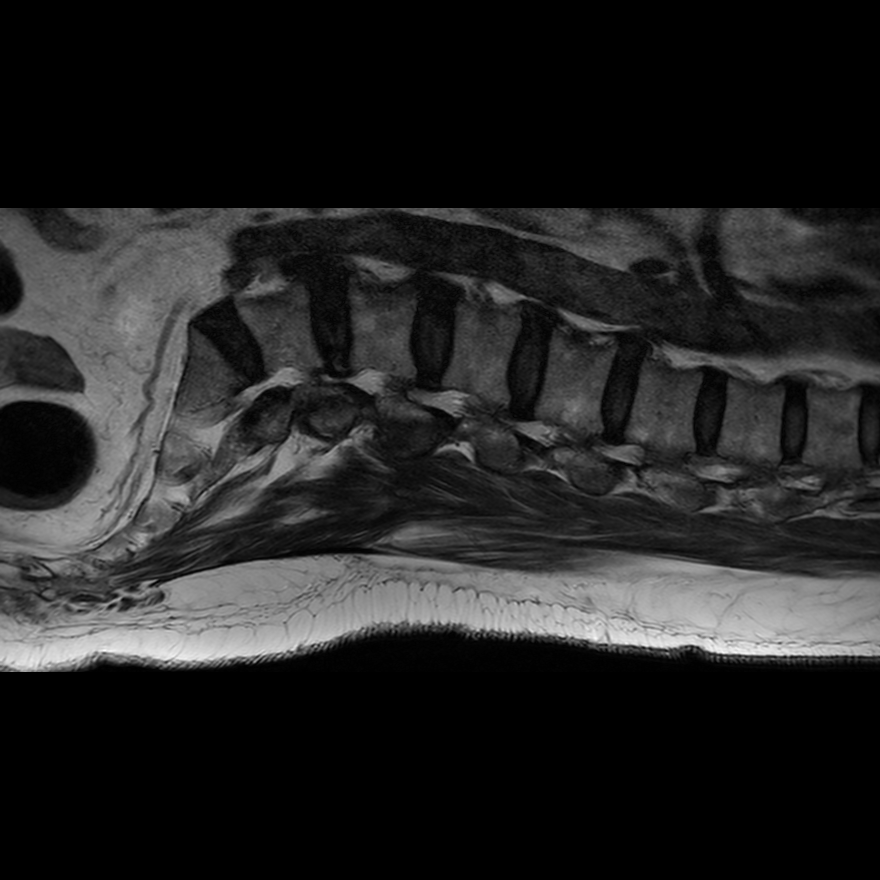

Supplement: S1 File — (ZIP) [file pone.0248303.s001.zip › Code and data/dataset/train/69.png]

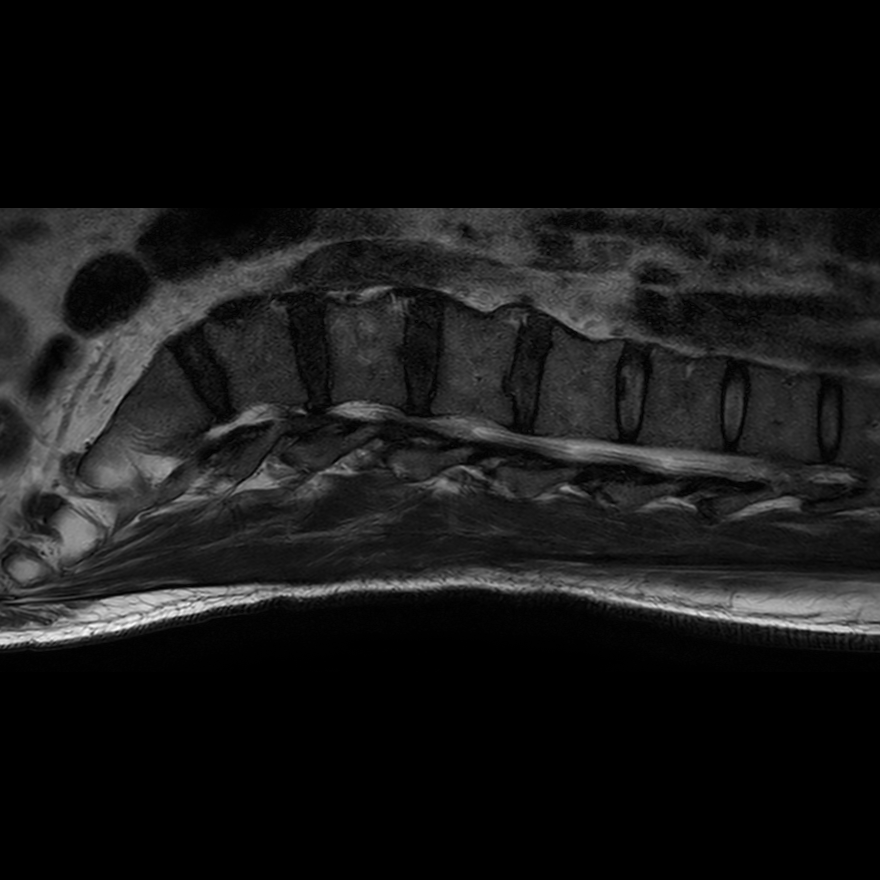

Supplement: S1 File — (ZIP) [file pone.0248303.s001.zip › Code and data/dataset/train/7.png]

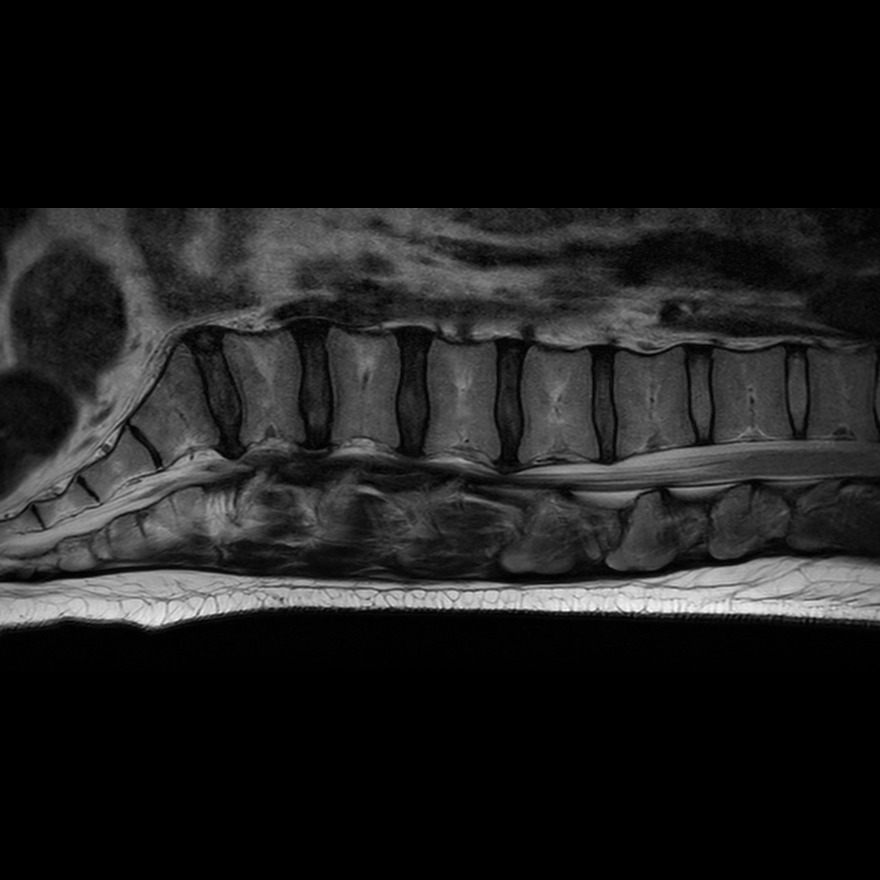

Supplement: S1 File — (ZIP) [file pone.0248303.s001.zip › Code and data/dataset/train/70.png]

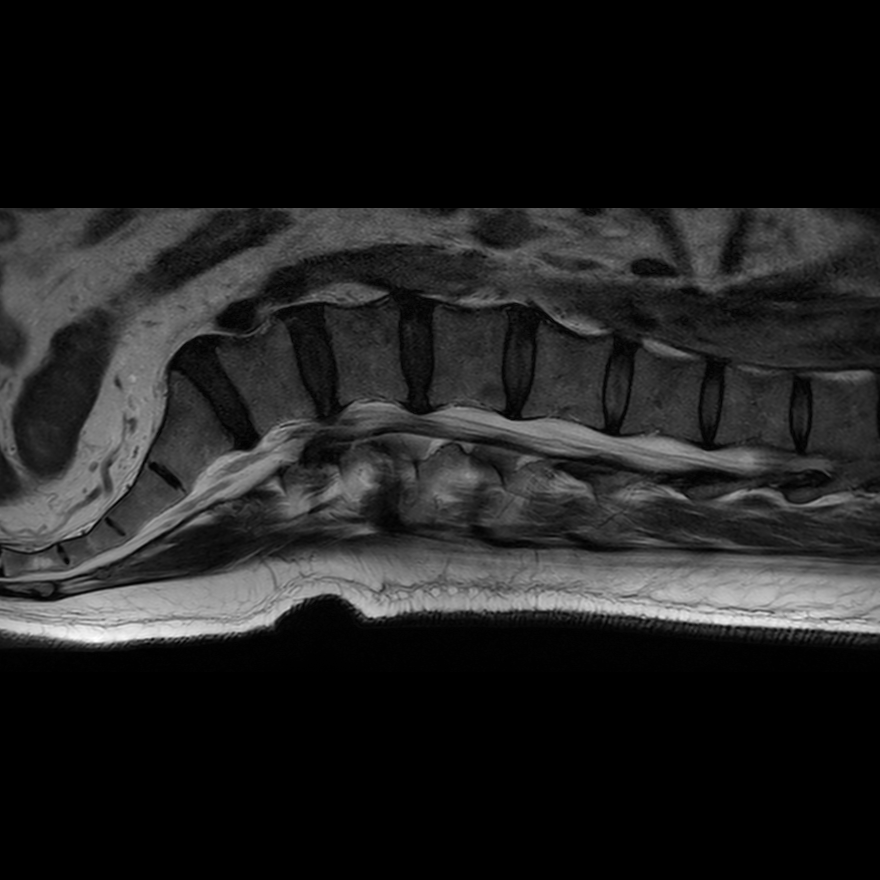

Supplement: S1 File — (ZIP) [file pone.0248303.s001.zip › Code and data/dataset/train/71.png]

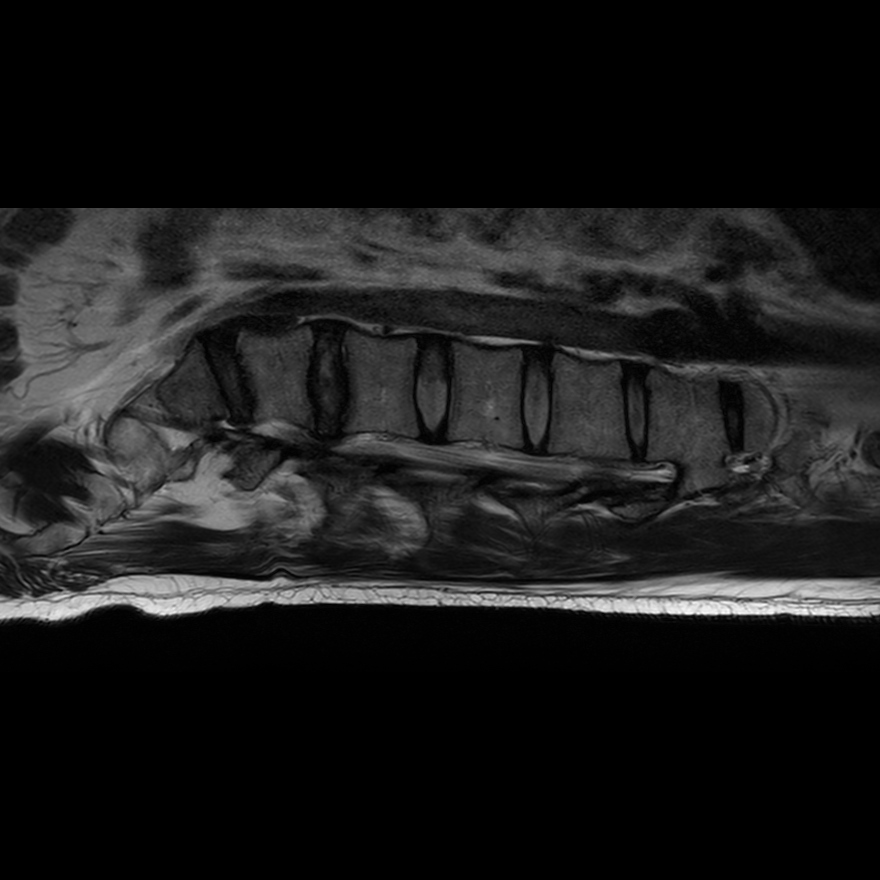

Supplement: S1 File — (ZIP) [file pone.0248303.s001.zip › Code and data/dataset/train/72.png]

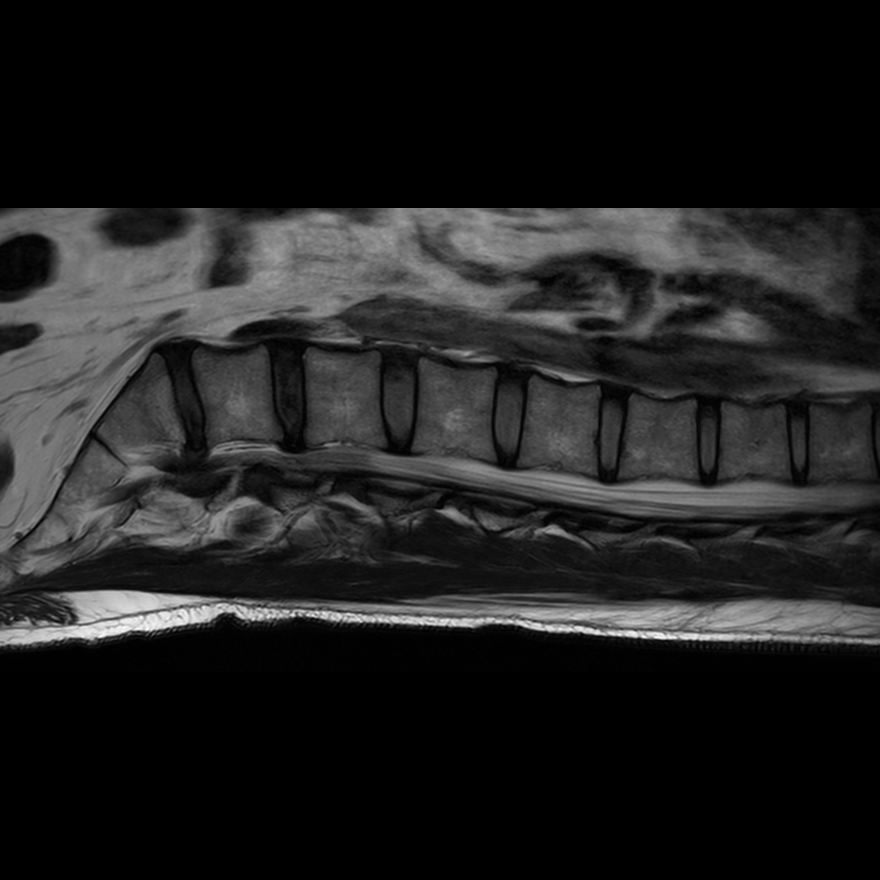

Supplement: S1 File — (ZIP) [file pone.0248303.s001.zip › Code and data/dataset/train/73.png]

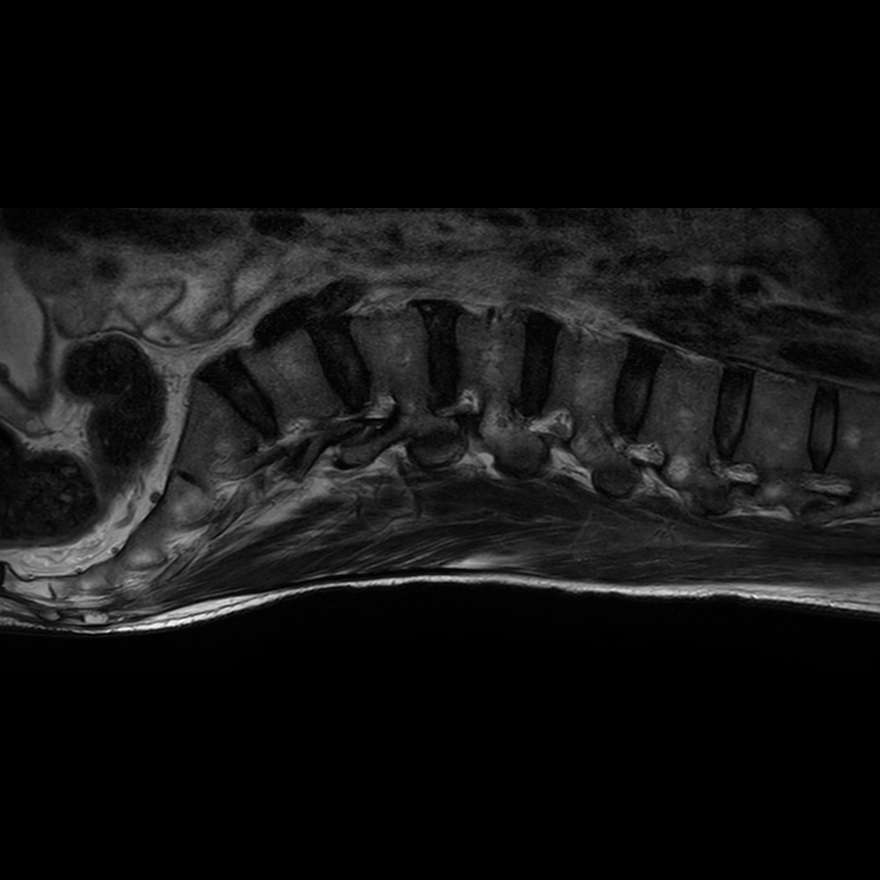

Supplement: S1 File — (ZIP) [file pone.0248303.s001.zip › Code and data/dataset/train/74.png]

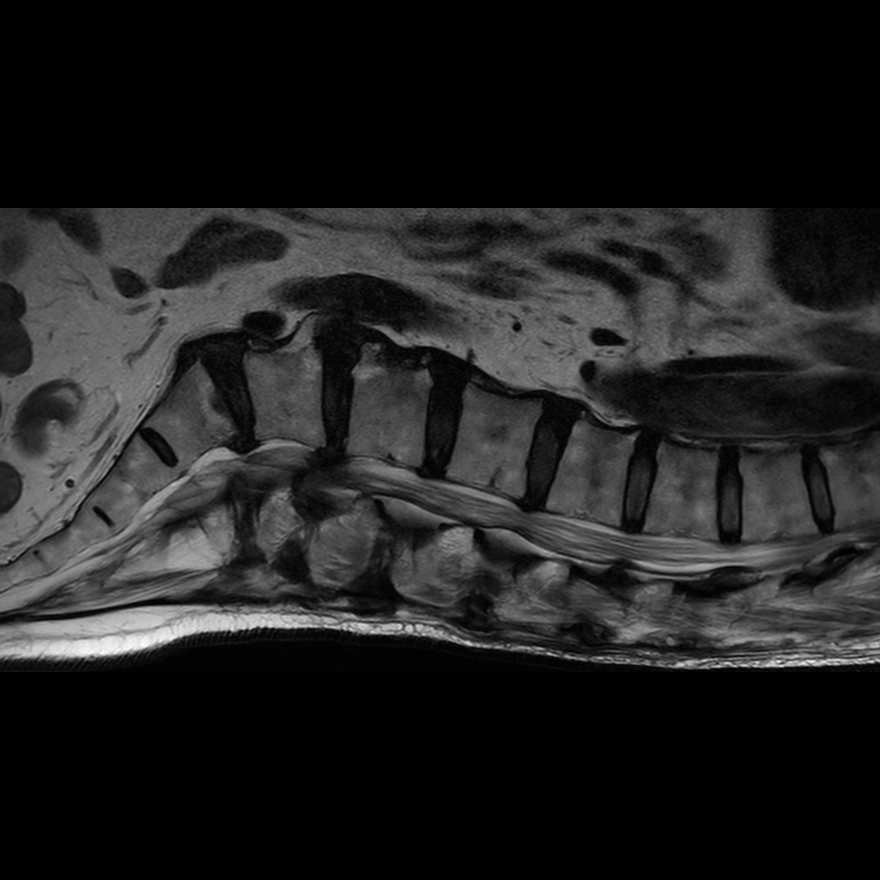

Supplement: S1 File — (ZIP) [file pone.0248303.s001.zip › Code and data/dataset/train/75.png]

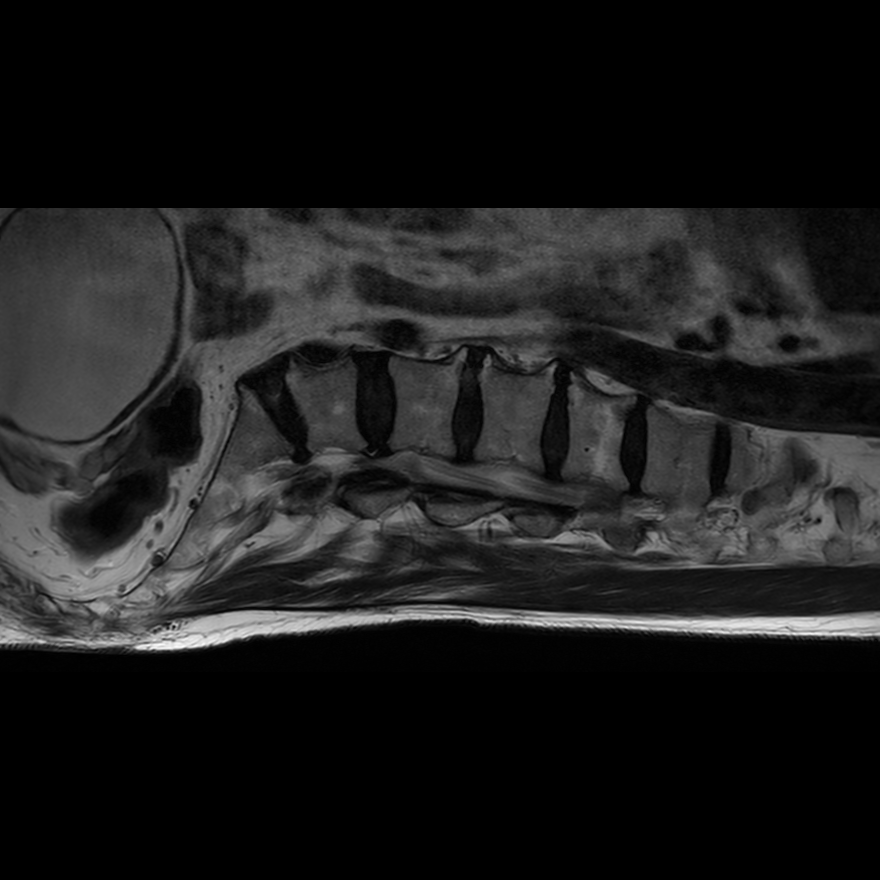

Supplement: S1 File — (ZIP) [file pone.0248303.s001.zip › Code and data/dataset/train/76.png]

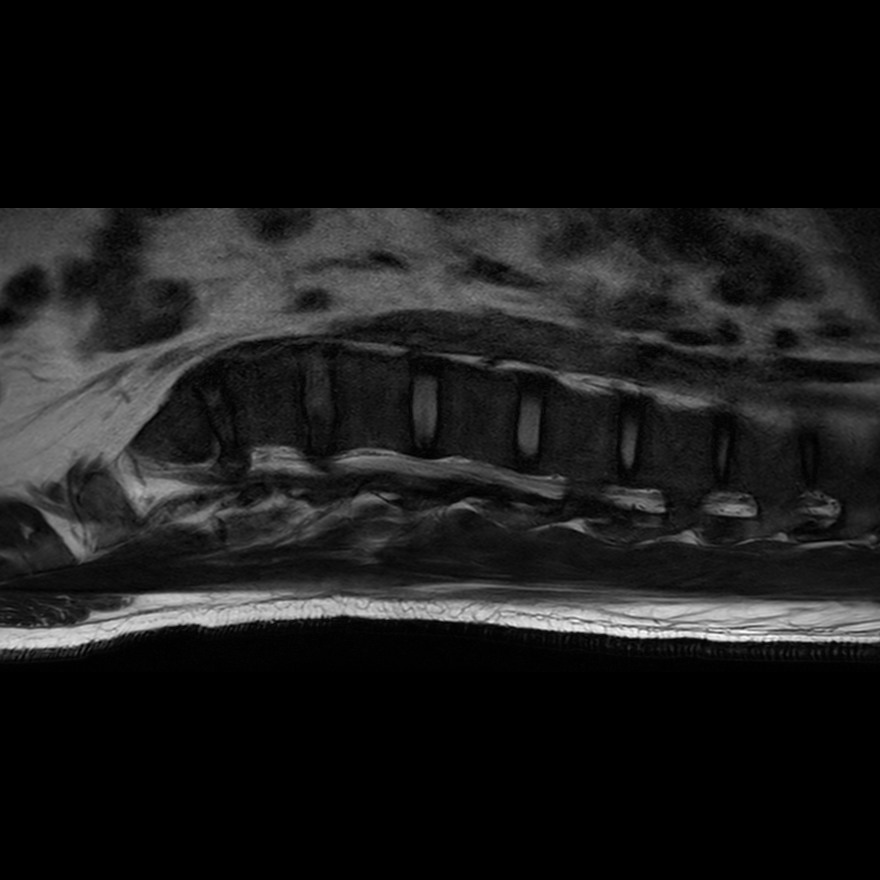

Supplement: S1 File — (ZIP) [file pone.0248303.s001.zip › Code and data/dataset/train/77.png]

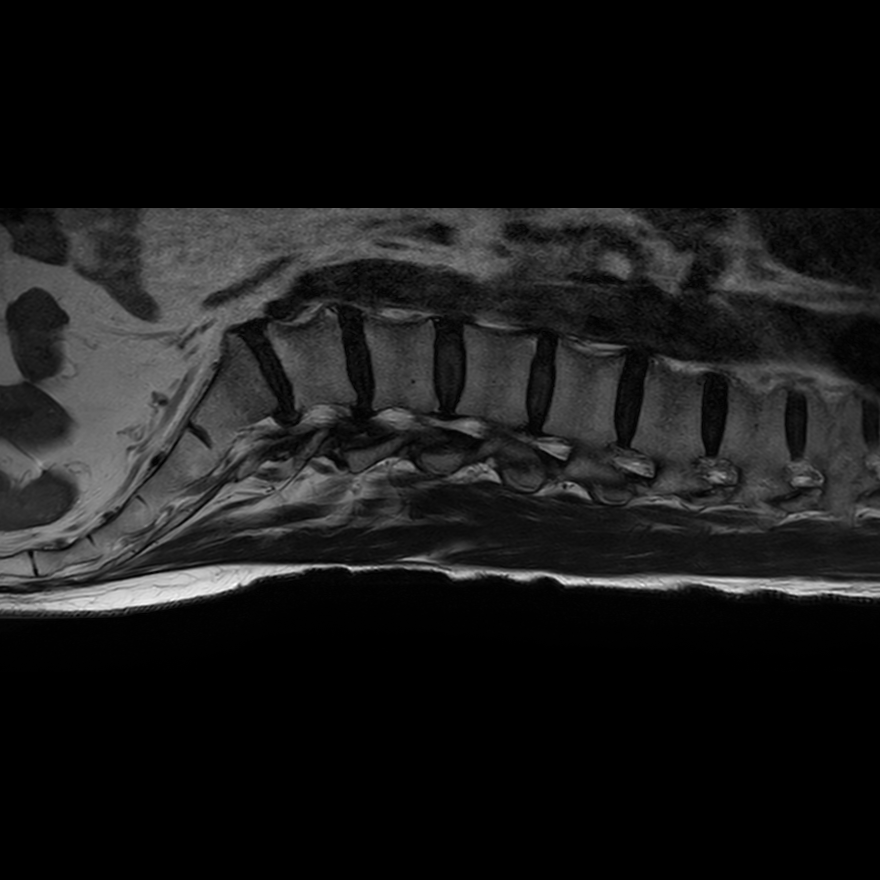

Supplement: S1 File — (ZIP) [file pone.0248303.s001.zip › Code and data/dataset/train/78.png]

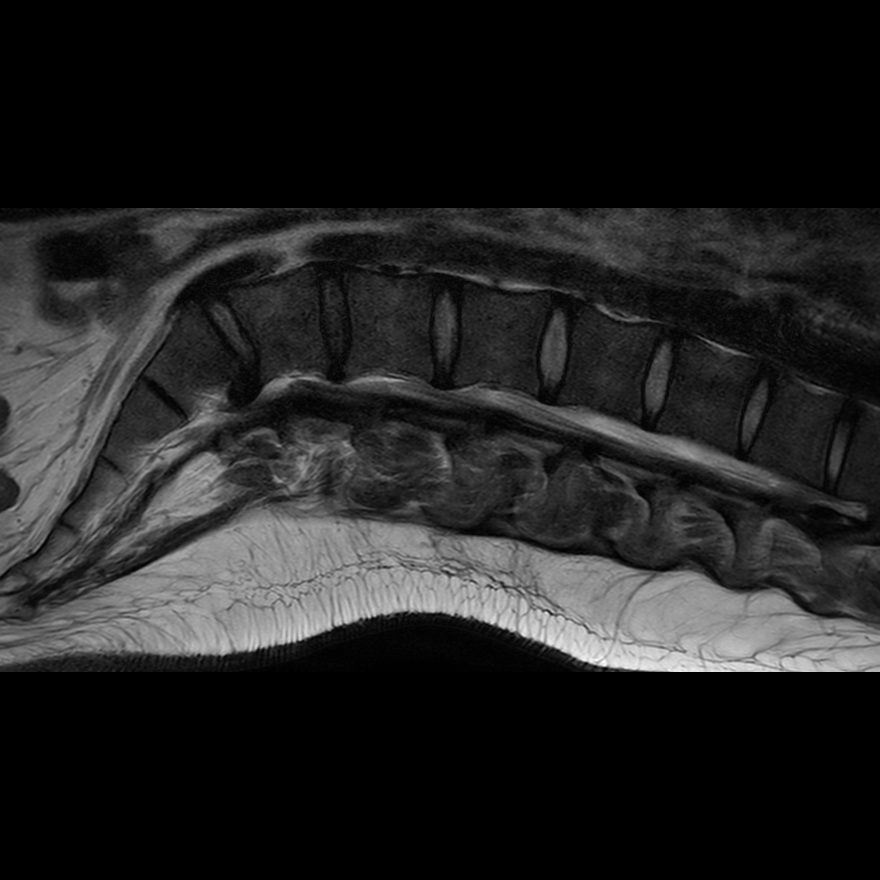

Supplement: S1 File — (ZIP) [file pone.0248303.s001.zip › Code and data/dataset/train/79.png]

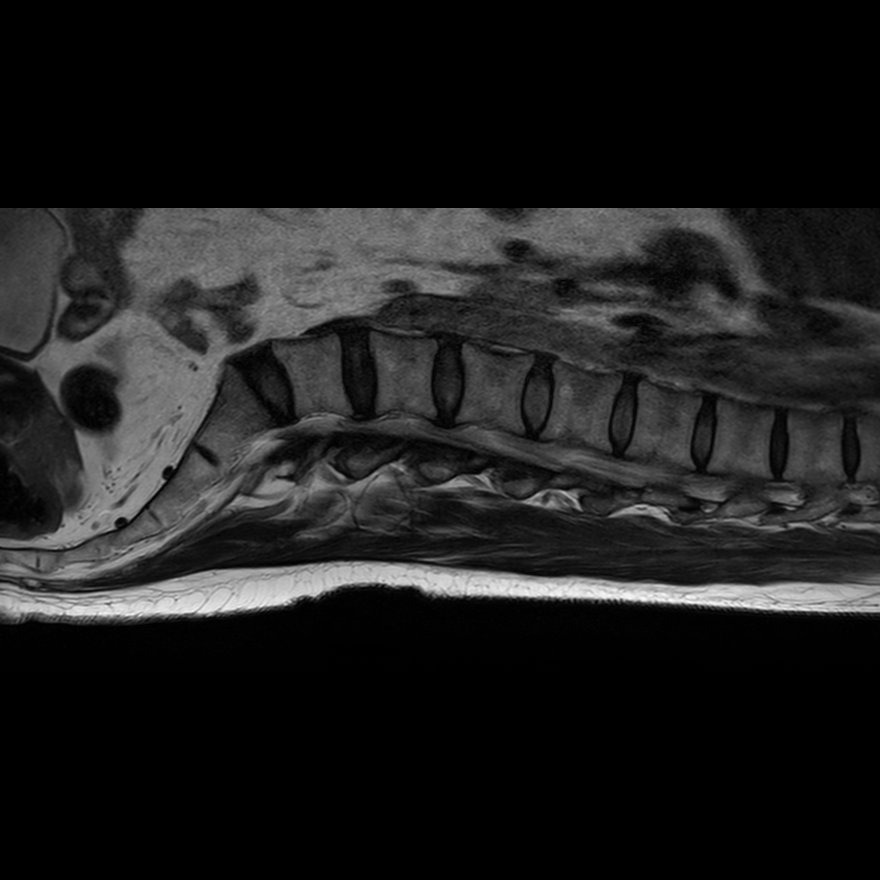

Supplement: S1 File — (ZIP) [file pone.0248303.s001.zip › Code and data/dataset/train/8.png]

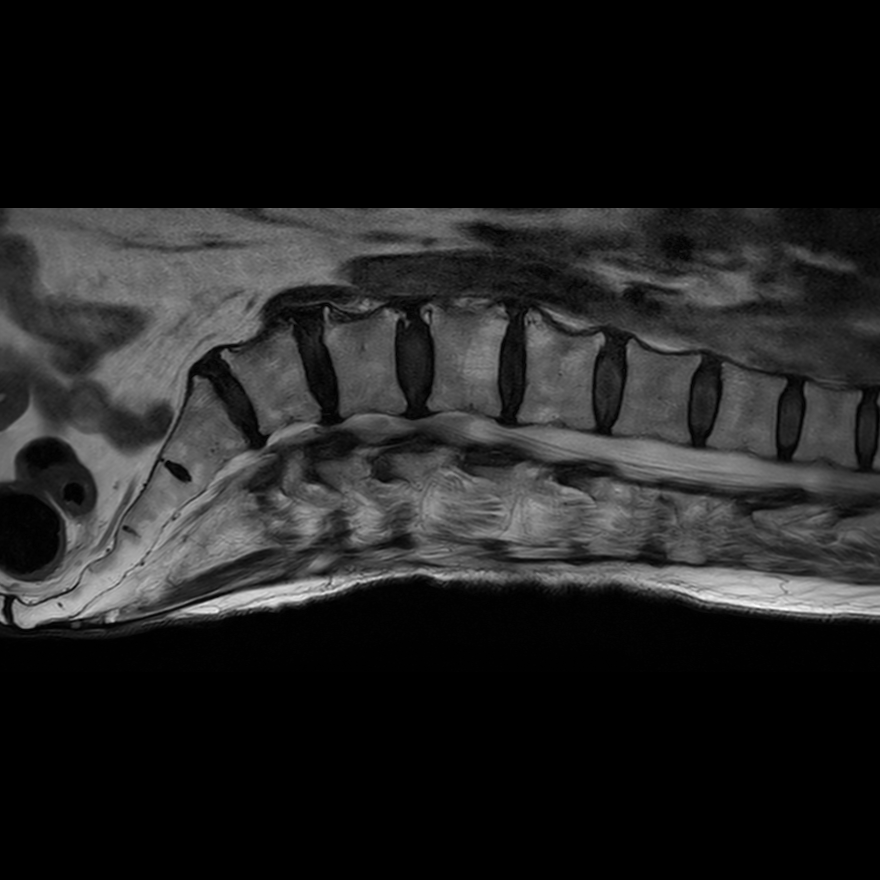

Supplement: S1 File — (ZIP) [file pone.0248303.s001.zip › Code and data/dataset/train/80.png]

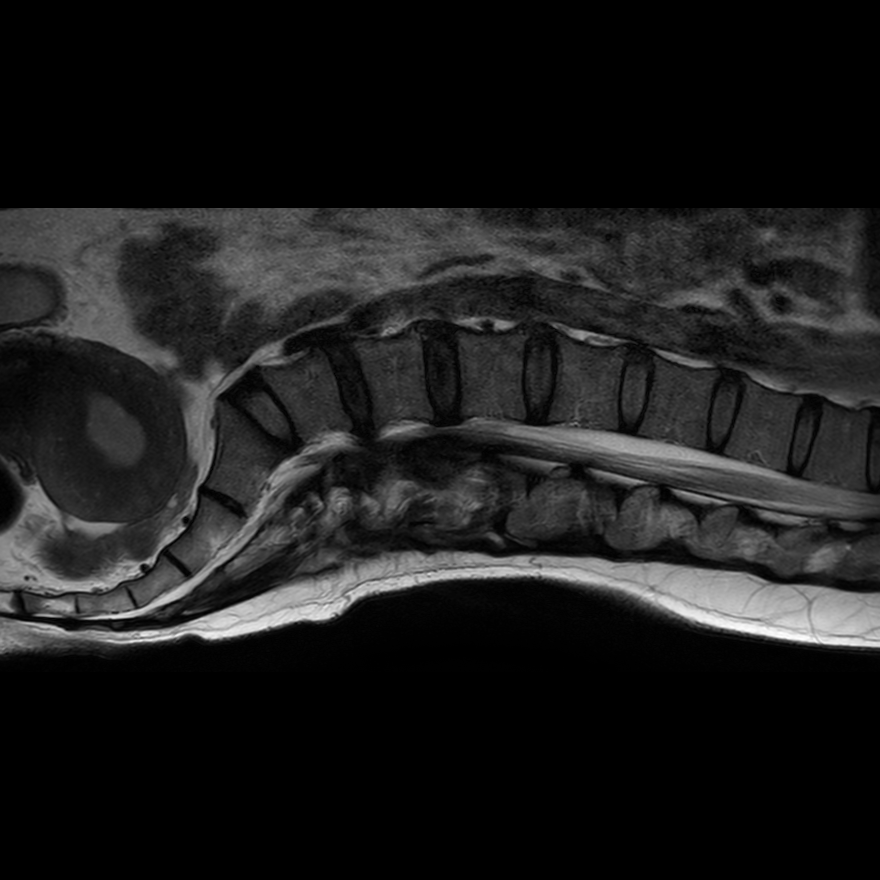

Supplement: S1 File — (ZIP) [file pone.0248303.s001.zip › Code and data/dataset/train/81.png]

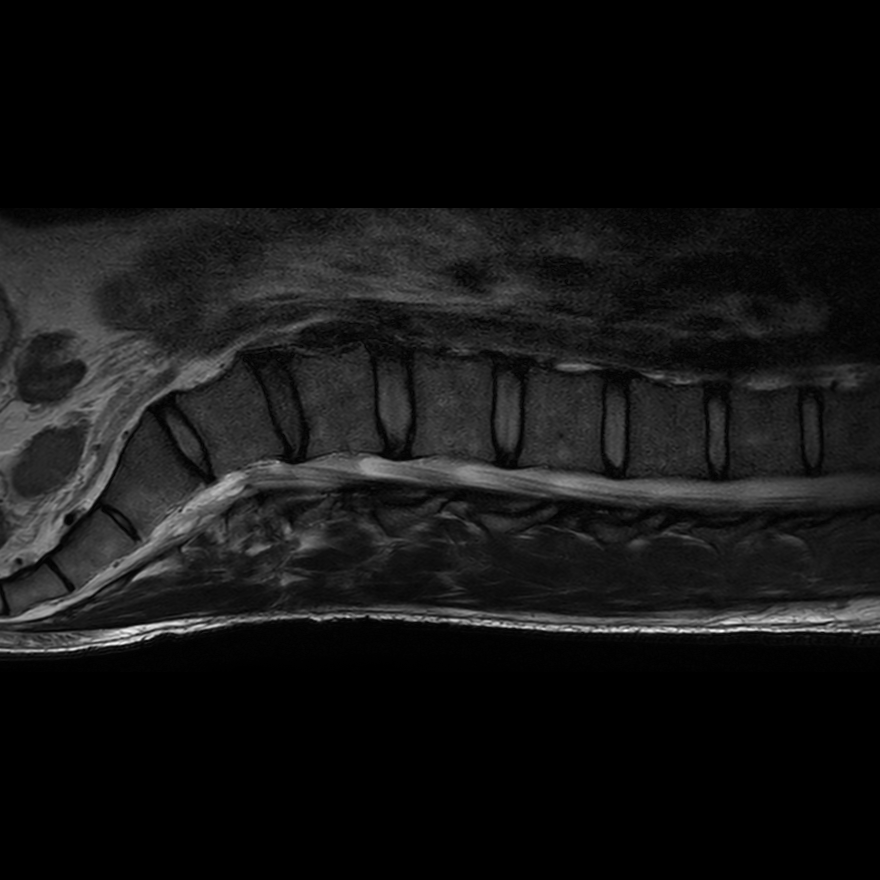

Supplement: S1 File — (ZIP) [file pone.0248303.s001.zip › Code and data/dataset/train/82.png]

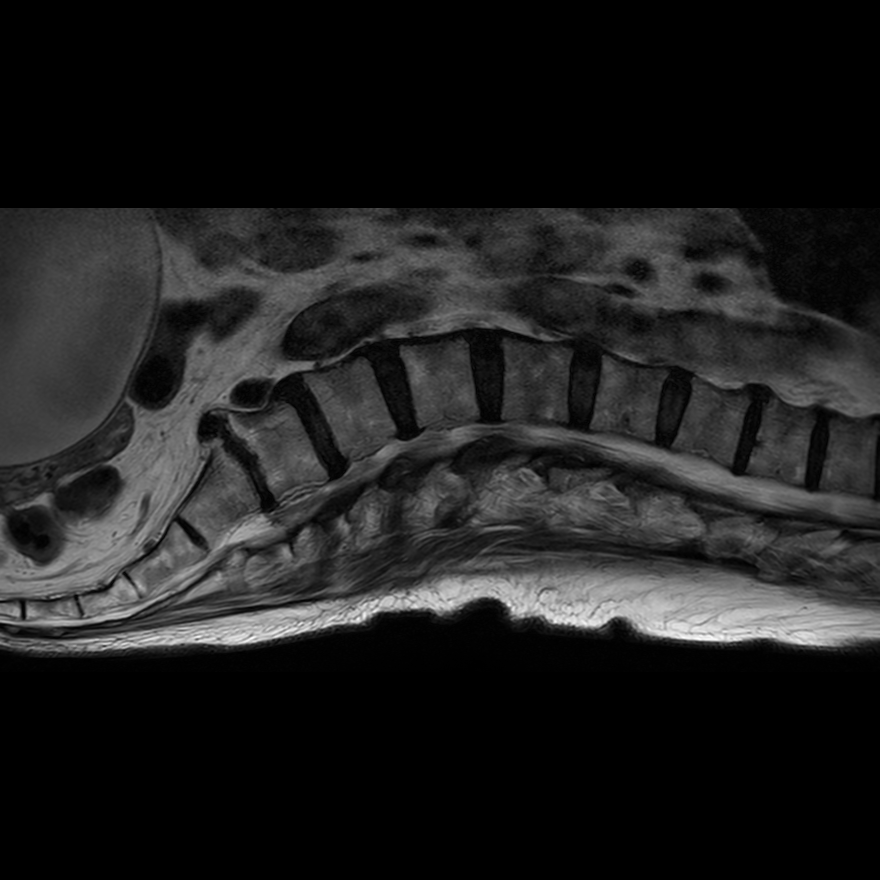

Supplement: S1 File — (ZIP) [file pone.0248303.s001.zip › Code and data/dataset/train/83.png]

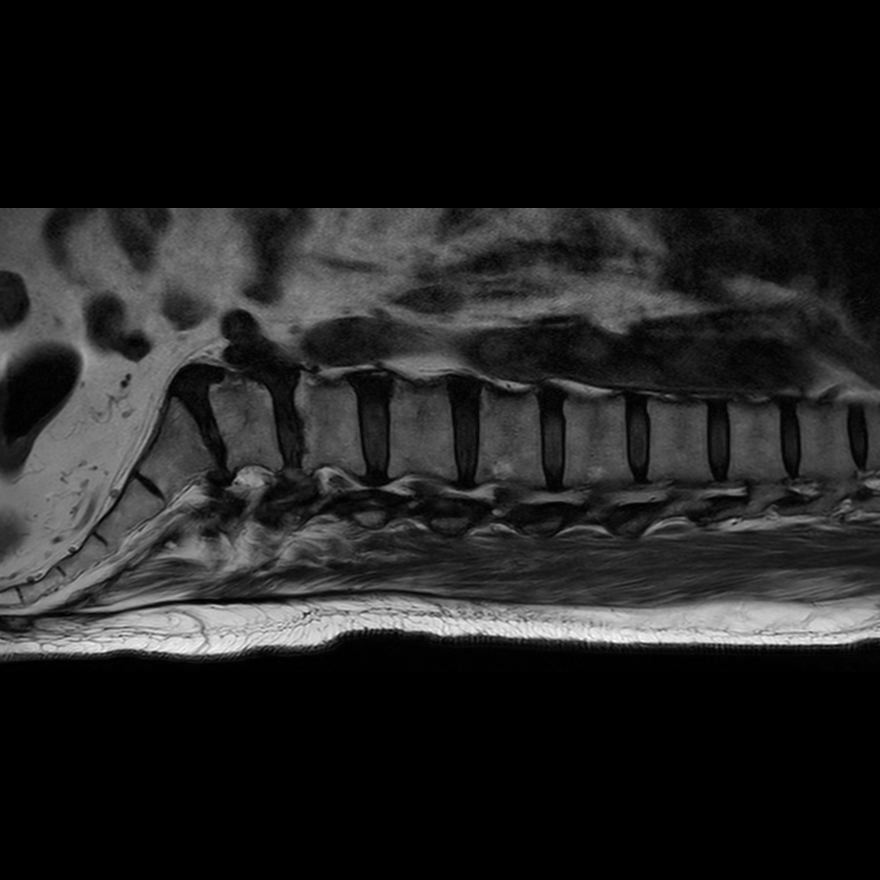

Supplement: S1 File — (ZIP) [file pone.0248303.s001.zip › Code and data/dataset/train/84.png]

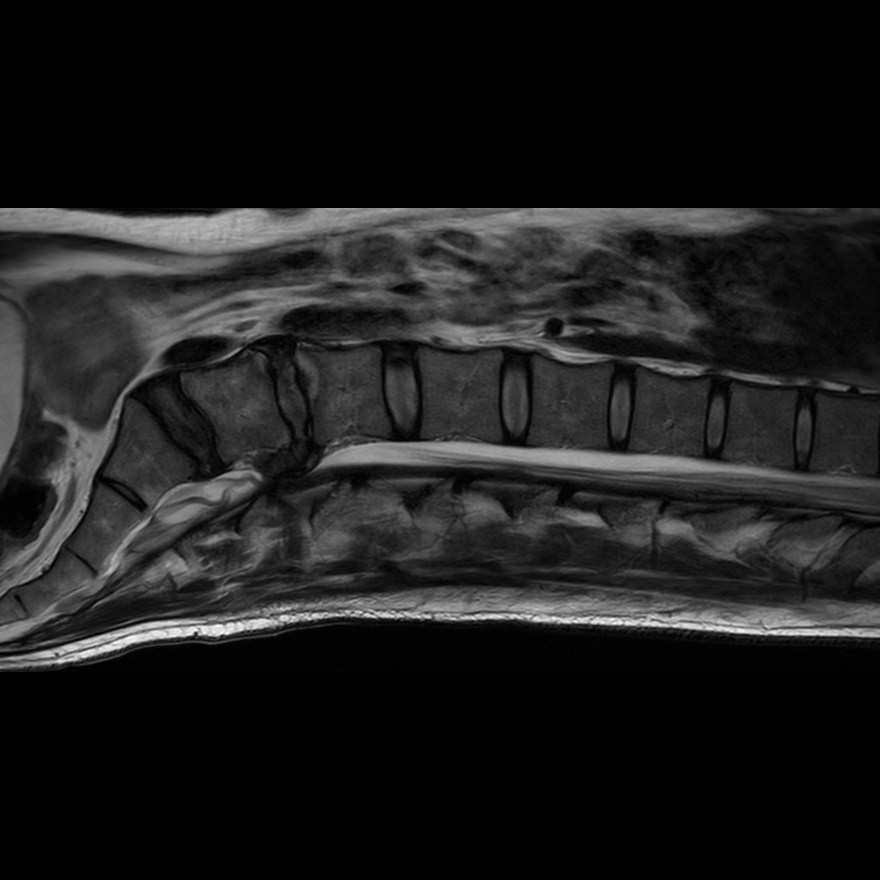

Supplement: S1 File — (ZIP) [file pone.0248303.s001.zip › Code and data/dataset/train/85.png]

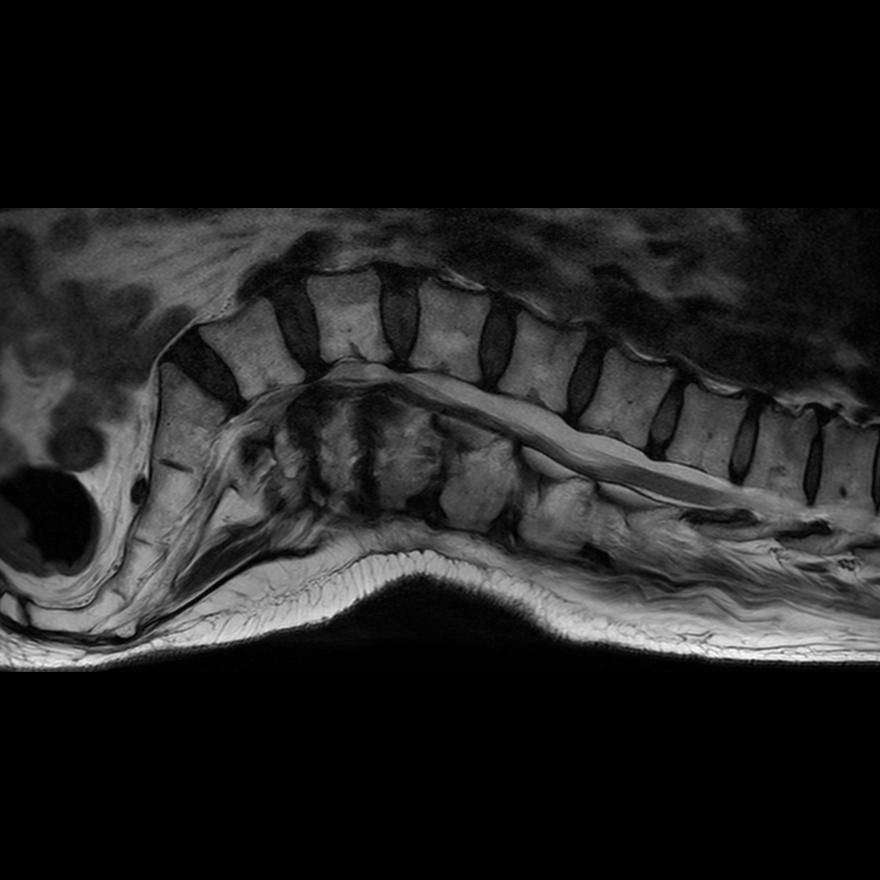

Supplement: S1 File — (ZIP) [file pone.0248303.s001.zip › Code and data/dataset/train/86.png]

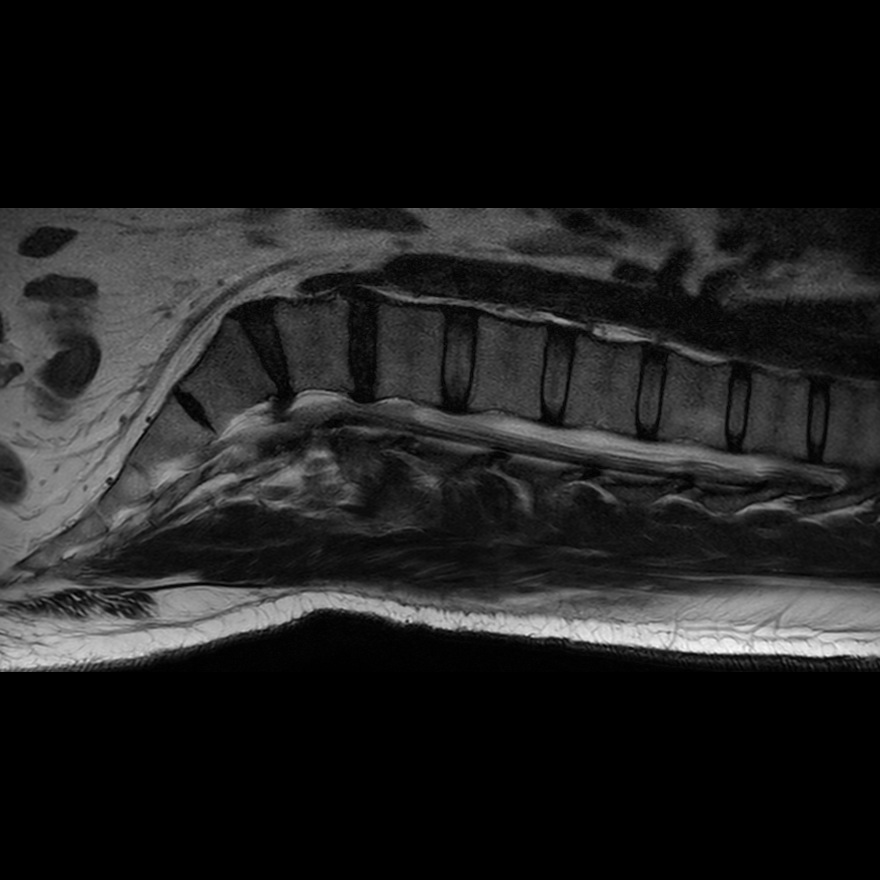

Supplement: S1 File — (ZIP) [file pone.0248303.s001.zip › Code and data/dataset/train/87.png]

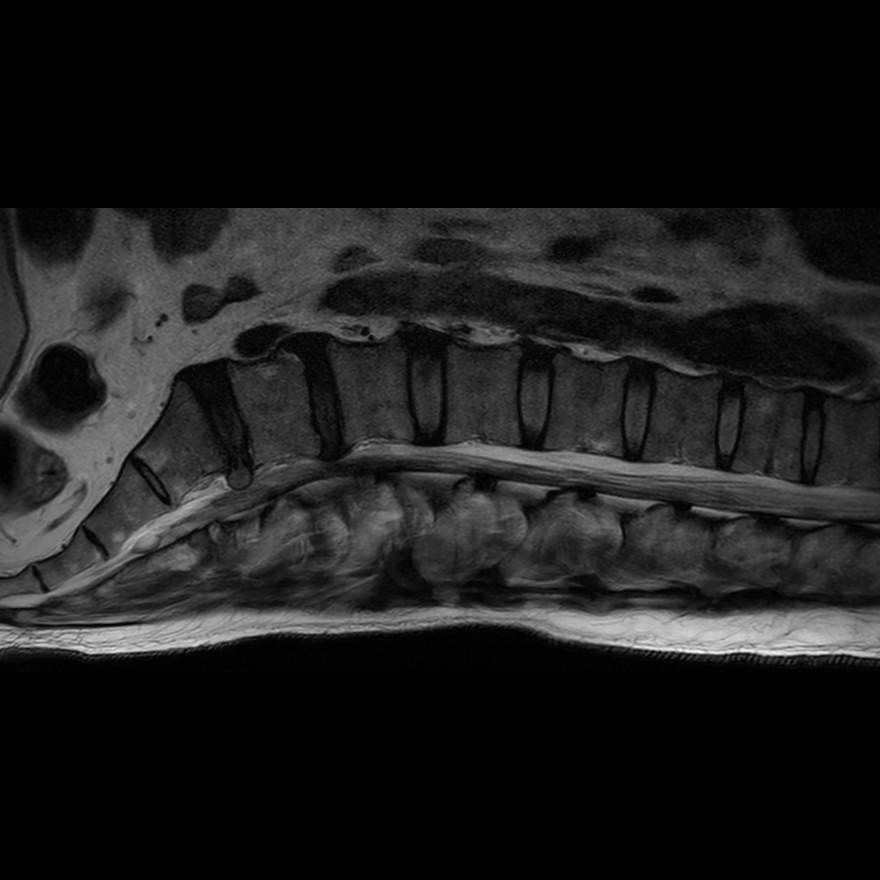

Supplement: S1 File — (ZIP) [file pone.0248303.s001.zip › Code and data/dataset/train/88.png]

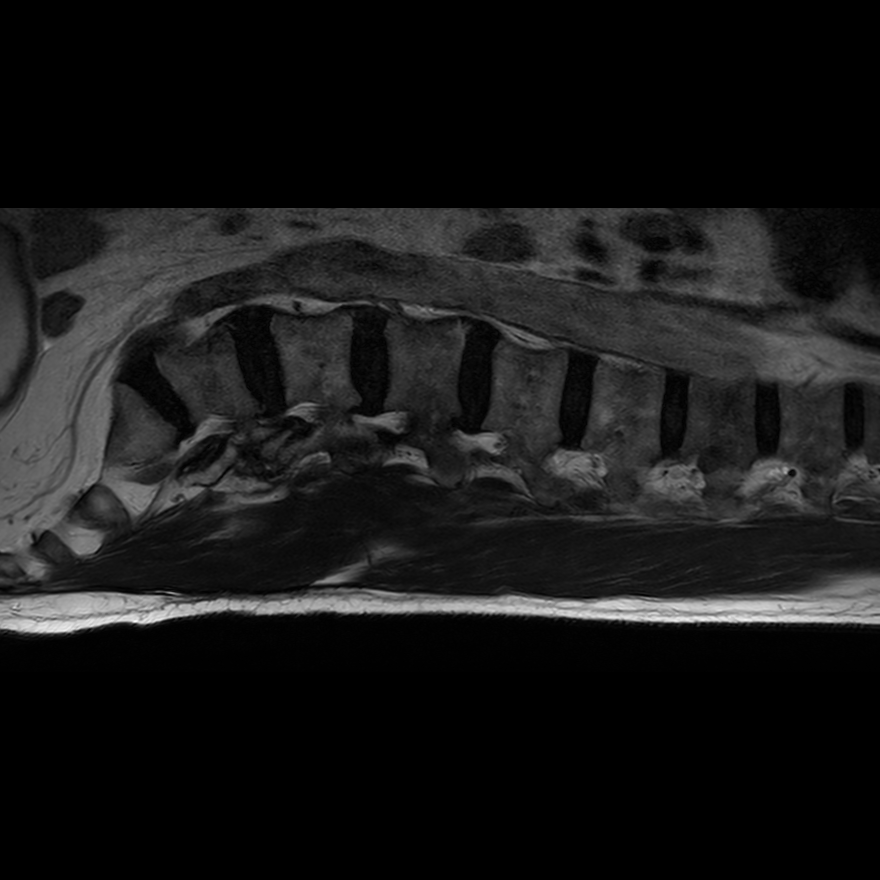

Supplement: S1 File — (ZIP) [file pone.0248303.s001.zip › Code and data/dataset/train/89.png]

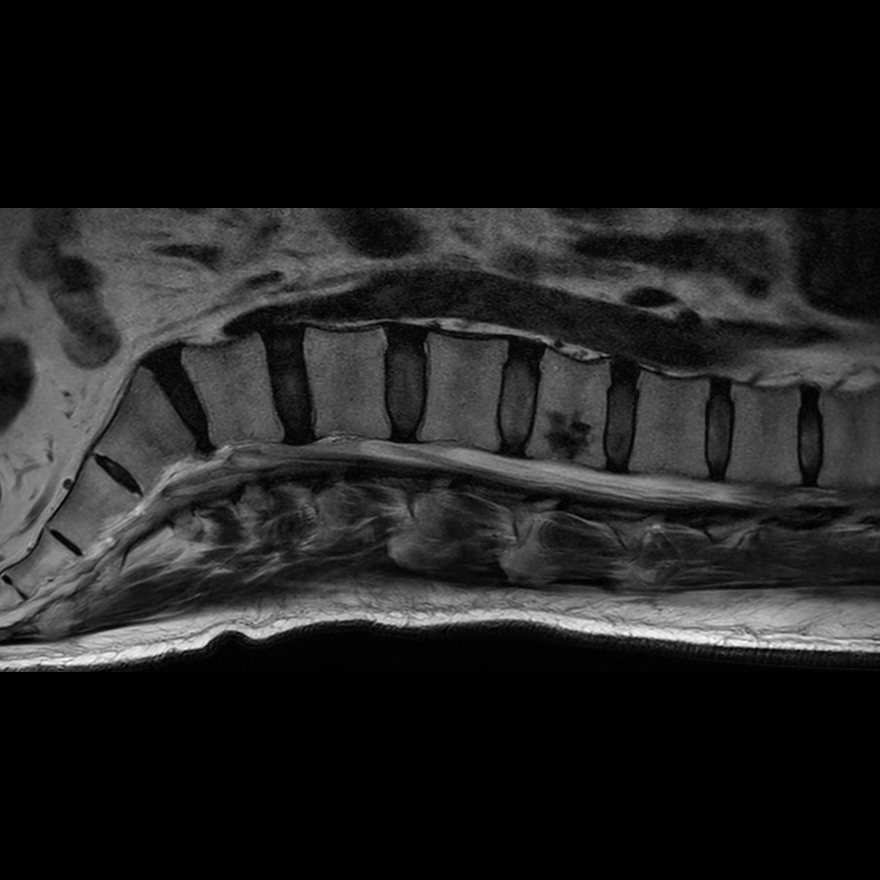

Supplement: S1 File — (ZIP) [file pone.0248303.s001.zip › Code and data/dataset/train/9.png]

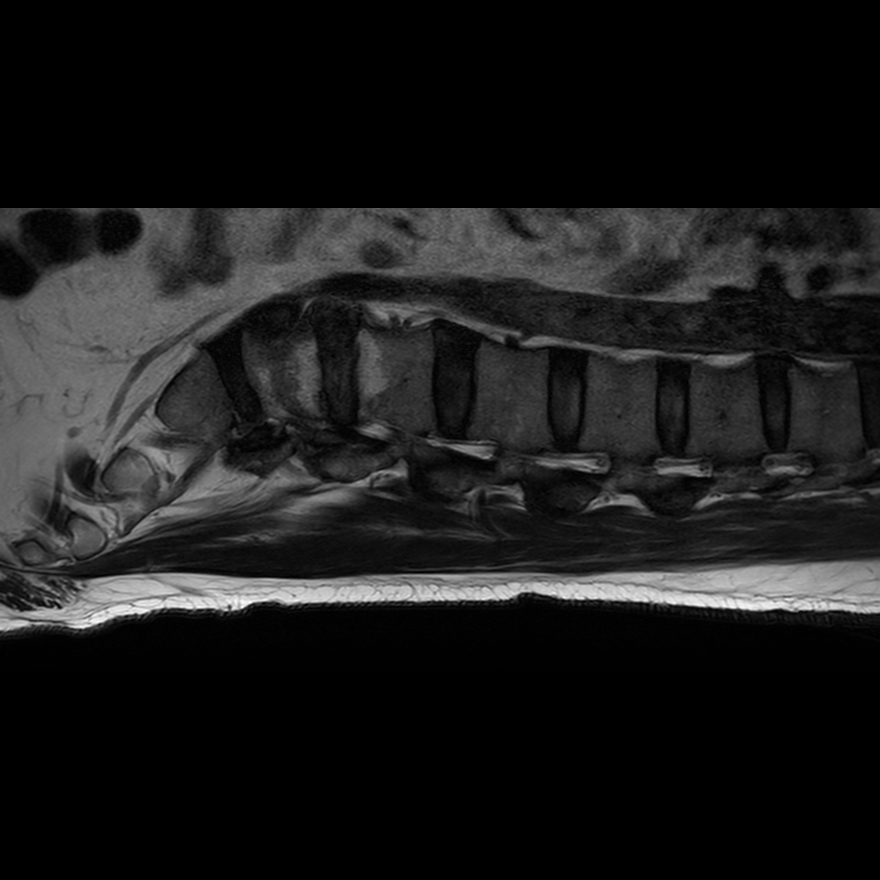

Supplement: S1 File — (ZIP) [file pone.0248303.s001.zip › Code and data/dataset/train/90.png]

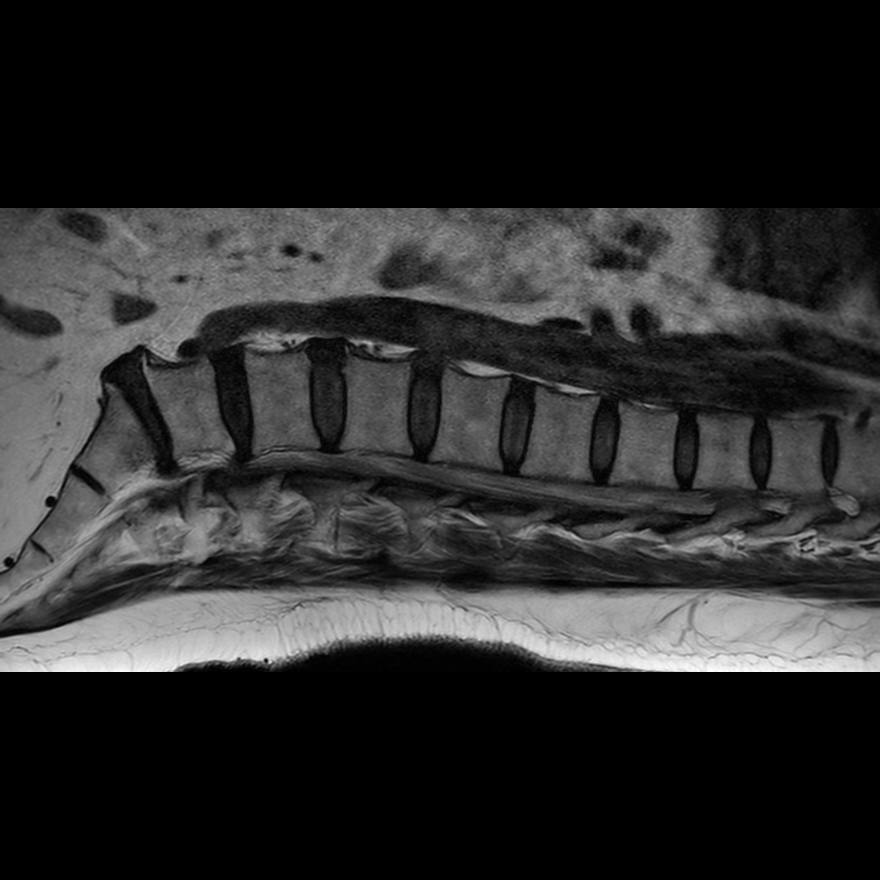

Supplement: S1 File — (ZIP) [file pone.0248303.s001.zip › Code and data/dataset/train/91.png]

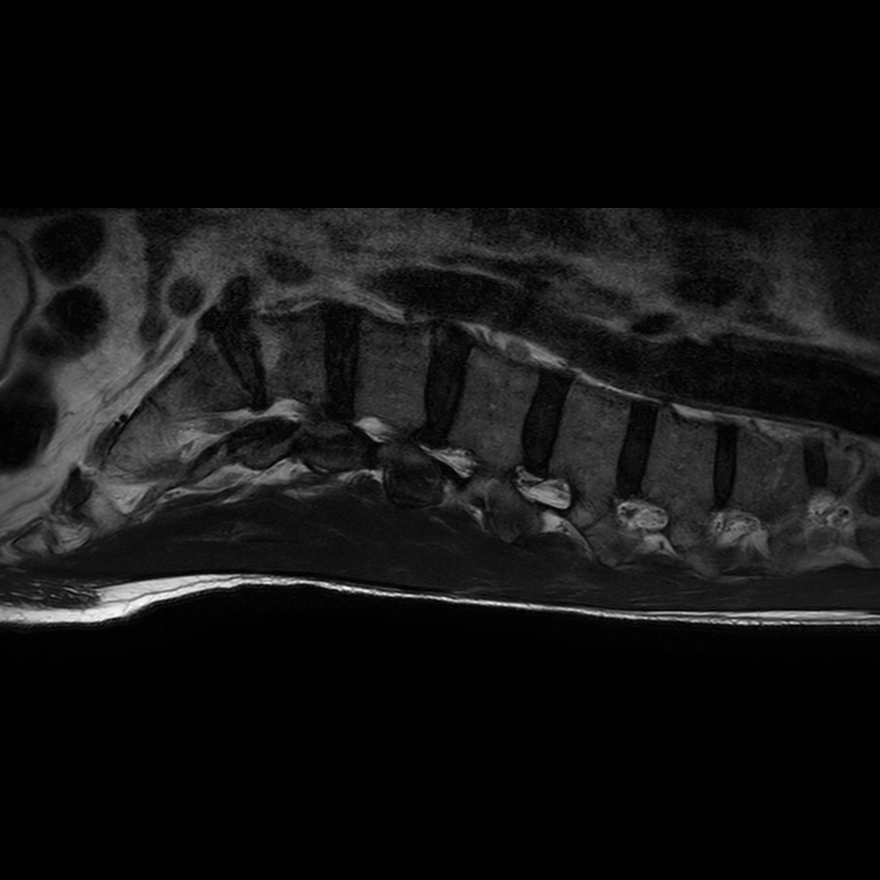

Supplement: S1 File — (ZIP) [file pone.0248303.s001.zip › Code and data/dataset/train/92.png]

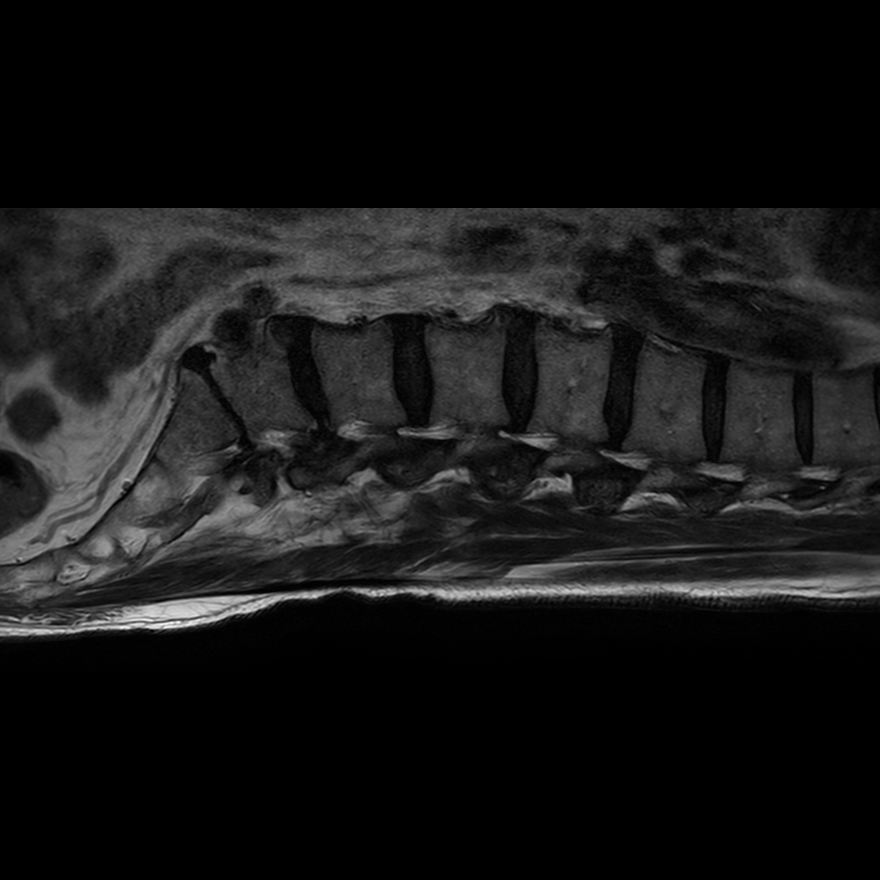

Supplement: S1 File — (ZIP) [file pone.0248303.s001.zip › Code and data/dataset/train/93.png]

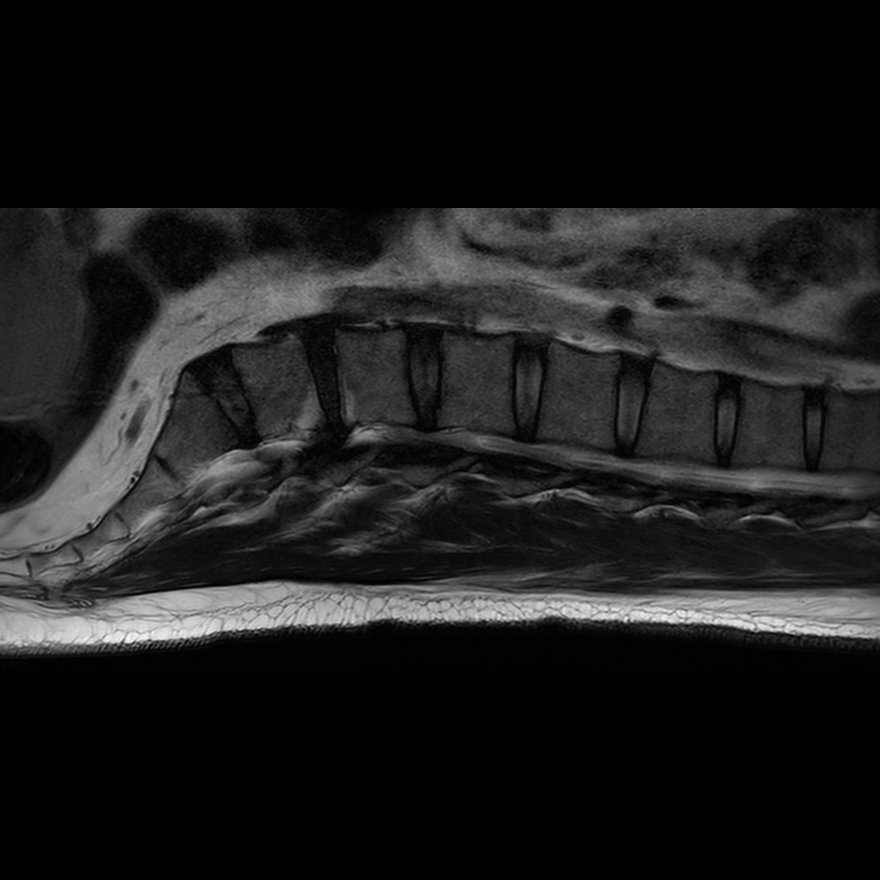

Supplement: S1 File — (ZIP) [file pone.0248303.s001.zip › Code and data/dataset/train/94.png]

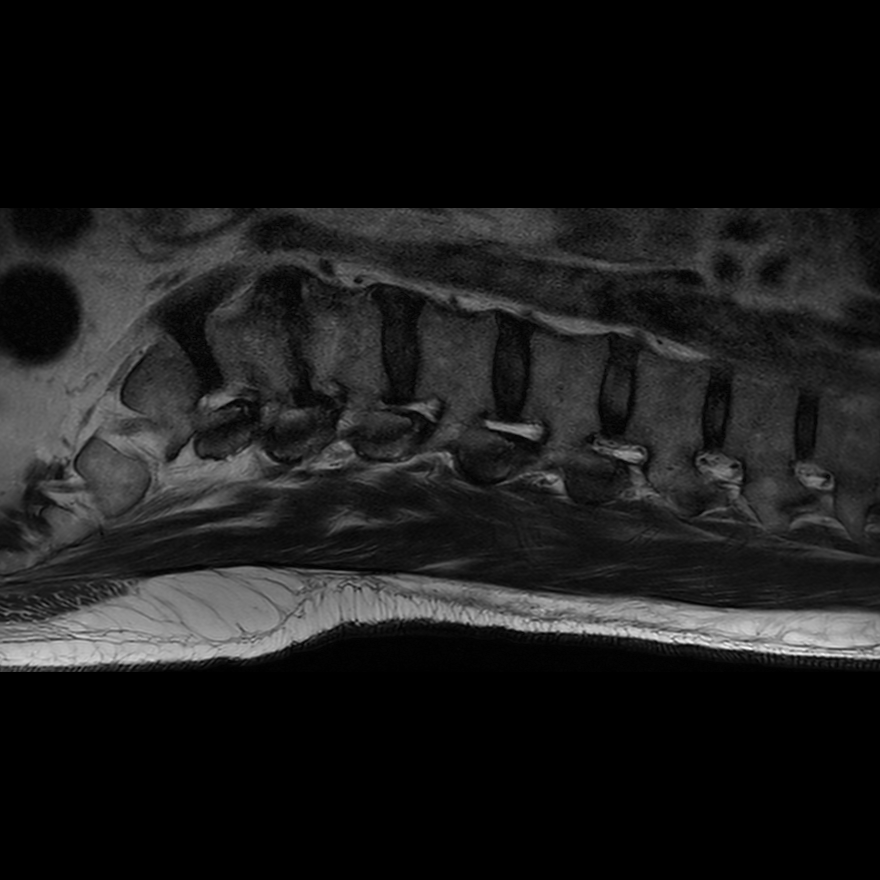

Supplement: S1 File — (ZIP) [file pone.0248303.s001.zip › Code and data/dataset/train/95.png]

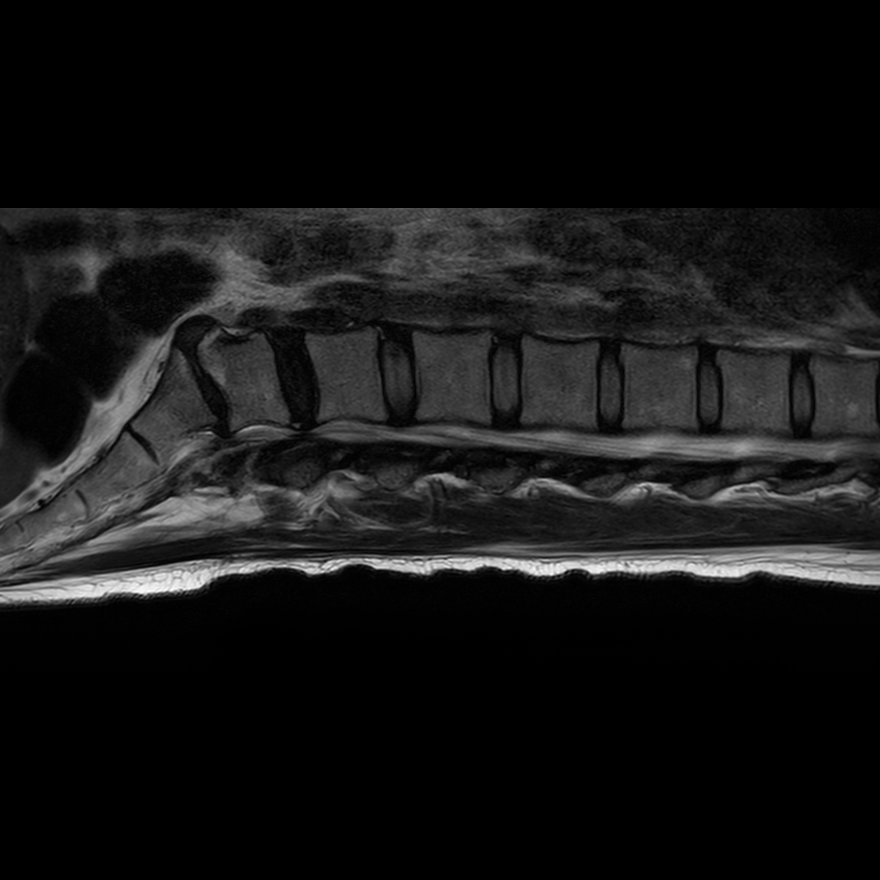

Supplement: S1 File — (ZIP) [file pone.0248303.s001.zip › Code and data/dataset/train/96.png]

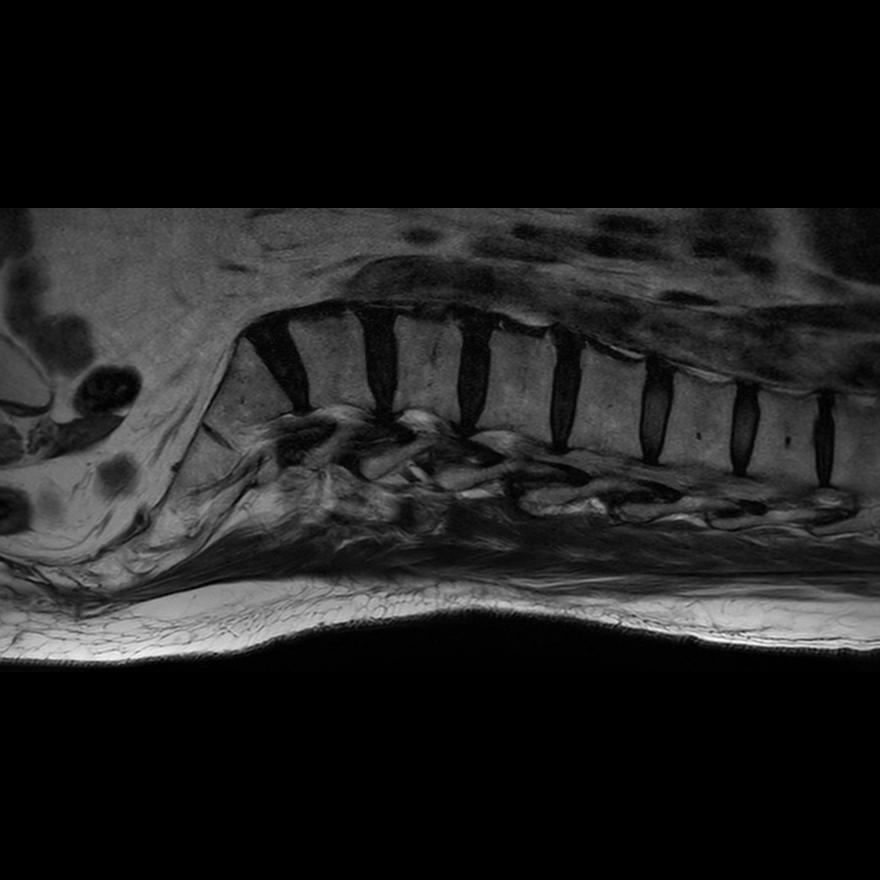

Supplement: S1 File — (ZIP) [file pone.0248303.s001.zip › Code and data/dataset/train/97.png]
