# Supplementary figures and images for: Research on multi-path dense networks for MRI spinal segmentation (part 1 of 2)
Source: PLoS One. 2021 Mar 12;16(3):e0248303. doi: 10.1371/journal.pone.0248303 (PMC7954354; doi:10.1371/journal.pone.0248303)

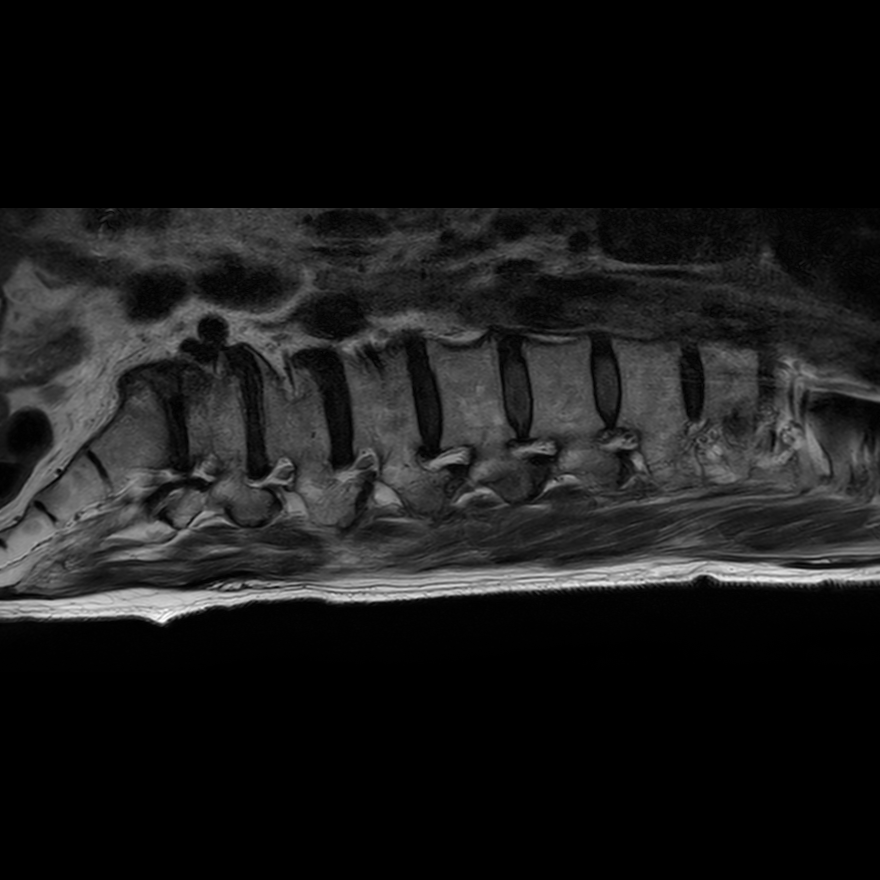

Supplement: S1 File — (ZIP) [file pone.0248303.s001.zip › Code and data/dataset/test/180.png]

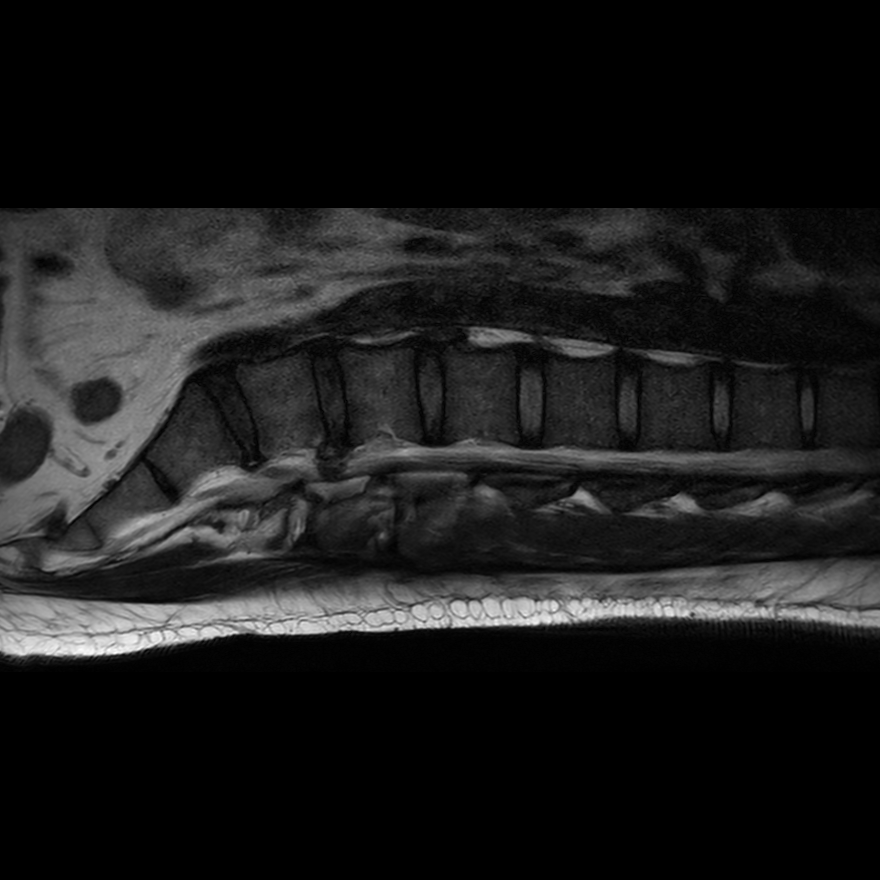

Supplement: S1 File — (ZIP) [file pone.0248303.s001.zip › Code and data/dataset/test/181.png]

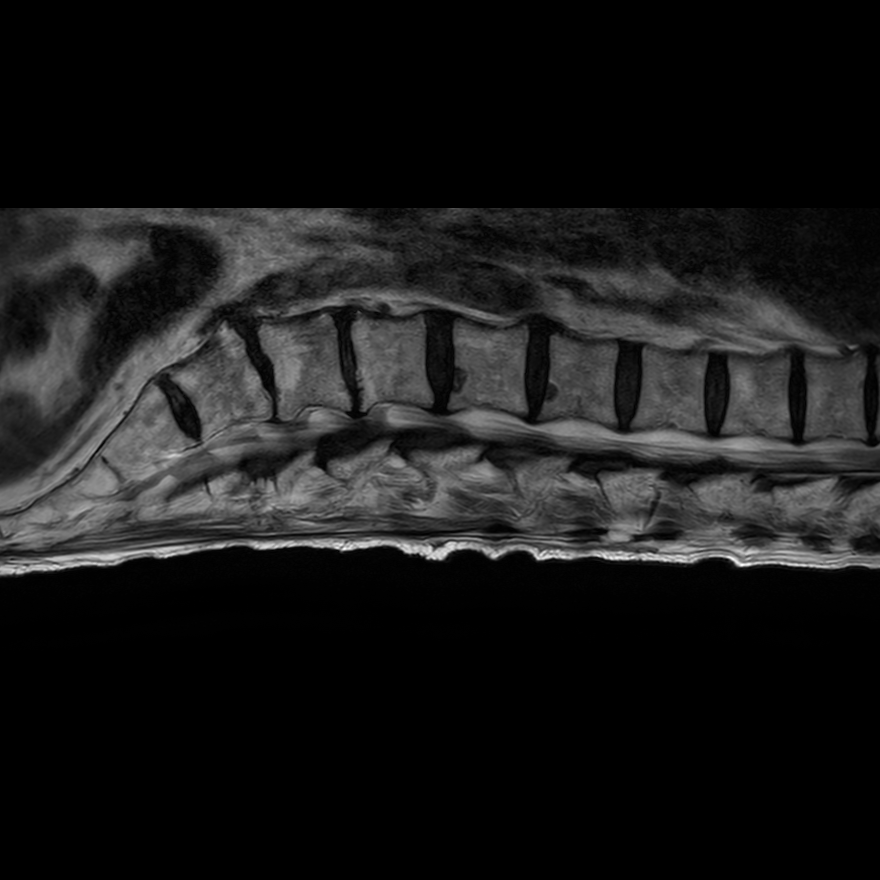

Supplement: S1 File — (ZIP) [file pone.0248303.s001.zip › Code and data/dataset/test/182.png]

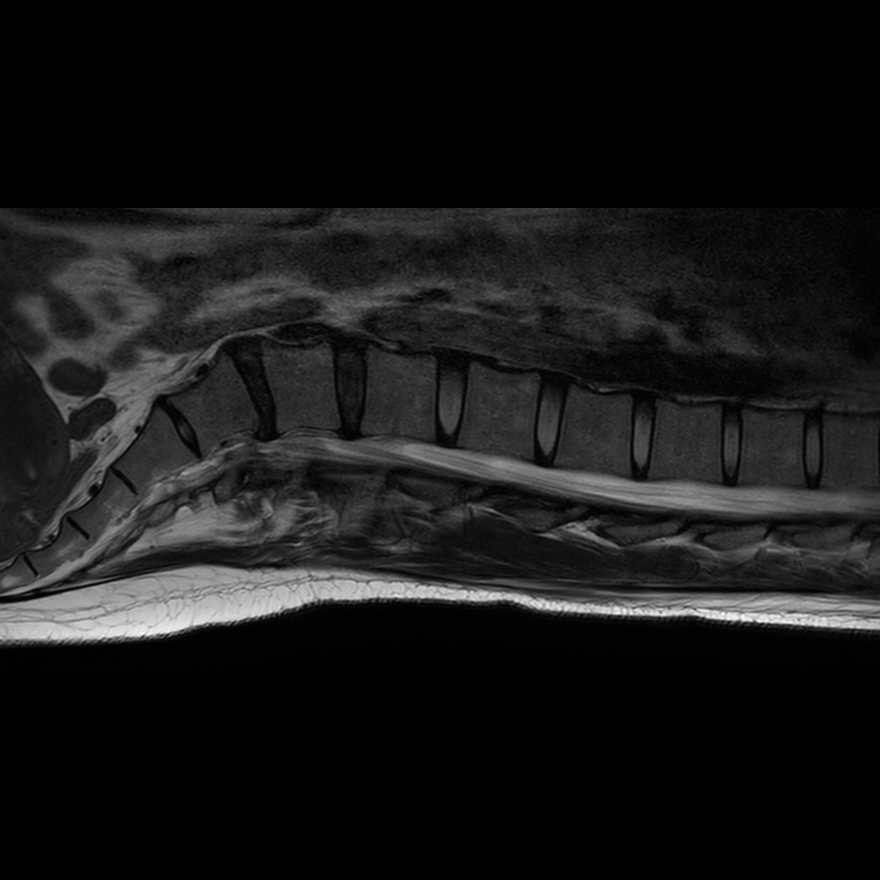

Supplement: S1 File — (ZIP) [file pone.0248303.s001.zip › Code and data/dataset/test/183.png]

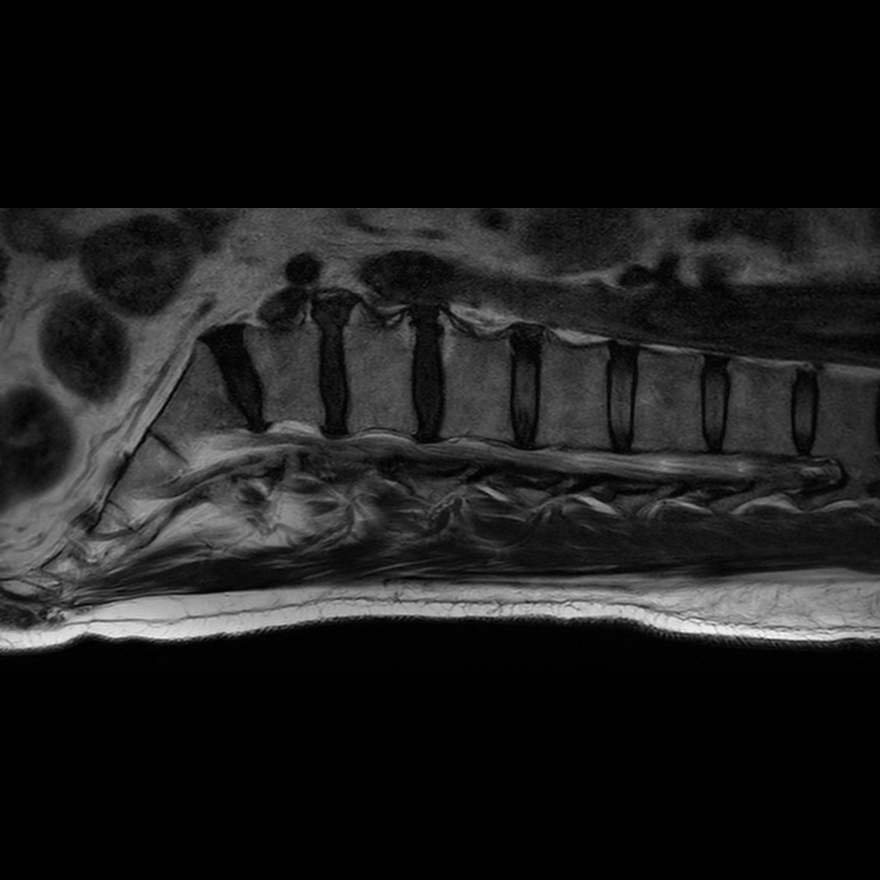

Supplement: S1 File — (ZIP) [file pone.0248303.s001.zip › Code and data/dataset/test/184.png]

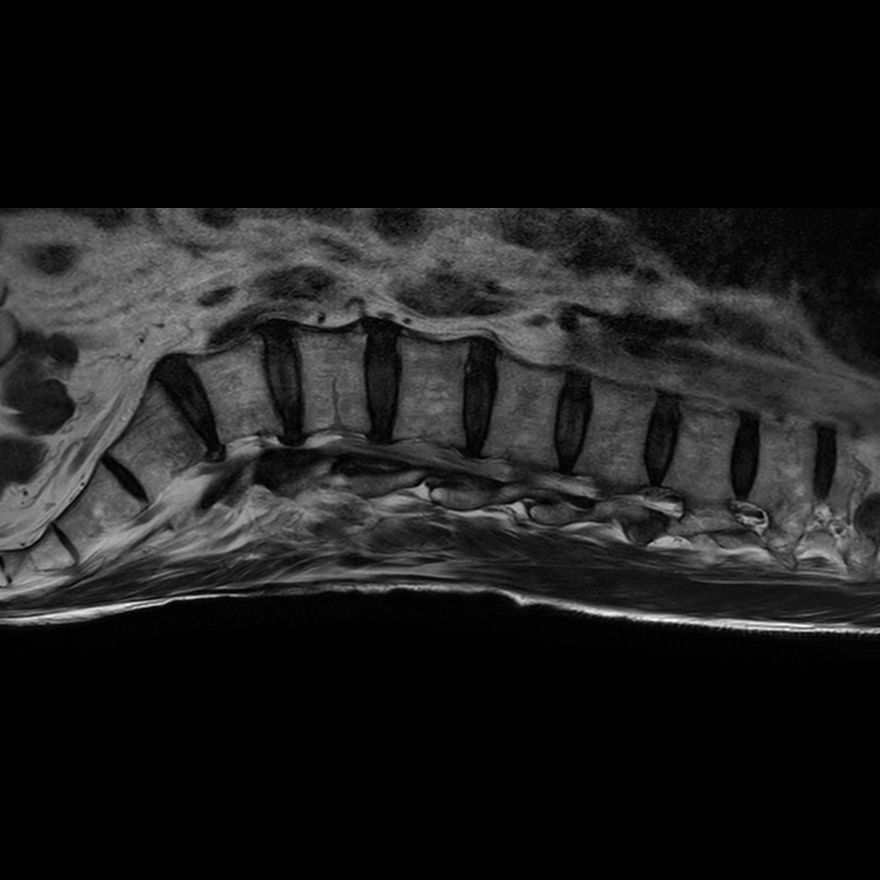

Supplement: S1 File — (ZIP) [file pone.0248303.s001.zip › Code and data/dataset/test/185.png]

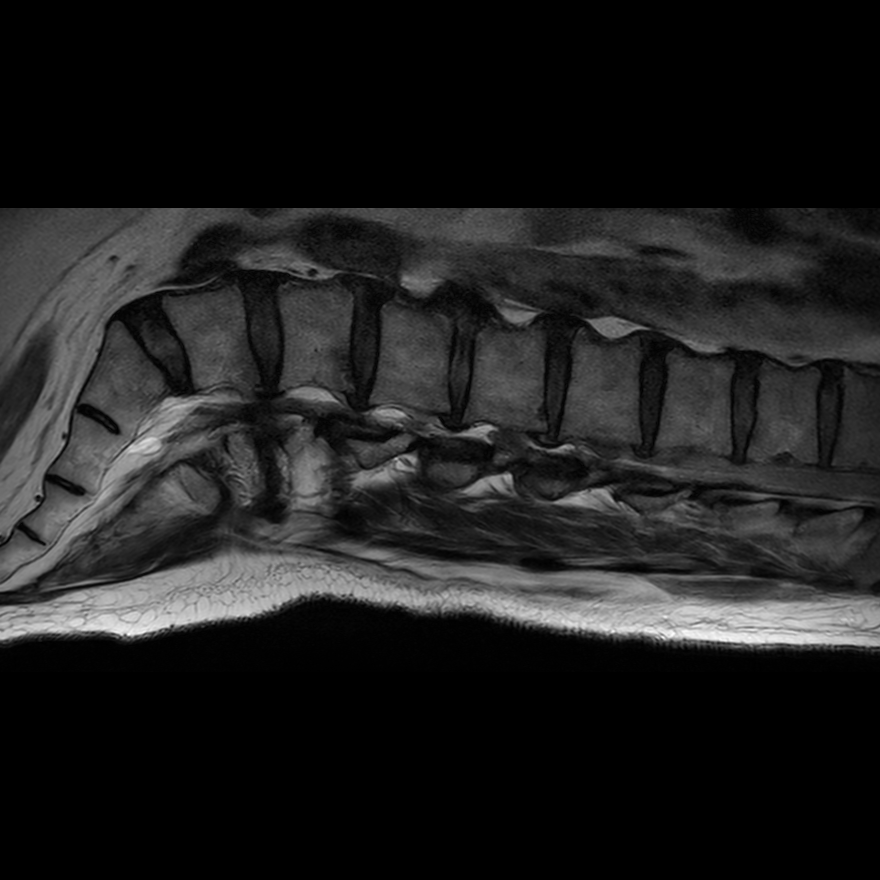

Supplement: S1 File — (ZIP) [file pone.0248303.s001.zip › Code and data/dataset/test/186.png]

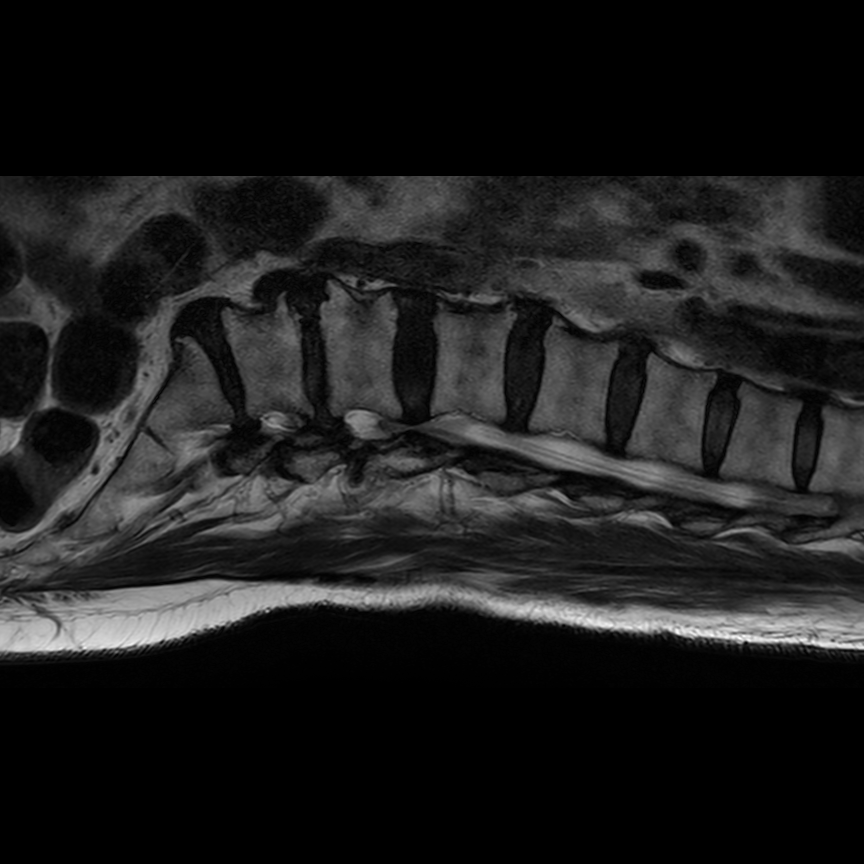

Supplement: S1 File — (ZIP) [file pone.0248303.s001.zip › Code and data/dataset/test/187.png]

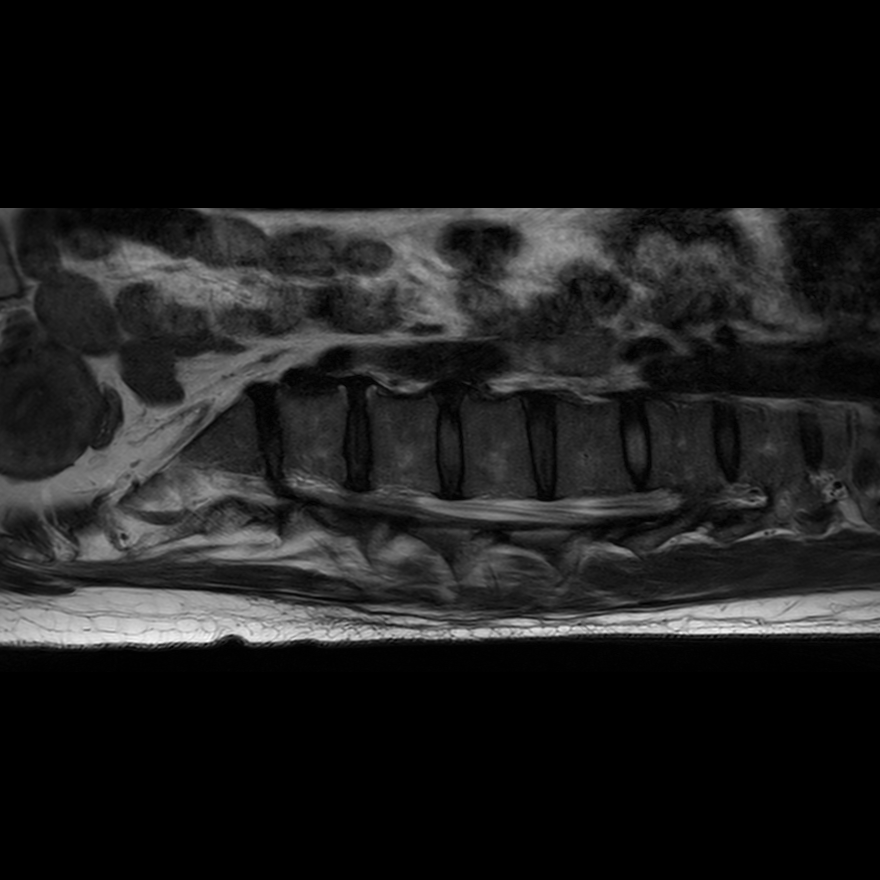

Supplement: S1 File — (ZIP) [file pone.0248303.s001.zip › Code and data/dataset/test/188.png]

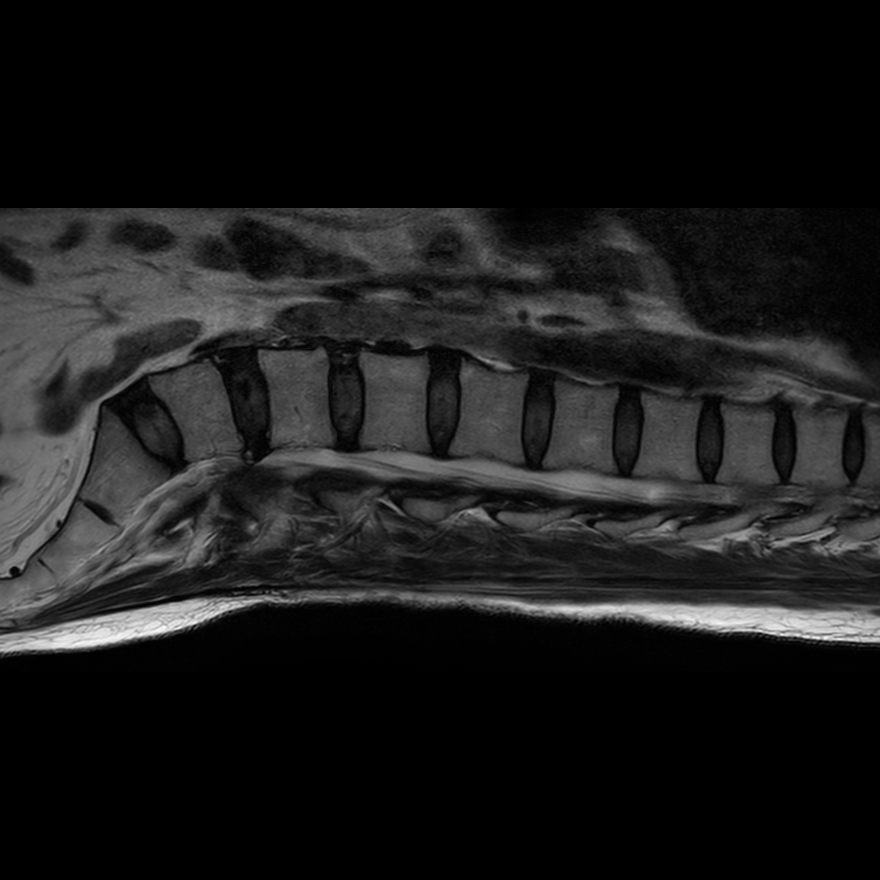

Supplement: S1 File — (ZIP) [file pone.0248303.s001.zip › Code and data/dataset/test/189.png]

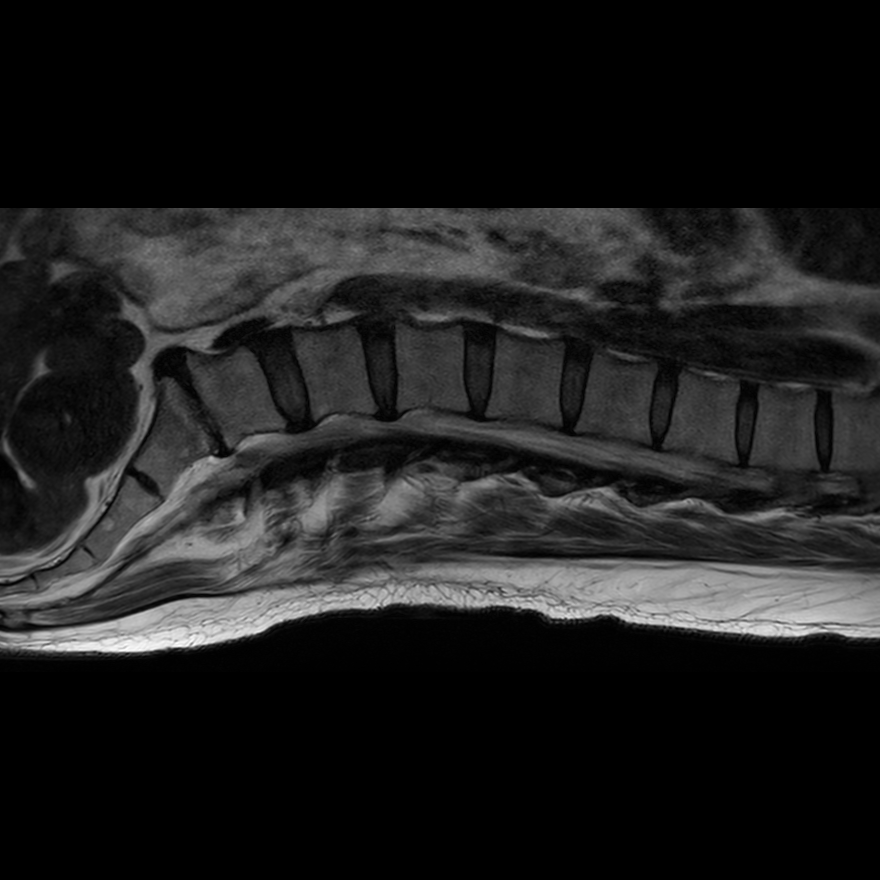

Supplement: S1 File — (ZIP) [file pone.0248303.s001.zip › Code and data/dataset/test/190.png]

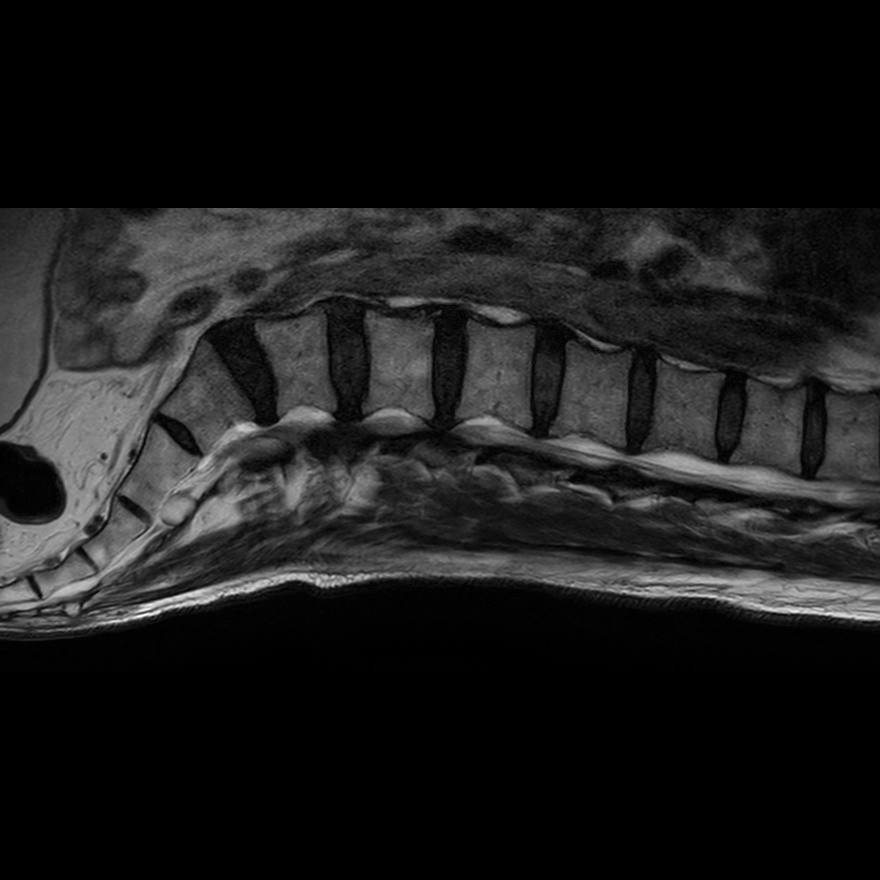

Supplement: S1 File — (ZIP) [file pone.0248303.s001.zip › Code and data/dataset/test/191.png]

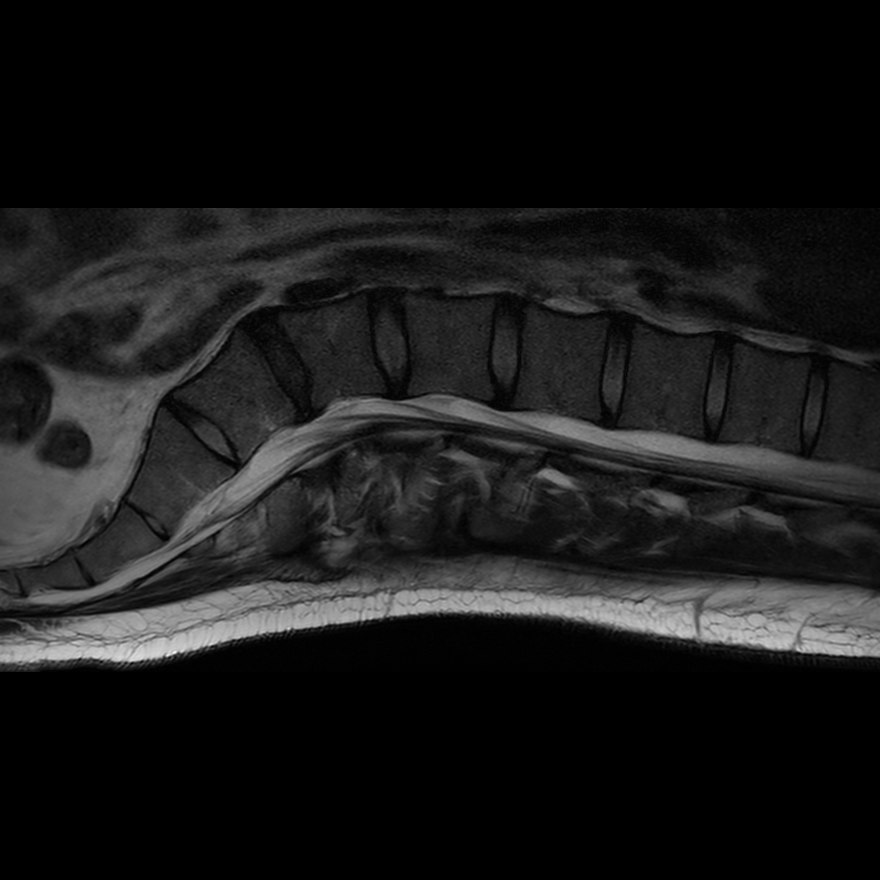

Supplement: S1 File — (ZIP) [file pone.0248303.s001.zip › Code and data/dataset/test/192.png]

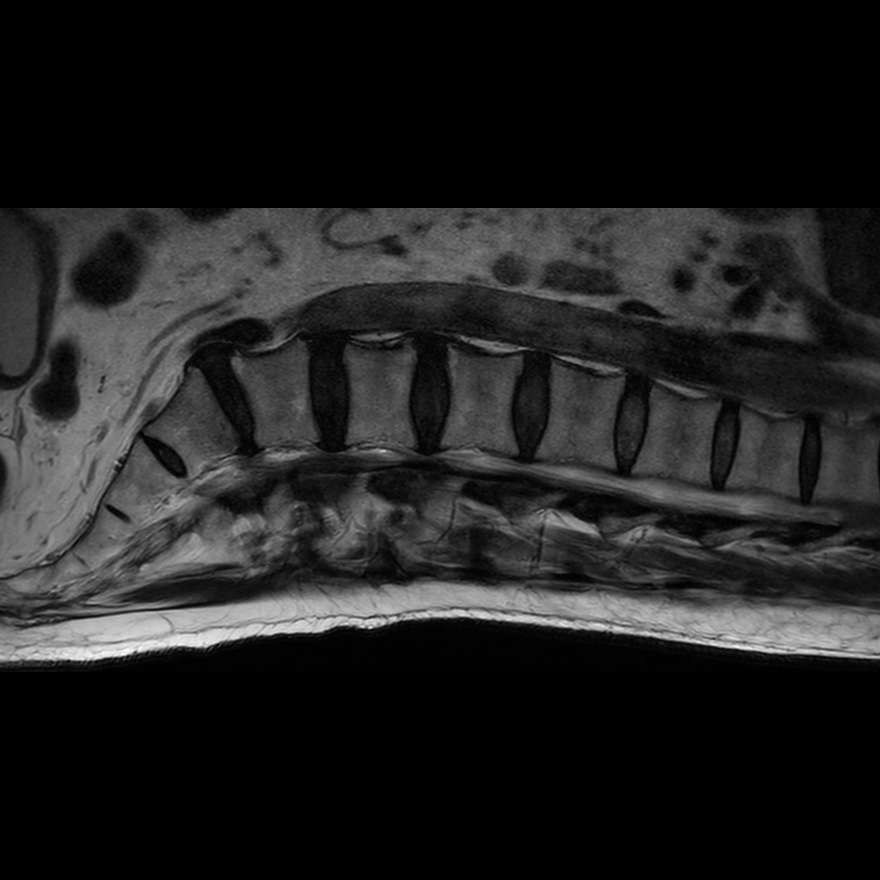

Supplement: S1 File — (ZIP) [file pone.0248303.s001.zip › Code and data/dataset/test/193.png]

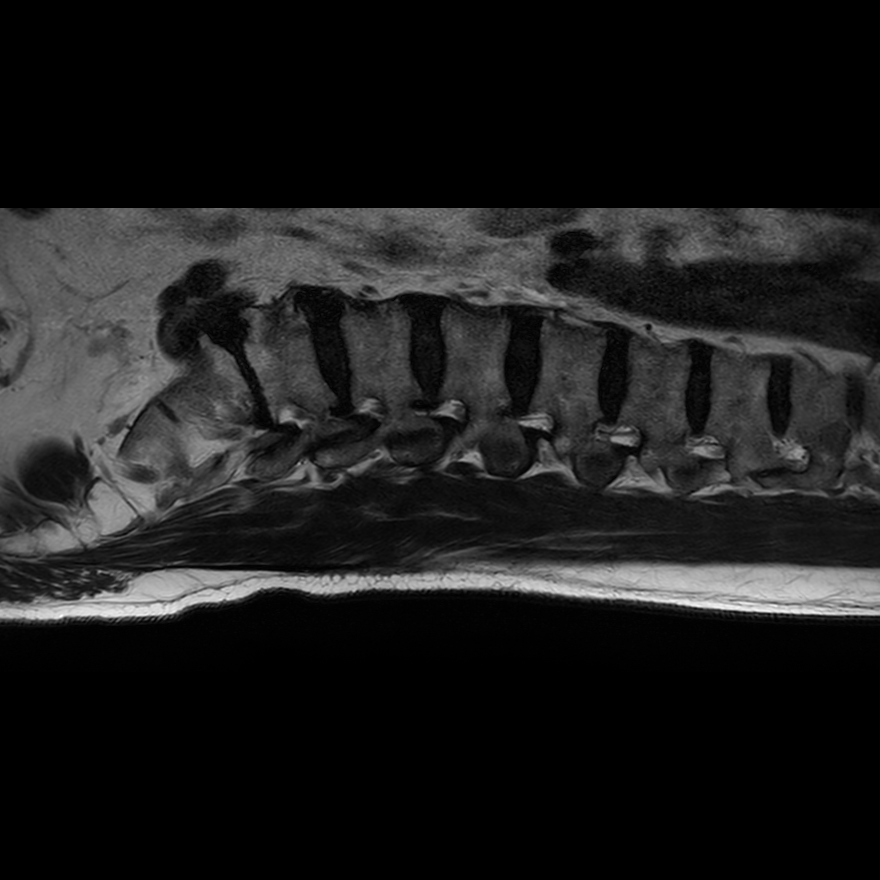

Supplement: S1 File — (ZIP) [file pone.0248303.s001.zip › Code and data/dataset/test/194.png]

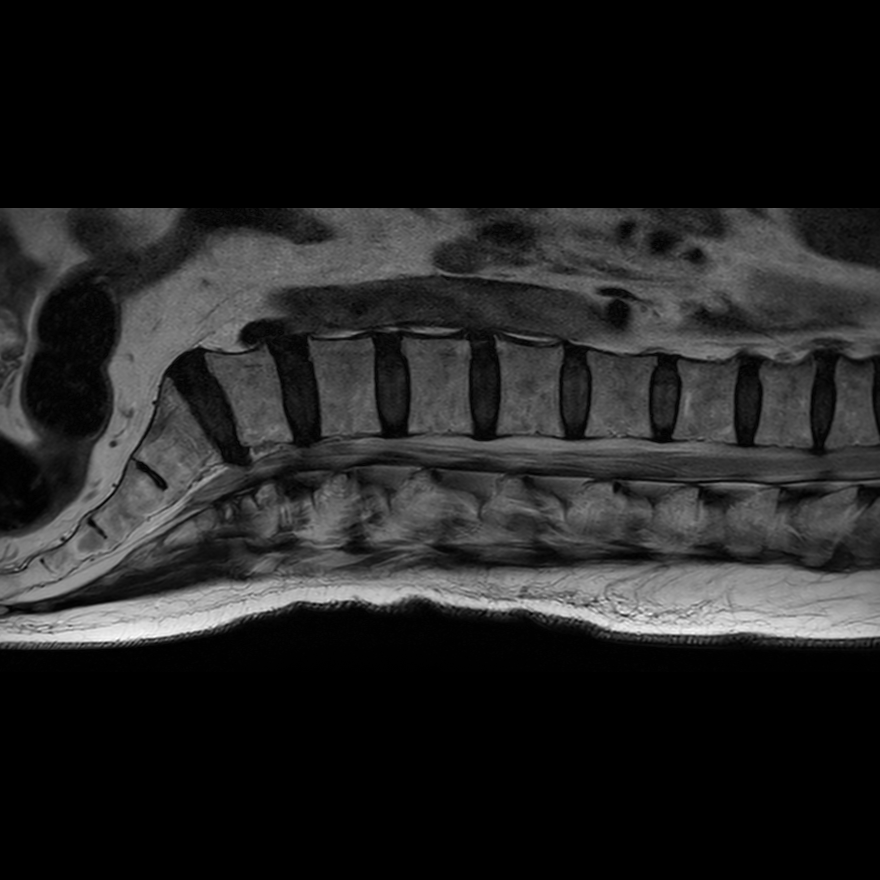

Supplement: S1 File — (ZIP) [file pone.0248303.s001.zip › Code and data/dataset/test/195.png]

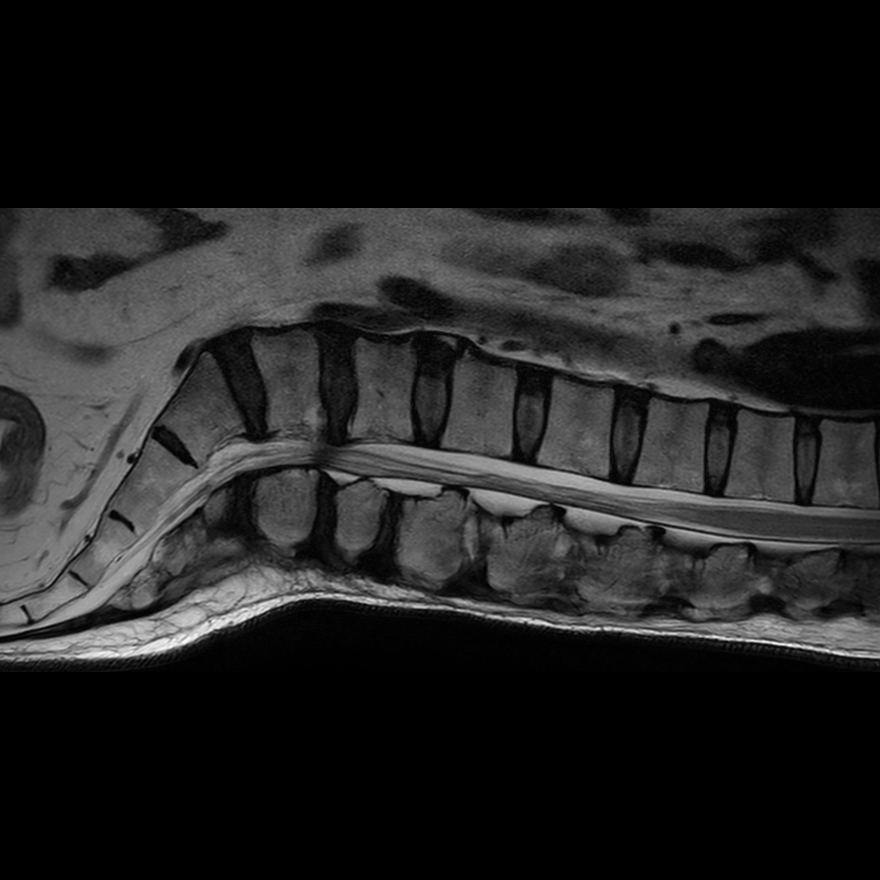

Supplement: S1 File — (ZIP) [file pone.0248303.s001.zip › Code and data/dataset/test/196.png]

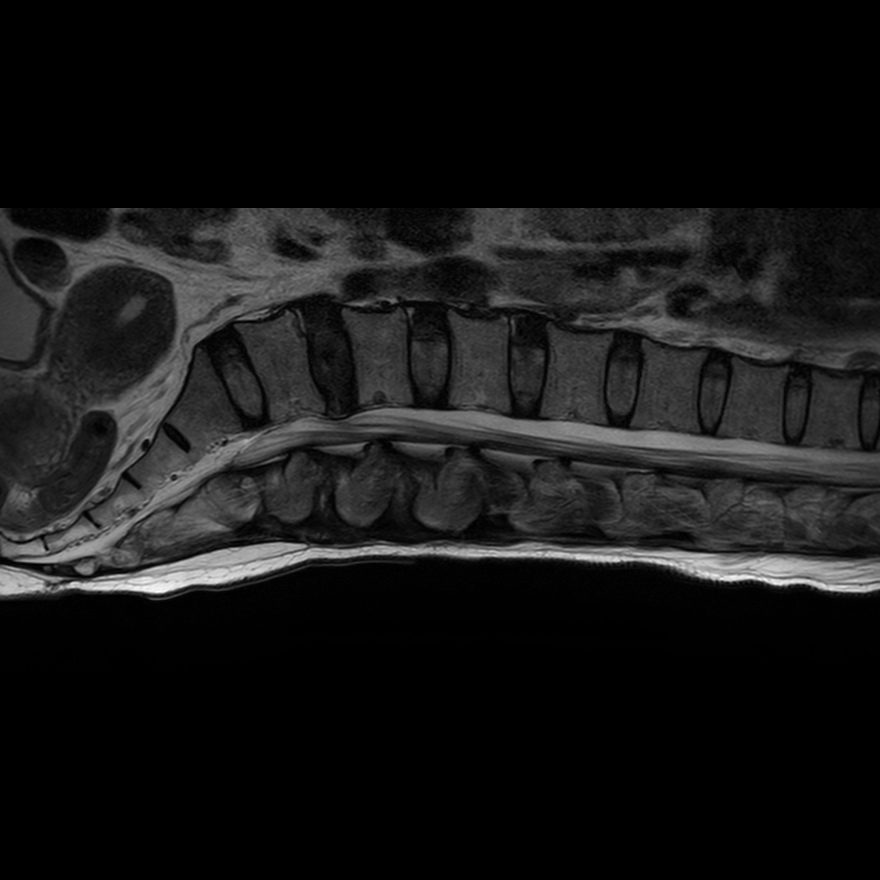

Supplement: S1 File — (ZIP) [file pone.0248303.s001.zip › Code and data/dataset/test/197.png]

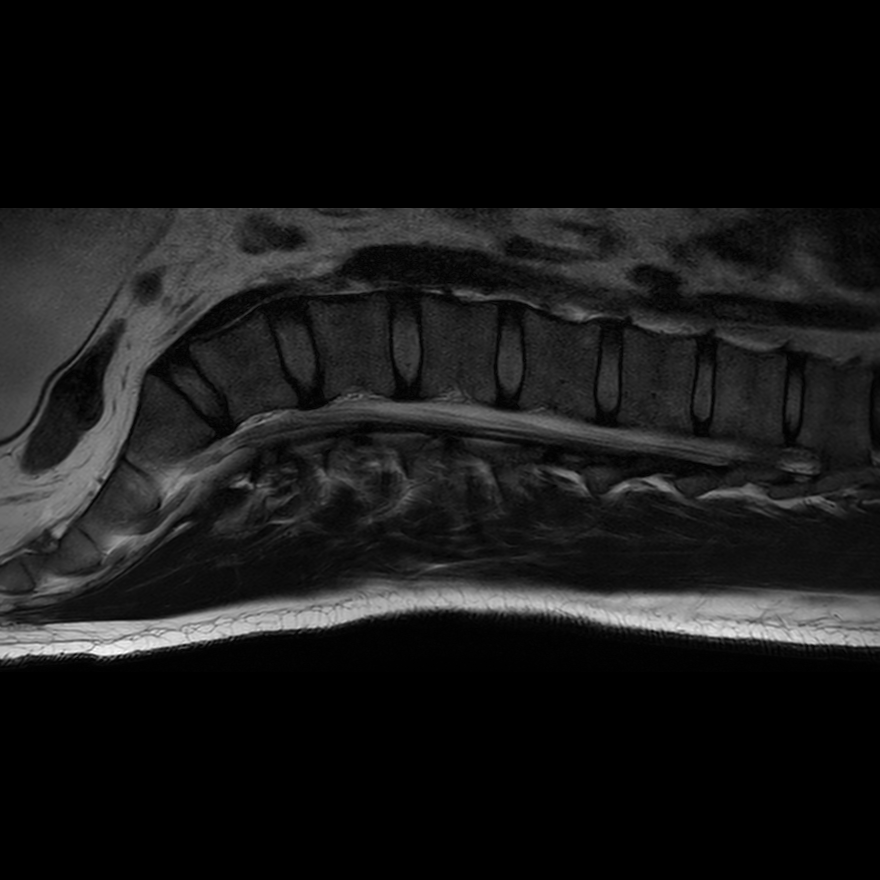

Supplement: S1 File — (ZIP) [file pone.0248303.s001.zip › Code and data/dataset/test/198.png]

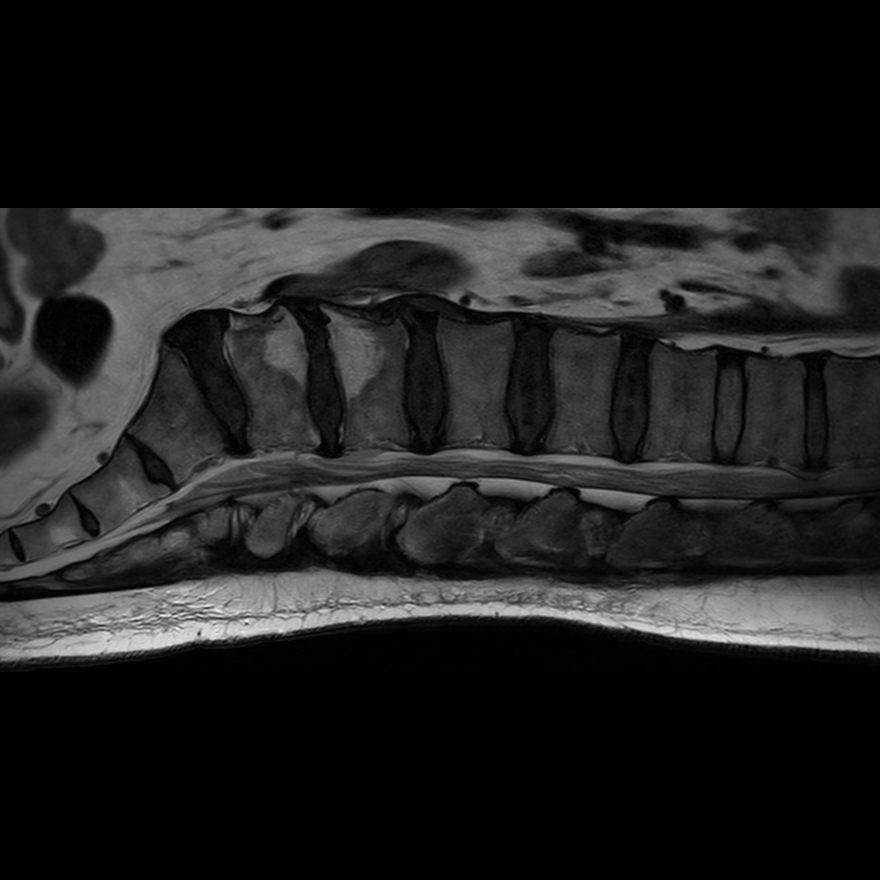

Supplement: S1 File — (ZIP) [file pone.0248303.s001.zip › Code and data/dataset/test/199.png]

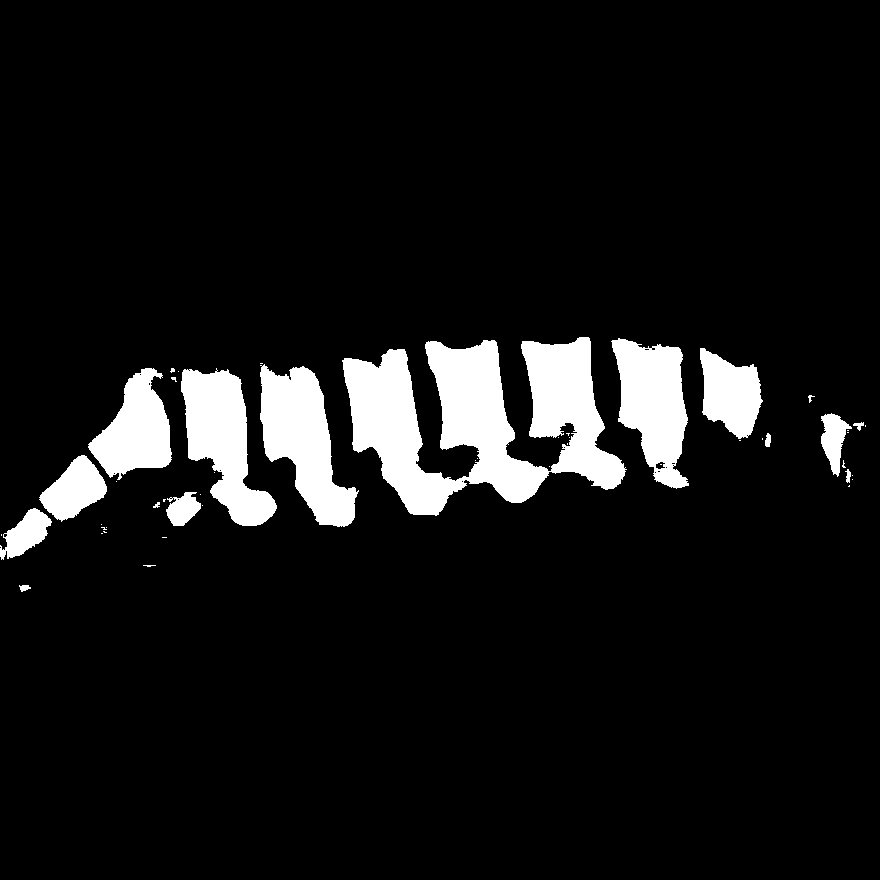

Supplement: S1 File — (ZIP) [file pone.0248303.s001.zip › Code and data/dataset/test_GT/180.png]

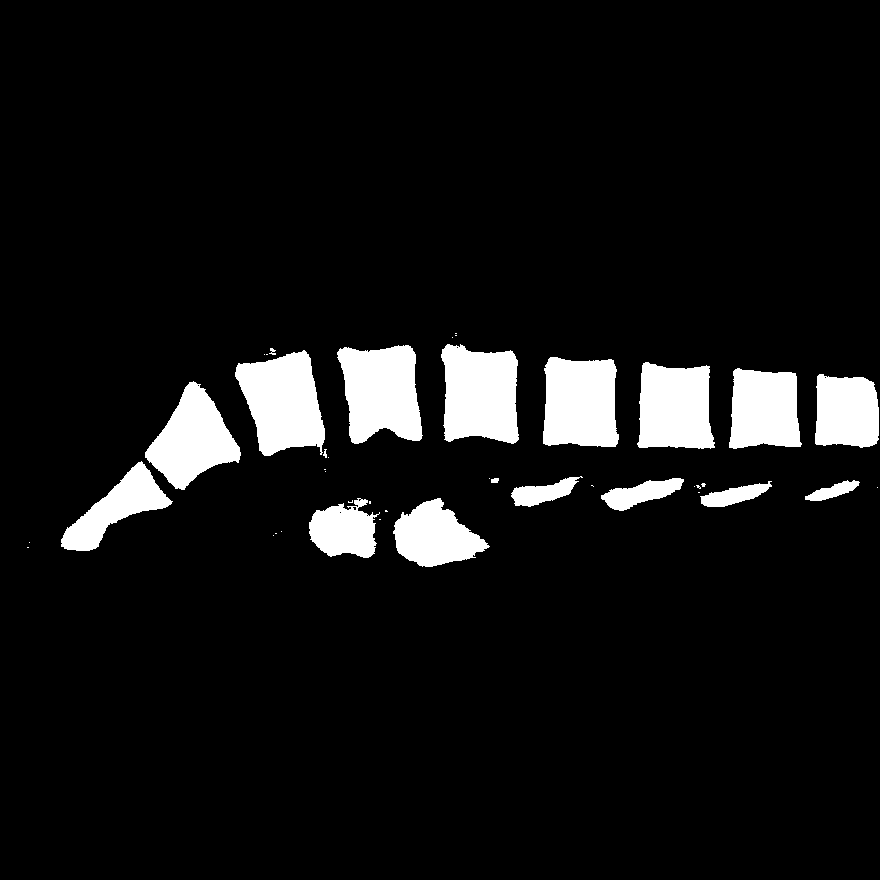

Supplement: S1 File — (ZIP) [file pone.0248303.s001.zip › Code and data/dataset/test_GT/181.png]

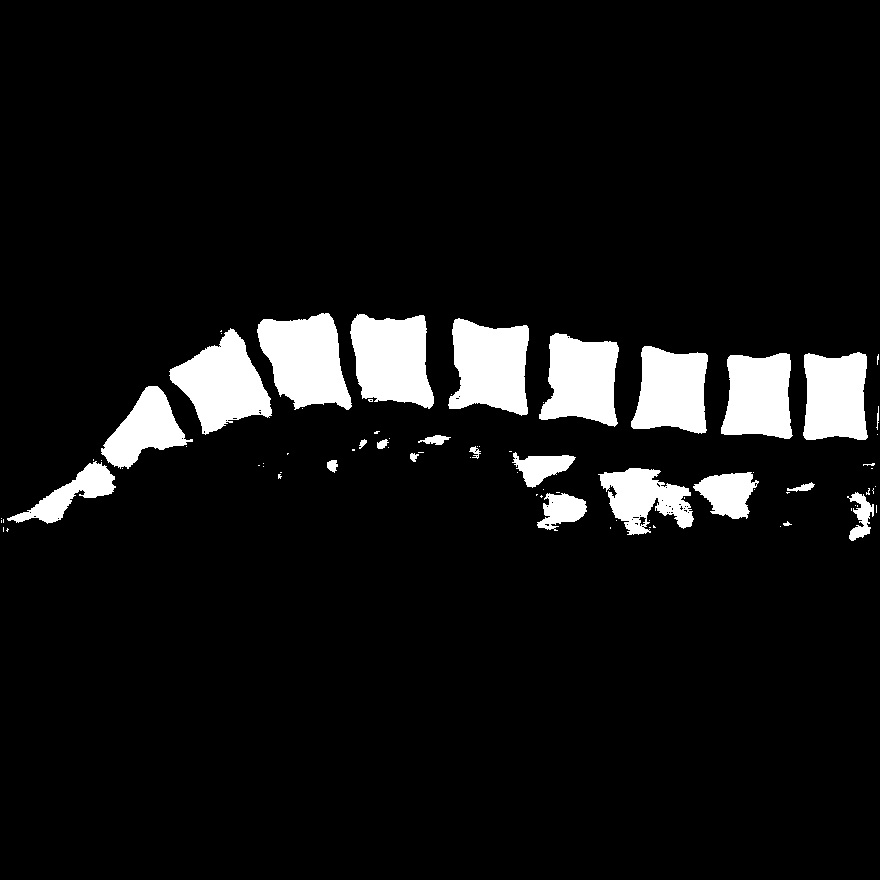

Supplement: S1 File — (ZIP) [file pone.0248303.s001.zip › Code and data/dataset/test_GT/182.png]

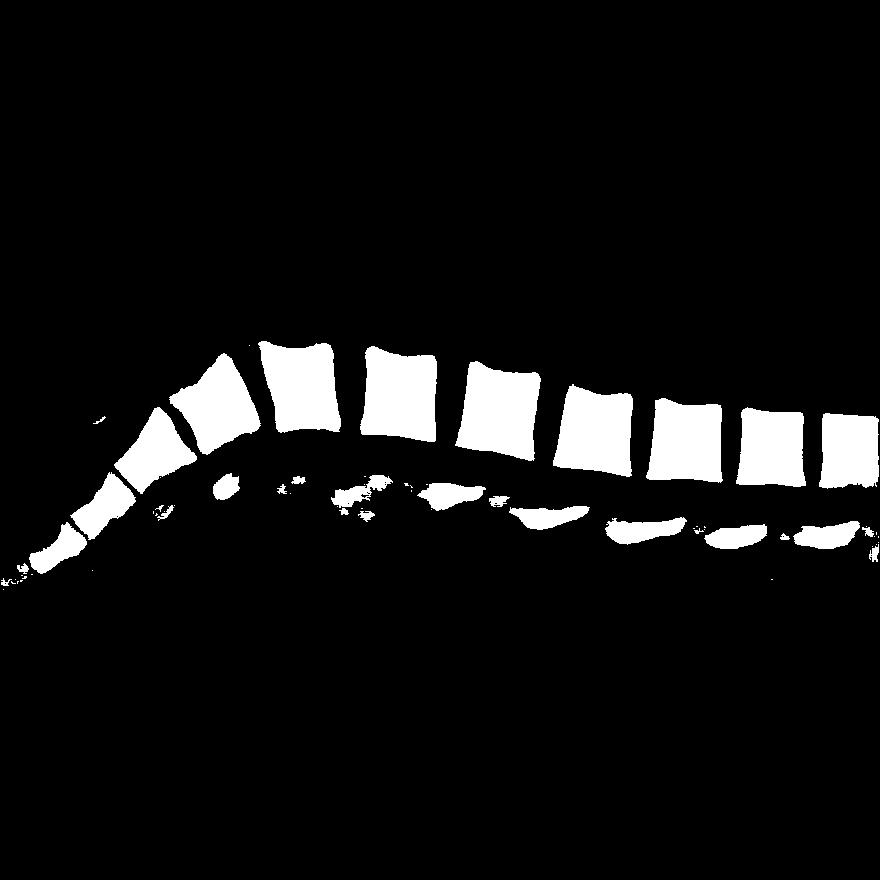

Supplement: S1 File — (ZIP) [file pone.0248303.s001.zip › Code and data/dataset/test_GT/183.png]

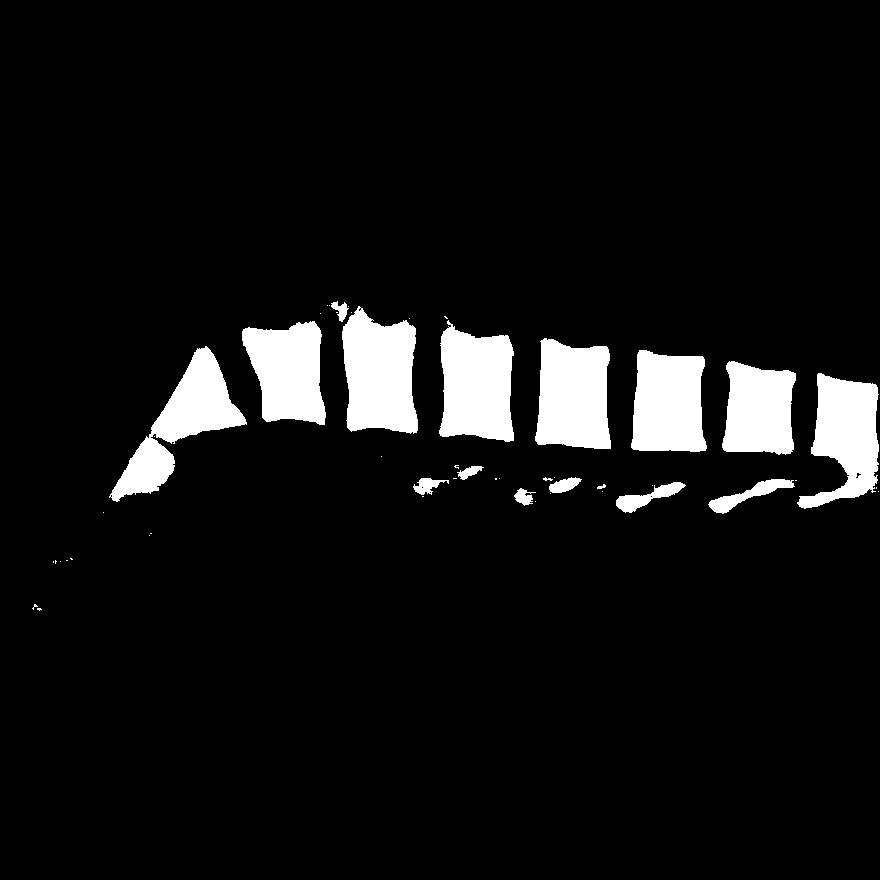

Supplement: S1 File — (ZIP) [file pone.0248303.s001.zip › Code and data/dataset/test_GT/184.png]

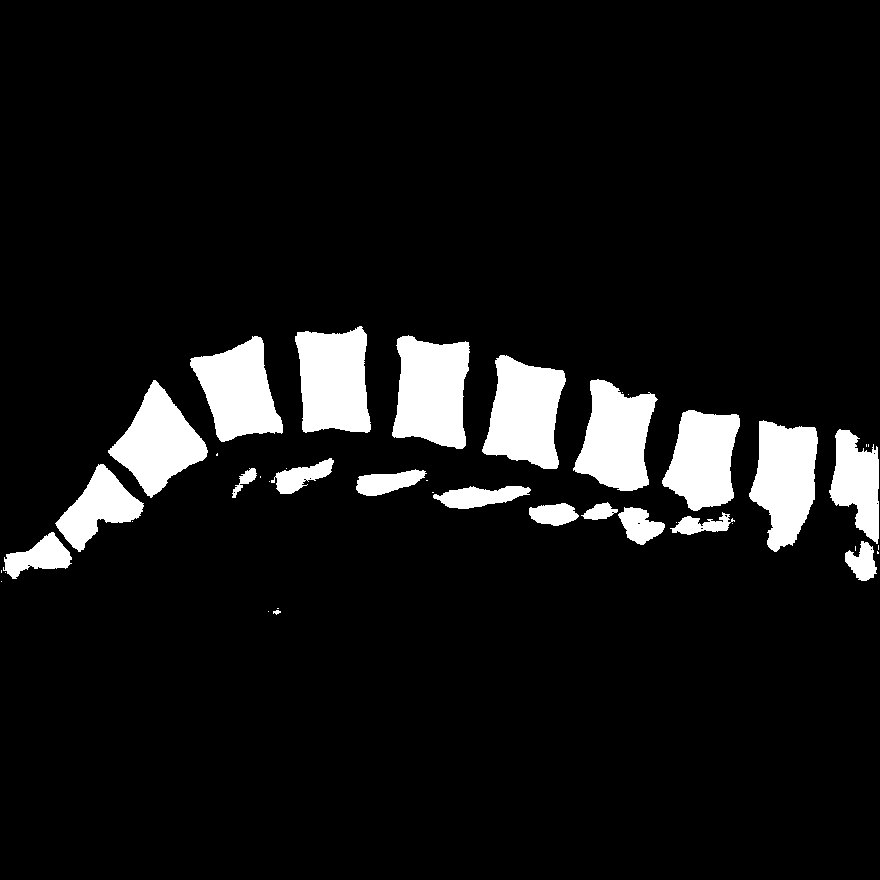

Supplement: S1 File — (ZIP) [file pone.0248303.s001.zip › Code and data/dataset/test_GT/185.png]

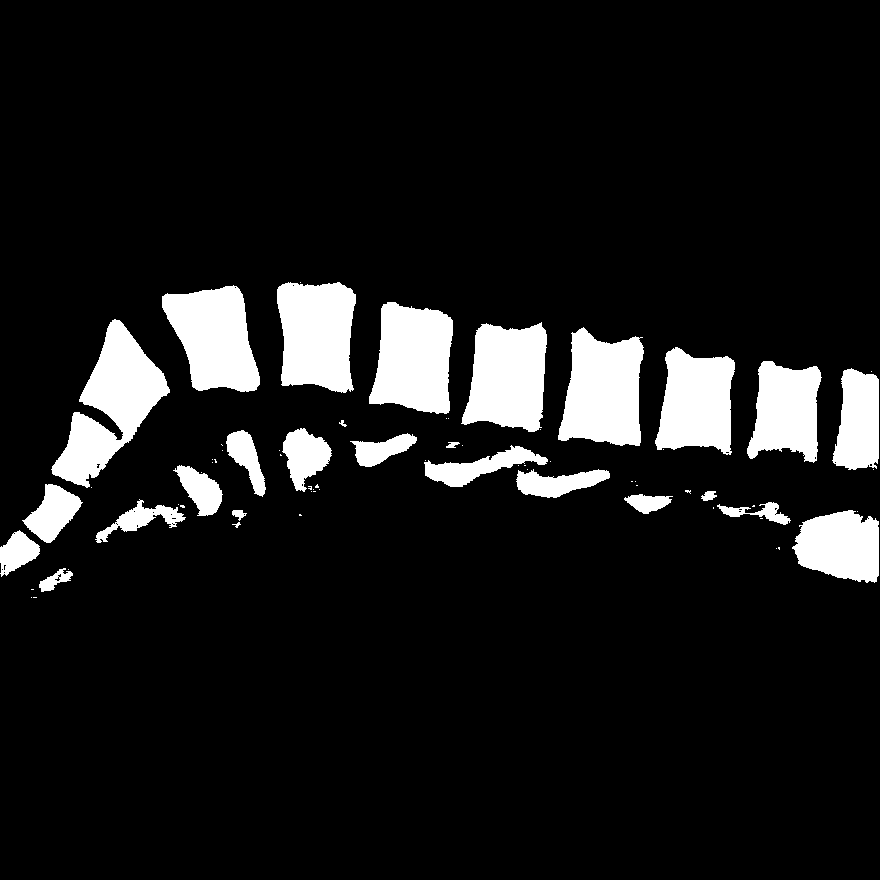

Supplement: S1 File — (ZIP) [file pone.0248303.s001.zip › Code and data/dataset/test_GT/186.png]

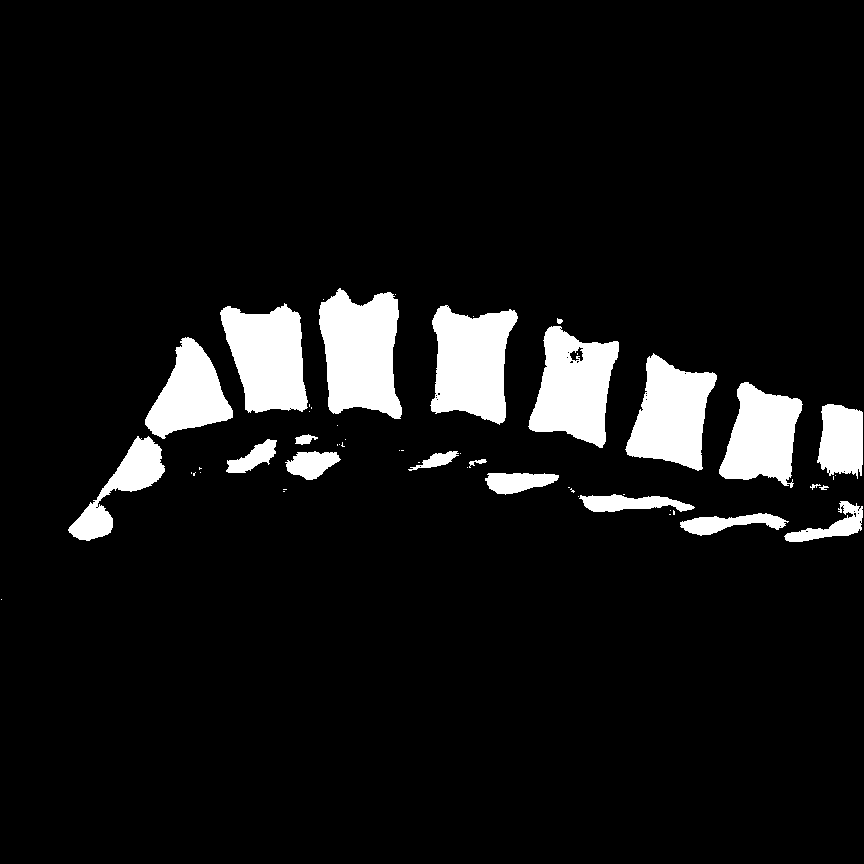

Supplement: S1 File — (ZIP) [file pone.0248303.s001.zip › Code and data/dataset/test_GT/187.png]

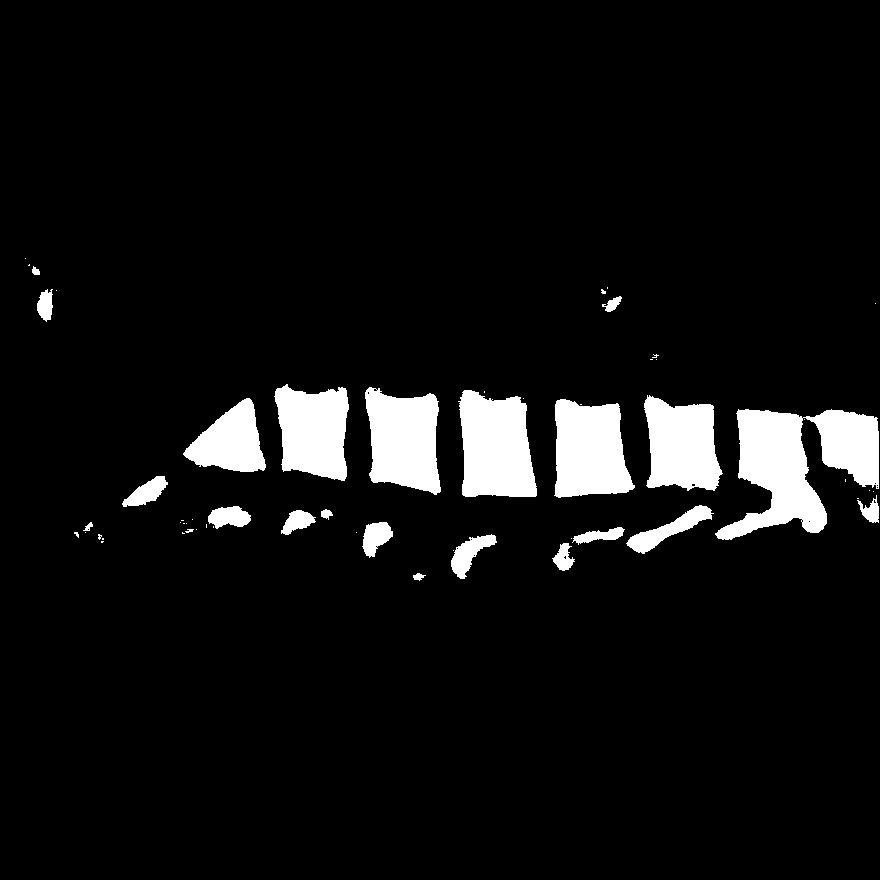

Supplement: S1 File — (ZIP) [file pone.0248303.s001.zip › Code and data/dataset/test_GT/188.png]

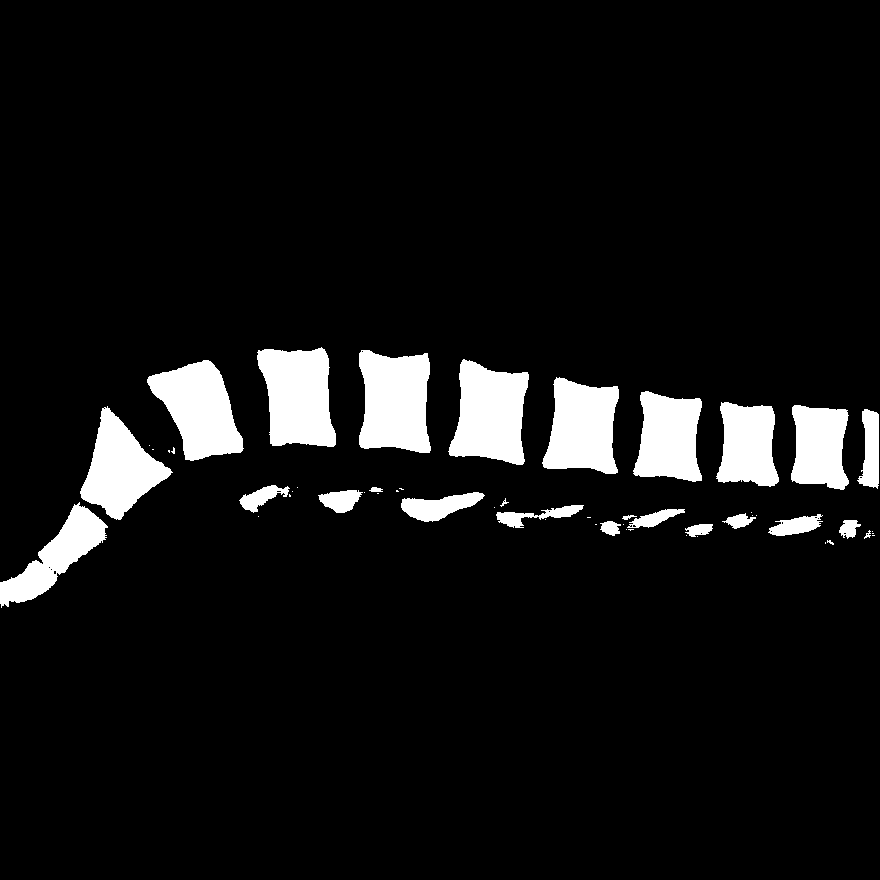

Supplement: S1 File — (ZIP) [file pone.0248303.s001.zip › Code and data/dataset/test_GT/189.png]

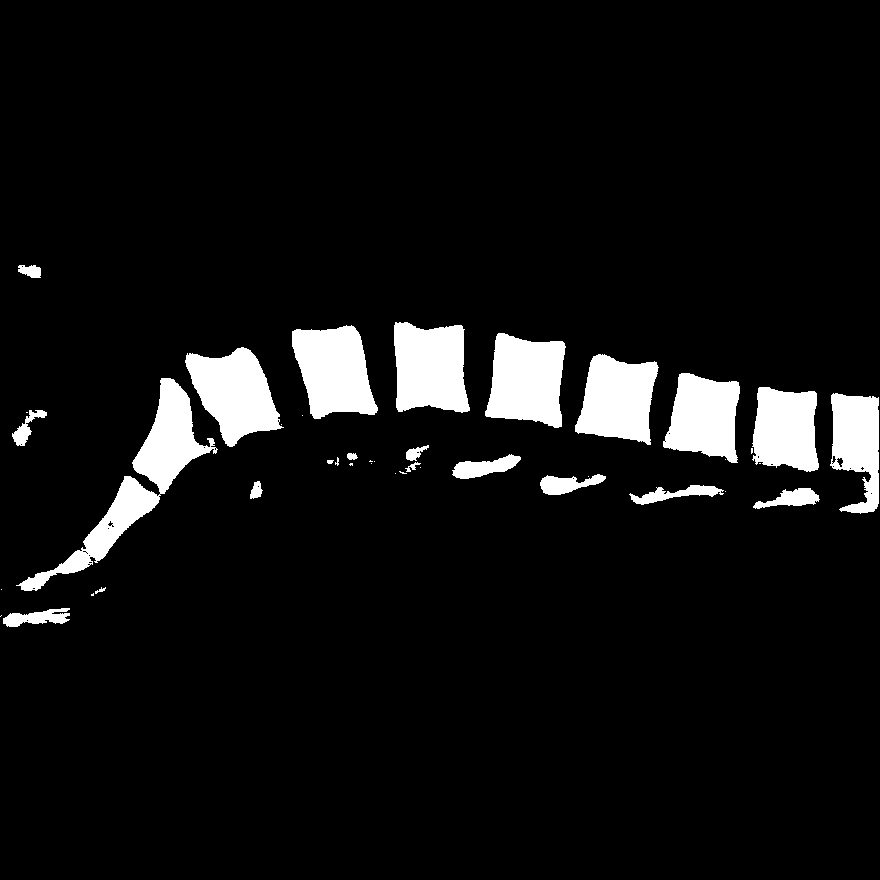

Supplement: S1 File — (ZIP) [file pone.0248303.s001.zip › Code and data/dataset/test_GT/190.png]

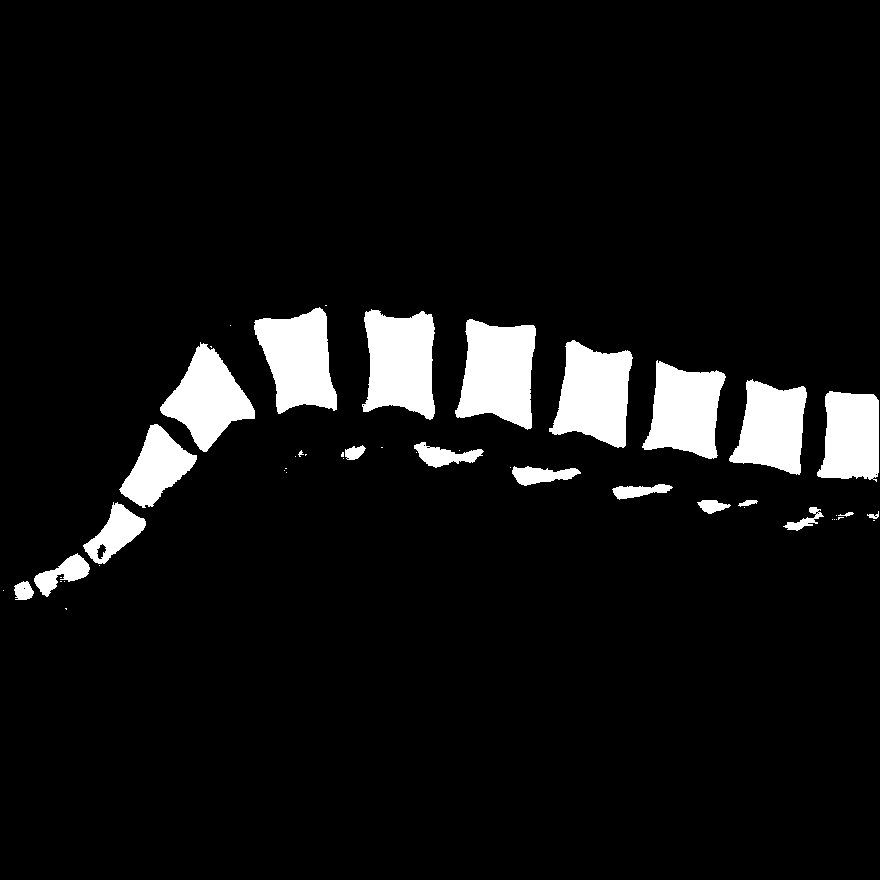

Supplement: S1 File — (ZIP) [file pone.0248303.s001.zip › Code and data/dataset/test_GT/191.png]

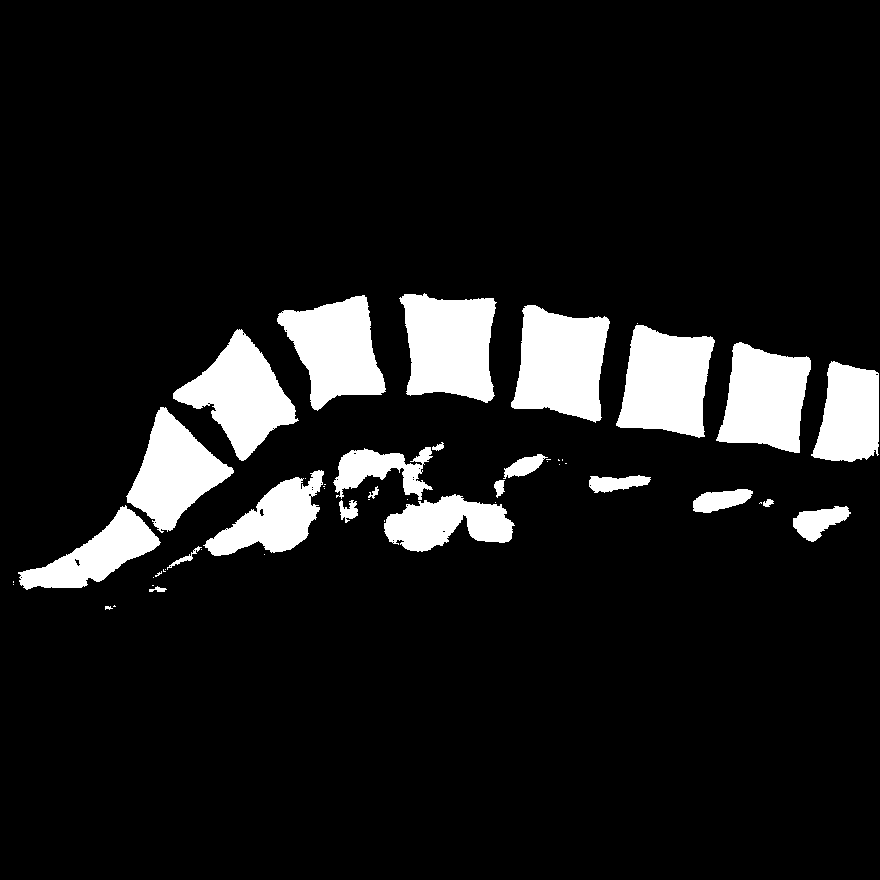

Supplement: S1 File — (ZIP) [file pone.0248303.s001.zip › Code and data/dataset/test_GT/192.png]

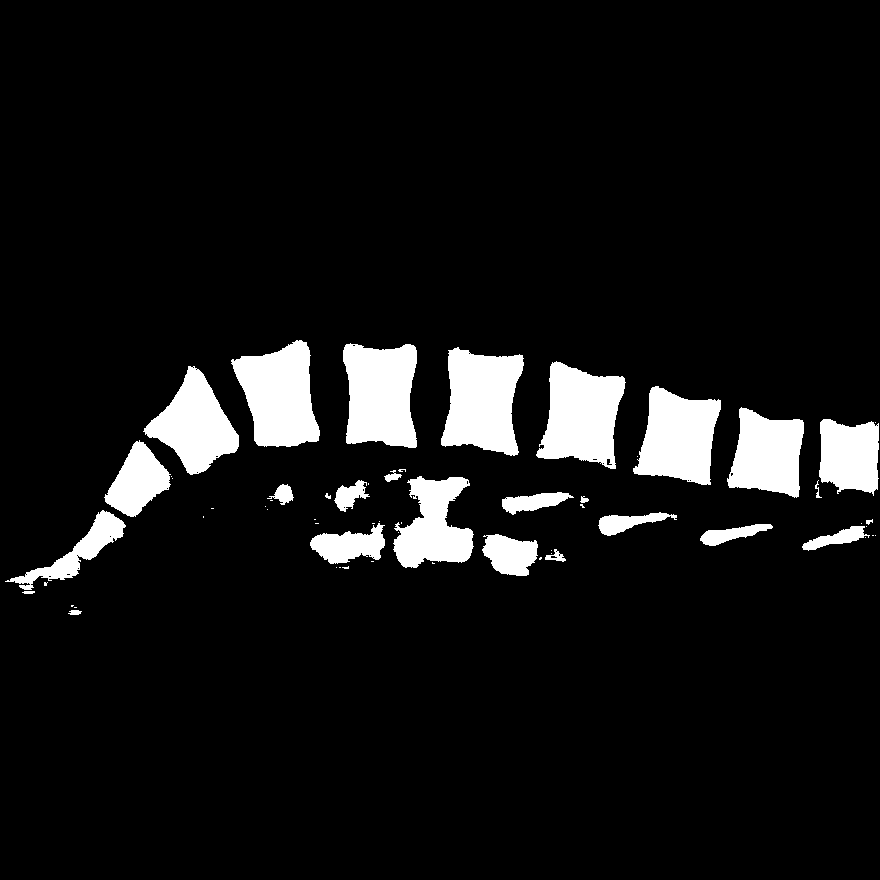

Supplement: S1 File — (ZIP) [file pone.0248303.s001.zip › Code and data/dataset/test_GT/193.png]

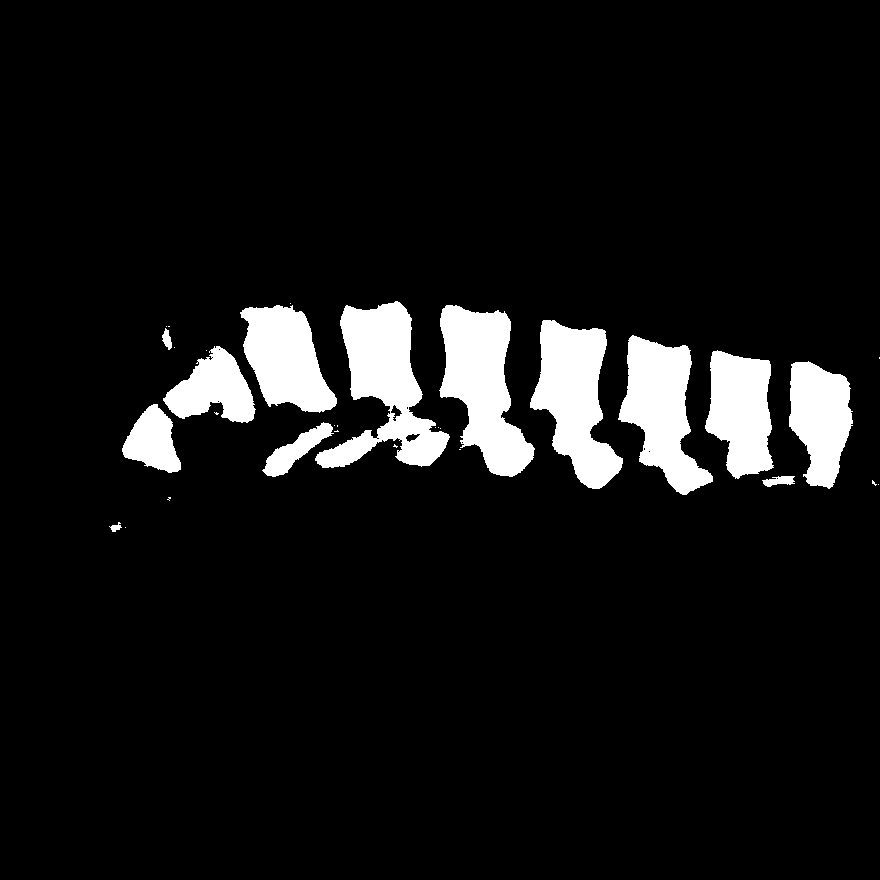

Supplement: S1 File — (ZIP) [file pone.0248303.s001.zip › Code and data/dataset/test_GT/194.png]

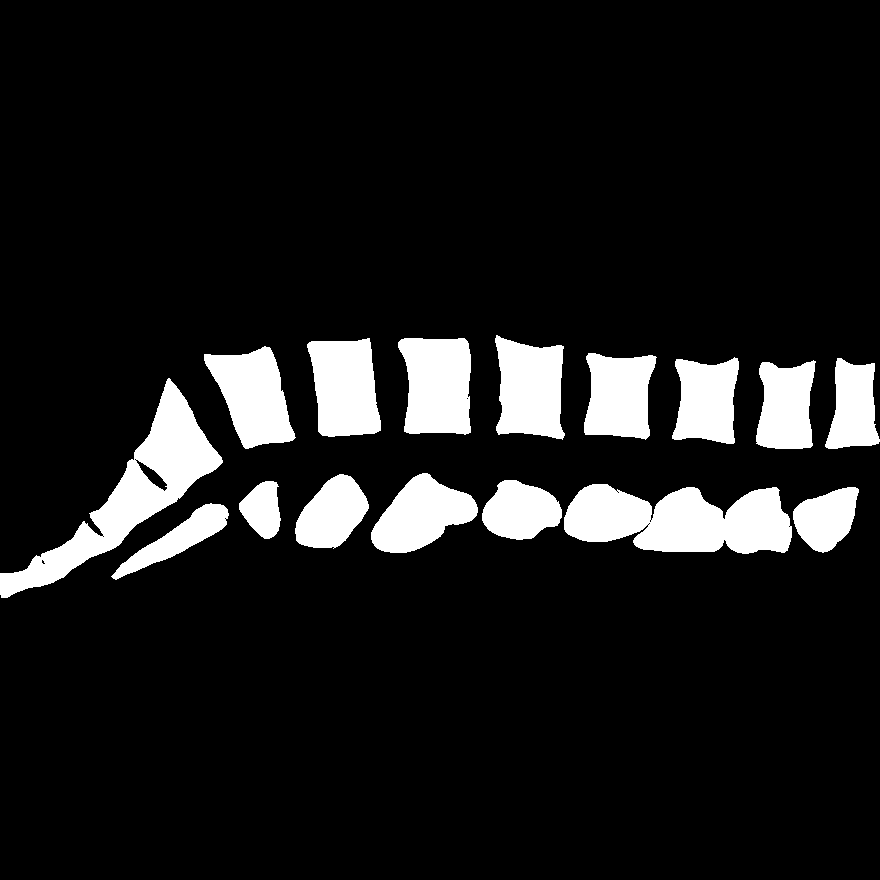

Supplement: S1 File — (ZIP) [file pone.0248303.s001.zip › Code and data/dataset/test_GT/195.png]

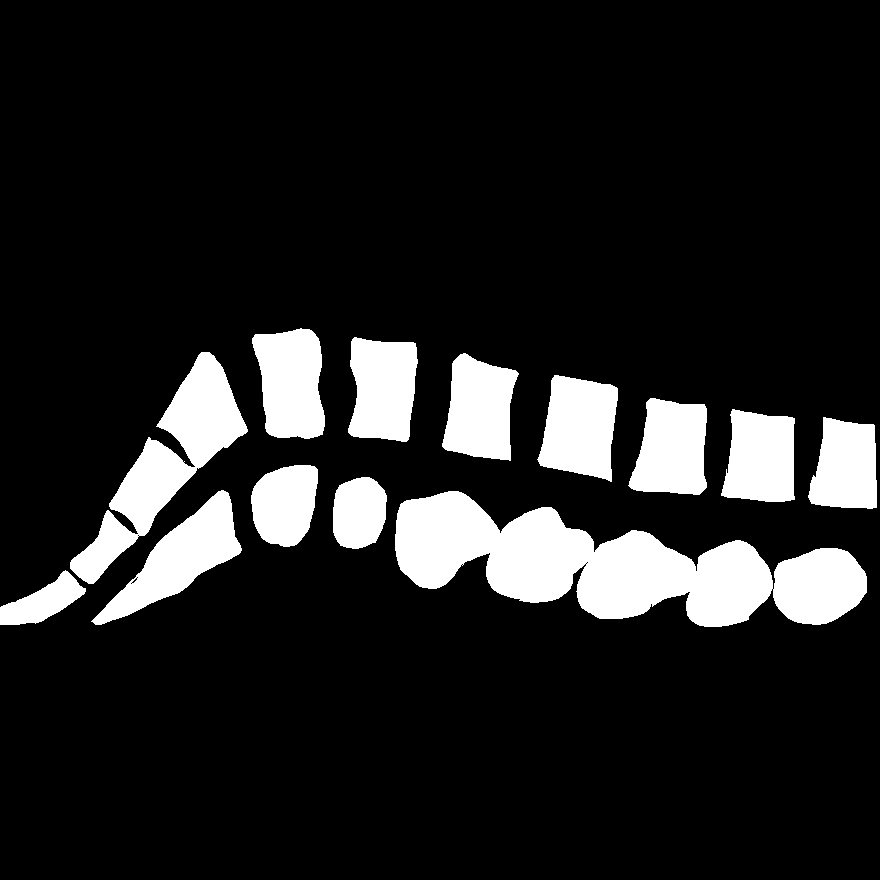

Supplement: S1 File — (ZIP) [file pone.0248303.s001.zip › Code and data/dataset/test_GT/196.png]

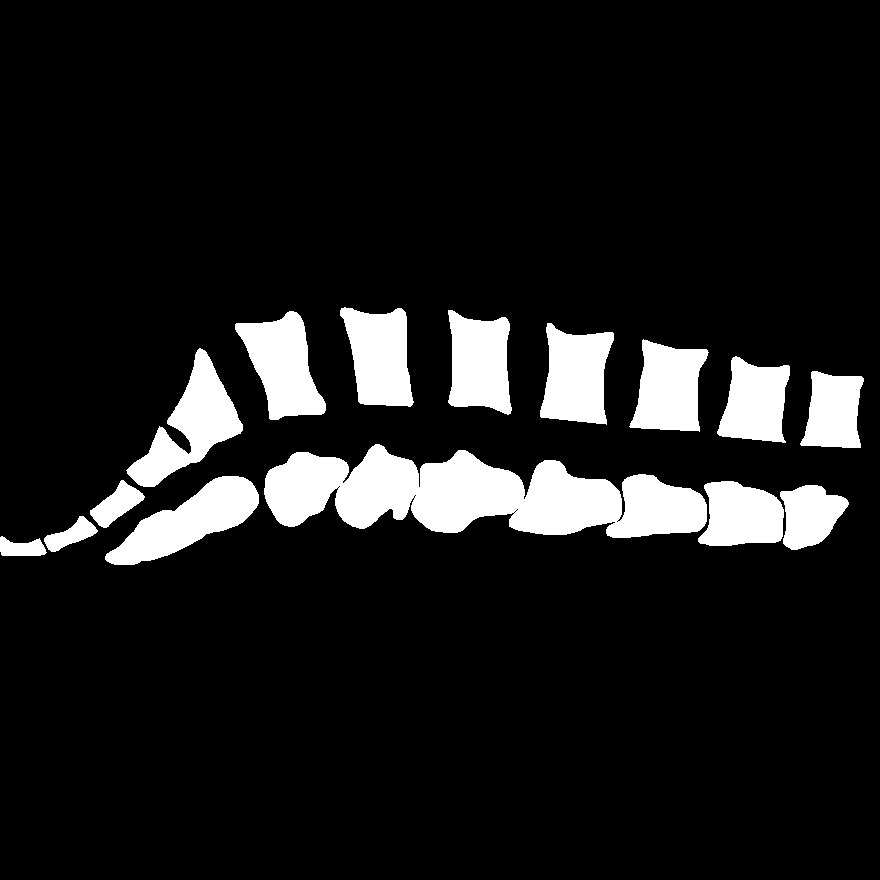

Supplement: S1 File — (ZIP) [file pone.0248303.s001.zip › Code and data/dataset/test_GT/197.png]

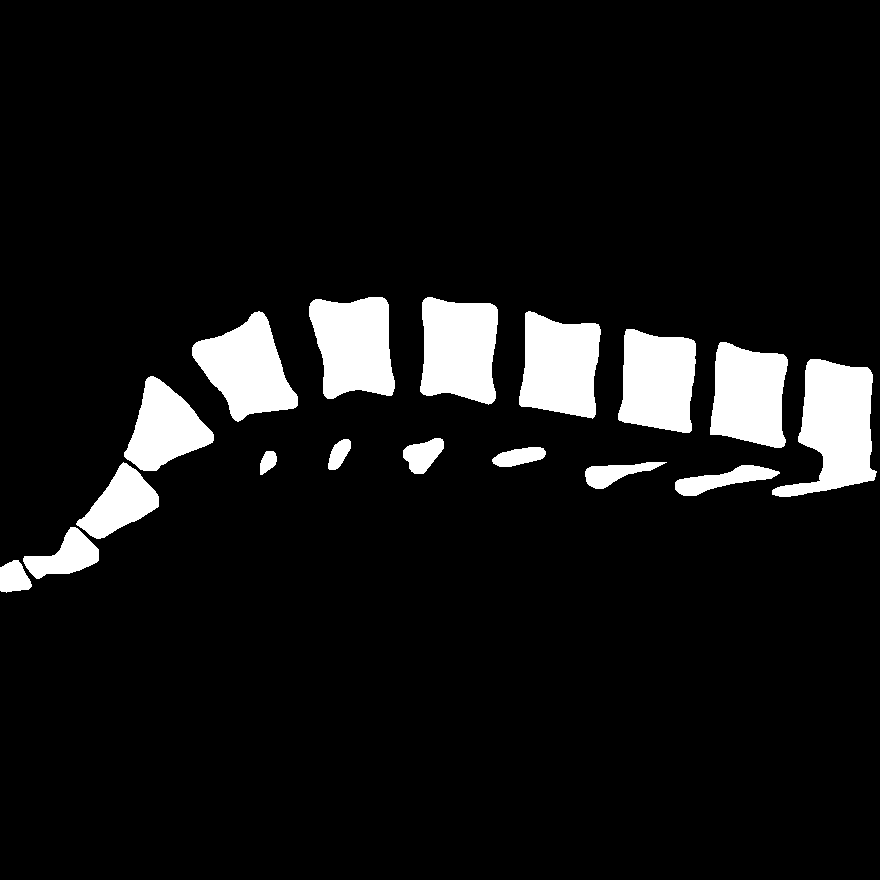

Supplement: S1 File — (ZIP) [file pone.0248303.s001.zip › Code and data/dataset/test_GT/198.png]

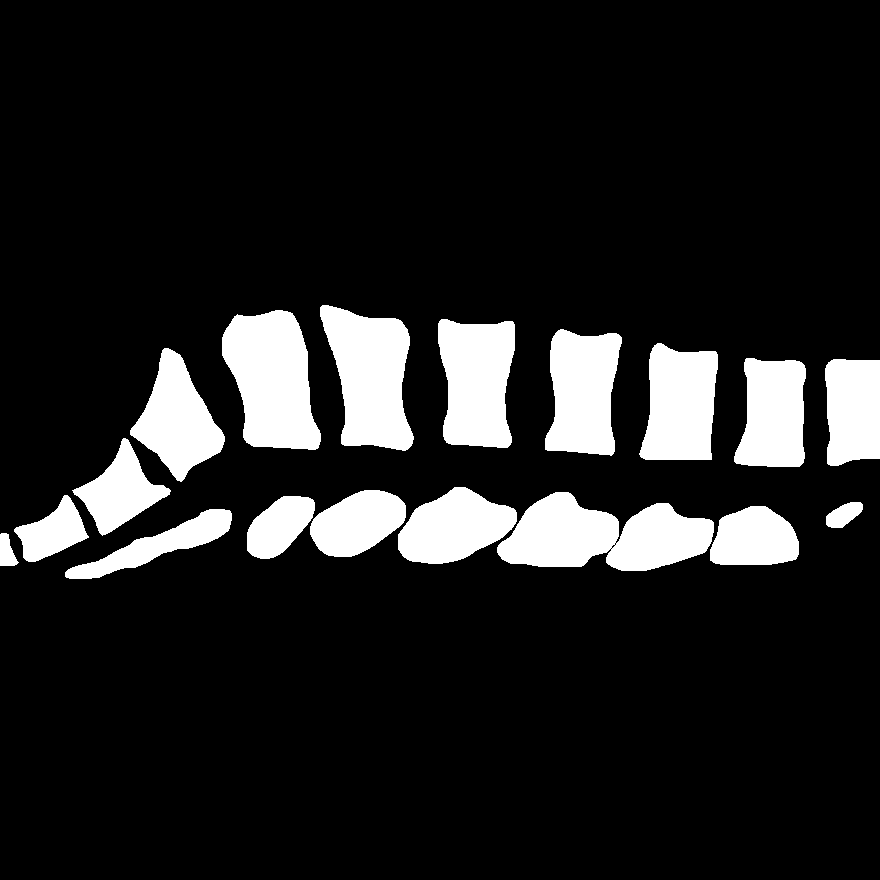

Supplement: S1 File — (ZIP) [file pone.0248303.s001.zip › Code and data/dataset/test_GT/199.png]

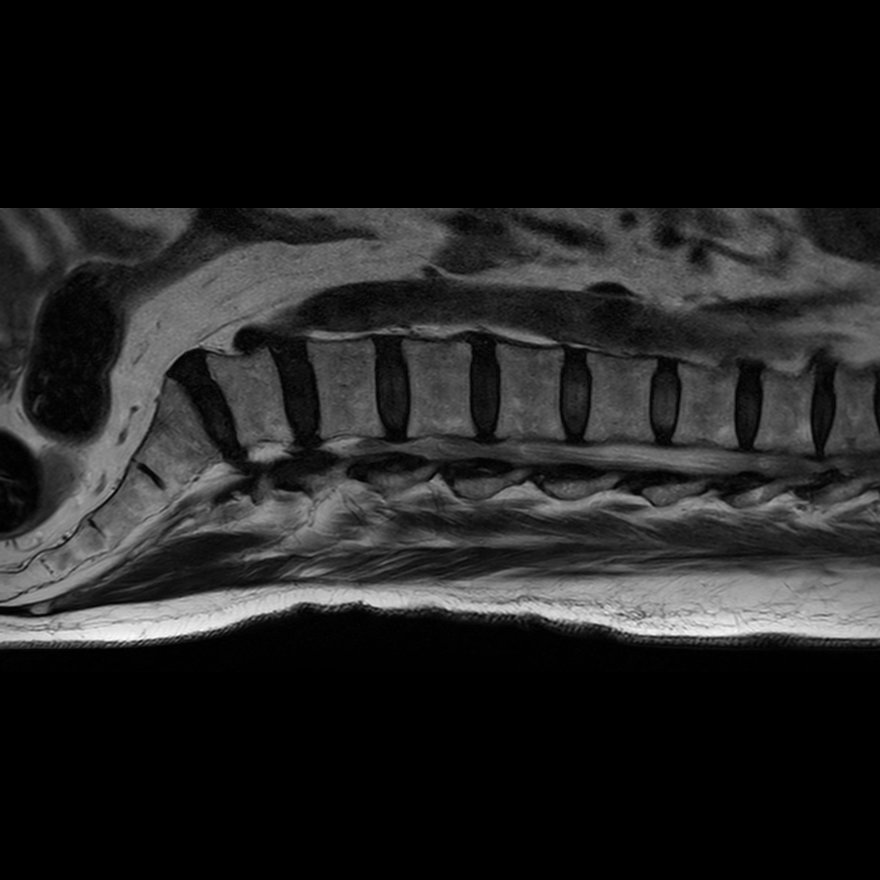

Supplement: S1 File — (ZIP) [file pone.0248303.s001.zip › Code and data/dataset/train/0.png]

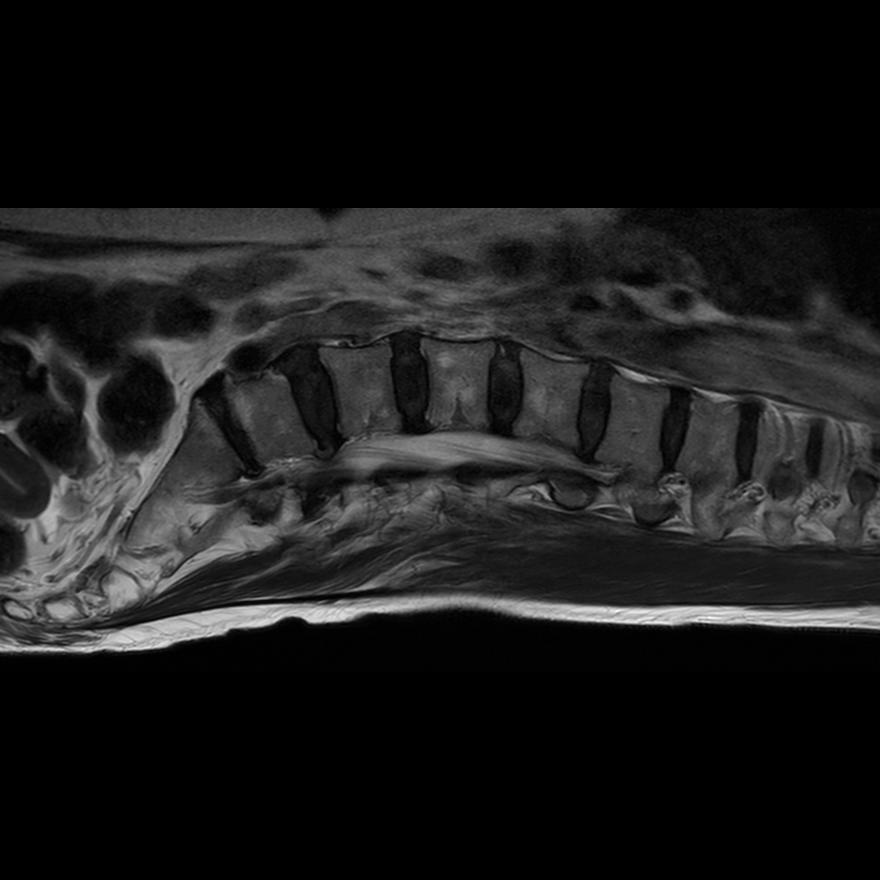

Supplement: S1 File — (ZIP) [file pone.0248303.s001.zip › Code and data/dataset/train/10.png]

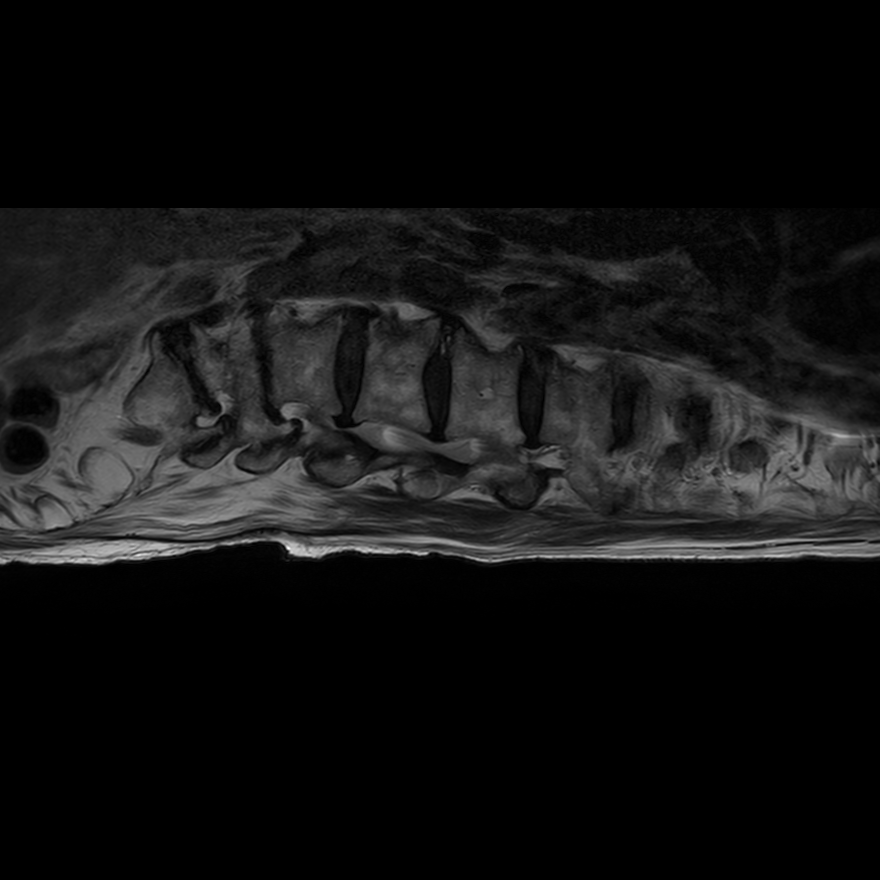

Supplement: S1 File — (ZIP) [file pone.0248303.s001.zip › Code and data/dataset/train/100.png]

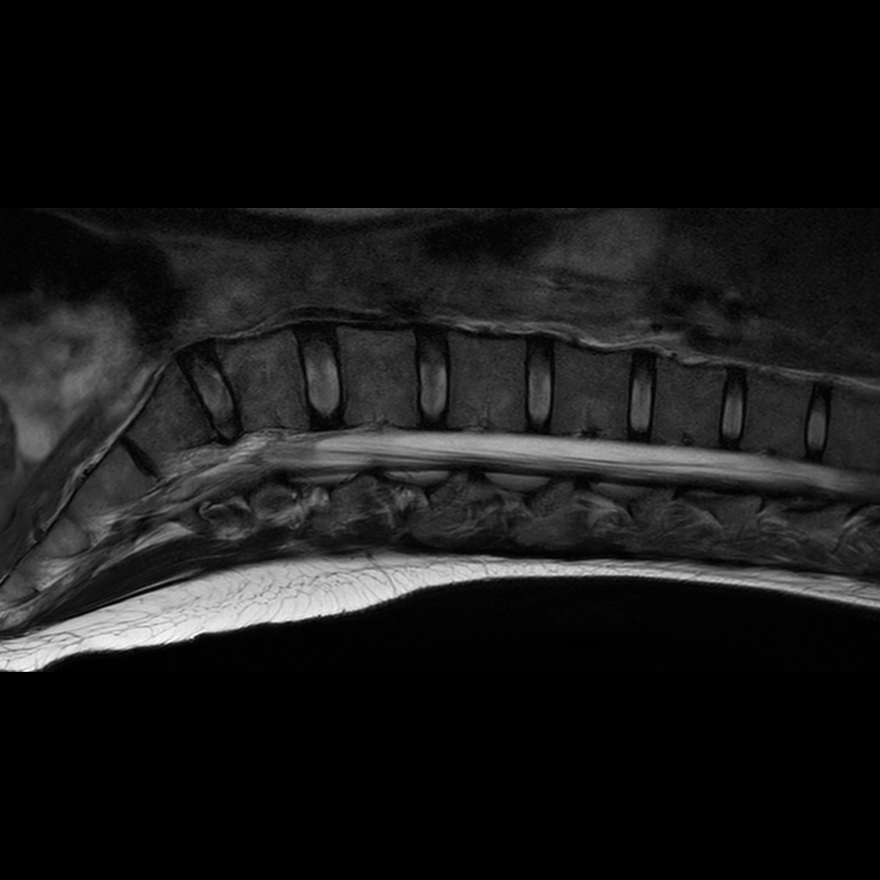

Supplement: S1 File — (ZIP) [file pone.0248303.s001.zip › Code and data/dataset/train/101.png]

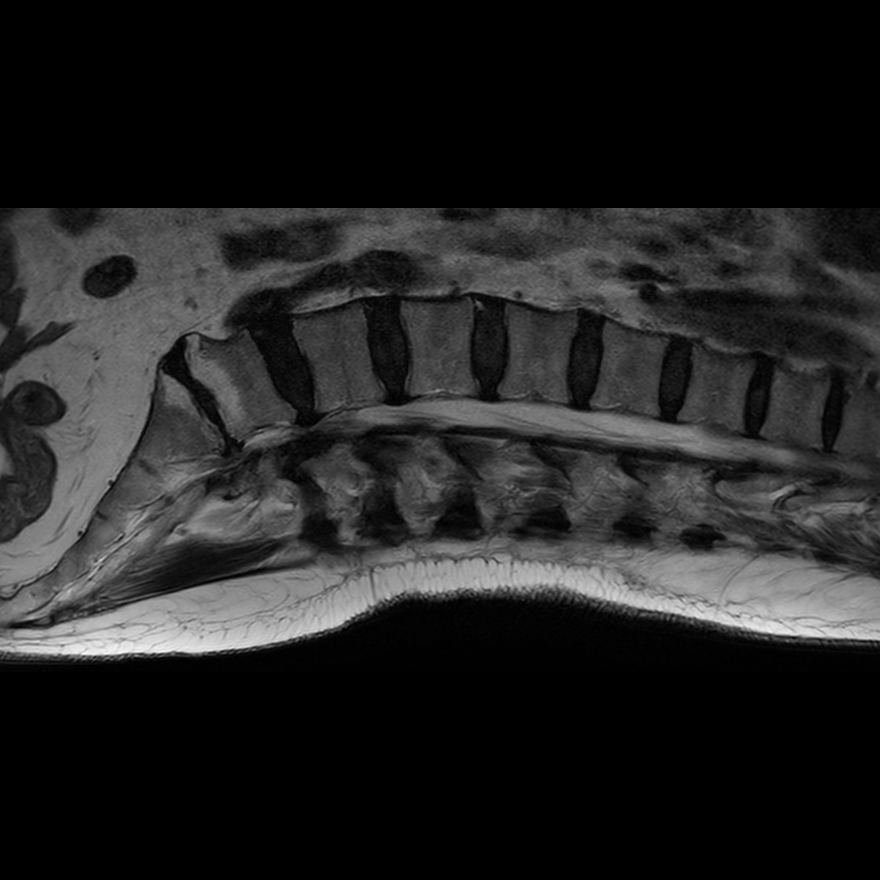

Supplement: S1 File — (ZIP) [file pone.0248303.s001.zip › Code and data/dataset/train/102.png]

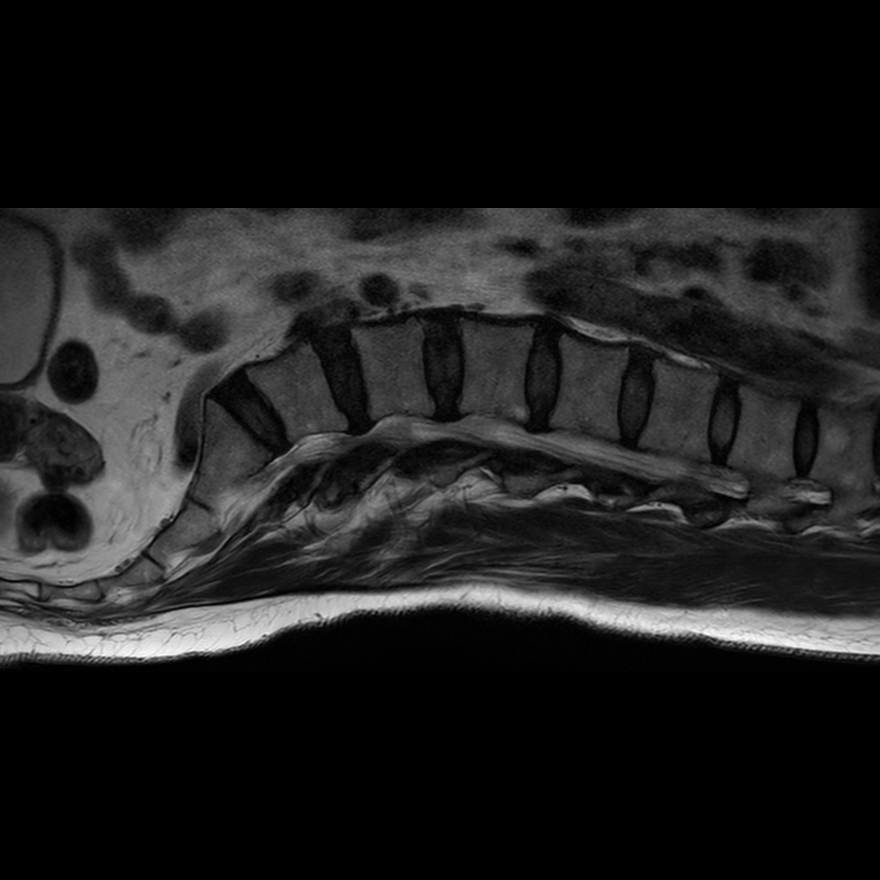

Supplement: S1 File — (ZIP) [file pone.0248303.s001.zip › Code and data/dataset/train/103.png]

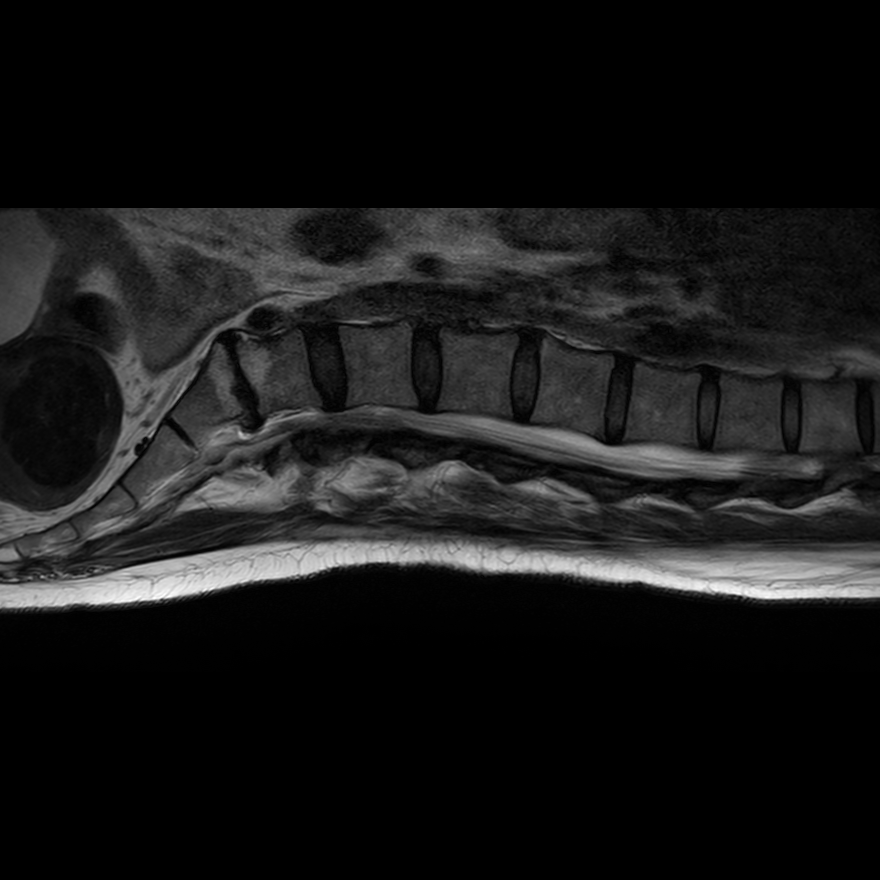

Supplement: S1 File — (ZIP) [file pone.0248303.s001.zip › Code and data/dataset/train/104.png]

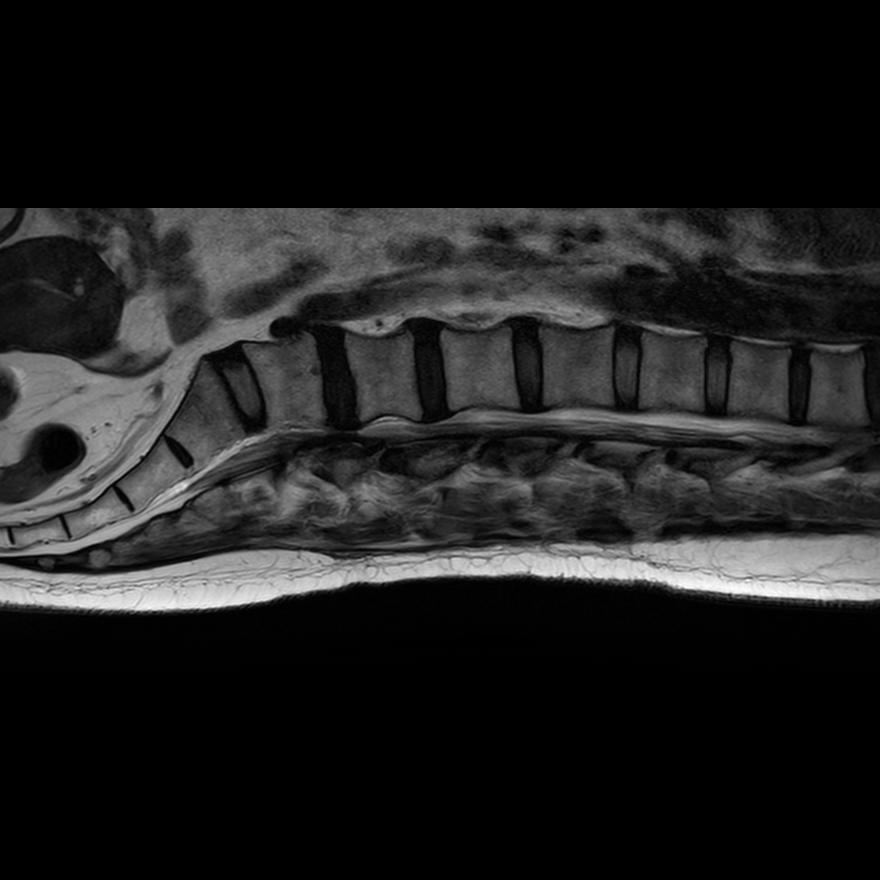

Supplement: S1 File — (ZIP) [file pone.0248303.s001.zip › Code and data/dataset/train/105.png]

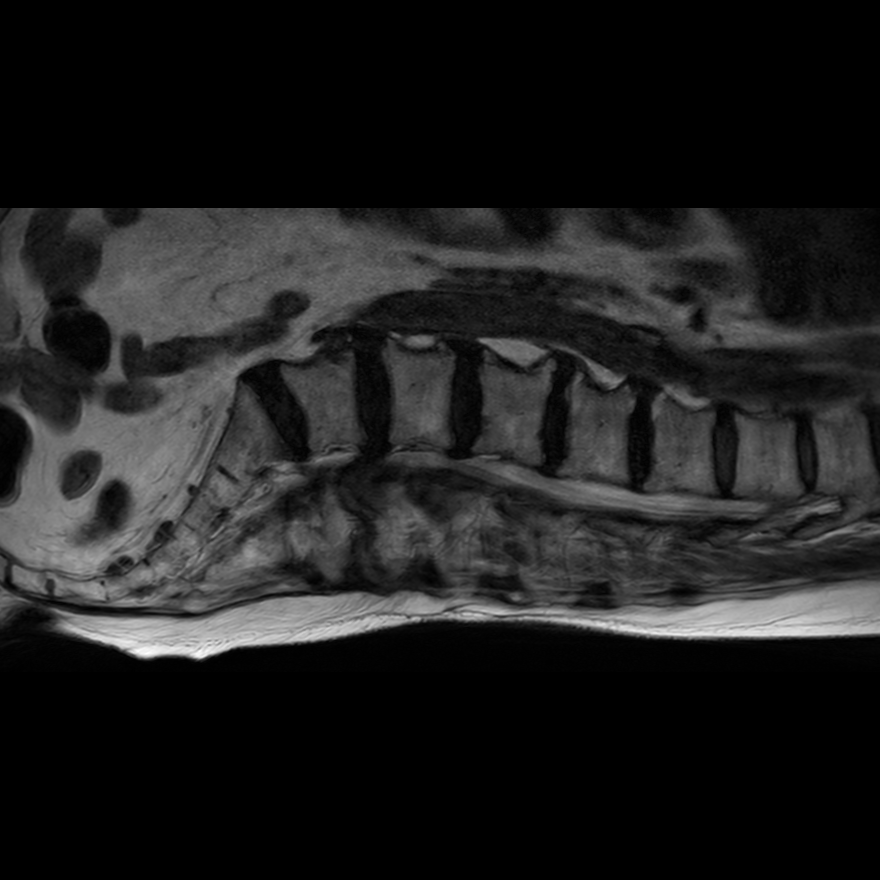

Supplement: S1 File — (ZIP) [file pone.0248303.s001.zip › Code and data/dataset/train/106.png]

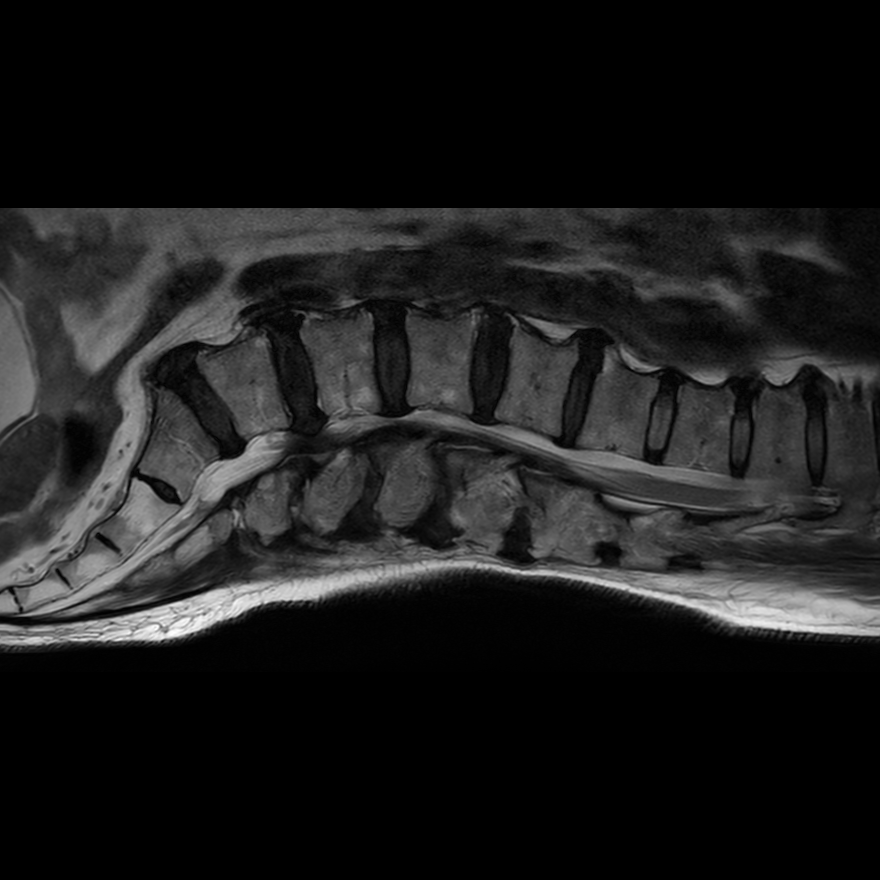

Supplement: S1 File — (ZIP) [file pone.0248303.s001.zip › Code and data/dataset/train/107.png]

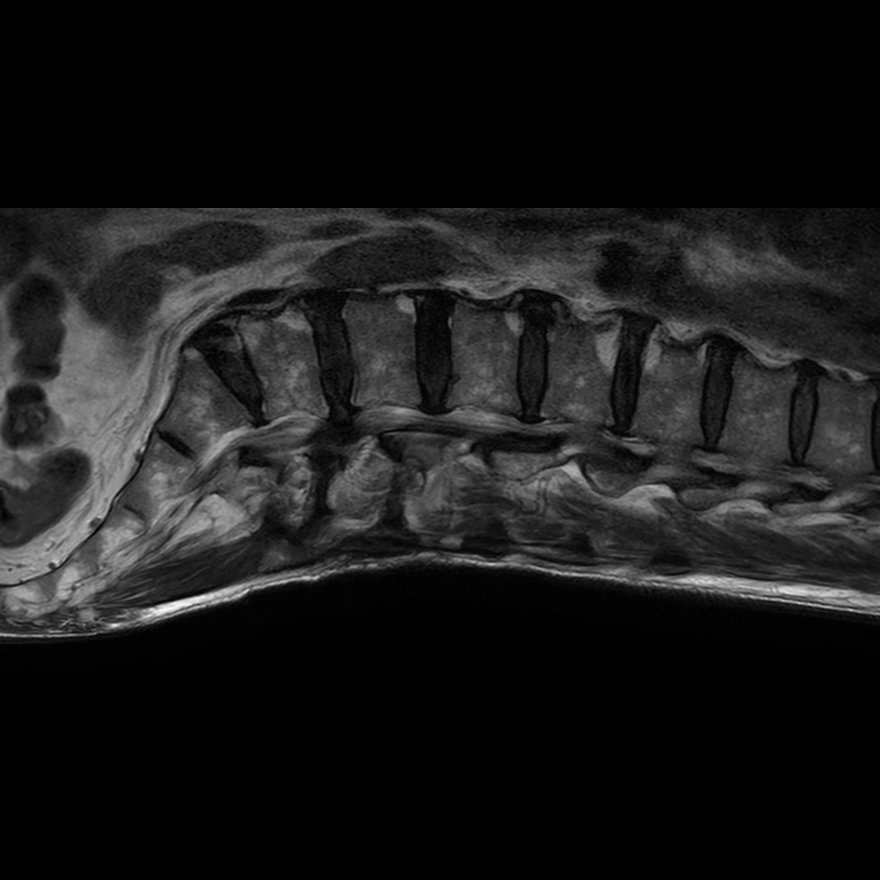

Supplement: S1 File — (ZIP) [file pone.0248303.s001.zip › Code and data/dataset/train/108.png]

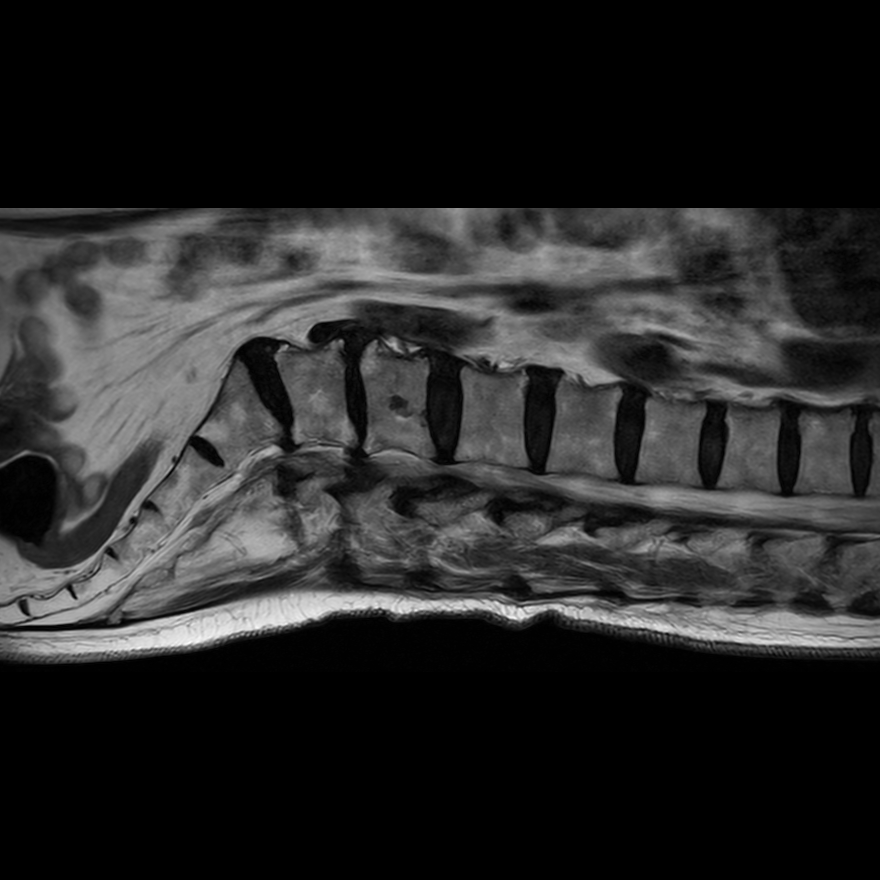

Supplement: S1 File — (ZIP) [file pone.0248303.s001.zip › Code and data/dataset/train/109.png]

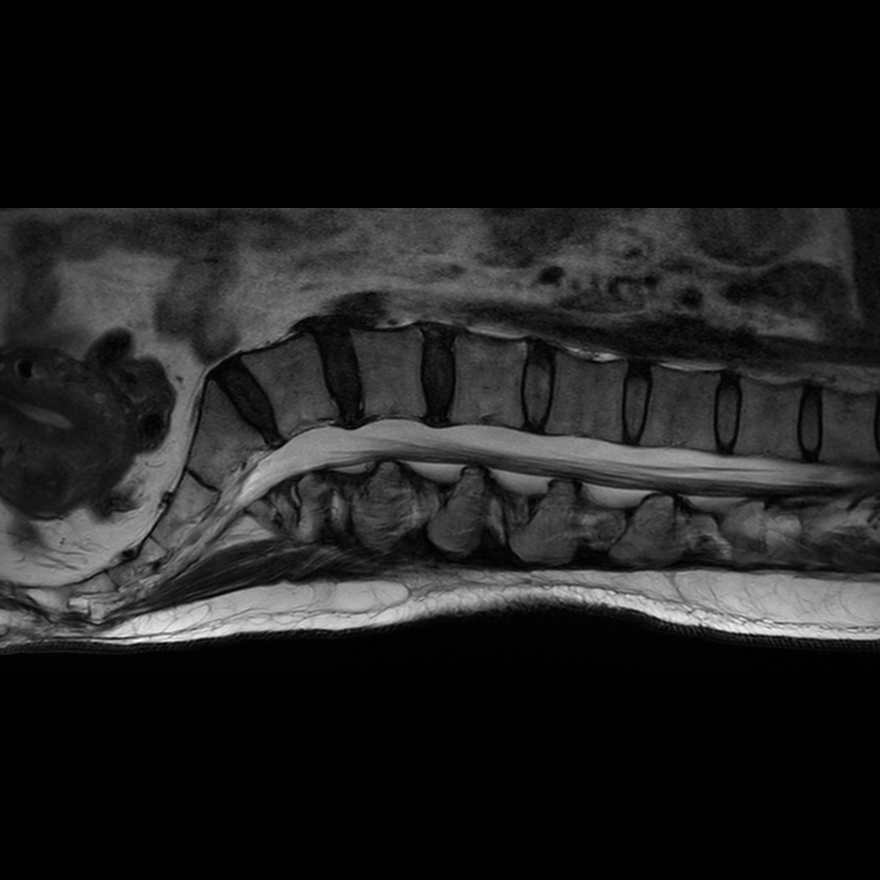

Supplement: S1 File — (ZIP) [file pone.0248303.s001.zip › Code and data/dataset/train/11.png]

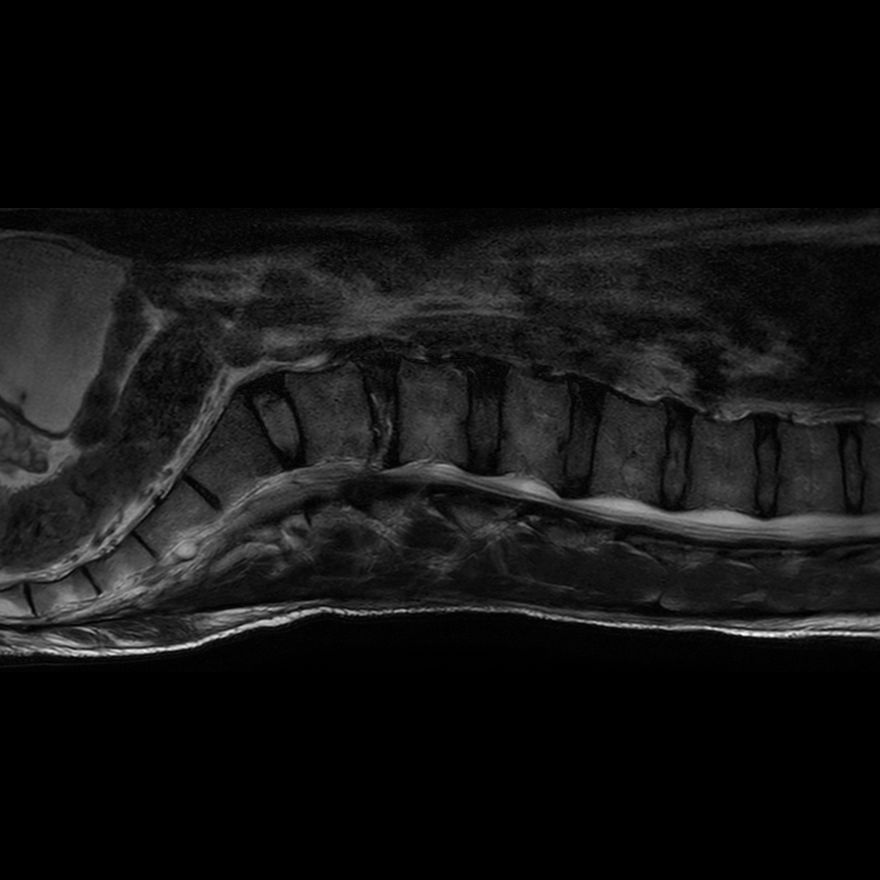

Supplement: S1 File — (ZIP) [file pone.0248303.s001.zip › Code and data/dataset/train/110.png]

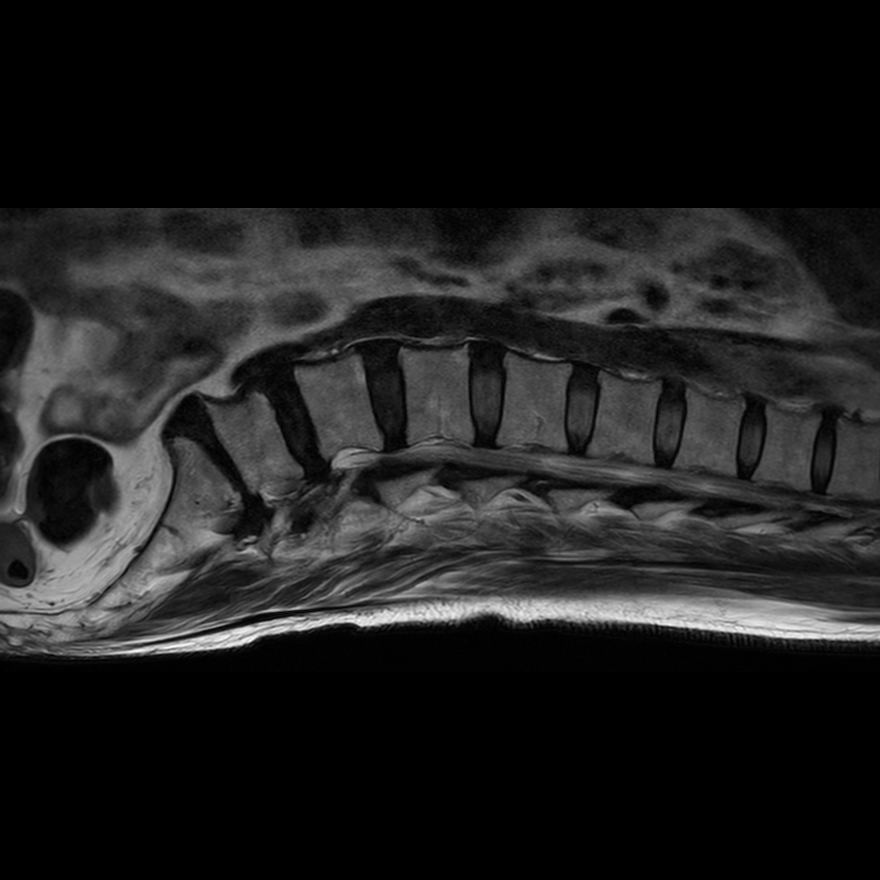

Supplement: S1 File — (ZIP) [file pone.0248303.s001.zip › Code and data/dataset/train/111.png]

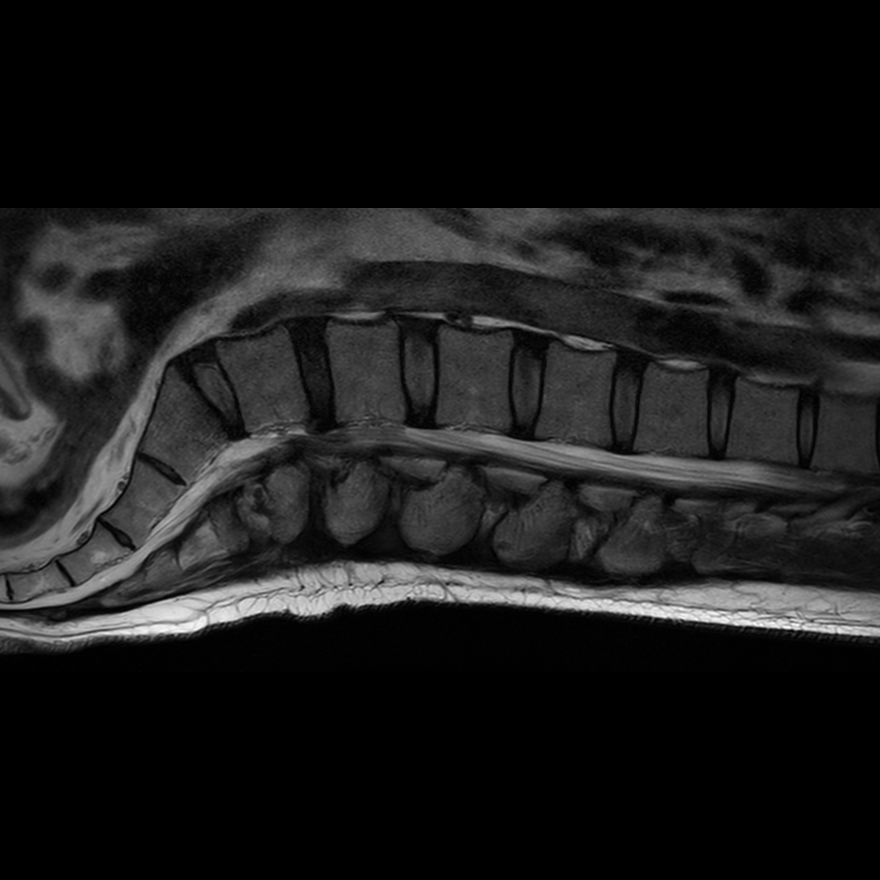

Supplement: S1 File — (ZIP) [file pone.0248303.s001.zip › Code and data/dataset/train/112.png]

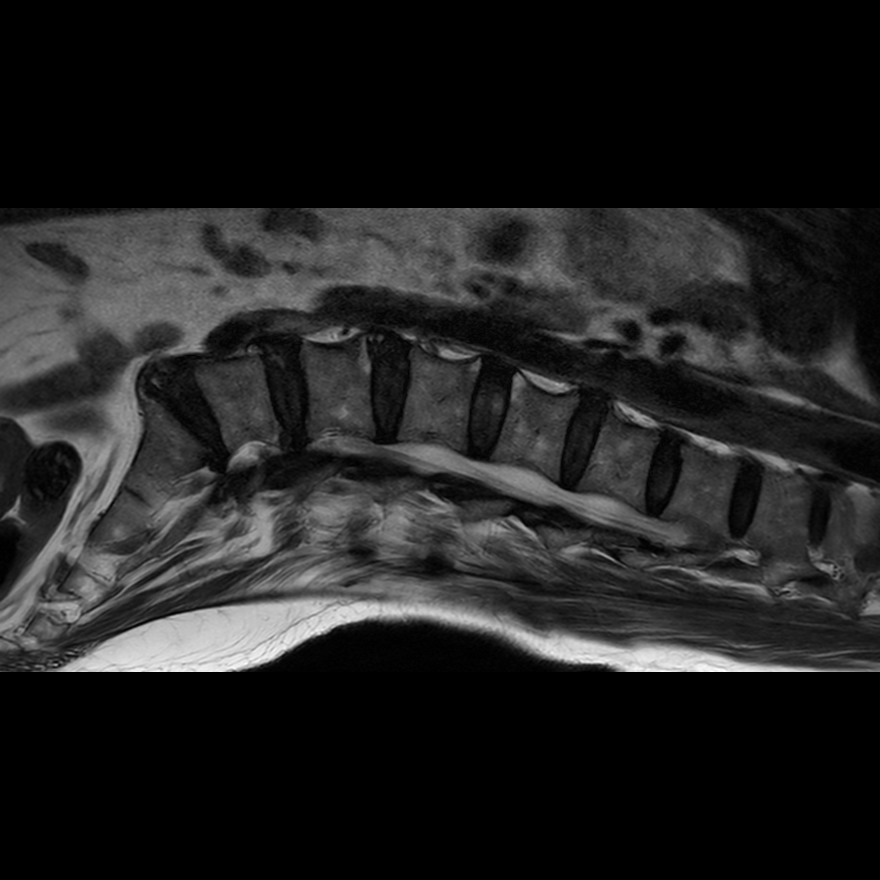

Supplement: S1 File — (ZIP) [file pone.0248303.s001.zip › Code and data/dataset/train/113.png]

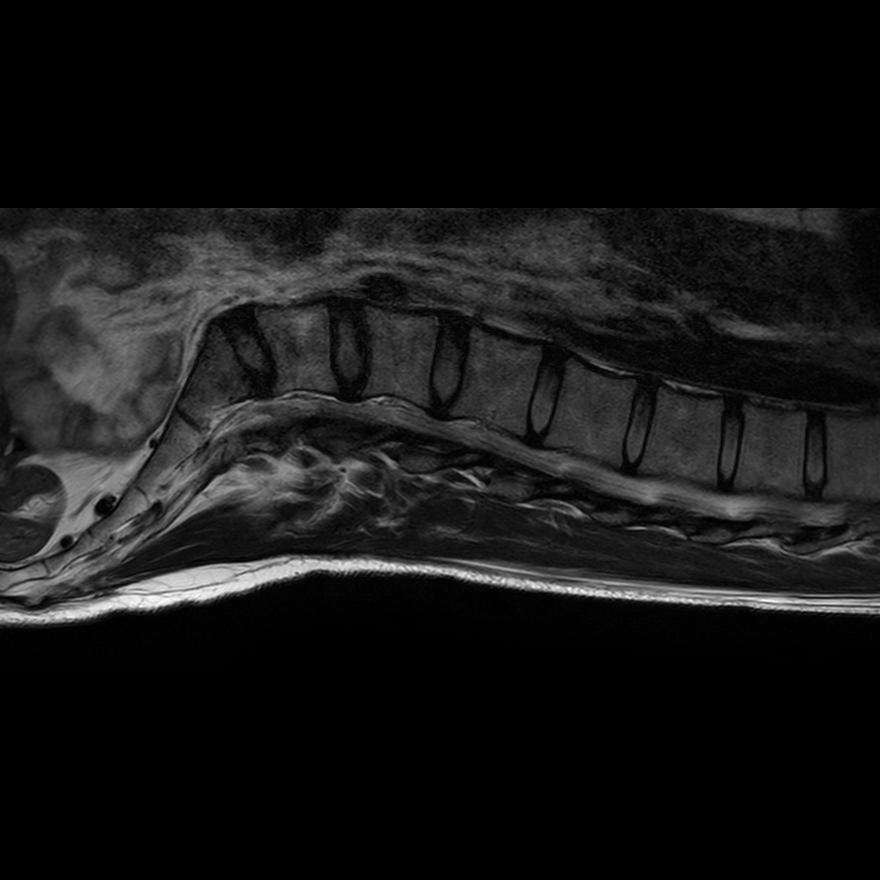

Supplement: S1 File — (ZIP) [file pone.0248303.s001.zip › Code and data/dataset/train/114.png]

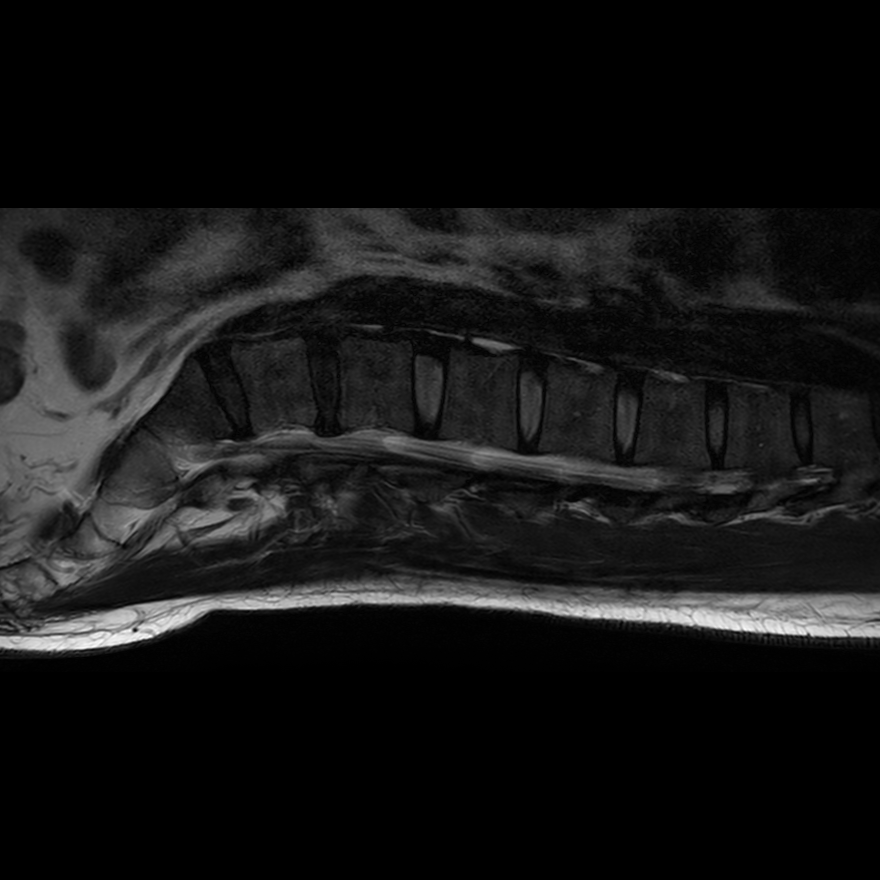

Supplement: S1 File — (ZIP) [file pone.0248303.s001.zip › Code and data/dataset/train/115.png]

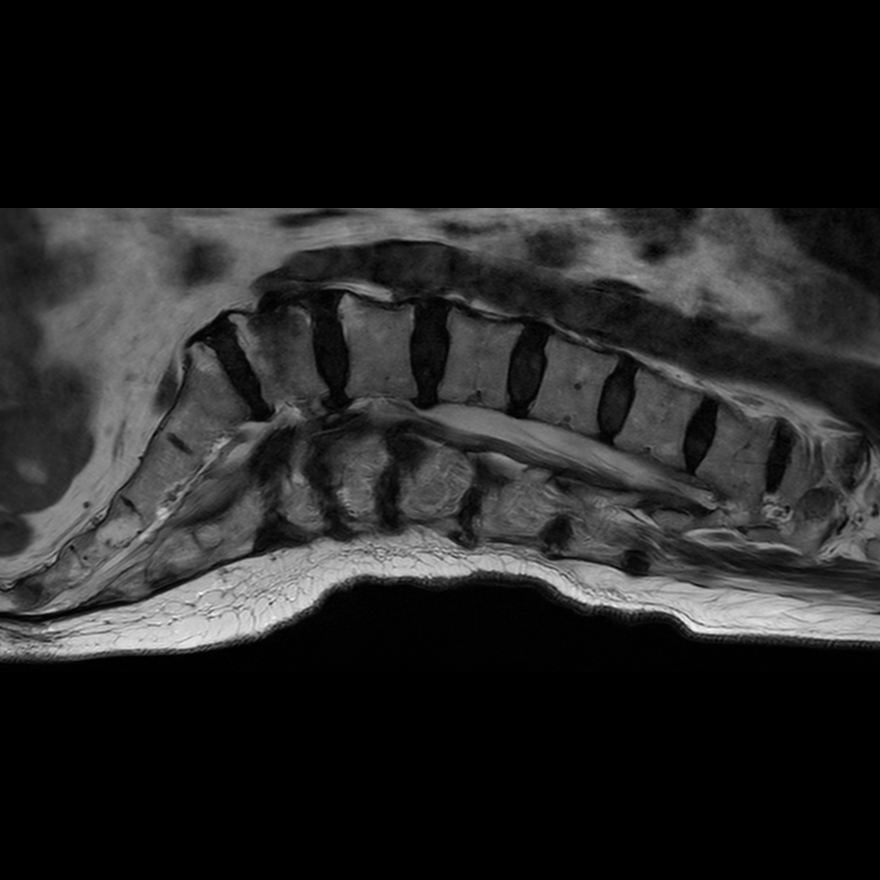

Supplement: S1 File — (ZIP) [file pone.0248303.s001.zip › Code and data/dataset/train/116.png]

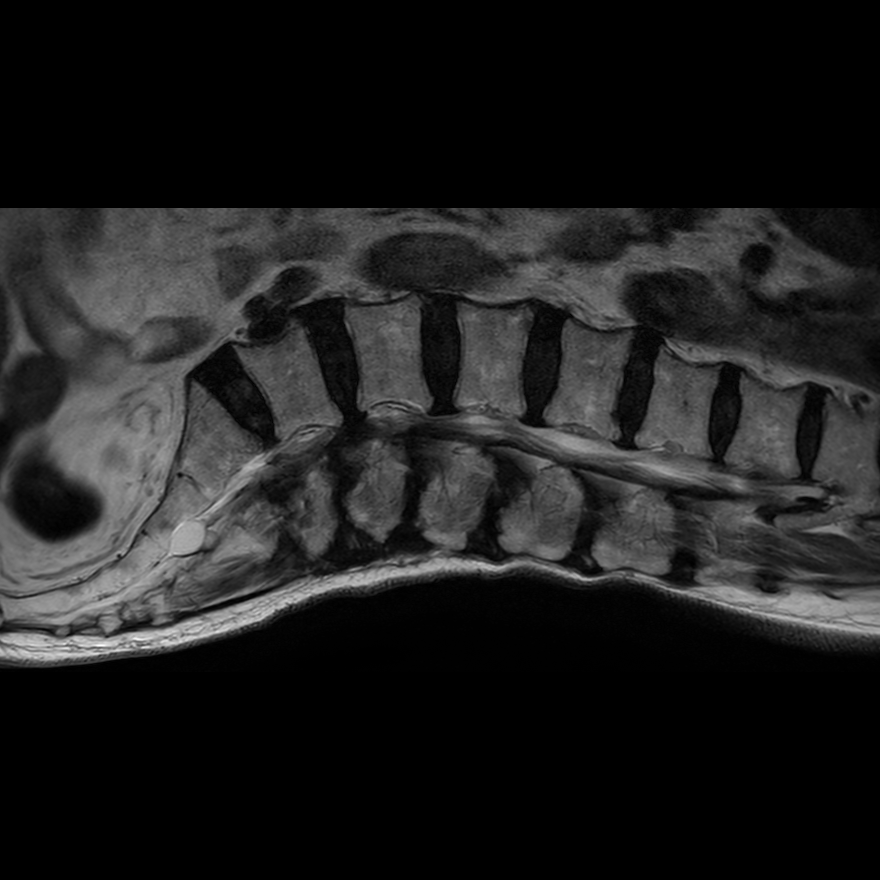

Supplement: S1 File — (ZIP) [file pone.0248303.s001.zip › Code and data/dataset/train/117.png]

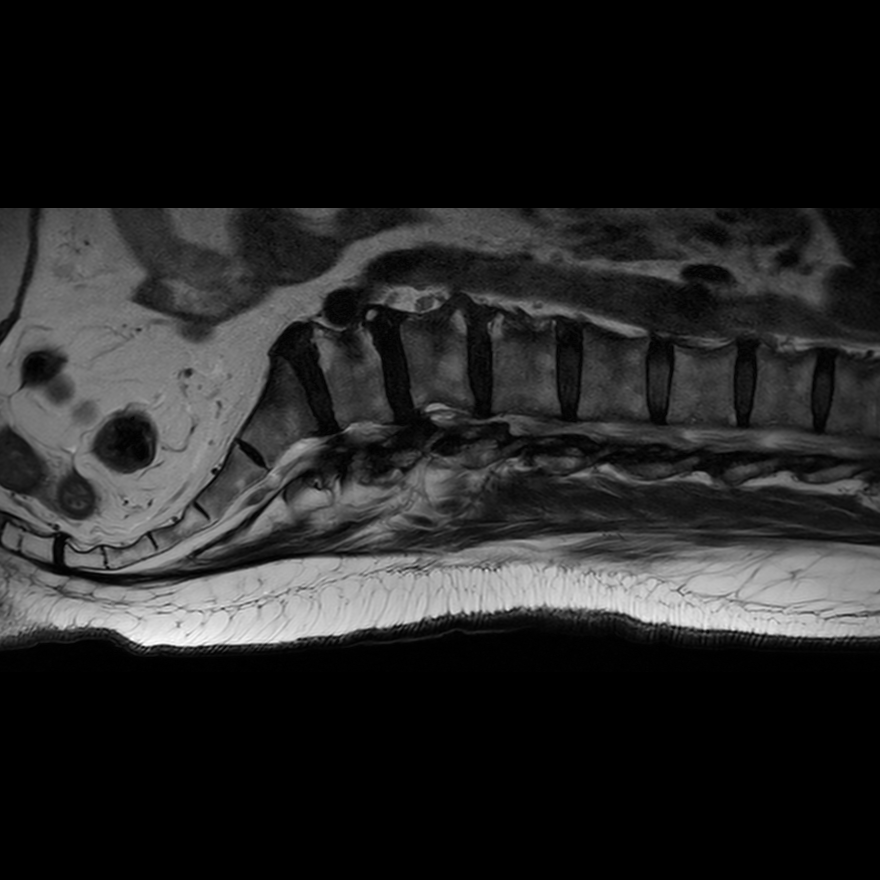

Supplement: S1 File — (ZIP) [file pone.0248303.s001.zip › Code and data/dataset/train/118.png]

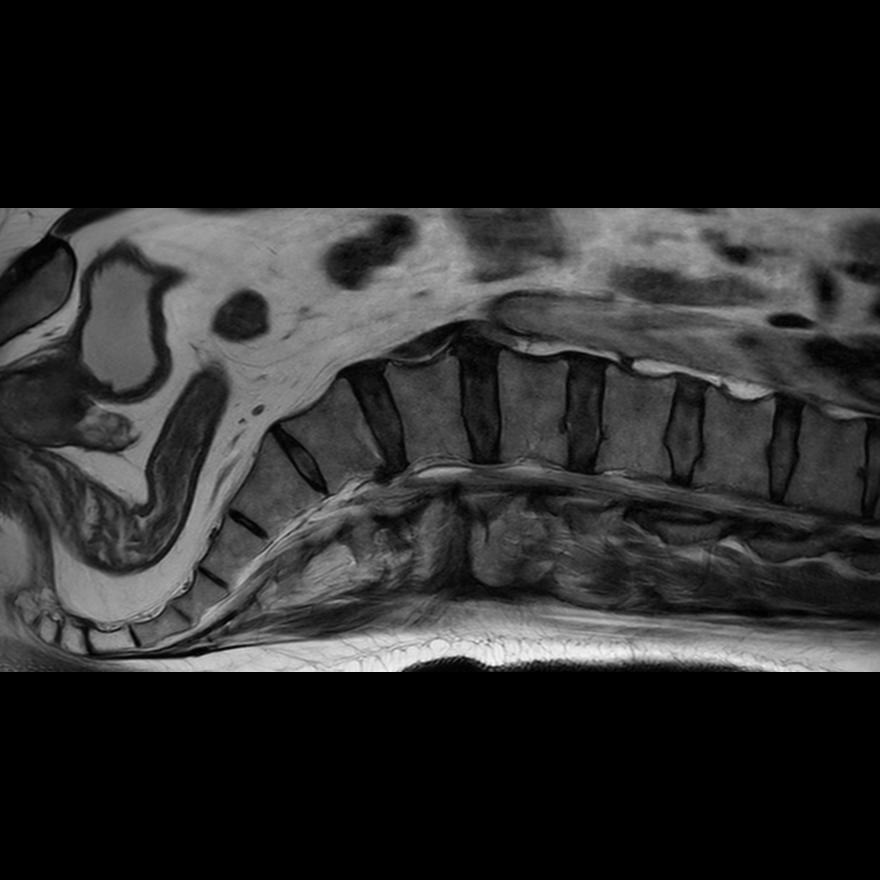

Supplement: S1 File — (ZIP) [file pone.0248303.s001.zip › Code and data/dataset/train/119.png]

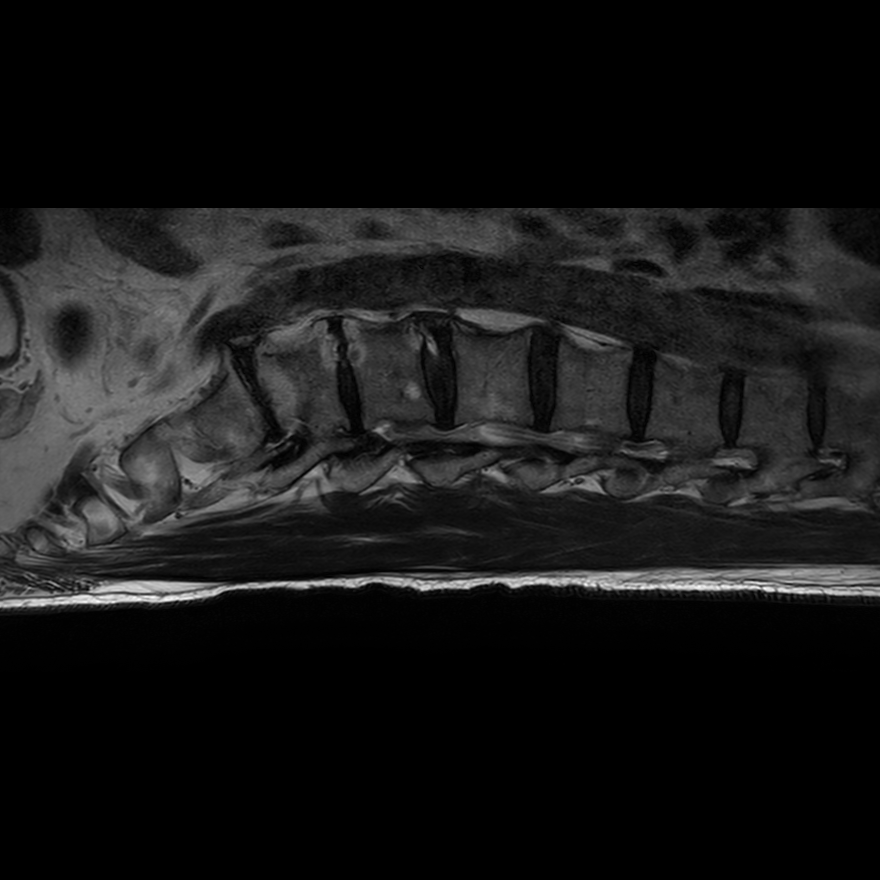

Supplement: S1 File — (ZIP) [file pone.0248303.s001.zip › Code and data/dataset/train/12.png]

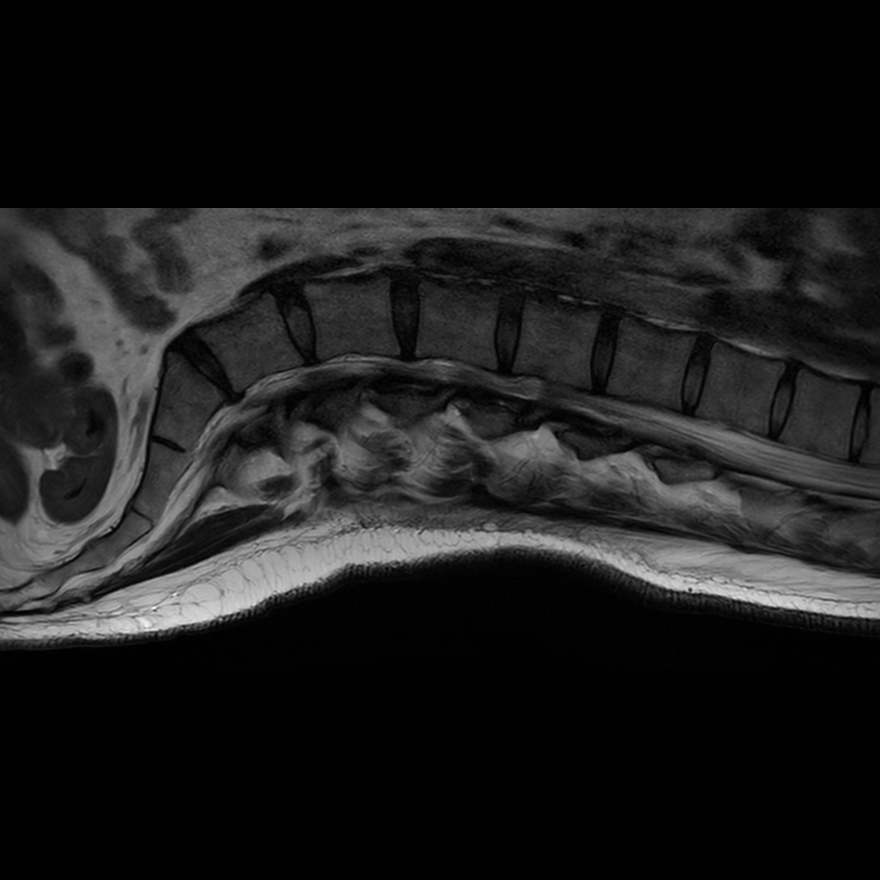

Supplement: S1 File — (ZIP) [file pone.0248303.s001.zip › Code and data/dataset/train/120.png]

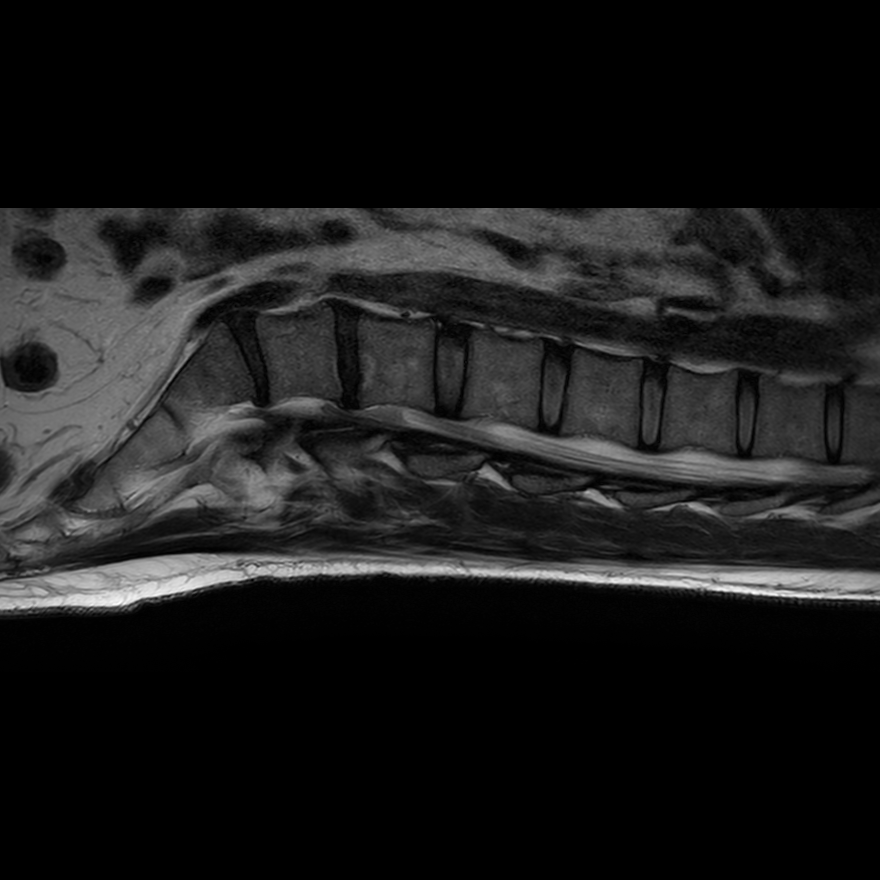

Supplement: S1 File — (ZIP) [file pone.0248303.s001.zip › Code and data/dataset/train/121.png]

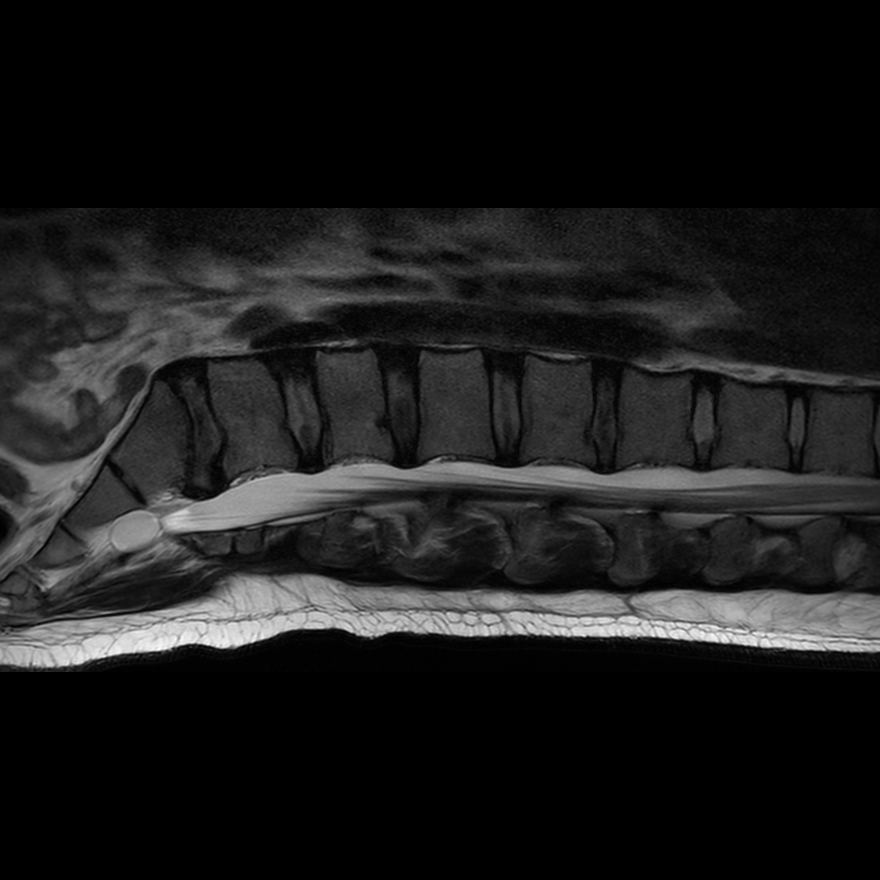

Supplement: S1 File — (ZIP) [file pone.0248303.s001.zip › Code and data/dataset/train/122.png]

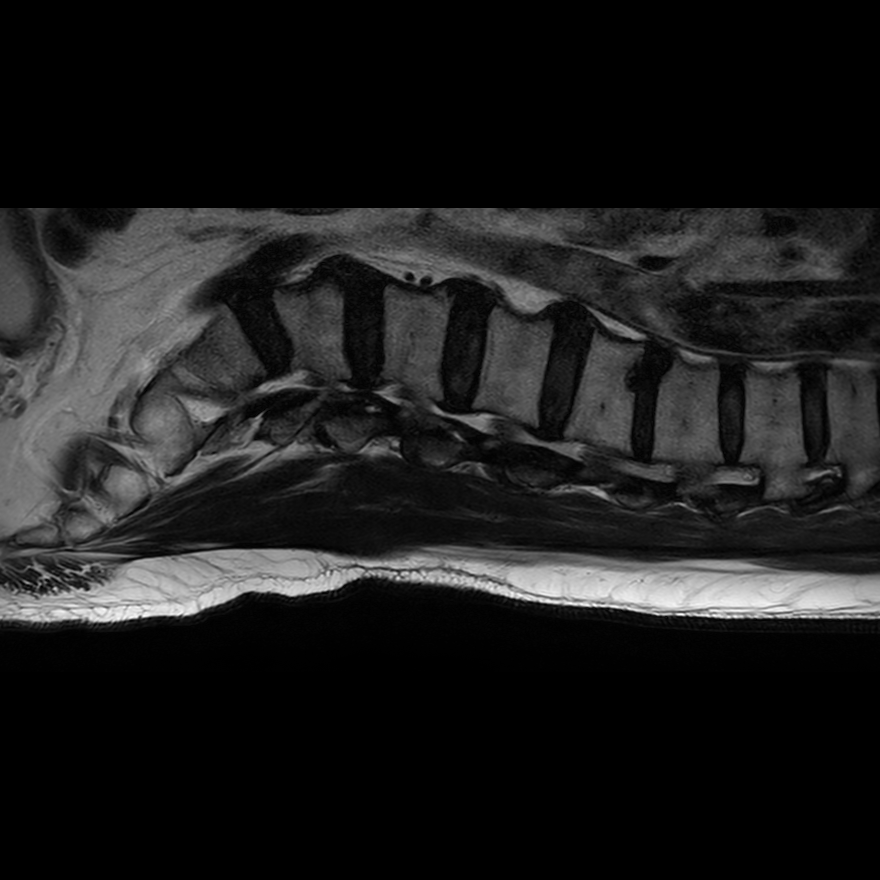

Supplement: S1 File — (ZIP) [file pone.0248303.s001.zip › Code and data/dataset/train/123.png]

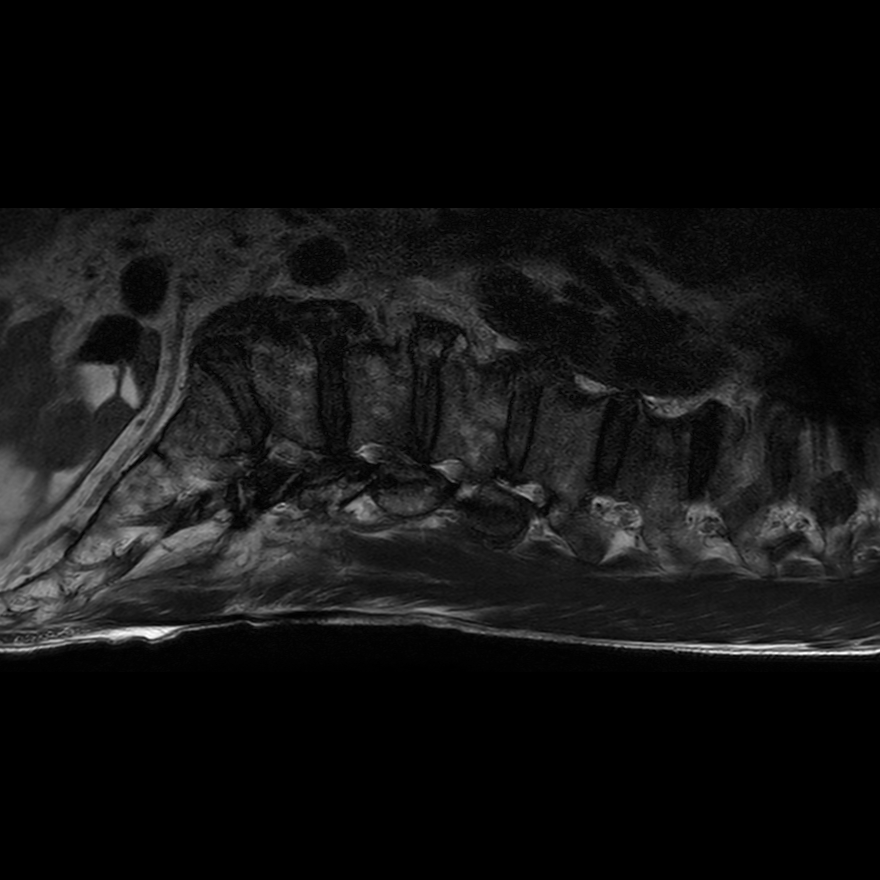

Supplement: S1 File — (ZIP) [file pone.0248303.s001.zip › Code and data/dataset/train/124.png]

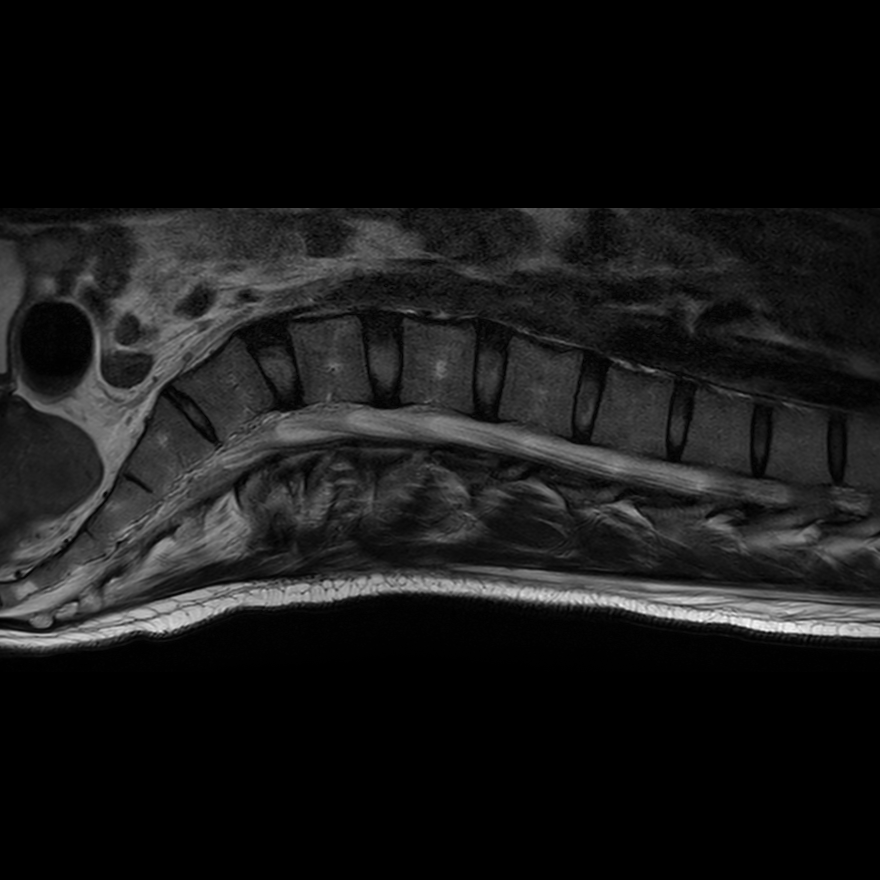

Supplement: S1 File — (ZIP) [file pone.0248303.s001.zip › Code and data/dataset/train/125.png]

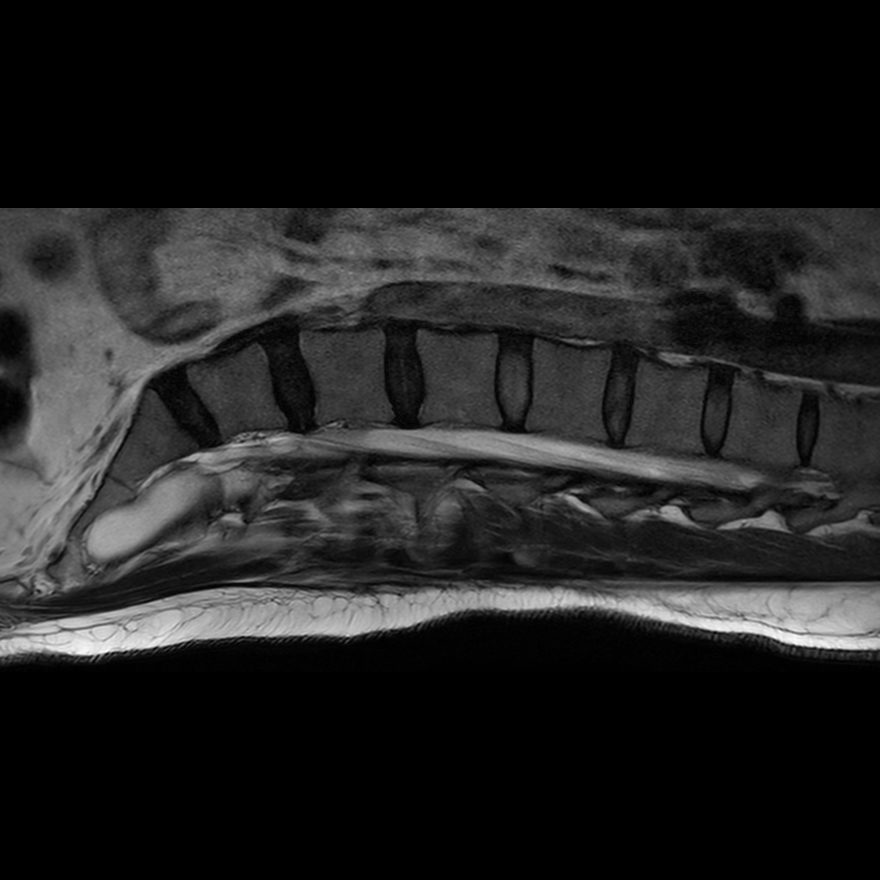

Supplement: S1 File — (ZIP) [file pone.0248303.s001.zip › Code and data/dataset/train/126.png]

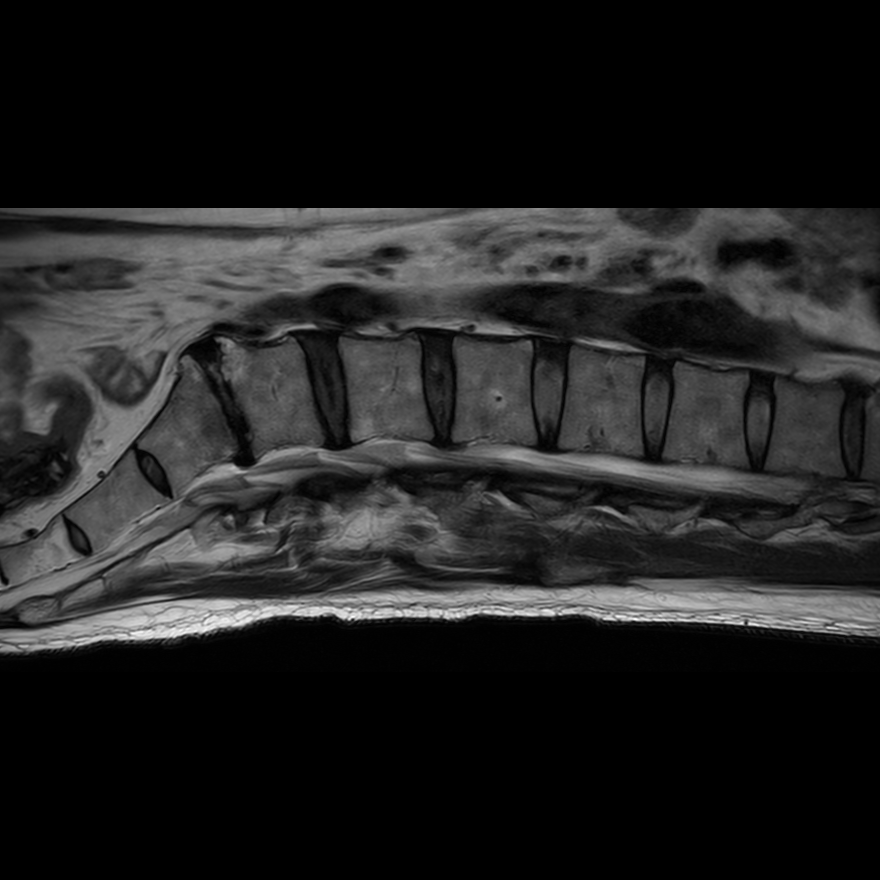

Supplement: S1 File — (ZIP) [file pone.0248303.s001.zip › Code and data/dataset/train/127.png]

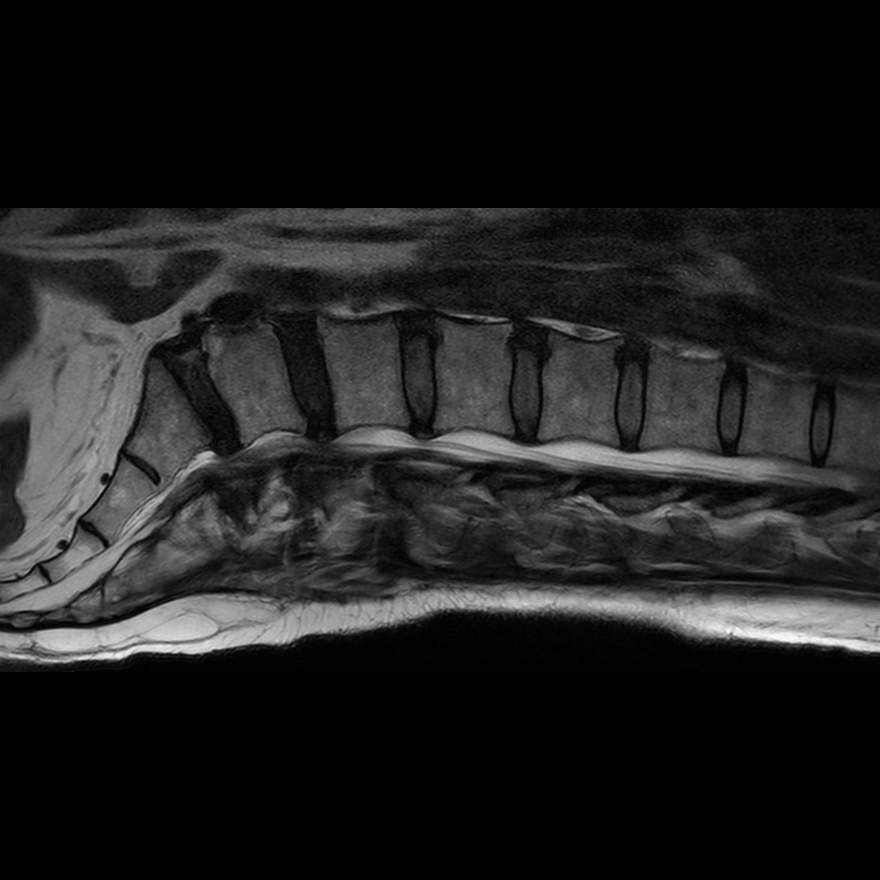

Supplement: S1 File — (ZIP) [file pone.0248303.s001.zip › Code and data/dataset/train/128.png]

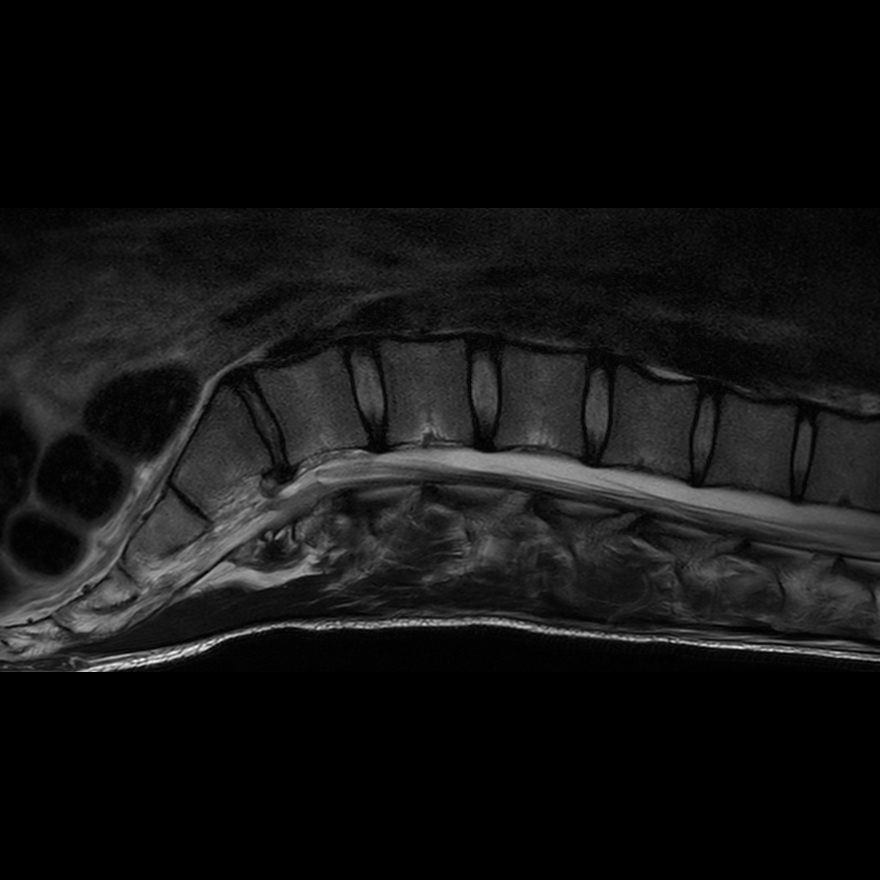

Supplement: S1 File — (ZIP) [file pone.0248303.s001.zip › Code and data/dataset/train/129.png]

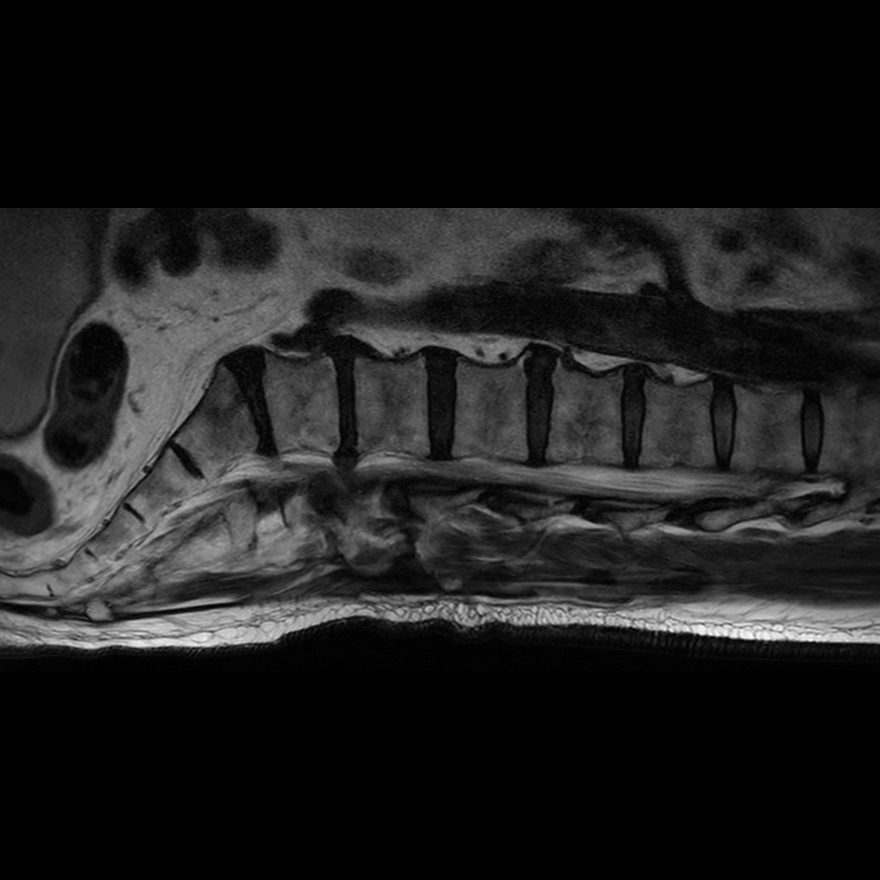

Supplement: S1 File — (ZIP) [file pone.0248303.s001.zip › Code and data/dataset/train/13.png]

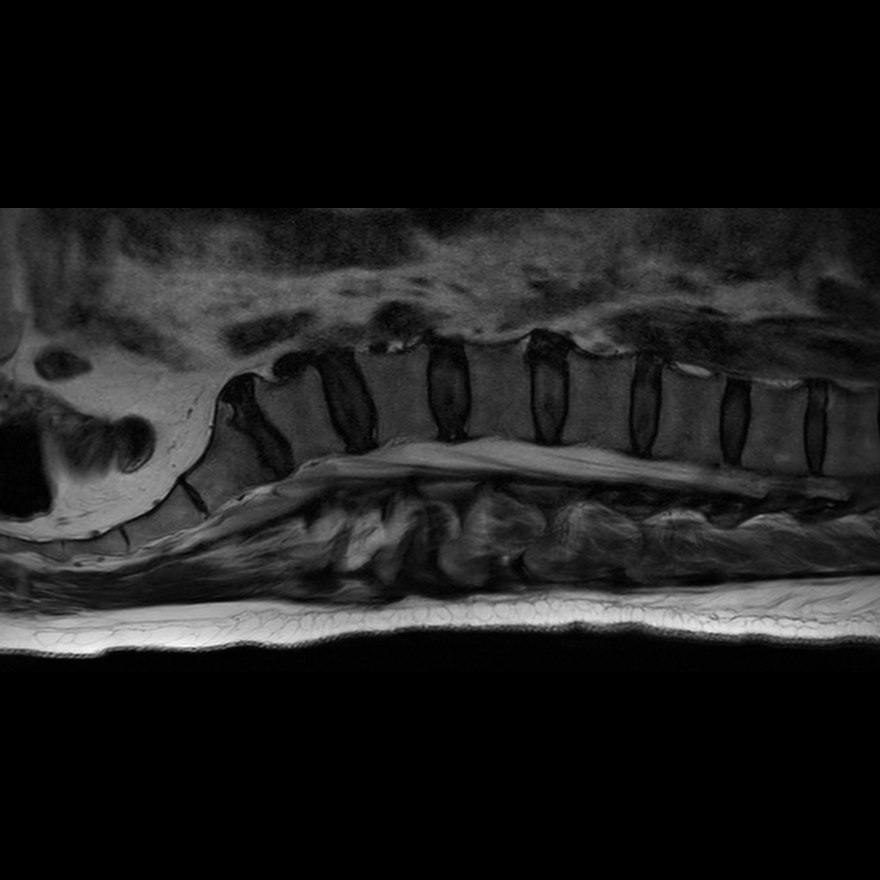

Supplement: S1 File — (ZIP) [file pone.0248303.s001.zip › Code and data/dataset/train/130.png]

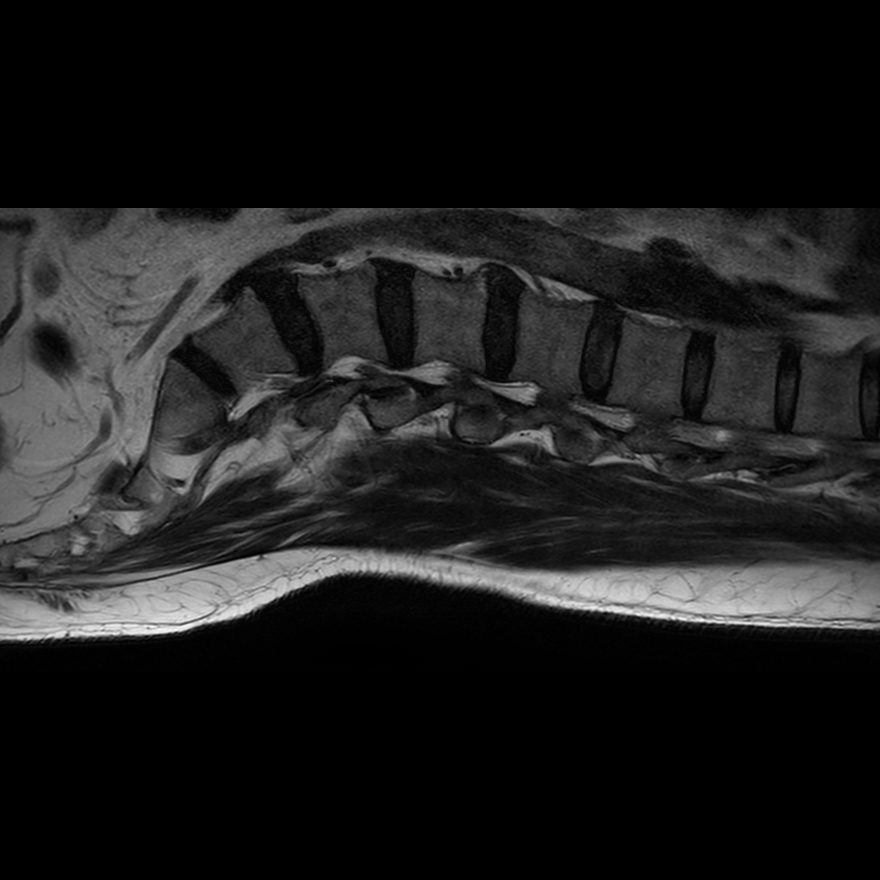

Supplement: S1 File — (ZIP) [file pone.0248303.s001.zip › Code and data/dataset/train/131.png]

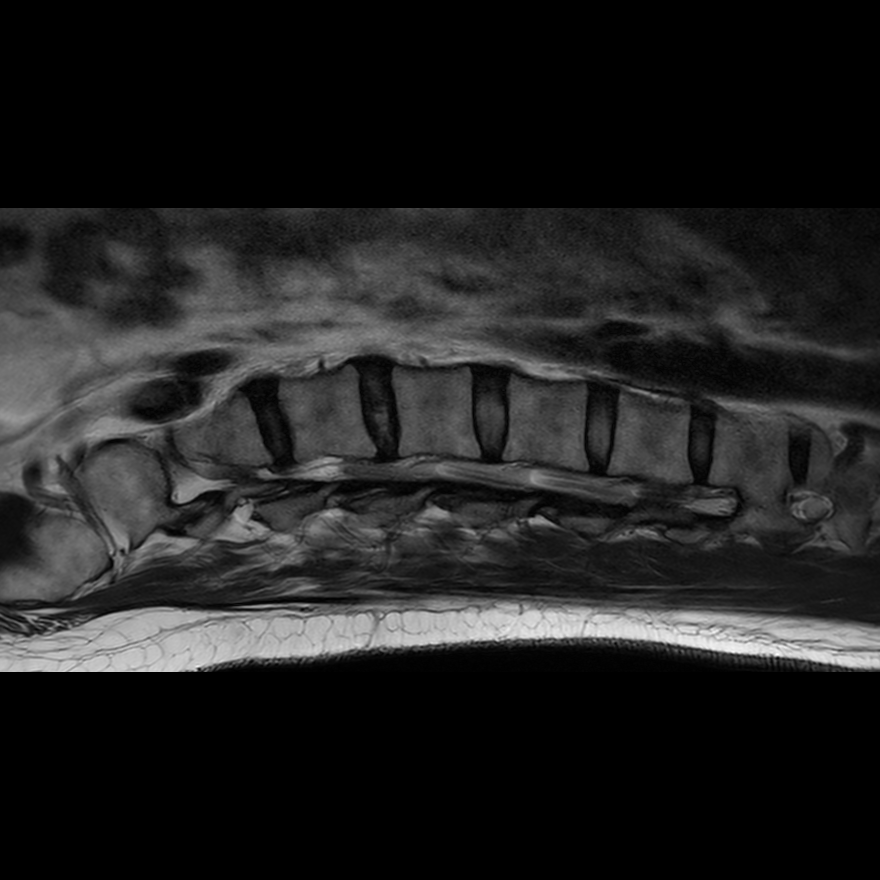

Supplement: S1 File — (ZIP) [file pone.0248303.s001.zip › Code and data/dataset/train/132.png]

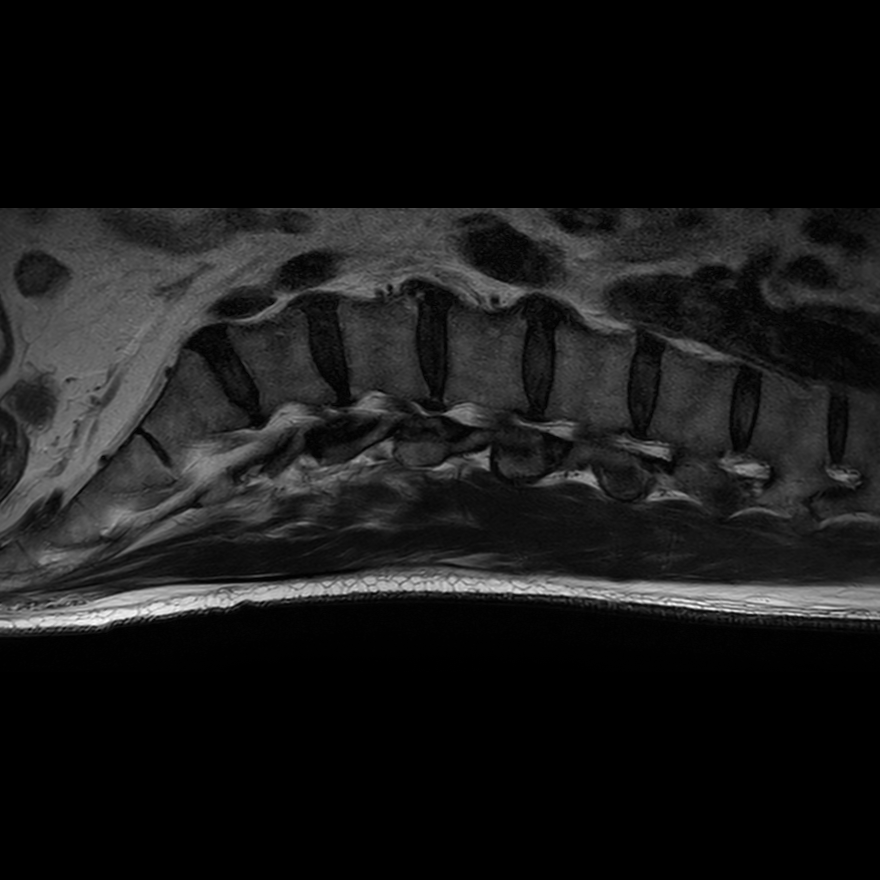

Supplement: S1 File — (ZIP) [file pone.0248303.s001.zip › Code and data/dataset/train/133.png]

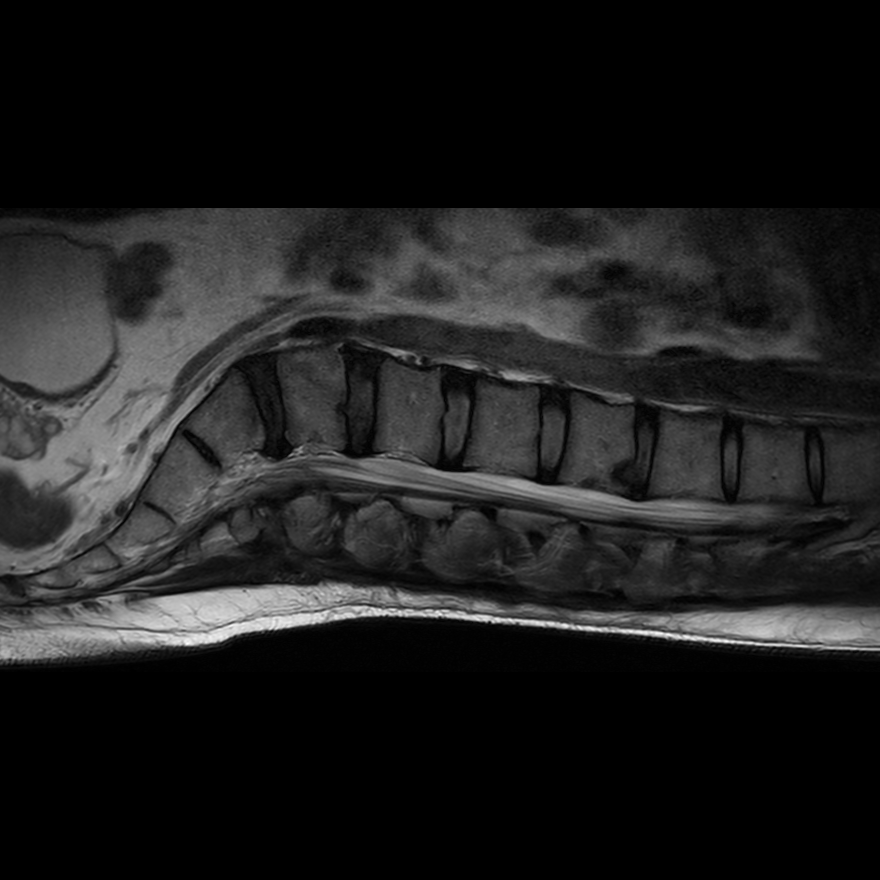

Supplement: S1 File — (ZIP) [file pone.0248303.s001.zip › Code and data/dataset/train/134.png]

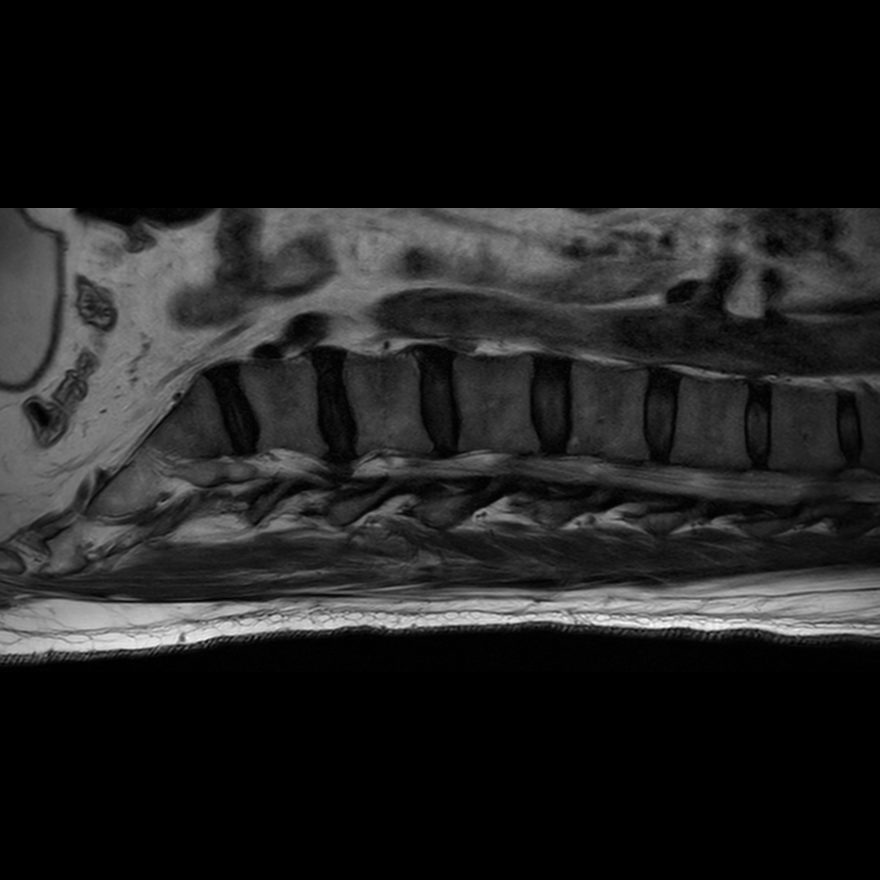

Supplement: S1 File — (ZIP) [file pone.0248303.s001.zip › Code and data/dataset/train/135.png]

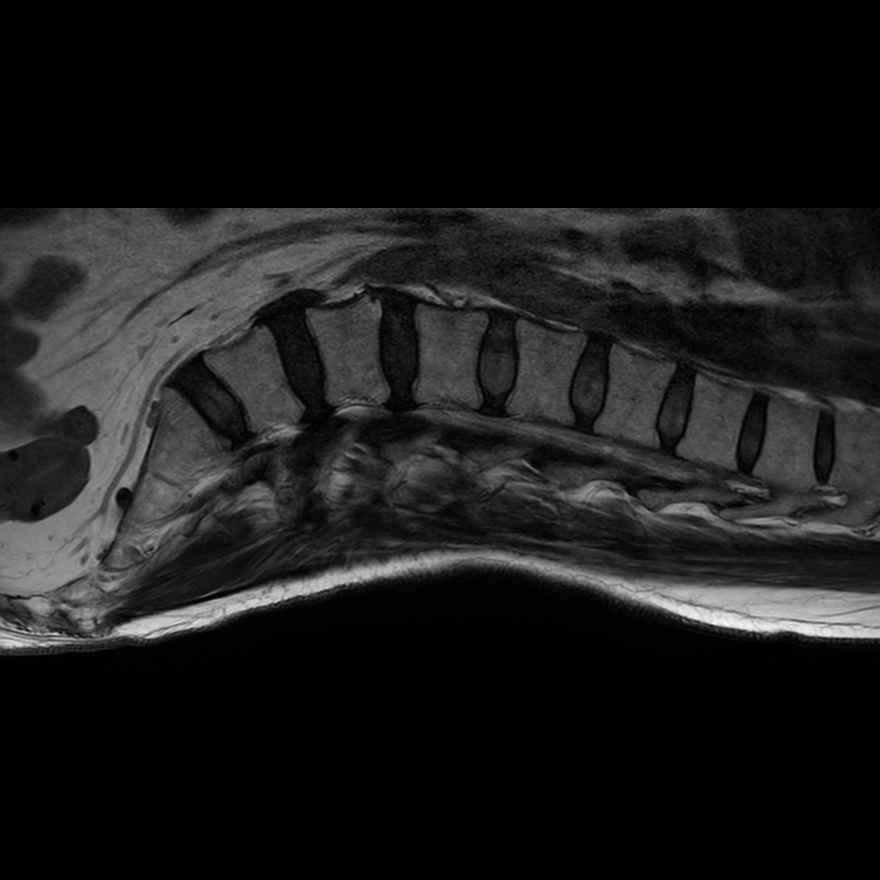

Supplement: S1 File — (ZIP) [file pone.0248303.s001.zip › Code and data/dataset/train/136.png]

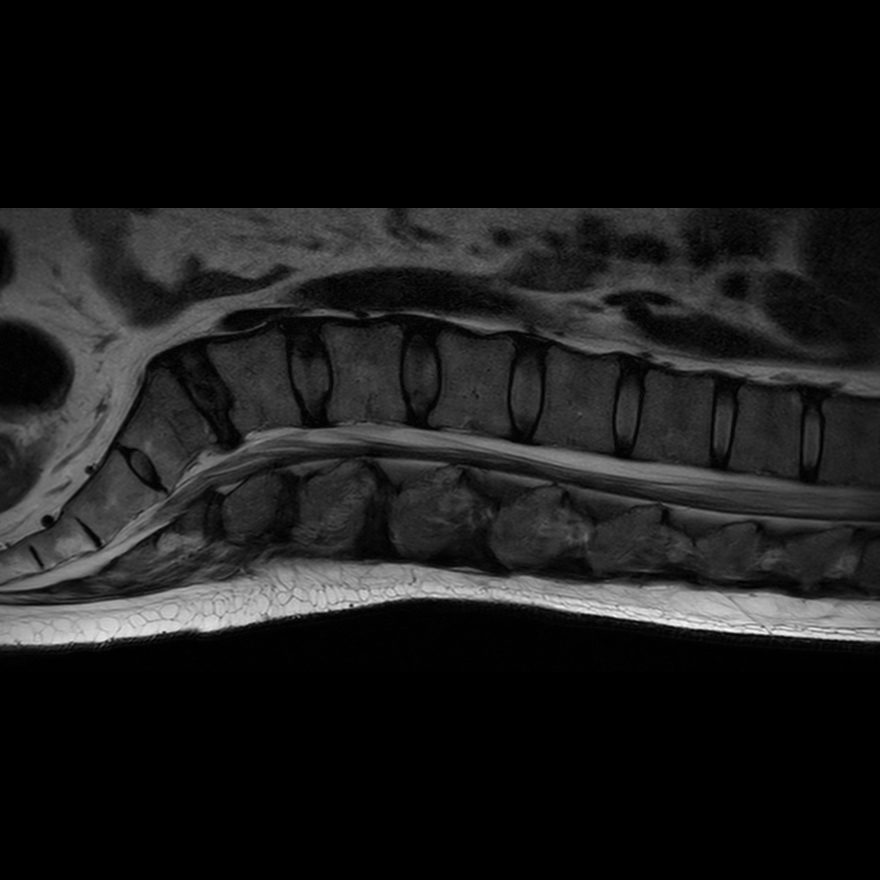

Supplement: S1 File — (ZIP) [file pone.0248303.s001.zip › Code and data/dataset/train/137.png]

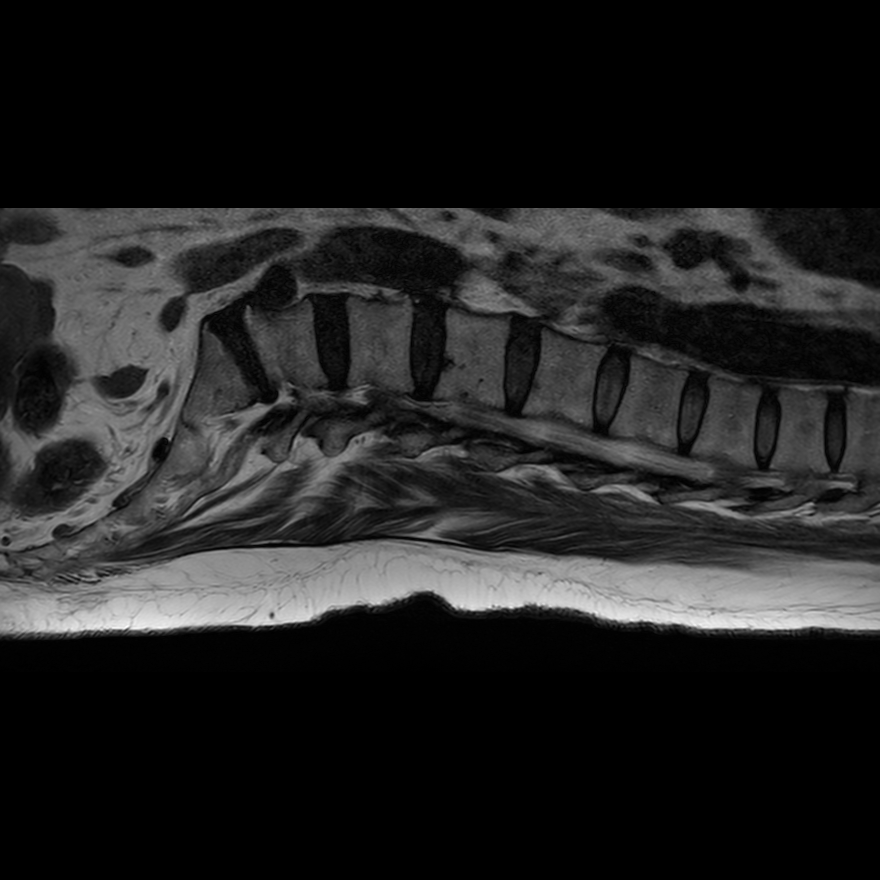

Supplement: S1 File — (ZIP) [file pone.0248303.s001.zip › Code and data/dataset/train/138.png]

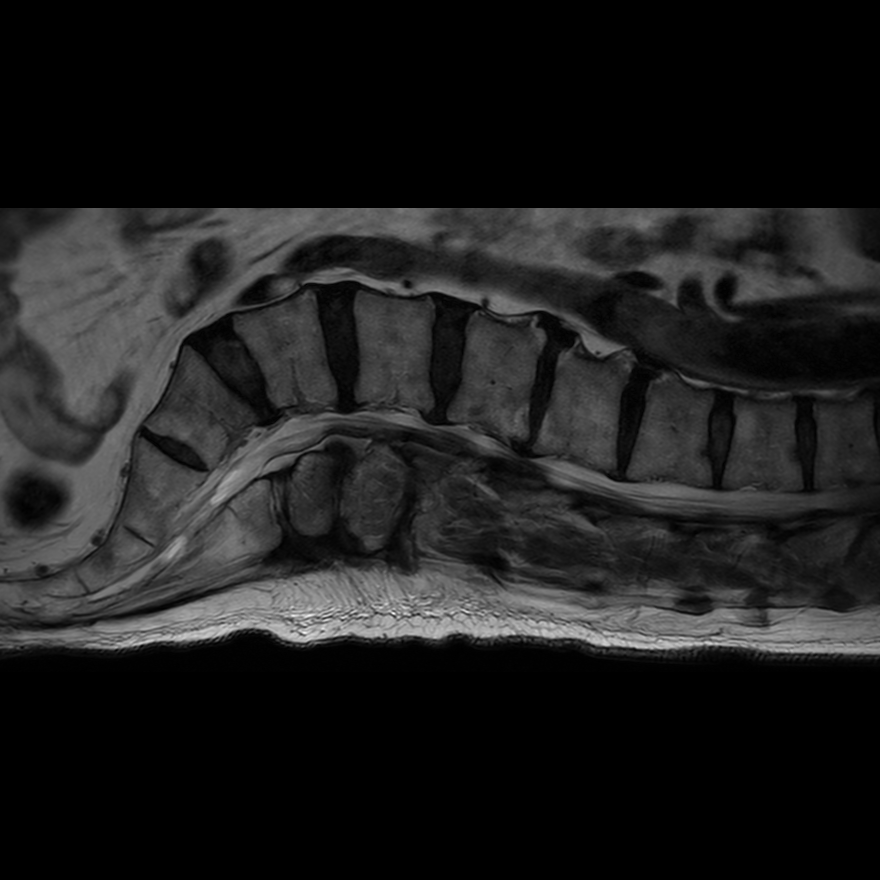

Supplement: S1 File — (ZIP) [file pone.0248303.s001.zip › Code and data/dataset/train/139.png]

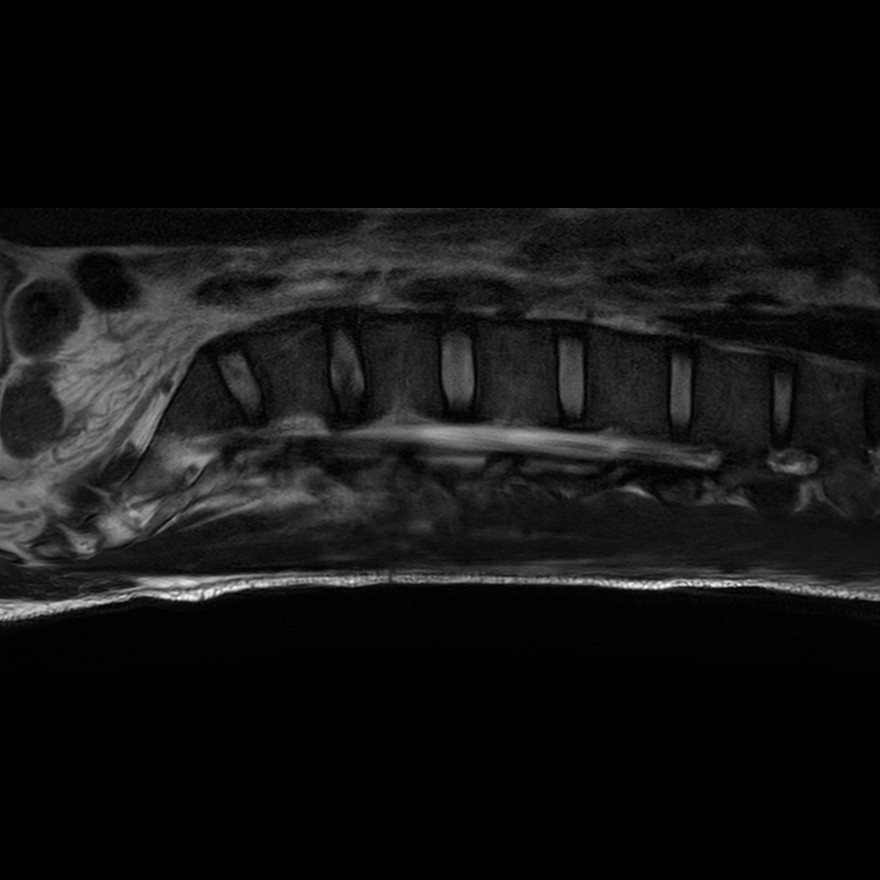

Supplement: S1 File — (ZIP) [file pone.0248303.s001.zip › Code and data/dataset/train/14.png]

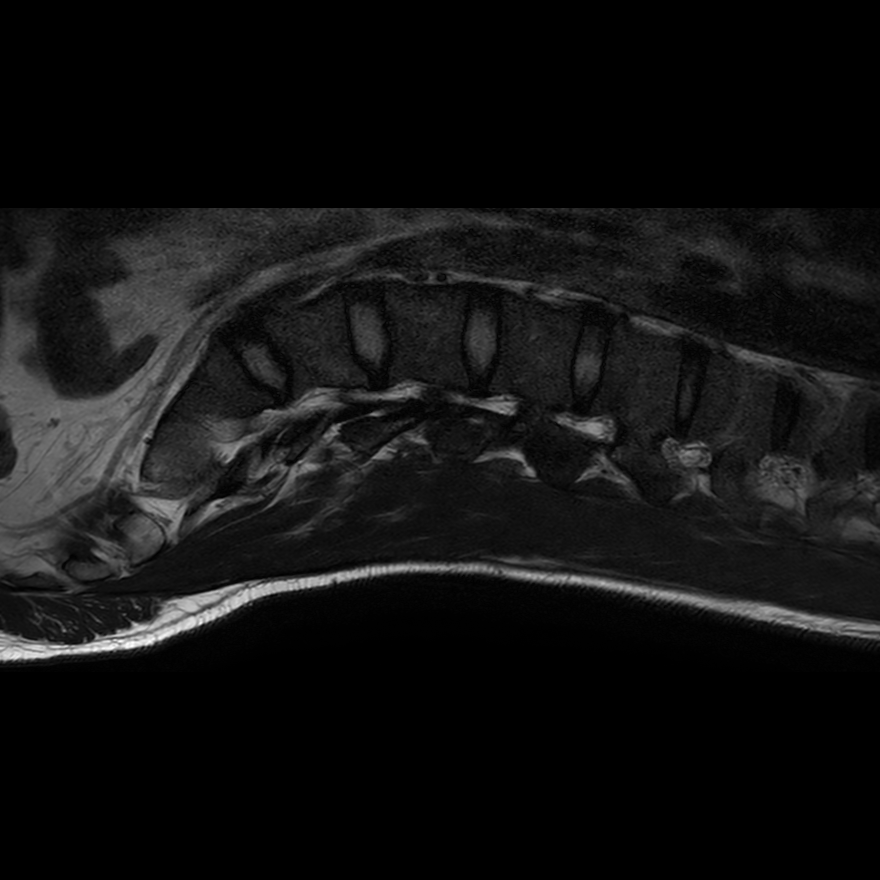

Supplement: S1 File — (ZIP) [file pone.0248303.s001.zip › Code and data/dataset/train/140.png]

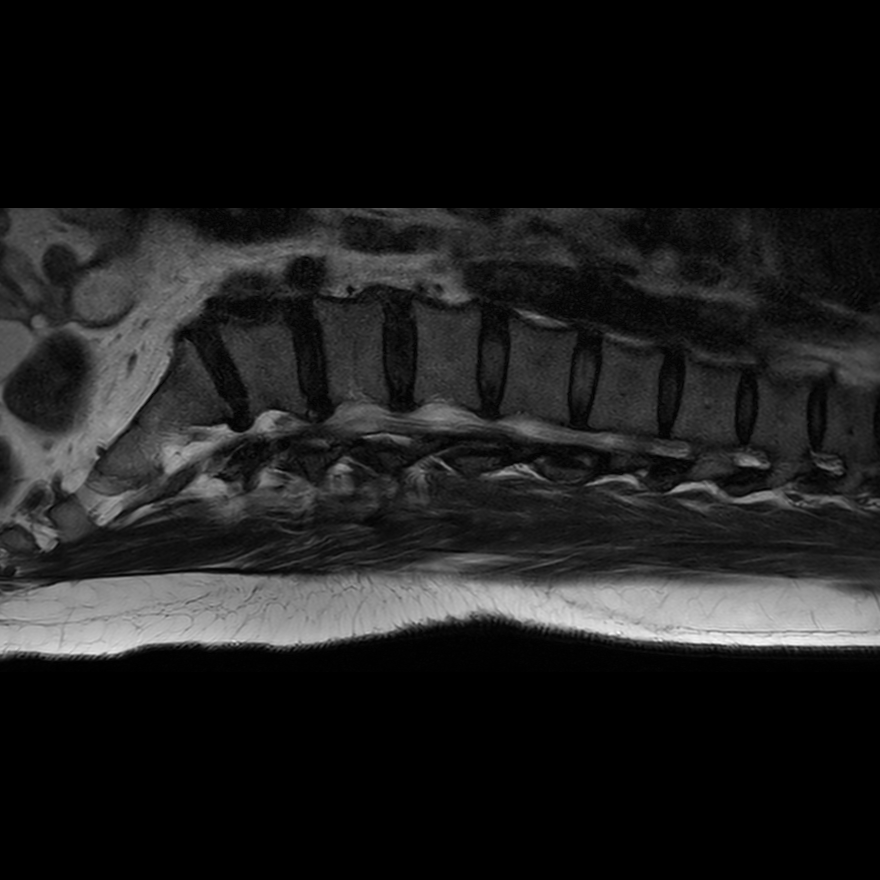

Supplement: S1 File — (ZIP) [file pone.0248303.s001.zip › Code and data/dataset/train/141.png]

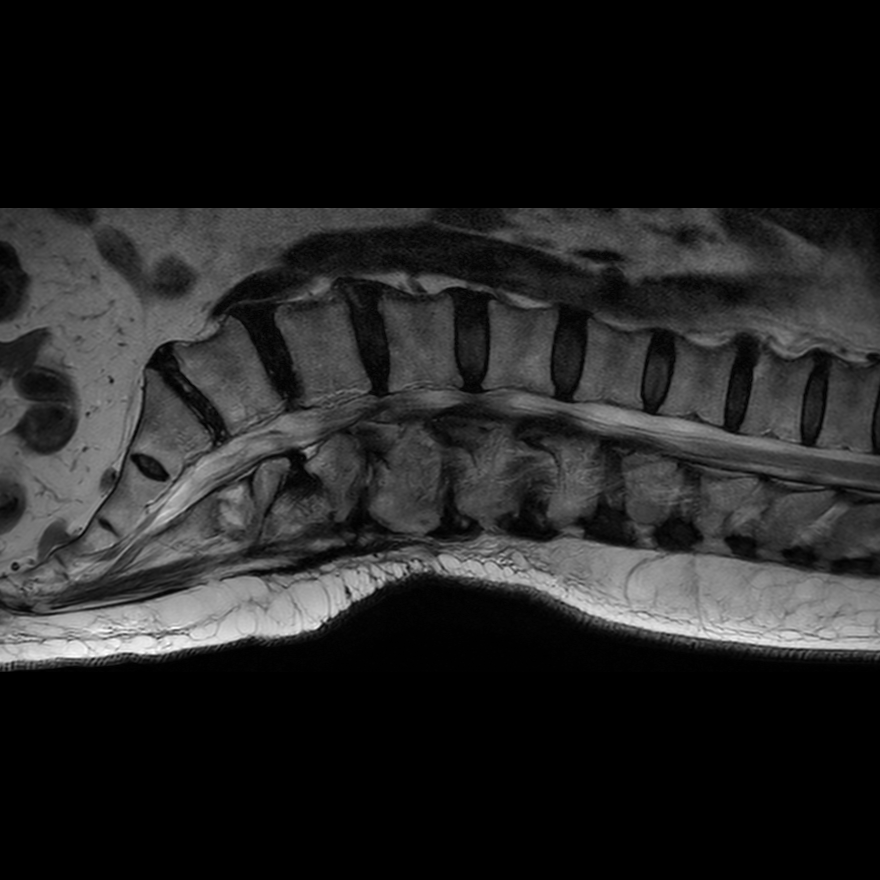

Supplement: S1 File — (ZIP) [file pone.0248303.s001.zip › Code and data/dataset/train/142.png]

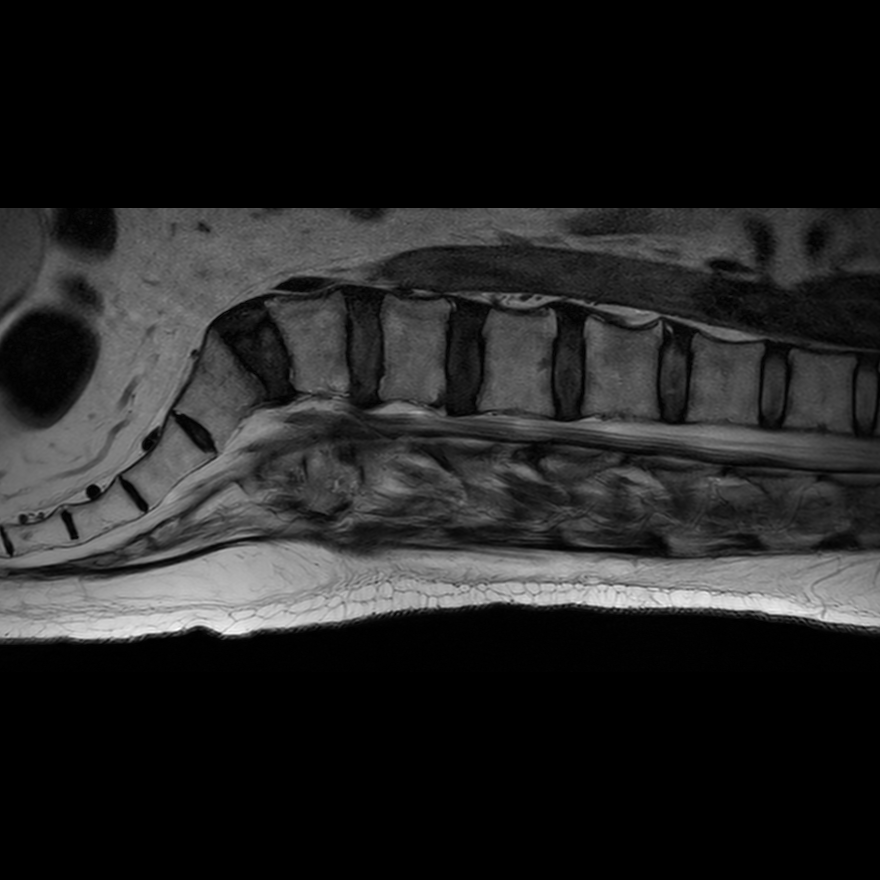

Supplement: S1 File — (ZIP) [file pone.0248303.s001.zip › Code and data/dataset/train/143.png]

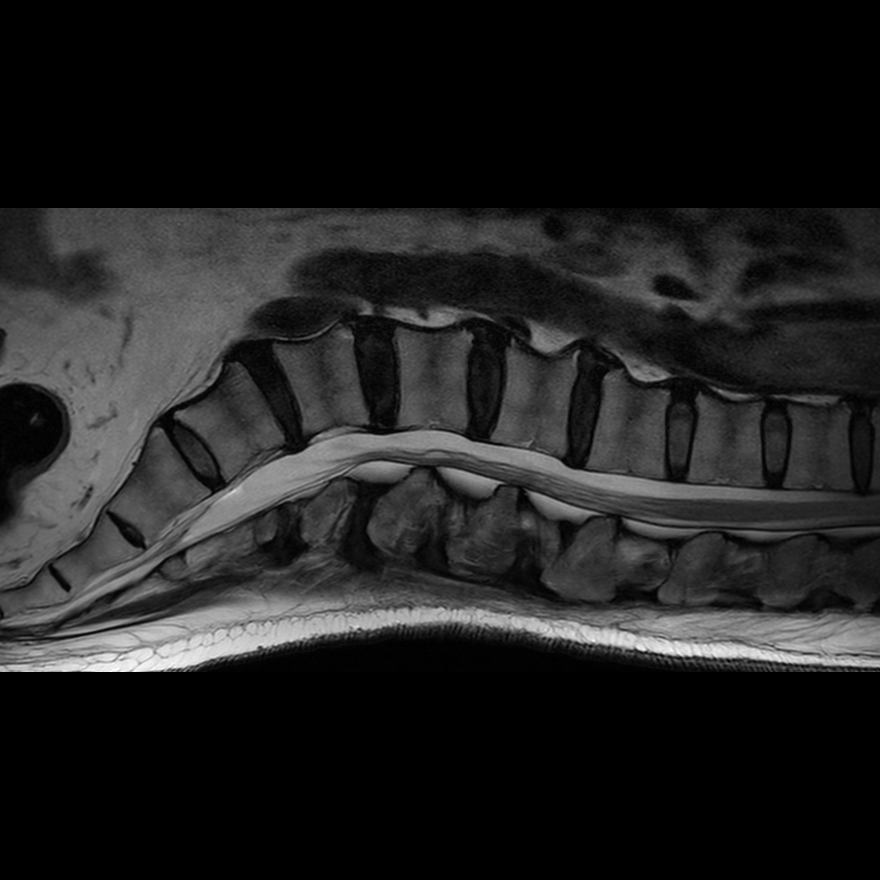

Supplement: S1 File — (ZIP) [file pone.0248303.s001.zip › Code and data/dataset/train/144.png]

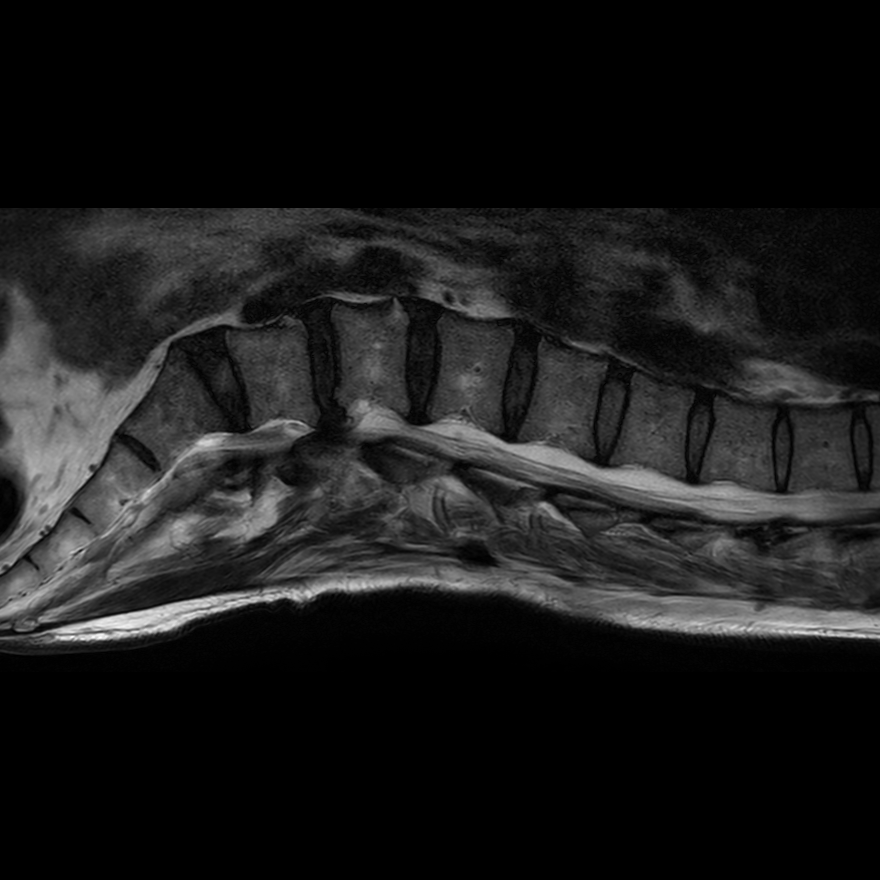

Supplement: S1 File — (ZIP) [file pone.0248303.s001.zip › Code and data/dataset/train/145.png]

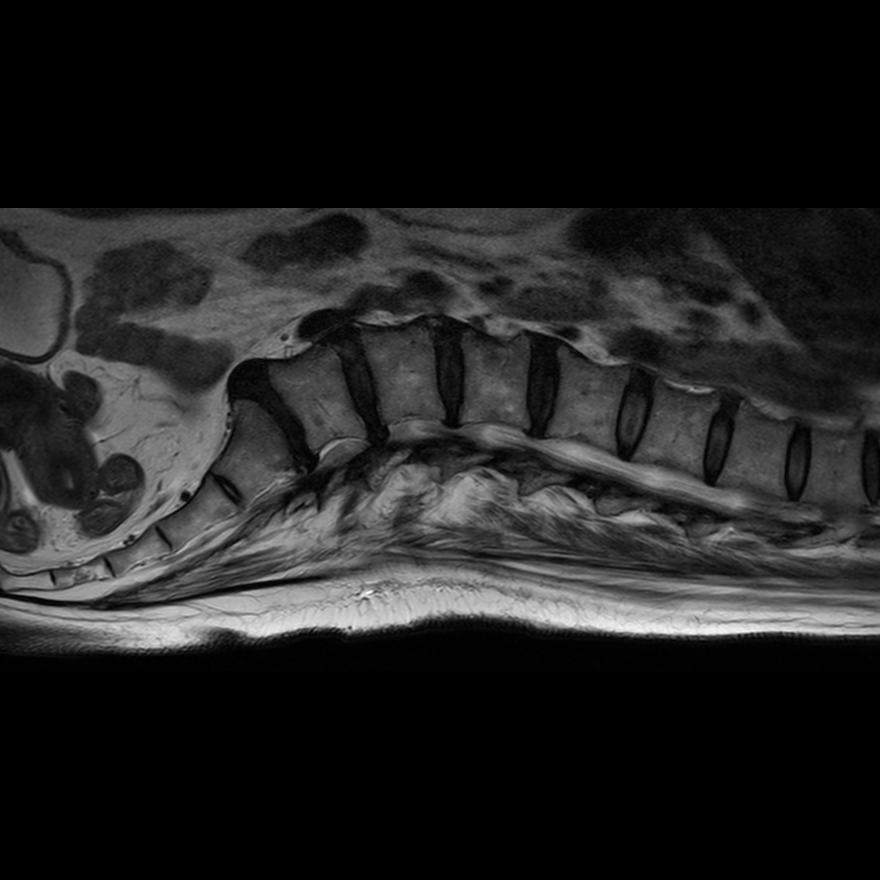

Supplement: S1 File — (ZIP) [file pone.0248303.s001.zip › Code and data/dataset/train/146.png]

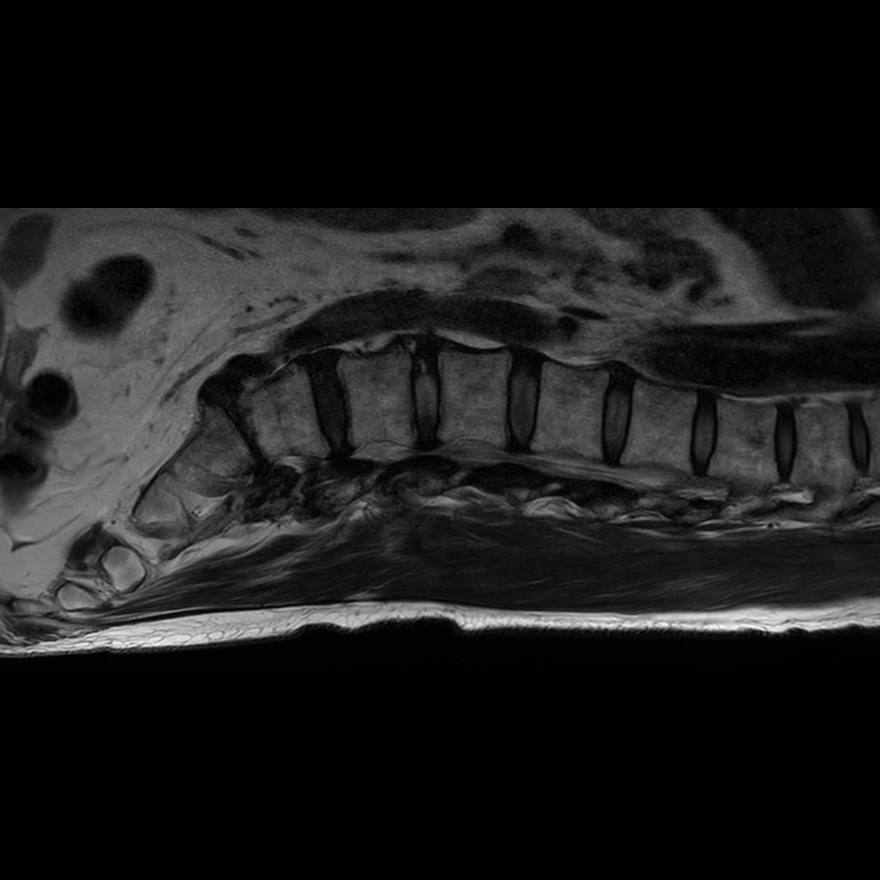

Supplement: S1 File — (ZIP) [file pone.0248303.s001.zip › Code and data/dataset/train/147.png]

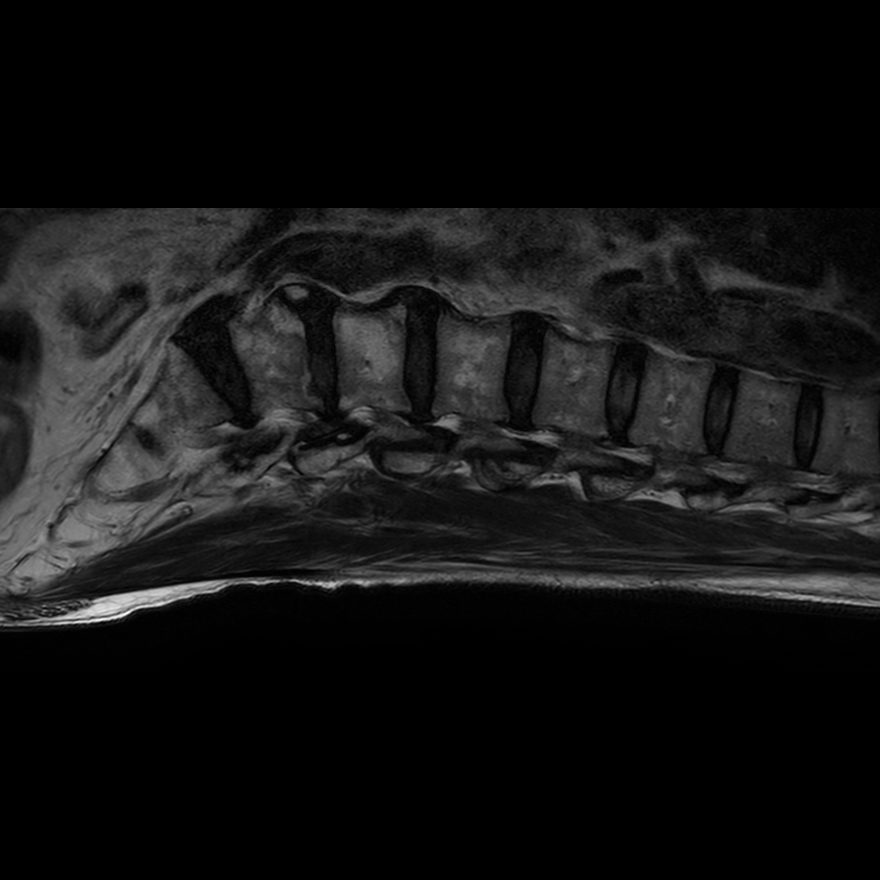

Supplement: S1 File — (ZIP) [file pone.0248303.s001.zip › Code and data/dataset/train/148.png]

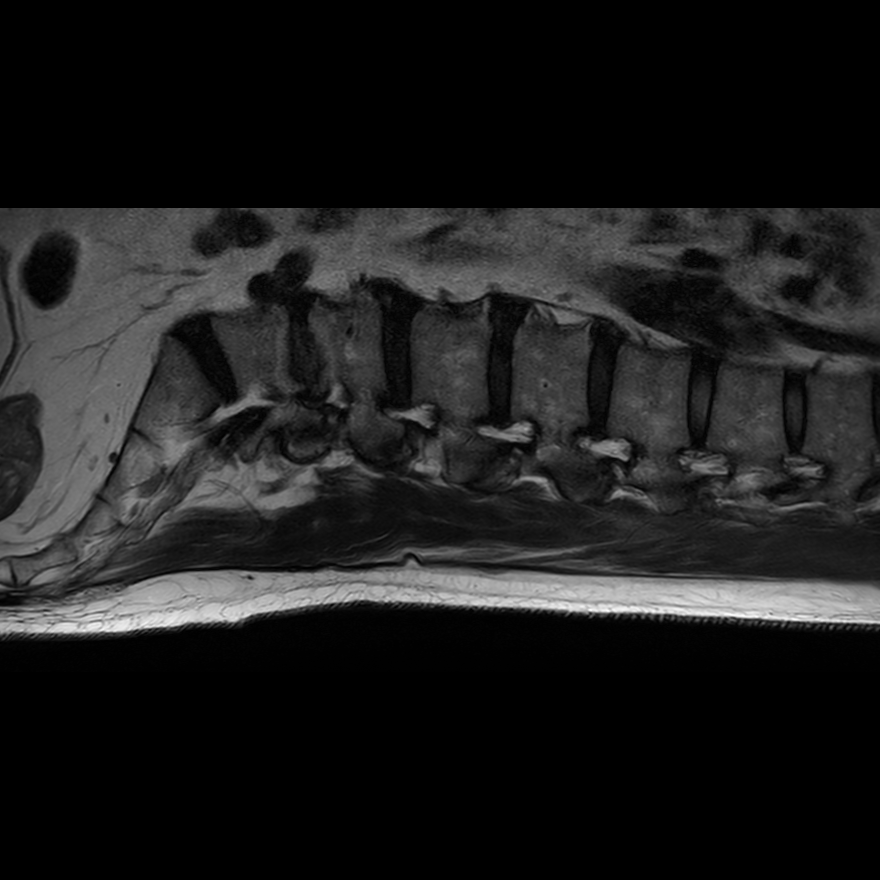

Supplement: S1 File — (ZIP) [file pone.0248303.s001.zip › Code and data/dataset/train/149.png]

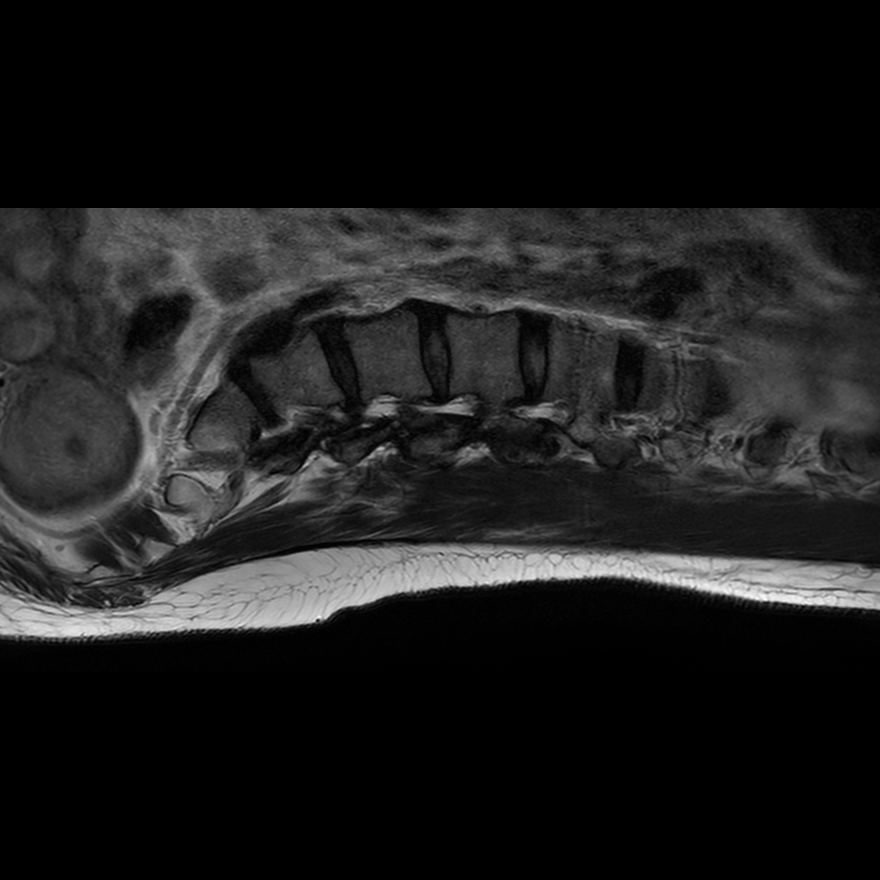

Supplement: S1 File — (ZIP) [file pone.0248303.s001.zip › Code and data/dataset/train/15.png]

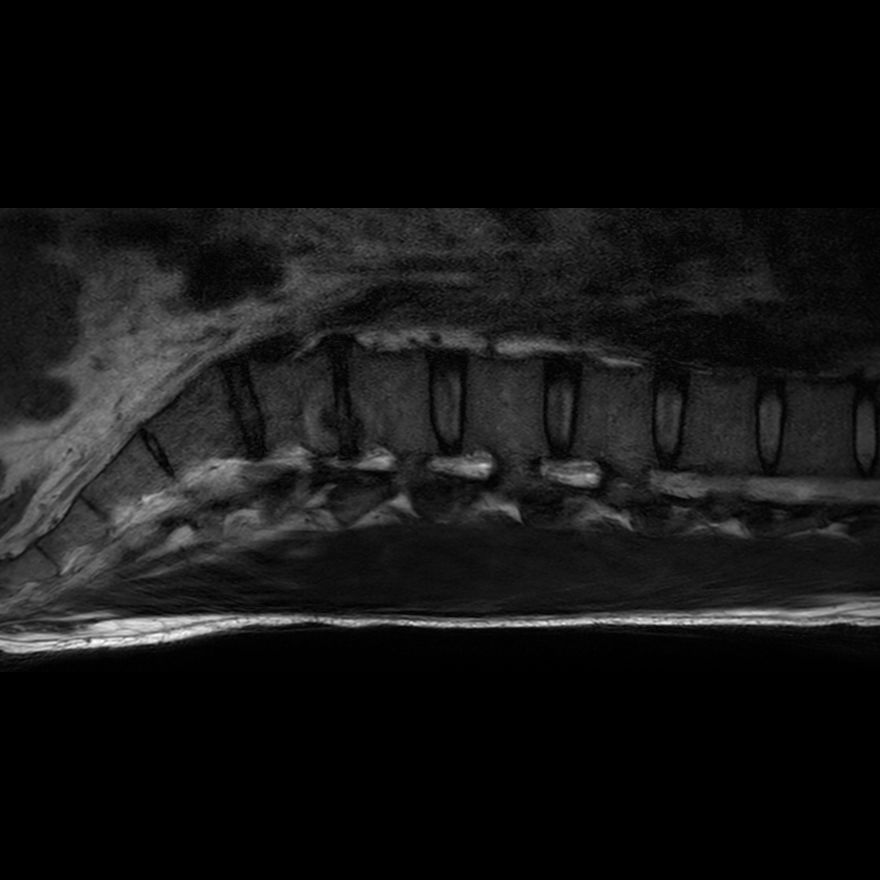

Supplement: S1 File — (ZIP) [file pone.0248303.s001.zip › Code and data/dataset/train/150.png]

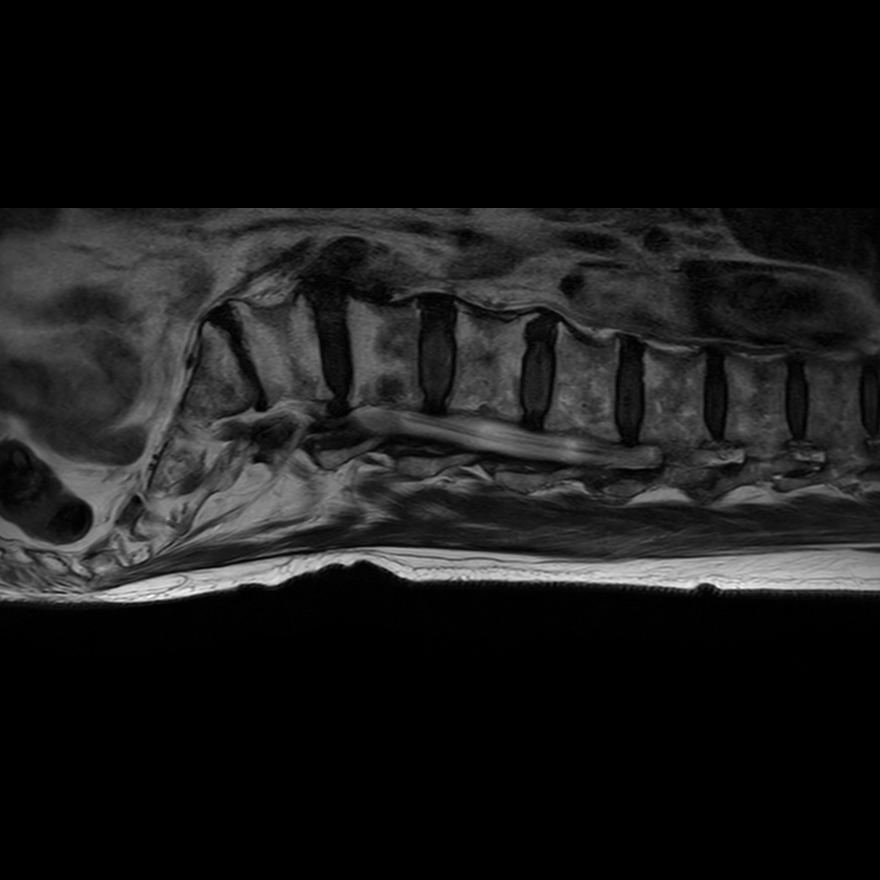

Supplement: S1 File — (ZIP) [file pone.0248303.s001.zip › Code and data/dataset/train/151.png]

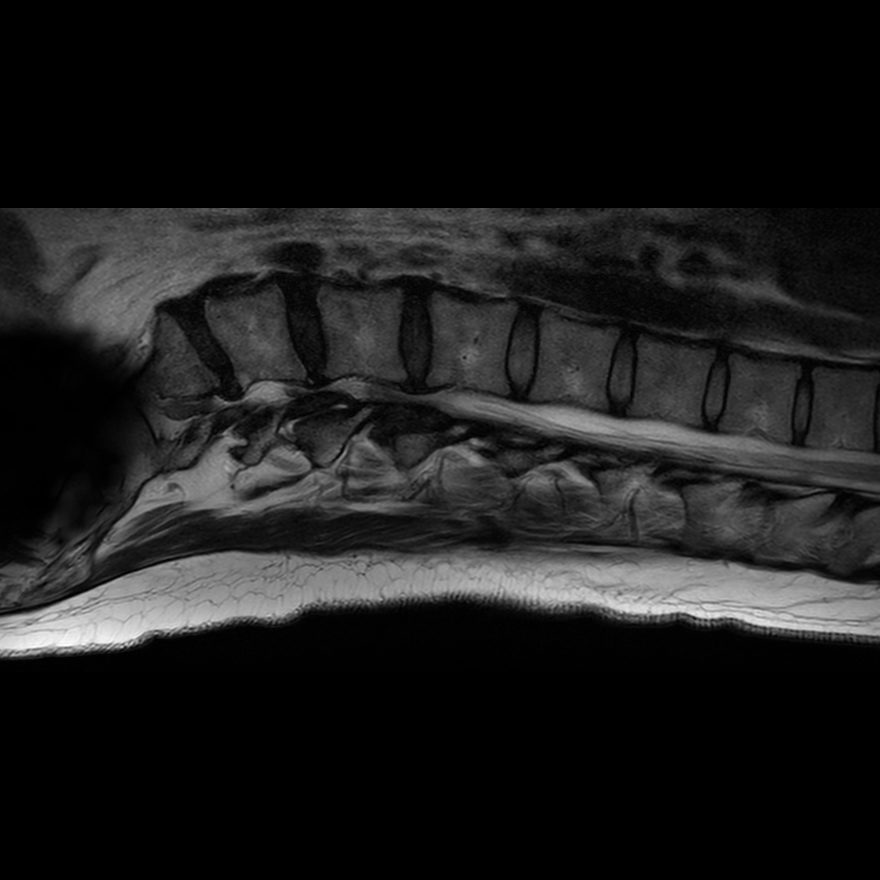

Supplement: S1 File — (ZIP) [file pone.0248303.s001.zip › Code and data/dataset/train/152.png]
